# Stereoselective Synthesis and Investigation of Isopulegol-Based Chiral Ligands

**DOI:** 10.3390/ijms20164050

**Published:** 2019-08-19

**Authors:** Tam Minh Le, Tamás Szilasi, Bettina Volford, András Szekeres, Ferenc Fülöp, Zsolt Szakonyi

**Affiliations:** 1 Institute of Pharmaceutical Chemistry, University of Szeged, Interdisciplinary excellent center, H-6720 Szeged, Eötvös utca 6, Hungary; 2 Stereochemistry Research Group of the Hungarian Academy of Sciences, H-6720 Szeged, Eötvös utca 6, Hungary; 3 Department of Microbiology, University of Szeged, 6726 Szeged, Közép fasor 52, Hungary; 4 Interdisciplinary Centre of Natural Products, University of Szeged, H-6720 Szeged, Eötvös utca 6, Hungary

**Keywords:** aminodiols, aminotriols, diols, triols, tetraols, chiral catalysts, antimicrobial activity

## Abstract

A library of isopulegol-based bi-, tri- and tetrafunctional chiral ligands has been developed from commercially available (−)-isopulegol and applied as chiral catalysts in the addition of diethylzinc to benzaldehyde. Michael addition of primary amines towards *α*-methylene-*γ*-butyrolactone, followed by reduction, was accomplished to provide aminodiols in highly stereoselective transformations. Stereoselective epoxidation of (+)-neoisopulegol, derived from natural (−)-isopulegol, and subsequent oxirane ring opening with primary amines afforded aminodiols. The regioselective ring closure of *N*-substituted aminodiols with formaldehyde was also investigated. Hydroxylation of (+)-neoisopulegol resulted in diol, which was then transformed into aminotriols by aminolysis of its epoxides. Dihydroxylation of (+)-neoisopulegol or derivatives with OsO_4_/NMO gave neoisopulegol-based di-, tri- and tetraols in highly stereoselective reactions. The antimicrobial activity of aminodiol and aminotriol derivatives as well as di-, tri- and tetraols was also explored. In addition, structure–activity relationships were examined by assessing substituent effects on the aminodiol and aminotriol systems.

## 1. Introduction

In recent years, the discovery of aminodiols and their applications as building moieties of complex bioactive molecules have attracted significant attention due to their biological activities. The aminodiol moieties possess a cardiovascular, cytostatic, and antiviral effect [1]. For example, aristeromycin, first isolated from *Streptomyces citricolor*, and its modified derivatives belong to an important group of carbocyclic nucleosides that exhibit a wide range of pharmacological properties such as antiviral, anticancer and antitoxoplasma activities. Aristeromycin analogues, in particular, are widely used as antiviral agents against a range of viruses, including the human immunodeficiency, hepatitis B, herpes simplex, varicella-zoster, influenza and hepatitis C viruses [2,3,4]. (2*R*,3*R*,7*Z*)-2-Aminotetradec-7-ene-1,3-diol, a new sphingosine derivative of the Caribbean sponge *Haliclona vansoesti*, is a potent antimicrobial metabolite [5]. The Abbott aminodiol, found to be a useful building block for the synthesis of the potent renin inhibitor Zankiren^®^, and Enalkiren^®^, was introduced into the therapy of hypertension [6,7]. Aminodiols can also exert antidepressive activity. For example, (*S*,*S*)-reboxetine, a selective norepinephrine reuptake inhibitor, was approved in many countries for the treatment of unipolar depression [8], while some aminodiols have been investigated as selective antagonists on receptor P2X_1_ [9]. Other aminodiols may serve as starting materials for the synthesis of biologically active natural compounds. For example, cytoxazone, a microbial metabolite isolated from *Streptomyces* species, is a selective modulator of the secretion of T_H_2 cytokine [10,11]. Some bicyclic aminodiol-based carbocyclic nucleoside analogues exert antiviral activity [12].

Besides their biological interest, aminodiols have also been applied as starting materials in asymmetric syntheses or as chiral auxiliaries and ligands in enantioselective transformations [13]. To develop new, efficient and commercially available chiral catalysts, chiral natural products including (+)- and (−)-*α*-pinene [14,15], (+)-carene [16,17], (-)-menthone [18], (−)-fenchone [19], (+)-sabinol [20], (−)-nopinone [21] or (−)-pulegone [22] can serve as important starting materials for the synthesis of aminodiols. Monoterpene-based aminodiols have been demonstrated to be excellent chiral auxiliaries in a wide range of stereoselective transformations including intramolecular radical cyclisation [23], intramolecular [2+2] photocycloaddition [24] and Grignard addition [25,26].

Monoterpene-based diols or triols have also proved to be good chiral auxiliaries and catalysts [27,28]. They also possess marked biological properties; e.g., antimicrobial, antifungal or enzyme inhibitor activities [29,30,31].

In the present contribution, we report the preparation of a new library of isopulegol-based chiral bi-, tri- and tetrafunctional synthons, such as aminodiols, aminotriols, di-, tri- and tetraols, starting from commercially available natural (−)-isopulegol. Our study also involved the evaluation of the resulting ligands as catalysts in the asymmetric transformation and antimicrobial activity on multiple bacterial and fungal strains of new isopulegol derivatives.

## 2. Results

### 2.1. Synthesis of (−)-α-methylene-γ-butyrolactone **4**

**Figure 1:**
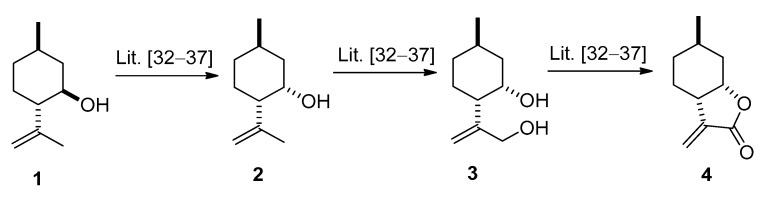
Synthesis of (−)-isopulegol-based *α*-methylene-*γ*-butyrolactone **4**.

The key intermediate (−)-α-methylene-γ-butyrolactone **4** was prepared from commercially available (−)-isopulegol **1** by oxidizing its hydroxy group, followed by stereoselective reduction of the resulting carbonyl group providing (+)-neoisopulegol **2**. Regioselective allylic hydroxylation of **2** gave diol **3**, which was transformed to **4** by oxidation and ring closure of the obtained γ-hydroxy-substituted α,β-unsaturated carboxylic acid applying literature methods [32,33,34,35,36,37] (Figure 1).

### 2.2. Synthesis of Isopulegol-based Aminodiols

**Scheme 1:**
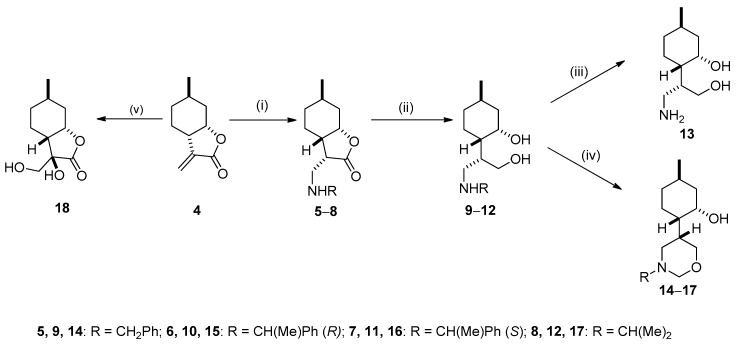
(i) RNH_2_ (1 equivalent), dry EtOH, 25 °C, 20 h, 60–70%; (ii) LiAlH_4_ (2 equivalent), dry Et_2_O, 25 °C, 4 h, 74–99%, (iii) 5% Pd/C, H_2_ (1 atm), 25 °C, 24 h, 50%, (iv) 35% HCHO, Et_2_O, 25 °C, 1 h, 64–83%; (v) 2% OsO_4_/*t*-BuOH, 50% NMO/H_2_O, acetone, 25 °C, 24 h, 50%.

Nucleophilic addition of primary amines to α-methylene-γ-butyrolactone **4** has proved to be an efficient method for the preparation of a highly diversified library of β-aminolactones **5**–**8** [38,39,40]. Treatment of β-aminolactones with LiAlH_4_ resulted in secondary aminodiols **9**–**12** [16]. Secondary aminodiols **9**–**11** were transformed into primary diol **13** with debenzylation through hydrogenolysis over Pd/C. In order to study the regioselectivity of ring closure of the aminodiol function, we attempted to incorporate the hydroxy groups of aminodiols into products with 1,3-oxazine or 1,3-oxazepine ring [16,22,41]. When aminodiols **9**–**12** were reacted with HCHO under mild conditions, 1,3-oxazine **14**–**17** were obtained in highly regioselective ring closure (Scheme 1).

**Figure 2:**
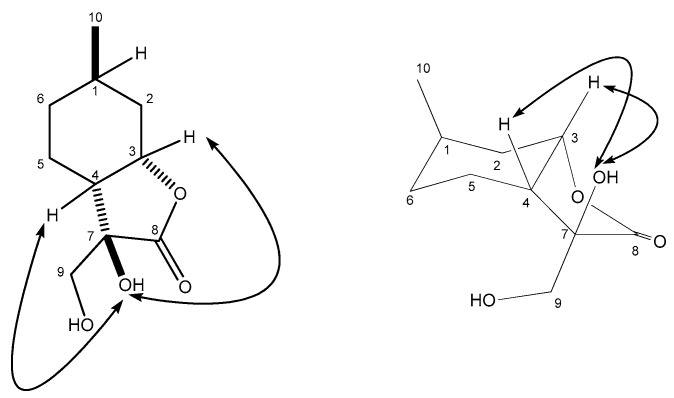
Determination of the structure of diol **18** by NOESY.

Dihydroxylation of **4** with OsO_4_ and NMO (4-Methylmorpholine *N*-oxide) furnished **18** in an acceptable yield [16,22] (Scheme 1). The relative configuration of compound **18** was determined by means of NOESY (Nuclear Overhauser Effect SpecroscopY) experiments: clear NOE (Nuclear Overhauser Effect) signals were observed between the OH-7 and H-3 as well as OH-7 and H-4 protons (Figure 2).

**Scheme 2:**
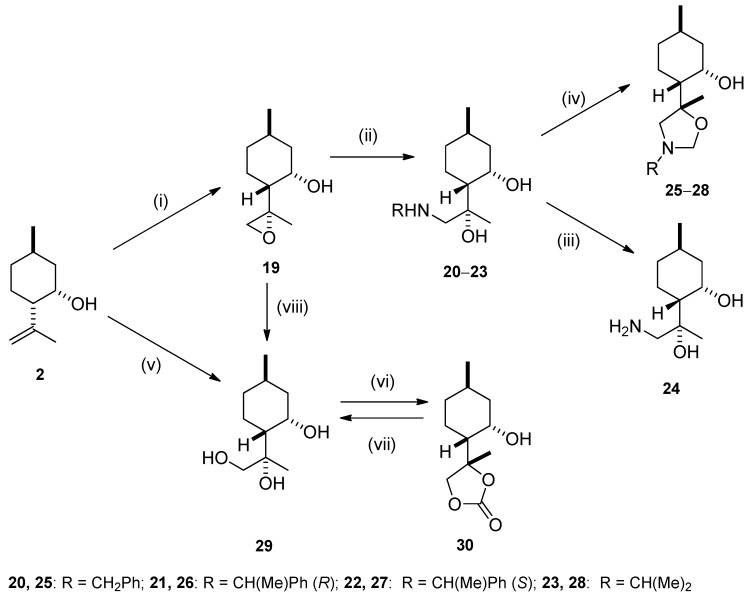
(**i**) VO(acac)_2_, 70% *t*-BuOOH (2 equivalent), dry toluene, 25 °C, 12 h, 76%; (**ii**) RNH_2_ (2 equivalent), LiClO_4_ (1 equivalent), MeCN, 70–80 °C, 6 h, 40–88%; (**iii**) 5% Pd/C, H_2_ (1 atm), MeOH, 25 °C, 24 h, 72%; (**iv**) 35% HCHO, Et_2_O, 25 °C, 1 h, 60–70%; (**v**) 2% OsO_4_/*t*-BuOH, 50% NMO/H_2_O, acetone, 25 °C, 24 h, 61%; (**vi**) triphosgene (0.5 equivalent), dry pyridine (4 equivalent), dry CH_2_Cl_2_, 25 °C, 2 h, 60%; (**vii**) LiAlH_4_ (2 equivalent), dry ether, 25 °C, 4 h, 80%, (**viii**) 3 M NaOH, DMSO, 80 °C, 2 h, 60%.

Epoxidation of **2** with *t*-BuOOH in the presence of vanadyl acetylacetonate (VO(acac)_2_) as catalyst furnished epoxide **19** in a stereospecific reaction [35,42,43,44]. Since our earlier results clearly demonstrated that substituents at nitrogen of aminodiols exerted definite influence on the efficiency of their catalytic activity, aminodiol library **20**–**23** was prepared by aminolysis of **19** with different primary amines and LiClO_4_ as catalyst [17,41,45,46], whereas exposure of **19** to NaOH furnished **29** with retention of stereochemistry [47]. Debenzylation via hydrogenolysis of compounds **20**–**22** over Pd/C in MeOH resulted in primary aminodiol **24** in moderate yield. When aminodiols **20**–**23** were treated with HCHO at room temperature, oxazolidines **25**–**28** were obtained in highly regioselective ring closures, similarly to the regioisomeric oxazine analogues (Scheme 2).

**Figure 3:**
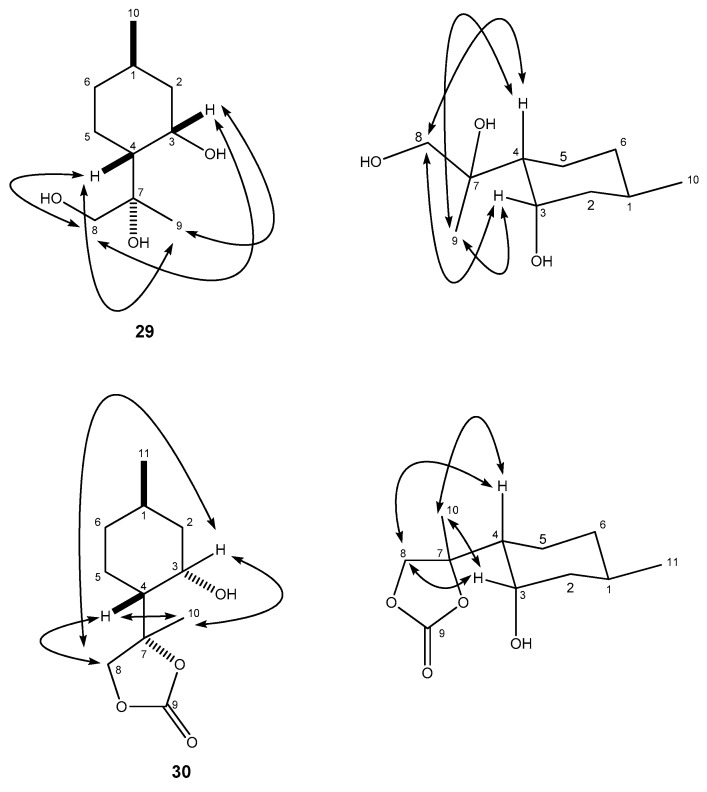
Determination of the structure of diol **29** and carbonate **30** by NOESY.

The *syn*-selective dihydroxylation of compound **2** with OsO_4_ in the presence of a stoichiometric amount of the co-oxidant NMO furnished product **29** as a single diastereomer in moderate yield (Scheme 2). The relative configuration of compound **29** was determined by means of NOESY experiments: clear NOE signals were observed between the H-9 and H-3, H-4 as well as H-8 and H-3, H-4 protons (Figure 3).

The structure of compound **29** was confirmed by its five-membered cyclic carbonate **30** synthesized from **29** by the reaction with triphosgene (Scheme 2) [48]. It is well known that this carbonation reaction maintains the stereochemical configuration of **29** [49,50]. The stereochemical structure of carbonate **30** was identified by NOESY analyses: characteristic NOE signals were observed between the protons H-8 and H-3, H-4 together with the protons H-10 and H-3, H-4 (Figure 3).

Reduction of **30** with LAH (Lithium aluminum hydride) proceeded smoothly giving **29** in an excellent yield (Scheme 2). It has been reported that reduction of the cyclic carbonate moiety of **30** with LAH gave the corresponding diol with the same stereochemical configuration at the carbon atoms as of the original **29** moiety [51,52,53].

### 2.3. Synthesis of Isopulegol-based Aminotriols

**Figure 4:**
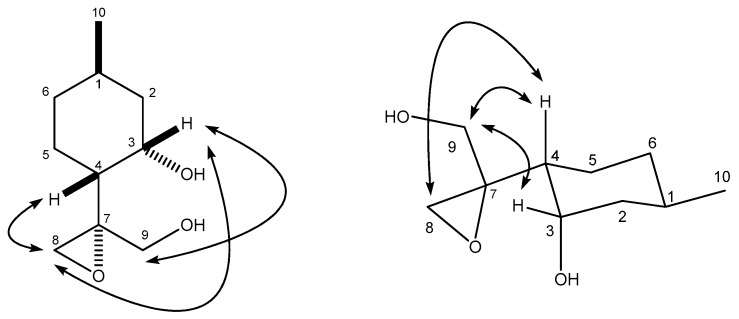
Determination of the structure of epoxide **31** by NOESY.

**Scheme 3:**
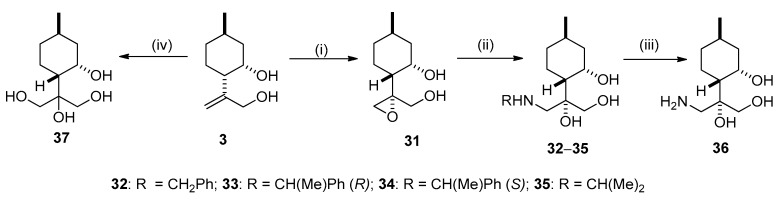
(**i**) VO(acac)_2_, 70% *t*-BuOOH (2 equivalent), dry toluene, 25 °C, 12 h, 80%; (**ii**) RNH_2_ (2 equivalent), LiClO_4_ (1 equivalent), MeCN, 70–80 °C, 6 h, 60–80%; (**iii**) 5% Pd/C, H_2_ (1 atm), MeOH, 25 °C, 24 h, 72%; (**iv**) 2% OsO_4_/*t*-BuOH, 50% NMO/H_2_O, acetone, 25 °C, 24 h, 40%.

Stereospecific epoxidation of allylic diol **3** with *t*-BuOOH and VO(acac)_2_ was successfully applied to prepare epoxy diol **31** [35,43,44] (Scheme 3). The relative configuration of epoxide **31** was determined by means of NOESY experiments. Significant NOE signals were shown between the H-8 and H-3, H-4 as well as the H-3 and H-9 protons (Figure 4).

The oxirane ring of **31** was opened with primary amines and LiClO_4_ as catalyst to give aminotriol library **32**–**35** [45,46]. Primary aminotriol **36** was obtained by debenzylation of the corresponding aminotriols **32**–**34** under standard condition by hydrogenation in the presence of a Pd/C catalyst. The synthesis of tetraol **37** was effectively performed by selective dihydroxylation of compound **3** with the OsO_4_/NMO system [16,22] (Scheme 3).

### 2.4. Application of Aminodiol Derivatives and Aminotriols as Chiral Ligands for Catalytic Addition of Diethylzinc to Benzaldehyde

**Scheme 4:**
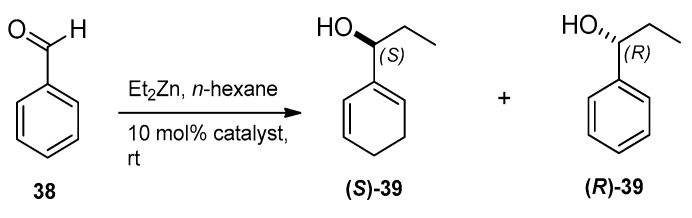
Model reaction for enantioselective catalysis.

Aminodiol derivatives **9**–**17** and **20**–**28** together with aminotriols **32**–**36** were applied as chiral catalysts in the enantioselective addition of diethylzinc to benzaldehyde **38** to form (*S*)- and (*R*)-1-phenyl-1-propanol **39** (Scheme 4).

The enantiomeric purity of 1-phenyl-1-propanols (*S*)-**39** and (*R*)-**39** was determined by GC on a CHIRASIL-DEX CB column using literature methods [14,54]. Low to moderate enantioselectives were observed. The results obtained (see Table 1) clearly show that all aminodiol derivatives favoured the formation of the *(R)-*enantiomer, whereas aminotriols led to the corresponding (*S*)-enantiomer. Aminodiol **10** afforded the best *ee* value (*ee* = 60%) with an (*R*)-selectivity, while aminotriol **34** showed the best *ee* value (*ee* = 28%) with an (*S*)-selectivity. Other compounds were also examined but their selectivities were less than 10% when applied as chiral ligands.

### 2.5. Antimicrobial Effects

Since several aminodiols [5], as well as polyols [29,30,31], exerted antimicrobial activities on various bacterial and fungal strains, antimicrobial activities of the prepared aminodiol analogues and polyols were also tested against two yeasts as well as two Gram-positive and two Gram-negative bacteria (Table 2, only the outstanding results are shown). Compounds **10** and **20** inhibited over 20% against the applied Gram-negative bacteria, while other derivatives showed weak activities. Furthermore, **11** showed an inhibition activity over 40% for *P. aeruginosa*, while it had no effect against *E. coli*. All compounds presented low to moderate inhibitions against the Gram-positive bacteria in the range of 5%–55% and 9%–35% for *B. subtilis* and *S. aureus*, respectively. In the case of *B. subtilis*, **24** showed much more potential antimicrobial activity, while for *S. aureus*, **18** and **29** proved to be the most effective agents. In the yeast assays, only **24** exhibited moderate inhibition against *C. albicans*, whereas significant effects were observed against *C. krusei* almost in all cases reaching up to 50% with analogue **16**.

Comparing the antimicrobial activities of the two families of aminodiols, our results suggest that both 3-aminomethyl-1,4-diols **9**–**13** and 4-amino-1,3-diols **20**–**24** have moderate antifungal activity against *C. krusei* and Gram-negative and Gram-positive bacteria. An interesting difference was observed between 1,3-oxazines and oxazolidines. Namely, oxazines **14**–**17** possess moderate antifungal and antibacterial activity, whereas oxazolidines **25**–**28** proved ineffective.

Polyols **29** and **37** have antibacterial effect only against the examined Gram-positive bacteria. From aminotriol library only primary aminotriol **36** has substantial antifungal and antibacterial effect. In contrast, *N*-substitution led to the loss of antimicrobial activity.

## 3. Discussion

Starting from the commercially available (−)-isopugeol, a new family of isopulegol-based chiral aminodiol and aminotriol libraries were prepared through chiral (+)-neoisopulegol as key intermediate via stereoselective transformations. Moreover, isopulegol-based chiral di-, tri- and tetraols, promising chiral substrates for the synthesis of chiral crown ethers were synthesized. 

The resulting aminodiols exert moderate antimicrobial action on a panel of bacterial and fungal strains. The in vitro pharmacological studies have clearly shown that these primary aminodiols have significant microbiological effects. In addition, aminodiol and aminotriol derivatives were applied as chiral catalysts in the enantioselective addition of diethylzinc to benzaldehyde with moderate but significantly opposite stereoselectivity.

## 4. Materials and Methods

### 4.1. General Methods

Commercially available compounds were used as obtained from suppliers (Molar Chemicals Ltd, Halásztelek, Hungary; Merck Ltd., Budapest, Hungary and VWR International Ltd., Debrecen, Hungary), while applied solvents were dried according to standard procedures. Optical rotations were measured in MeOH at 20 °C with a Perkin-Elmer 341 polarimeter (PerkinElmer Inc., Shelton, CT, USA). Chromatographic separations and monitoring of reactions were carried out on Merck Kieselgel 60 (Merck Ltd., Budapest, Hungary). Elemental analyses for all prepared compounds were performed on a Perkin-Elmer 2400 Elemental Analyzer (PerkinElmer Inc., Waltham, MA, USA). GC measurements for direct separation of commercially available enantiomers of isopulegol to determine the enantiomeric purity of starting material **1** and separation of *O*-acetyl derivatives of enantiomers were performed on a Chirasil-DEX CB column (2500 × 0.25 mm I.D.) on a Perkin-Elmer Autosystem XL GC consisting of a Flame Ionization Detector (Perkin-Elmer Corporation, Norwalk, CT, USA) and a Turbochrom Workstation data system (Perkin-Elmer Corp., Norwalk, CT, USA). Melting points were determined on a Kofler apparatus (Nagema, Dresden, Germany) and are uncorrected. ^1^H- and ^13^C-NMR were recorded on Brucker Avance DRX 500 spectrometer [500 MHz (^1^H) and 125 MHz (^13^C), δ = 0 (TMS)]. Chemical shifts are expressed in ppm (δ) relative to TMS as the internal reference. *J* values are given by Hz. Supplementary material, all 1H/13C NMR spectra, in addition to NOESY, also 2D-HMBC and 2D-HMQC spectra are involved in Supporting Information file.

### 4.2. Starting Materials

(*−*)-Isopulegol **1** is available commercially from Merck Co with *ee* = 95%. (+)-Neoisopulegol **2**, diol **3** and (*−*)-*α*-methylene-*γ*-butyrolactone **4** were prepared according to literature procedures, and all spectroscopic data were similar to those described therein [35]. The nucleophilic addition of *α*-methylene-*γ*-butyrolactone to amines were carried out according to our literature procedures with all spectroscopic data of compounds **5**–**7** being consistent with literature values [38].

#### (3*S*,3a*S*,6*R*,7a*S*)-3-((Isopropylamino)methyl)-6-methylhexahydrobenzofuran-2(3*H*)-one (**8**)

Yield: 70%, white crystals, m.p.: 192–195 °C. ${\left[\alpha \right]}_{D}^{20}$ = +42.0 (c 0.22, MeOH). ^1^H NMR (500 MHz, CDCl_3_): δ = 0.91 (3H, d, *J* = 6.5 Hz), 0.95–1.01 (2H, m), 1.20–1.26 (1H, m), 1.47 (6H, d, *J* = 6.5 Hz), 1.53–1.54 (1H, m), 1.67–1.70 (1H, m), 1.82–1.92 (1H, m), 2.22 (1H, d, *J* = 15.0 Hz), 2.72–2.76 (1H, m), 3.12–3.16 (1H, m), 3.22–3.26 (1H, m), 3.41–3.47 (1H, m), 3.62 (1H, q, *J* = 6.4 Hz), 4.60 (1H, d, *J* = 2.0 Hz), 8.69 (1H, brs), 10.04 (1H, brs). ^13^C NMR (125 MHz, CDCl_3_): δ = 18.9, 19.4, 21.9, 23.2, 26.1, 31.6, 35.7, 37.5, 40.5, 45.0, 51.5, 79.5, 177.1. Anal. Calcd for C_13_H_23_NO_2_: C, 69.29; H, 10.29; N, 6.22. Found: C, 69.30; H, 10.27; N, 6.25.

### 4.3. General Procedure for Reduction with LiAlH_4_

To a stirred suspension of LiAlH_4_ (8 mmol) in dry ether (16 mL), a solution of compounds **5**–**8** (4 mmol) in dry ether (20 mL) was added at 0 °C. The reaction mixture was stirred for 4 h at room temperature, while the reaction progress was monitored by TLC. A mixture of H_2_O (1.0 mL) and THF (10 mL) was then added dropwise with cooling. The inorganic material was filtered off and washed with Et_2_O (for compounds **9**–**12**) or EtOAc (for compound **29**). The filtrate was dried (Na_2_SO_4_) and evaporated to dryness. The crude product was purified by column chromatography on silica gel using CHCl_3_:MeOH = 9:1 (for compounds **9**–**12**) or *n*-hexane:EtOAc = 1:4 (for compound **29**).

#### 4.3.1. (1*S*,2*S*,5*R*)-2-((*S*)-1-(Benzylamino)-3-hydroxypropan-2-yl)-5-methylcyclohexanol (**9**)

Yield: 98%, white crystals, m.p.: 101–103 °C. ${\left[\alpha \right]}_{D}^{20}$ = +8.0 (c 0.29, MeOH). ^1^H NMR (500 MHz, CDCl_3_): δ = 0.86 (3H, d, *J* = 6.4 Hz), 0.88–0.94 (1H, m), 1.05–1.10 (1H, m), 1.32–1.36 (1H, m), 1.40–1.43 (1H, m), 1.53–1.58 (1H, m), 1.66–1.73 (2H, m), 1.78–1.87 (2H, m), 2.78 (1H, dd, *J* = 3.8, 12.1 Hz), 2.87 (1H, dd, *J* = 5.7, 12.2 Hz), 3.60 (1H, q, *J* = 7.7 Hz), 3.72–3.75 (1H, m), 3.77 (2H, s), 3.94 (1H, s), 7.25–7.34 (5H, m). ^13^C NMR (125 MHz, CDCl_3_): δ = 22.5, 25.2, 26.2, 35.4, 42.1, 42.3, 43.7, 49.1, 54.2, 64.6, 66.5, 127.5, 128.5, 128.7, 138.8. Anal. Calcd for C_17_H_27_NO_2_: C, 73.61; H, 9.81; N, 5.05. Found: C, 73.65; H, 9.80; N, 5.10.

#### 4.3.2. (1*S*,2*S*,5*R*)-2-((*S*)-1-Hydroxy-3-(((*R*)-1-phenylethyl)amino)propan-2-yl)-5-methylcyclohexanol (**10**)

Yield: 74%, colorless oil. ${\left[\alpha \right]}_{D}^{20}$ = +33.0 (c 0.26, MeOH). ^1^H NMR (500 MHz, CDCl_3_): δ = 0.85 (3H, d, *J* = 6.5 Hz), 0.82–0.95 (1H, m), 1.05–1.10 (1H, m), 1.25–1.29 (1H, m), 1.39 (3H, d, *J* = 6.6 Hz), 1.42–1.48 (1H, m), 1.57–1.59 (1H, m), 1.68 (1H, d, *J* = 12.9 Hz), 1.78–1.87 (2H, m), 2.57 (1H, dd, *J* = 4.9, 12.1 Hz), 2.76 (1H, dd, *J* = 5.6, 12.1 Hz), 3.16 (2H, brs), 3.57 (1H, q, *J* = 7.4 Hz), 3.71–3.75 (2H, m), 3.96 (1H, s), 7.24–7.35 (5H, m). ^13^C NMR (125 MHz, CDCl_3_): δ = 22.5, 23.8, 25.1, 26.1, 35.3, 41.9, 42.4, 43.8, 48.3, 59.0, 65.3, 66.8, 126.8, 127.4, 128.7, 144.3. Anal. Calcd for C_18_H_29_NO_2_: C, 74.18; H, 10.03; N, 4.81. Found: C, 74.20; H, 10.05; N, 4.85.

#### 4.3.3. (1*S*,2*S*,5*R*)-2-((*S*)-1-Hydroxy-3-(((*S*)-1-phenylethyl)amino)propan-2-yl)-5-methylcyclohexanol (**11**)

Yield: 74%, white crystals, m.p.: 144–146 °C. ${\left[\alpha \right]}_{D}^{20}$ = −16.0 (c 0.25, MeOH). ^1^H NMR (500 MHz, CDCl_3_): δ = 0.86 (3H, d, *J* = 6.3 Hz), 0.88–0.94 (1H, m), 1.06 (1H, t, *J* = 12.5 Hz), 1.30–1.34 (1H, m), 1.39 (3H, d, *J* = 6.6 Hz), 1.38–1.42 (1H, m), 1.56–1.64 (2H, m), 1.71–1.74 (1H, m), 1.75–1.90 (2H, m), 2.60 (1H, dd, *J* = 6.0, 12.2 Hz), 2.66 (1H, dd, *J* = 3.3 Hz, 12.2 Hz), 3.11 (2H, brs), 3.58 (1H, q, *J* = 3.0 Hz), 3.69–3.72 (2H, m), 3.86 (1H, s), 7.26–7.35 (5H, m). ^13^C NMR (125 MHz, CDCl_3_): δ = 22.5, 23.7, 25.4, 26.2, 35.4, 42.0, 42.2, 44.1, 47.0, 58.9, 64.5, 66.2, 126.8, 127.5, 128.7, 144.2. Anal. Calcd for C_18_H_29_NO_2_: C, 74.18; H, 10.03; N, 4.81. Found: C, 74.15; H, 10.00; N, 4.79.

#### 4.3.4. (1*S*,2*S*,5*R*)-2-((*S*)-1-Hydroxy-3-(isopropylamino)propan-2-yl)-5-methylcyclohexanol (**12**)

Yield: 99%, white crystals, m.p.: 155–160 °C. ${\left[\alpha \right]}_{D}^{20}$ = +18.0 (c 0.275, MeOH). ^1^H NMR (500 MHz, CDCl_3_): δ = 0.85 (3H, d, *J* = 6.4 Hz), 0.85–0.95 (1H, m), 1.09 (1H, t, *J* = 12.8 Hz), 1.43 (6H, dd, *J* = 5.9, 11.6 Hz), 1.41–1.54 (3H, m), 1.72–1.79 (2H, m), 1.91 (1H, d, *J* = 13.3 Hz), 2.17 (1H, s), 3.25–3.30 (2H, m), 3.71 (1H, t, *J* = 9.9 Hz), 3.85–3.87 (1H, m), 4.06 (1H, s), 8.96 (1H, brs), 9.04 (1H, brs). ^13^C NMR (125 MHz, CDCl_3_): δ = 19.1, 19.5, 22.3, 24.8, 25.9, 35.0, 41.0, 42.1, 42.2, 43.6, 51.3, 62.1, 65.6. Anal. Calcd for C_13_H_27_NO_2_: C, 68.08; H, 11.87; N, 6.11. Found: C, 68.10; H, 11.90; N, 6.15.

### 4.4. General Procedure of Epoxidation

To a stirred pale red solution of **9** or **31** (5.87 mmol) and vanadyl acetylacetonate (10 mg) in dry toluene (30 mL) *t*-BuOOH (70% solution in H_2_O, 11.8 mmol) dried briefly (Na_2_CO_3_) was added dropwise at 25 °C. The red colour of the solution darkened during the addition then faded to brownish yellow. Stirring was continued (12 h), whereupon KOH (9.8 mmol) in brine (25 mL) was added. The mixture was extracted with toluene (3 × 100 mL) and the organic layer was washed with brine before drying (Na_2_SO_4_) and evaporation. Compounds **10** and **32** were isolated after flash column chromatography (*n*-hexane:EtOAc = 4:1 for **10** and *n*-hexane:EtOAc = 1:1 for **32**).

#### 4.4.1. (1*S*,2*R*,5*R*)-5-Methyl-2-((*S*)-2-methyloxiran-2-yl)cyclohexanol (**19**)

Yield: 76%, white crystals, m.p.: 38–41 °C. ${\left[\alpha \right]}_{D}^{20}$ = +69.0 (c 0.25, MeOH). All spectroscopic data of compound **19** was consistent with literature data [35,42].

#### 4.4.2. (1*S*,2*R*,5*R*)-2-((*R*)-2-(Hydroxymethyl)oxiran-2-yl)-5-methylcyclohexanol (**31**)

Yield: 80%, colorless oil. ${\left[\alpha \right]}_{D}^{20}$ = +36.0 (c 0.275, MeOH). ^1^H NMR (500 MHz, CDCl_3_): δ= 0.77 (3H, d, *J* = 6.3 Hz), 0.81–0.86 (1H, m), 1.00 (1H, t, *J* = 12.9 Hz), 1.26 (1H, d, *J* = 12.4 Hz), 1.43–1.46 (1H, m), 1.60–1.72 (4H, m), 2.67 (2H, dd, *J* = 4.5, 19.8 Hz), 3.18 (1H, d, *J* = 11.7 Hz), 3.81 (1H, d, *J* = 11.7 Hz), 4.04 (1H, s), 4.14 (1H, s), 4.62 (1H, brs). ^13^C NMR (125 MHz, CDCl_3_): δ = 22.0, 22.1, 25.6, 34.4, 41.7, 45.5, 50.4, 61.6, 63.2, 68.0. Anal. Calcd for C_10_H_18_O_3_: C, 64.49; H, 9.74. Found: C, 64.45; H, 9.70.

### 4.5. General Procedure for Ring-Opening of Epoxide with Primary Amines

To a solution of epoxide **10** or **32** (2.94 mmol) in MeCN (30 mL) was added a solution of the appropriate amine (5.88 mmol) in MeCN (10 mL) and LiClO_4_ (2.94 mmol). The mixture was kept at reflux temperature for 4 hours. When the reaction completed (indicated by TLC), the mixture was evaporated to dryness, the residue was dissolved in water (15 mL) and extracted with CH_2_Cl_2_ (3 × 50 mL). The combined organic phase was dried (Na_2_SO_4_), filtered and concentrated. The crude product was purified by column chromatography on silica gel with an appropriate solvent mixture (CHCl_3_:MeOH = 19:1). Purification by recrystallization from a mixture of *n*-hexane:Et_2_O resulted in compounds **20**–**23** or **32**–**35**, respectively.

#### 4.5.1. (1*S*,2*R*,5*R*)-2-((*S*)-1-(Benzylamino)-2-hydroxypropan-2-yl)-5-methylcyclohexanol (**20**)

Yield: 40%, white crystals, m.p.: 146–148 °C. ${\left[\alpha \right]}_{D}^{20}$ = +15.0 (c 0.27, MeOH). ^1^H NMR (500 MHz, CDCl_3_): δ = 0.83 (3H, d, *J* = 6.4 Hz), 0.83–0.93 (1H, m), 0.95–1.03 (1H, m), 1.07–1.17 (1H, m), 1.31 (3H, s), 1.50 (1H, d, *J* = 12.4 Hz), 1.64 (1H, d, *J* = 12.5 Hz), 1.70–1.80 (2H, m), 1.89 (1H, brs), 2.78 (1H, t, *J* = 11.4 Hz), 2.95 (1H, t, *J* = 10.6 Hz), 3.29 (1H, brs), 3.74 (1H, s), 3.92–3.96 (1H, m), 4.49–4.53 (2H, m), 6.89 (1H, brs), 7.42 (5H, s), 10.2 (1H, s). ^13^C NMR (125 MHz, CDCl_3_): δ = 21.8, 21.9, 25.6, 29.6, 35.0, 42.2, 50.2, 51.2, 51.3, 65.2, 71.6, 129.5, 129.8, 130.2, 130.4. Anal. Calcd for C_17_H_27_NO_2_: C, 73.61; H, 9.81; N, 5.05. Found: C, 73.65; H, 9.80; N, 5.10.

#### 4.5.2. (1*S*,2*R*,5*R*)-2-((*S*)-2-Hydroxy-1-(((*R*)-1-phenylethyl)amino)propan-2-yl)-5-methylcyclohexanol (**21**)

Yield: 43%, white crystals, m.p.: 118–119 °C. ${\left[\alpha \right]}_{D}^{20}$ = +5.0 (c 0.295, MeOH). ^1^H NMR (500 MHz, CDCl_3_): δ = 0.85 (3H, d, *J* = 6.3 Hz), 0.84–0.89 (2H, m), 1.05–1.15 (2H, m), 1.25–1.33 (1H, m), 1.28 (3H, s), 1.52 (1H, d, *J* = 9.3 Hz), 1.63–1.65 (1H, m), 1.70–1.88 (3H, m), 1.79 (3H, s), 2.56 (1H, s), 2.97 (1H, s), 4.06 (1H, brs), 4.26 (1H, s), 4.59 (1H, s), 6.99 (1H, s), 7.30–7.50 (5H, m), 10.3 (1H, s). ^13^C NMR (125 MHz, CDCl_3_): δ = 20.7, 21.8, 22.0, 25.8, 29.8, 35.1, 42.3, 50.3, 51.1, 59.4, 65.4, 71.6, 127.9, 129.6, 129.9, 135.8. Anal. Calcd for C_18_H_29_NO_2_: C, 74.18; H, 10.03; N, 4.81. Found: C, 74.20; H, 10.05; N, 4.80.

#### 4.5.3. (1*S*,2*R*,5*R*)-2-((*S*)-2-hydroxy-1-(((*S*)-1-phenylethyl)amino)propan-2-yl)-5-methylcyclohexanol (**22**)

Yield: 43%, white crystals, m.p.: 160–161 °C. ${\left[\alpha \right]}_{D}^{20}$ = −9.0 (c 0.255, MeOH). ^1^H NMR (500 MHz, CDCl_3_): δ = 0.87 (3H, d, *J* = 6.3 Hz), 0.87–0.95 (1H, m), 1.04 (1H, t, *J* = 14.5 Hz), 1.19 (3H, s), 1.34 (2H, d, *J* = 10.5 Hz), 1.42 (3H, d, *J* = 6.87 Hz), 1.59–1.68 (1H, m), 1.75–1.77 (1H, m), 1.90–1.97 (1H, m), 2.36 (1H, d, *J* = 12.1 Hz), 2.54 (1H, d, *J* = 12.1 Hz), 3.73 (1H, q, *J* = 6.6 Hz), 4.29 (1H, s), 7.26–7.36 (5H, m). ^13^C NMR (125 MHz, CDCl_3_): δ = 22.1, 22.4, 23.1, 26.0, 29.0, 35.6, 42.3, 52.3, 52.7, 58.5, 64.6, 74.1, 126.6, 127.5, 128.8, 143.9. Anal. Calcd for C_18_H_29_NO_2_: C, 74.18; H, 10.03; N, 4.81. Found: C, 74.15; H, 10.02; N, 4.85.

#### 4.5.4. (1*S*,2*R*,5*R*)-2-((*S*)-2-Hydroxy-1-(isopropylamino)propan-2-yl)-5-methylcyclohexanol (**23**)

Yield: 88%, yellow oil. ${\left[\alpha \right]}_{D}^{20}$ = +20.0 (c 0.245, MeOH). ^1^H NMR (500 MHz, CDCl_3_): δ = 0.87 (3H, d, *J* = 6.1 Hz), 0.95–1.01 (1H, m), 1.16 (1H, t, *J* = 13.3 Hz), 1.35 (3H, d, *J* = 6.5 Hz), 1.39 (3H, s), 1.389 (3H, d, *J* = 6.8 Hz), 1.30–1.53 (3H, m), 1.59 (1H, d, *J* = 12.0 Hz), 1.78–1.87 (3H, m), 2.91 (1H, t, *J* = 11.1 Hz), 2.97–3.03 (1H, m), 3.39 (1H, quin, *J* = 6.0 Hz), 3.85 (1H, brs), 4.54 (1H, s), 6.37 (1H, s), 9.77 (1H, s). ^13^C NMR (125 MHz, CDCl_3_): δ =19.1, 19.6, 22.0, 22.2, 25.8, 29.6, 35.1, 42.1, 49.0, 51.0., 51.2, 65.3, 71.6. Anal. Calcd for C_13_H_27_NO_2_: C, 68.08; H, 11.87; N, 6.11. Found: C, 68.10; H, 11.85; N, 6.13.

#### 4.5.5. (*S*)-3-(Benzylamino)-2-((1*R*,2*S*,4*R*)-2-hydroxy-4-methylcyclohexyl)propane-1,2-diol (**32**)

Yield: 60%, colorless oil. ${\left[\alpha \right]}_{D}^{20}$ = +20.0 (c 0.26, MeOH). ^1^H NMR (500 MHz, DMSO-*d*_6_): δ = 0.81 (3H. d, *J* = 6.3 Hz), 0.80–0.87 (1H, m), 1.01 (1H, t, *J* = 13.2 Hz), 1.30–1.38 (2H, m), 1.50 (1H, t, *J* = 7.8 Hz), 1.63 (1H, d, *J* = 12.5 Hz), 1.67–1.77 (2H, m), 2.85 (1H, dd, *J* = 12.8, 21.0 Hz), 3.32 (1H, d, *J* = 11.4 Hz), 3.41 (1H, d, *J* = 11.3 Hz), 4.11 (1H, q, *J* = 12.8 Hz), 4.98 (1H, s), 7.41–7.47 (5H, m). ^13^C NMR (125 MHz, DMSO-*d*_6_): δ = 21.0, 22.1, 25.3, 34.6, 41.8, 45.6, 48.4, 50.8, 64.3, 66.0, 73.7, 128.7, 128.8, 129.9, 132.4. Anal. Calcd for C_13_H_27_NO_3_: C, 69.59; H, 9.28; N, 4.77. Found: C, 69.60; H, 9.25; N, 4.82.

#### 4.5.6. (*S*)-2-((1*R*,2*S*,4*R*)-2-Hydroxy-4-methylcyclohexyl)-3-(((*R*)-1-phenylethyl)amino)propane-1,2-diol (**33**)

Yield: 60%, colorless oil. ${\left[\alpha \right]}_{D}^{20}$ = +10.0 (c 0.24, MeOH). ^1^H NMR (500 MHz, DMSO-*d*_6_): δ = 0.81 (3H, d, *J* = 6.3 Hz), 0.80–0.85 (1H, m), 1.01 (1H, t, *J* = 12.9 Hz), 1.10–1.15 (1H, m), 1.19–1.23 (1H, m), 1.49 (1H. d, *J* = 12.5 Hz), 1.55 (3H, d, *J* = 6.7 Hz), 1.54–1.58 (1H, m), 1.67–1.75 (2H, m), 2.54 (1H, d, *J* = 13.0 Hz), 2.83 (1H, d, *J* = 12.7 Hz), 3.27 (1H, d, *J* = 11.3 Hz), 4.12 (1H, s), 4.32 (1H, d, *J* = 6.3 Hz), 5.03 (1H, s), 5.11 (1H, brs), 6.05 (1H, brs), 7.39–7.50 (5H, m), 8.72 (1H, brs), 9.40 (1H, brs). ^13^C NMR (125 MHz, DMSO-*d*_6_): δ = 19.0, 21.2, 22.1, 25.3, 34.6, 41.7, 45.6, 46.7, 57.5, 64.1, 66.2, 73.9, 127.9, 128.9, 129.0, 137.1. Anal. Calcd for C_18_H_27_NO_3_: C, 70.32; H, 9.51; N, 4.56. Found: C, 70.35; H, 9.55; N, 4.52.

#### 4.5.7. (*S*)-2-((1*R*,2*S*,4*R*)-2-Hydroxy-4-methylcyclohexyl)-3-(((*S*)-1-phenylethyl)amino)propane-1,2-diol (**34**)

Yield: 80%, colorless oil. ${\left[\alpha \right]}_{D}^{20}$ = +3.0 (c 0.235, MeOH). ^1^H NMR (500 MHz, DMSO-*d*_6_): δ = 0.83 (3H, d, *J* = 6.2 Hz), 0.82–0.92 (1H, m), 1.04 (1H, t, *J* = 12.9 Hz), 1.31–1.44 (2H, m), 1.49–1.55 (1H, m), 1.54 (3H, d, *J* = 6.8 Hz), 1.65 (1H, d, *J* = 12.5 Hz), 1.70–1.79 (2H, m), 2.56 (1H, d, *J* = 12.7 Hz), 2.73 (1H, d, *J* = 12.7 Hz), 3.27 (1H, d, *J* = 11.3 Hz), 3.33 (1H, s), 4.19 (1H, s), 4.33 (1H, q, *J* = 6.6 Hz), 5.02 (1H, s), 7.39–7.53 (5H, m). ^13^C NMR (125 MHz, DMSO-*d*_6_): δ = 19.4, 21.2, 22.1, 25.4, 34.6, 41.8, 45.6, 47.1, 57.5, 64.3, 66.0, 73.7, 127.7, 128.9, 137.2. Anal. Calcd for C_18_H_27_NO_3_: C, 70.32; H, 9.51; N, 4.56. Found: C, 70.30; H, 9.48; N, 4.60.

#### 4.5.8. (*S*)-2-((1*R*,2*S*,4*R*)-2-Hydroxy-4-methylcyclohexyl)-3-(isopropylamino)propane-1,2-diol (**35**)

Yield: 75%, colorless oil. ${\left[\alpha \right]}_{D}^{20}$ = +15.0 (c 0.29, MeOH). ^1^H NMR (500 MHz, DMSO-*d*_6_): δ = 0.83 (3H, d, *J* = 6.2 Hz), 0.83–0.90 (1H, m), 1.05 (1H, t, *J* = 12.8 Hz), 1.20 (6H, d, *J* = 6.3 Hz), 1.40–1.47 (2H, m), 1.54–1.57 (1H, m), 1.65–1.80 (3H, m), 2.86 (1H, dd, *J* = 12.8, 25.4 Hz), 3.25 (1H, quin, *J* = 6.4 Hz), 3.43 (1H, d, *J* = 11.3 Hz), 4.19 (1H, s), 5.03 (1H, s). ^13^C NMR (125 MHz, DMSO-*d*_6_): δ = 18.2, 18.8, 21.2, 22.1, 25.4, 34.7, 42.0, 45.6, 46.0, 50.0, 64.3, 65.7, 73.7. Anal. Calcd for C_13_H_27_NO_3_: C, 63.64; H, 11.09; N, 5.71. Found: C, 63.63; H, 11.05; N, 4.75.

### 4.6. General Procedure for Ring Closure with Formaldehyde

To a solution of aminodiols **9**–**12** or **20**–**23** (1.8 mmol) in 5 mL Et_2_O was added 20 mL of 35% aqueous formaldehyde and the mixture was stirred at room temperature. After 1 h, it was made alkaline with 10% aqueous KOH (20 mL) and extracted with Et_2_O (3 × 50 mL). After drying (Na_2_SO_4_) and solvent evaporation crude products **14**–**17** or **25**–**28** were purified by column chromatography (CHCl_3_:MeOH = 19:1).

#### 4.6.1. (1*S*,2*S*,5*R*)-2-((*S*)-3-Benzyl-1,3-oxazinan-5-yl)-5-methylcyclohexanol (**14**)

Yield: 64%, colorless oil. ${\left[\alpha \right]}_{D}^{20}$ = −4.0 (c, 0.285 MeOH). ^1^H NMR (500 MHz, CDCl_3_): δ = 0.86 (3H, d, *J* = 6.4 Hz), 0.86–0.94 (1H, m), 1.07 (1H, t, *J* = 12.6 Hz), 1.25–1.33 (2H, m), 1.56–1.62 (1H, m), 1.70–1.73 (1H, m), 1.75–1.90 (3H, m), 2.64 (1H, s), 2.85 (1H, s), 3.58–3.67 (3H, m), 3.90 (2H, d, *J* = 7.4 Hz), 4.13 (1H, s), 4.32 (1H, d, *J* = 8.2 Hz), 7.25–7.33 (5H, m). ^13^C NMR (125 MHz, CDCl_3_): δ = 22.5, 24.5, 26.0, 35.3, 42.3, 44.7, 52.3, 57.4, 65.9, 72.1, 85.0, 127.6, 128.6, 129.3, 136.6. Anal. Calcd for C_18_H_27_NO_2_: C, 74.70; H, 9.40; N, 4.84. Found: C, 74.73; H, 9.37; N, 4.85.

#### 4.6.2. (1*S*,2*S*,5*R*)-5-Methyl-2-((*S*)-3-((*R*)-1-phenylethyl)-1,3-oxazinan-5-yl)cyclohexanol (**15**)

Yield: 65%, colorless oil. ${\left[\alpha \right]}_{D}^{20}$ = +11.0 (c 0.28, MeOH). ^1^H NMR (500 MHz, CDCl_3_): δ = 0.84 (3H, d, *J* = 6.3 Hz), 0.84–0.89 (1H, m), 1.03 (1H, t, *J* = 12.8 Hz), 1.15–1.27 (2H, m), 1.41 (3H, d, *J* = 6.6 Hz), 1.42–1.50 (1H, m), 1.65–1.70 (1H, m), 1.70–1.85 (3H, m), 2.43 (1H, s), 2.65 (1H, s), 3.66 (1H, s), 3.71 (1H, s), 3.85 (1H, d, *J* = 8.2 Hz), 4.00 (1H, s), 4.11 (1H, brs), 4.54 (1H, d, *J* = 5.4 Hz), 7.26–7.36 (5H, m). ^13^C NMR (125 MHz, CDCl_3_): δ = 20.2, 22.5, 24.3, 26.0, 35.3, 42.2, 44.5, 49.7, 59.8, 65.8, 71.9, 83.5, 127.6, 127.7, 128.6. Anal. Calcd for C_19_H_29_NO_2_: C, 75.21; H, 9.63; N, 4.62. Found: C, 75.25; H, 9.60; N, 4.65.

#### 4.6.3. (1*S*,2*S*,5*R*)-5-Methyl-2-((*S*)-3-((*S*)-1-phenylethyl)-1,3-oxazinan-5-yl)cyclohexanol (**16**)

Yield: 65%, colorless oil. ${\left[\alpha \right]}_{D}^{20}$ = −22.0 (c 0.27, MeOH). ^1^H NMR (500 MHz, CDCl_3_): δ = 0.88 (3H, d, *J* = 6.4 Hz), 0.91–0.99 (1H, m), 1.11 (1H, td, *J* = 1.9, 12.9 Hz), 1.25–1.30 (1H, m), 1.35–1.45 (1H, m), 1.43 (3H, d, *J* = 6.7 Hz), 1.70–1.80 (3H, m), 1.90 (2H, d, *J* = 12.0 Hz), 2.60 (1H, brs), 3.14 (1H, d, *J* = 8.7 Hz), 3.54 (1H, s), 3.64 (1H, s), 3.72 (1H, d, *J* = 10.2 Hz), 3.79 (1H, d, *J =* 8.6 Hz), 4.22 (1H, s), 4.26 (1H, d, *J* = 8.2 Hz), 7.23–7.33 (5H, m). ^13^C NMR (125 MHz, CDCl_3_): δ = 20.3, 22.5, 24.9, 26.1, 35.5, 42.2, 45.5, 49.5, 60.5, 65.8, 72.1, 83.8, 127.4, 127.5, 128.7, 142.5. Anal. Calcd for C_19_H_29_NO_2_: C, 75.21; H, 9.63; N, 4.62. Found: C, 75.20; H, 9.65; N, 4.65.

#### 4.6.4. (1*S*,2*S*,5*R*)-2-((*S*)-3-Isopropyl-1,3-oxazinan-5-yl)-5-methylcyclohexanol (**17**)

Yield: 83%, colorless oil. ${\left[\alpha \right]}_{D}^{20}$ = −2.0 (c 0.20, MeOH). ^1^H NMR (500 MHz, CDCl_3_): δ = ^13^C NMR (125 MHz, CDCl_3_): δ = 0.85 (3H, d, *J* = 6.4 Hz), 0.85–0.90 (1H, m), 0.90–0.97 (1H, m), 1.06–1.11 (1H, m), 1.09 (3H, d, *J* = 4.7 Hz), 1.10 (3H, d, *J* = 4.8 Hz), 1.19–1.30 (3H, m), 1.40–1.45 (1H, m), 1.72–1.75 (3H, m), 1.80–1.90 (2H, m), 2.55–2.75 (1H, m), 2.83 (1H, quin, *J* = 6.4 Hz), 2.90 (1H, d, *J* = 11.1 Hz), 3.79 (2H, s), 3.98 (1H, s), 4.22 (1H, s), 4.45 (1H, d, *J* =7.7 Hz). ^13^C NMR (125 MHz, CDCl_3_): δ = 18.6, 18.8, 22.5, 25.0, 26.1, 29.8, 35.6, 42.2, 45.8, 47.3, 51.2, 65.7, 72.2, 82.8. Anal. Calcd for C_14_H_27_NO_2_: C, 69.66; H, 11.27; N, 5.80. Found: C, 69.70; H, 9.29; N, 4.78.

#### 4.6.5. (1*S*,2*R*,5*R*)-2-((*S*)-3-Benzyl-5-methyloxazolidin-5-yl)-5-methylcyclohexanol (**25**)

Yield: 60%, colorless oil. ${\left[\alpha \right]}_{D}^{20}$ = +29.0 (c 0.24, MeOH). ^1^H NMR (500 MHz, CDCl_3_): δ = 0.86 (3H, d, *J* = 6.4 Hz), 0.85–0.90 (1H, m), 1.00–1.05 (1H, m), 1.33 (3H, s), 1.33–1.38 (2H, m), 1.52–1.56 (1H, m), 1.73 (1H, d, *J* = 12.8 Hz), 1.82–1.92 (2H, m), 2.06 (1H, d, *J* = 9.4 Hz), 3.35 (1H, d, *J* = 9.3 Hz), 3.53 (1H, d, *J* = 12.6 Hz), 3.73 (1H, d, *J* = 12.6 Hz), 3.90 (1H, s), 4.26 (1H, s), 4.57 (1H, s), 5.80 (1H, brs), 7.26–7.34 (5H, m). ^13^C NMR (125 MHz, CDCl_3_): δ = 22.4, 23.1, 26.2, 27.2, 35.3, 41.8, 51.1, 56.5, 58.9, 65.4, 84.9, 85.8, 127.8, 128.7, 128.9, 136.9. Anal. Calcd for C_18_H_27_NO_2_: C, 74.70; H, 9.40; N, 4.84. Found: C, 74.68; H, 9.35; N, 4.80.

#### 4.6.6. (1*S*,2*R*,5*R*)-5-Methyl-2-((*S*)-5-methyl-3-((*R*)-1-phenylethyl)oxazolidin-5-yl)cyclohexanol (**26**)

Yield: 65%, colorless oil. ${\left[\alpha \right]}_{D}^{20}$ = +3.0 (c 0.30, MeOH). ^1^H NMR (500 MHz, CDCl_3_): δ = 0.84 (3H, d, *J* = 6.5 Hz), 0.83–0.89 (1H, m), 1.02 (1H, td, *J* = 2.0, 12.2 Hz), 1.19–1.30 (2H, m), 1.26 (3H, s), 1.29–1.43 (2H, m), 1.36 (3H, t, *J* = 6.1 Hz), 1.66 (1H, d, *J* = 12.8 Hz), 1.80–1.92 (2H, m), 2.92 (1H, d, *J* = 7.8 Hz), 3.30 (1H, d, *J* = 4.8 Hz), 4.20 (1H, s), 4.30 (1H, s), 4.91 (1H, s), 5.94 (1H, brs), 7.27–7.37 (5H, m). ^13^C NMR (125 MHz, CDCl_3_): δ = 22.4, 22.5, 22.9, 26.1, 27.3, 35.3, 41.8, 51.2, 58.0, 63.4, 65.4, 85.0, 85.4, 127.0, 127.8, 129.0, 143.0. Anal. Calcd for C_19_H_29_NO_2_: C, 75.21; H, 9.63; N, 4.62. Found: C, 75.19; H, 9.65; N, 4.63.

#### 4.6.7. (1*S*,2*R*,5*R*)-5-Methyl-2-((*S*)-5-methyl-3-((*S*)-1-phenylethyl)oxazolidin-5-yl)cyclohexanol (**27**)

Yield: 65%, colorless oil. ${\left[\alpha \right]}_{D}^{20}$ = +28.0 (c 0.20, MeOH). ^1^H NMR (500 MHz, CDCl_3_): δ = 0.88 (3H, d, *J* = 6.3 Hz), 0.89–1.08 (1H, m), 1.05 (1H, t, *J* = 12.8 Hz), 1.34 (3H, s), 1.41 (3H, d, *J* = 7.7 Hz), 1.37–1.42 (2H, m), 1.62–1.70 (1H, m), 1.75–1.82 (1H, m), 1.85–1.95 (2H, m), 2.04 (1H, d, *J* = 8.4 Hz), 3.27 (1H, s), 3.59 (1H, d, *J* = 7.3 Hz), 3.77 (1H, s), 4.26 (2H, s), 7.26–7.34 (5H, m). ^13^C NMR (125 MHz, CDCl_3_): δ = 22.5, 23.2, 26.3, 27.2, 35.4, 41.8, 51.2, 57.3, 62.6, 65.4, 85.2, 85.5, 126.7, 127.8, 129.0, 143.2. Anal. Calcd for C_19_H_29_NO_2_: C, 75.21; H, 9.63; N, 4.62. Found: C, 75.25; H, 9.60; N, 4.60.

#### 4.6.8. (1*S*,2*R*,5*R*)-2-((*S*)-3-Isopropyl-5-methyloxazolidin-5-yl)-5-methylcyclohexanol (**28**)

Yield: 70%, colorless oil. ${\left[\alpha \right]}_{D}^{20}$ = +14.0 (c 0.25, MeOH). ^1^H NMR (500 MHz, CDCl_3_): δ = 0.84 (3H, d, *J* = 6.4 Hz), 0.85–0.92 (1H, m), 1.00–1.06 (1H, m), 1.05 (3H, d, *J* = 6.2 Hz), 1.10 (3H, d, *J* = 6.3 Hz), 1.30 (3H, s), 1.32–1.37 (2H, m), 1.52–1.61 (1H, m), 1.71–1.74 (1H, m), 1.77–1.87 (2H, m), 1.91 (1H, d, *J* = 9.1 Hz), 2.40 (1H, quin, *J* = 6.2 Hz), 3.37 (1H, d, *J* = 9.1 Hz), 3.89 (1H, s), 4.22 (1H, s), 4.73 (1H, s), 6.17 (1H, brs). ^13^C NMR (125 MHz, CDCl_3_): δ = 21.7, 21.9, 22.5, 23.1, 26.2, 27.1, 35.4, 41.9, 51.4, 52.2, 56.8, 65.3, 84.8, 85.0. Anal. Calcd for C_14_H_27_NO_2_: C, 69.66; H, 11.27; N, 5.80. Found: C, 69.70; H, 11.25; N, 4.84.

### 4.7. General Procedure for Debenzylation

To a suspension of palladium-on-carbon (5% Pd, 0.22 g) in MeOH (50 mL) was added aminodiols **9**–**11, 20**–**22** or aminotriols **32**–**34** (14.0 mmol) in MeOH (100 mL), and the mixture was stirred under a H_2_ atmosphere (1 atm) at room temperature. After completion of the reaction (as monitored by TLC, 24 h), the mixture was filtered through a Celite pad and the solution was evaporated to dryness. The crude products were recrystallized in diethyl ether, resulting in primary aminodiols **13** and **24** or aminotriol **36**, respectively.

#### 4.7.1. (1*S*,2*S*,5*R*)-2-((*S*)-1-Amino-3-hydroxypropan-2-yl)-5-methylcyclohexanol (**13**)

Yield: 50%, colorless oil. ${\left[\alpha \right]}_{D}^{20}$ = +21.0 (c 0.25, MeOH). ^1^H NMR (500 MHz, CDCl_3_): δ = 0.85 (3H, d, *J* = 5.8 Hz), 0.90–0.95 (1H, m), 1.10 (1H, t, *J* = 12.3 Hz), 1.24 (1H, s), 1.35–1.45 (2H, m) 1.58 (1H, t, *J* = 12.0 Hz), 1.60–1.87 (5H, m), 2.85–3.00 (2H, m), 3.42 (4H, brs), 3.60–3.70 (1H, m), 3.70–3.80 (1H, m), 3.98 (1H, s). ^13^C NMR (125 MHz, CDCl_3_): δ = 22.5, 24.7, 26.2, 35.4, 40.9, 42.0, 42.4, 44.7, 62.8, 63.7, 66.7. Anal. Calcd for C_10_H_21_NO_2_: C, 64.13; H, 11.30; N, 7.48. Found: C, 64.15; H, 11.28; N, 7.50.

#### 4.7.2. (1*S*,2*R*,5*R*)-2-((*S*)-1-Amino-2-hydroxypropan-2-yl)-5-methylcyclohexanol (**24**)

Yield: 72%, white crystals, m.p.: 139–140 °C. ${\left[\alpha \right]}_{D}^{20}$ = +12.0 (c 0.295, MeOH). ^1^H NMR (500 MHz, DMSO-*d*_6_): δ = 0.82 (3H, d, *J* = 6.3 Hz), 0.78–0.87 (1H, m), 1.01 (1H, t, *J* = 12.6 Hz), 1.17 (3H, s), 1.30–1.33 (1H, m), 1.45–1.48 (2H, m), 1.70–1.75 (3H, m), 2.62 (1H, d, *J* = 12.8 Hz), 2.92 (1H, d, *J* = 12.9 Hz), 4.10 (1H, s), 4.95 (1H, s). ^13^C NMR (125 MHz, DMSO-*d*_6_): δ = 20.9, 22.3, 25.1, 25.4, 34.8, 42.6, 45.1, 49.7, 64.4, 71.0. Anal. Calcd for C_10_H_21_NO_2_: C, 64.13; H, 11.30; N, 7.48. Found: C, 64.15; H, 11.28; N, 7.50.

#### 4.7.3. (*S*)-3-Amino-2-((1*R*,2*S*,4*R*)-2-hydroxy-4-methylcyclohexyl)propane-1,2-diol (**36**)

Yield: 72%, colorless oil. ${\left[\alpha \right]}_{D}^{20}$ = +22.0 (c 0.275, MeOH). ^1^H NMR (500 MHz, DMSO-*d*_6_): δ = 0.82 (3H, d, *J* = 6.3 Hz), 0.82–0.88 (1H, m), 1.02 (1H, t, *J* = 13.2 Hz), 1.45–1.50 (3H, m), 1.67–1.74 (3H, m), 2.78–2.87 (2H, m), 3.31–3.47 (2H, m), 4.12 (1H, s), 4.89 (1H, brs), 5.14 (1H, brs), 5.37 (1H, brs), 7.65 (3H, s). ^13^C NMR (125 MHz, DMSO-*d*_6_): δ = 20.6, 22.2, 25.4, 34.7, 45.5, 42.2, 45.2, 64.5, 65.8, 73.2. Anal. Calcd for C_10_H_21_NO_3_: C, 59.08; H, 10.41; N, 6.89. Found: C, 59.10; H, 10.45; N, 6.85.

### 4.8. General Procedure for Dihydroxylation

To a solution of compounds **2**–**4** (14 mmol) in acetone (60 mL), an aqueous solution of NMO (12 mL, 50% aqueous solution) and a solution of OsO_4_ in *t*-BuOH (6 mL, 2% *t*-BuOH solution) were added in one portion. The reaction mixture was stirred at room temperature for 24 h, and then quenched by the addition of a saturated aqueous solution of Na_2_SO_3_ (100 mL) and extracted with EtOAc (3 x 80 mL). The organic layer was dried and evaporated. The crude products were purified by chromatography on silica gel by using *n*-hexane:EtOAc (1:4 for compounds **18** and **29** and 1:9 for compound **37**). The products after purification were recrystallized in diethyl ether resulting in compounds **18**, **29** and **37** as white crystals.

#### 4.8.1. (3*R*,3a*R*,6*R*,7a*S*)-3-Hydroxy-3-(hydroxymethyl)-6-methylhexahydrobenzofuran-2(3*H*)-one (**18**)

Yield: 50%, white crystals, m.p.: 73–75 °C. ${\left[\alpha \right]}_{D}^{20}$ = −63.0 (c 0.26, MeOH). ^1^H NMR (500 MHz, DMSO-*d*_6_): δ = 0.86 (3H, d, *J* = 6.4 Hz), 0.83–0.95 (2H, m), 1.19–1.25 (1H, m), 1.34–1.39 (1H, m), 1.56 (1H, d, *J* = 12.4 Hz), 1.73–1.77 (1H, m), 2.05–2.13 (2H, m), 3.46 (1H, dd, *J* = 6.0, 11.9 Hz), 3.54 (1H, dd, *J* = 4.8, 12.0 Hz), 4.77 (1H, t, *J* = 5.7 Hz), 4.81 (1H, q, *J* = 3.4 Hz), 5.84 (1H, s). ^13^C NMR (125 MHz, DMSO-*d*_6_): δ = 21.1, 21.2, 25.9, 31.6, 35.4, 43.0, 61.0, 77.1, 79.5, 176.0. Anal. Calcd for C_10_H_16_O_4_: C, 63.80; H, 10.71. Found: C, 63.83; H, 10.69.

#### 4.8.2. (*R*)-2-((1*R*,2*S*,4*R*)-2-Hydroxy-4-methylcyclohexyl)propane-1,2-diol (**29**)

Yield: 61%, white crystals, m.p.: 137–139 °C. ${\left[\alpha \right]}_{D}^{20}$ = +12.0 (c 0.275, MeOH). ^1^H NMR (500 MHz, DMSO-*d*_6_): δ = 0.80 (3H, d, *J* = 6.5 Hz), 0.85–0.79 (1H, m), 0.95 (1H, t, *J* = 11.9 Hz), 1.05 (3H, s), 1.30–1.32 (1H, m), 1.48–1.57 (2H, m), 1.65–1.74 (3H, m), 3.11 (1H, dd, *J* = 5.7, 10.5 Hz), 3.43 (1H, dd, *J* = 5.2, 10.5 Hz), 4.12 (1H, s), 4.40 (1H, s), 4.80 (1H, t, *J* = 5.4 Hz), 4.83 (1H, d, *J* = 1.9 Hz). ^13^C NMR (125 MHz, DMSO-*d*_6_): δ = 19.8, 22.4, 23.8, 25.4, 34.9, 42.4, 45.3, 65.8, 67.3, 73.9. Anal. Calcd for C_10_H_20_O_3_: C, 63.80; H, 10.71. Found: C, 63.78; H, 10.75.

#### 4.8.3. 2-((1*R*,2*S*,4*R*)-2-Hydroxy-4-methylcyclohexyl)propane-1,2,3-triol (**37**)

Yield: 40%, white crystals, m.p.: 93–95 °C. ${\left[\alpha \right]}_{D}^{20}$ = +17.0 (c 0.235, MeOH). ^1^H NMR (500 MHz, DMSO-*d*_6_): δ = 0.80 (3H, d, *J* = 6.5 Hz), 0.82–0.85 (1H, m), 0.96 (1H, t, *J* = 12.2 Hz), 1.46–1.57 (3H, m), 1.65–1.68 (2H, m), 1.73–1.75 (1H, m), 3.38–3.43 (2H, m), 4.09 (1H, s), 4.26 (1H, s), 4.49 (1H, t, *J* = 5.6 Hz), 4.73 (1H, t, *J* = 5.4 Hz), 4.95 (1H, d, *J* = 1.0 Hz). ^13^C NMR (125 MHz, DMSO-*d*_6_): δ = 19.8, 22.4, 25.4, 35.0, 42.3, 43.3, 63.1, 63.2, 65.4, 75.6, 75.6. Anal. Calcd for C_10_H_20_O_4_: C, 58.80; H, 9.87. Found: C, 58.83; H, 9.85.

### 4.9. (S)-4-((1R,2S,4R)-2-Hydroxy-4-methylcyclohexyl)-4-methyl-1,3-dioxolan-2-one *(**30**)*

To the solution of **29** (2.00 mmol) in dry pyridine (8.22 mmol) and dry CH_2_Cl_2_ (20 mL), triphosgene (1.00 mmol) in dry CH_2_Cl_2_ (5 mL) was added under cooling in an ice bath. After stirring at room temperature for 2 h under Ar atmosphere, the consumption of **29** was confirmed by TLC. Then water (20 mL) was added to the solution and then the organic layer was washed with saturated aqueous NH_4_Cl solution. The organic layer was collected, dried over anhydrous Na_2_SO_4_ and filtered. The crude product after evaporation was purified by column chromatography on silica gel (*n*-hexane:EtOAc = 4:1) to obtain **30** in a 60% yield.

Yield: 60%, colorless oil. ${\left[\alpha \right]}_{D}^{20}$ = +35.0 (c 0.28, MeOH). ^1^H NMR (500 MHz, CDCl_3_): δ = 0.95 (3H, d, *J* = 6.2 Hz), 1.16–1.25 (1H, m), 1.57 (3H, s), 1.58–1.70 (2H, m), 1.74–1.80 (1H, m), 1.94–2.08 (2H, m), 2.13–2.20 (1H, m), 4.11 (1H, d, *J* = 8.2 Hz), 4.27 (1H, d, *J* = 8.2 Hz), 5.78 (1H, t, *J* = 1.8 Hz). ^13^C NMR (125 MHz, CDCl_3_): δ = 21.6, 24.0, 24.3, 28.1, 30.6, 33.4, 74.2, 85.0, 123.4, 135.6, 154.8. Anal. Calcd for C_11_H_18_O_4_: C, 61.66; H, 8.47. Found: C, 66.63; H, 10.51.

#### (*R*)-2-((1*R*,2*S*,4*R*)-2-Hydroxy-4-methylcyclohexyl)propane-1,2-diol (**29**)

Compound **19** (0.60 mmol) was treated with DMSO (3.0 mL) and 3 M NaOH (3.0 mL). The resulting homogenous solution was stirred at 80 °C for 2 hours. After being cooled to room temperature, EtOAc (20 mL) was added and the aqueous layer was washed with EtOAc (3 x 20 mL). The combined organic layers were dried over Na_2_SO_4_, filtered and concentrated in vacuo. The crude material was purified by column chromatography on silica gel (*n*-hexane:EtOAc = 1:4) then recrystallized in Et_2_O to provide compound **29** (60%).

### 4.10. General Procedure for the Reaction of Benzaldehyde with Diethylzinc in the Presence of Chiral Catalysts

To the respective catalyst (0.1 mmol), 1 M Et_2_Zn in *n*-hexane solution (3 mL, 3 mmol) was added under an argon atmosphere at room temperature. The solution was stirred for 25 min at room temperature then benzaldehyde (1 mmol) was added. After stirring at room temperature for a further 20 h, the reaction was quenched with saturated NH_4_Cl solution (15 mL) and the mixture was extracted with EtOAc (2 × 20 mL). The combined organic phase was washed with H_2_O (10 mL), dried (Na_2_SO_4_) and evaporated under vacuum. The crude secondary alcohols obtained were purified by flash column chromatography (*n*-hexane:EtOAc = 4:1). The *ee* and absolute configuration of the resulting material were determined by chiral GC on CHIRASIL-DEX CB column after *O*-acetylation in Ac_2_O/DMPA/pyridine.

### 4.11. Antimicrobial Analyses

For the antimicrobial analyses, the pure compounds were first dissolved in MeOH and diluted with H_2_O to two concentration levels (400 µg/mL and 40 µg/mL)—keeping the final MeOH content at 10%. Then, these solutions were investigated in microdilution assay with two Gram-positive bacteria including *Bacillus subtilis* SZMC 0209 and *Staphylococcus aureus* SZMC 14611, two Gram-negative bacteria *Escherichia coli* SZMC 6271 and *Pseudomonas aeruginosa* SZMC 23290, as well as two yeast strains *Candida albicans* SZMC 1533 and *C. krusei* SZMC 1352 according to the M07-A10 CLSI guideline [55] and our previous work [56]. Suspensions of the test microbes were prepared from overnight cultures cultivated in ferment broth (bacteria: 10 g/L peptone, 5 g/L NaCl, 5 g/L yeast extract; yeast: 20 g/L peptone, 10 g/L yeast extract, 20 g/L glucose) at 37 °C. Then the concentrations of the suspensions were set to 2 x 10^5^ cells/mL with sterile media. For the assay, 96-well plates were prepared by dispensing into each well 100 μL of suspension containing the bacterial or yeast cells and 50 μL of sterile broth as well as 50 μL of the test solutions and incubated for 24 h at 37 °C. The mixture of 150 μL broth and 50 μL of 10% methanol was used as the blank sample for the background correction, while 100 μL of microbial suspension supplemented with 50 μL sterile broth and 50 μL of 10% methanol was applied as negative control. The positive control contained ampicillin (Sigma) or nystatin (Sigma) for bacteria or fungi, respectively at two concentration levels (100 µg/mL and 10 µg/mL). The inhibitory effects of the derivatives were observed spectrophotometrically at 620 nm after the incubation, and inhibition was calculated as the percentage of the positive control after blank correction. 

## 5. Conclusions

Starting from the commercially available (−)-isopugeol, a new family of isopulegol-based chiral aminodiol, aminotriol libraries and di-, tri- and tetraols were prepared through chiral (+)-neoisopulegol as key intermediate via stereoselective transformations. 

The resulting aminodiols exert moderate antimicrobial action on a panel of bacterial and fungal strains, while aminodiol and aminotriol derivatives were applied as chiral catalysts in the enantioselective addition of diethylzinc to benzaldehyde with moderate but significantly opposite stereoselectivity.

## Figures and Tables

**Table 1:** Addition of diethylzinc to benzaldehyde, catalyzed by aminodiol derivatives and aminotriols.

Entry	Ligand	Yield ^a^ (%)	*ee*^b^ (%)	Configuration ^c^
1	**9**	90	18	(*R*)
2	**10**	92	60	(*R*)
3	**11**	95	43	(*R*)
4	**12**	95	17	(*R*)
5	**22**	97	18	(*R*)
6	**25**	93	37	(*R*)
7	**32**	86	19	(*S*)
8	**34**	87	28	(*S*)
9	**35**	80	16	(*S*)

^a^ After silica column chromatography. ^b^ Determined using the crude product by GC (Chirasil-DEX CB column). ^c^ Determined by comparing the t_R_ of GC analysis and optical rotations with literature data [14,54].

**Table 2:** Antimicrobial activities of the synthesized compounds.

				Inhibitory Effect (%) ± RSD (%)			
		*Yeast*		*Gram-negative*		*Gram-positive*	
Analogue	Conc. (µg/mL)	*C. albicans*	*C. krusei*	*E. coli*	*P. aeruginosa*	*B. subtilis*	*S. aureus*
**9**	10	−	38.2 ± 4.2	−	−	−	−
	100	−	41.8 ± 1.2	24.8 ± 1.3	−	−	−
**10**	10	−	−	21.1 ± 8.0	−	−	−
	100	−	−	25.8 ± 10.1	19.5 ± 0.5	−	−
**11**	10	0.2 ± 0.9	0.9 ± 0.5	−	22.6 ± 2.1	18.4 ± 0.7	8.5 ± 0.6
	100	2.2 ± 2.3	3.3 ± 4.7	0.2 ± 1.8	41.6 ± 12.2	21.0 ± 7.5	9.8 ± 4.0
**12**	10	−	−	−	5.4 ± 0.3	−	8.5 ± 6.3
	100	−	−	−	14.3 ± 4.5	−	9.4 ± 5.4
**13**	10	−	−	−	−	26.1 ± 8.3	24.5 ± 15.6
	100	−	40.4 ± 2.4	−	−	42.9 ± 20.0	25.2 ± 1.1
**14**	10	−	8.4 ± 4.1	−	3.2 ± 7.1	−	−
	100	−	6.7 ± 2.0	25.6 ± 2.1	4.4 ± 5.8	−	12.6 ± 4.5
**15**	10	−	1.9 ± 0.7	−	8.6 ± 2.5	−	3.6 ± 1.4
	100	−	2.8 ± 4.2	18.0 ± 1.8	20.1 ± 0.2	−	10.4 ± 1.5
**16**	10	−	−	−	4.4 ± 5.8	−	−
	100	−	48.9 ± 0.1	15.7 ± 1.7	8.4 ± 5.1	4.6 ± 12.5	−
**17**	10	−	−	−	15.2 ± 10.4	−	−
	100	−	33.1 ± 0.4	8.5 ± 2.06	16.7 ± 7.2	−	−
**18**	10	−	−	−	−	16.9 ± 17.7	34.4 ± 11.7
	100	−	−	−	−	27.1 ± 16.0	34.2 ± 2.6
**20**	10	−	−	−	2.0 ± 0.9	10.6 ± 6.4	−
	100	4.1 ± 1.6	−	35.4 ± 0.8	19.9 ± 4.8	11.5 ± 1.3	19.7 ± 7.2
**24**	10	−	34.6 ± 3.3	−	−	47.6 ± 10.6	30.0 ± 2.0
	100	23.6 ± 1.2	37.8 ± 3.6	−	−	55.1 ± 19.9	33.9 ± 4.0
**29**	10	−	−	−	−	−	32.4 ± 4.1
	100	−	−	−	−	21.2 ± 5.2	34.7 ± 6.6
**32**	10	−	−	−	−	−	−
	100	−	−	−	−	−	24.6 ± 11.9
	10	−	−	−	−	14.9 ± 13.8	31.2 ± 7.9
**36**	100		39.9 ± 4.1	−	−	43.1 ± 2.6	33.3 ± 2.1
**37**	10	−	−	−	−	−	31.6 ± 15.1
	100	−	26.8 ± 7.9	−	−	40.9 ± 16.6	32.8 ± 8.2

## Supplementary Materials

The following are available online at https://www.mdpi.com/1422-0067/20/16/4050/s1.

## Abbreviations

Et_2_O	Diethyl ether
EtOH	Ethanol
HCHO	Formaldehyde
EtOAc	Ethyl acetate
*t*-BuOOH	*tert*-Butyl hydroperoxide
VO(acac)_2_	Vanadyl aceatylacetonate
NMO	4-Methylmorpholine *N*-oxide
MeCN	Acetonitrile
THF	Tetrahydrofuran
LiAlH_4_	Lithium aluminum hydride
