# Supplementary material for: Stereoselective Synthesis and Investigation of Isopulegol-Based Chiral Ligands
Source: Int J Mol Sci. 2019 Aug 19;20(16):4050. doi: 10.3390/ijms20164050 (PMC6719113; doi:10.3390/ijms20164050)

Supporting informations
for

Synthesis and transformation of isopulegol-based chiral ligands

Tam Minh Le [1], Tamás Szilasi [1], Volford Bettina[3], Szekeres Ándras[3], Ferenc Fülöp [1,2] and Zsolt Szakonyi [1,4*]

[1] Institute of Pharmaceutical Chemistry, University of Szeged, H-6720 Szeged, Eötvös utca 6, Hungary; leminhtam@pharm.u-szeged.hu; szilasitomi19@gmail.com; fulop@pharm.u-szeged.hu
[2] Stereochemistry Research Group of the Hungarian Academy of Sciences, H-6720 Szeged, Eötvös utca 6, Hungary
[3] Department of Microbiology, University of Szeged, 6726 Szeged, Közép fasor 52, Hungary; bettina.volford86@gmail.com; andras.j.szekeres@gmail.com
[4] Interdisciplinary Centre of Natural Products, University of Szeged, H-6720 Szeged, Eötvös utca 6, Hungary
* Correspondence: szakonyi@pharm.u-szeged.hu; Tel.: +36-62-546809; Fax: +36-62-545705

Contents

^{1}H, ^{13}C NMR spectra of new compounds 3 - 133

^{1}H-NMR of compound **8**

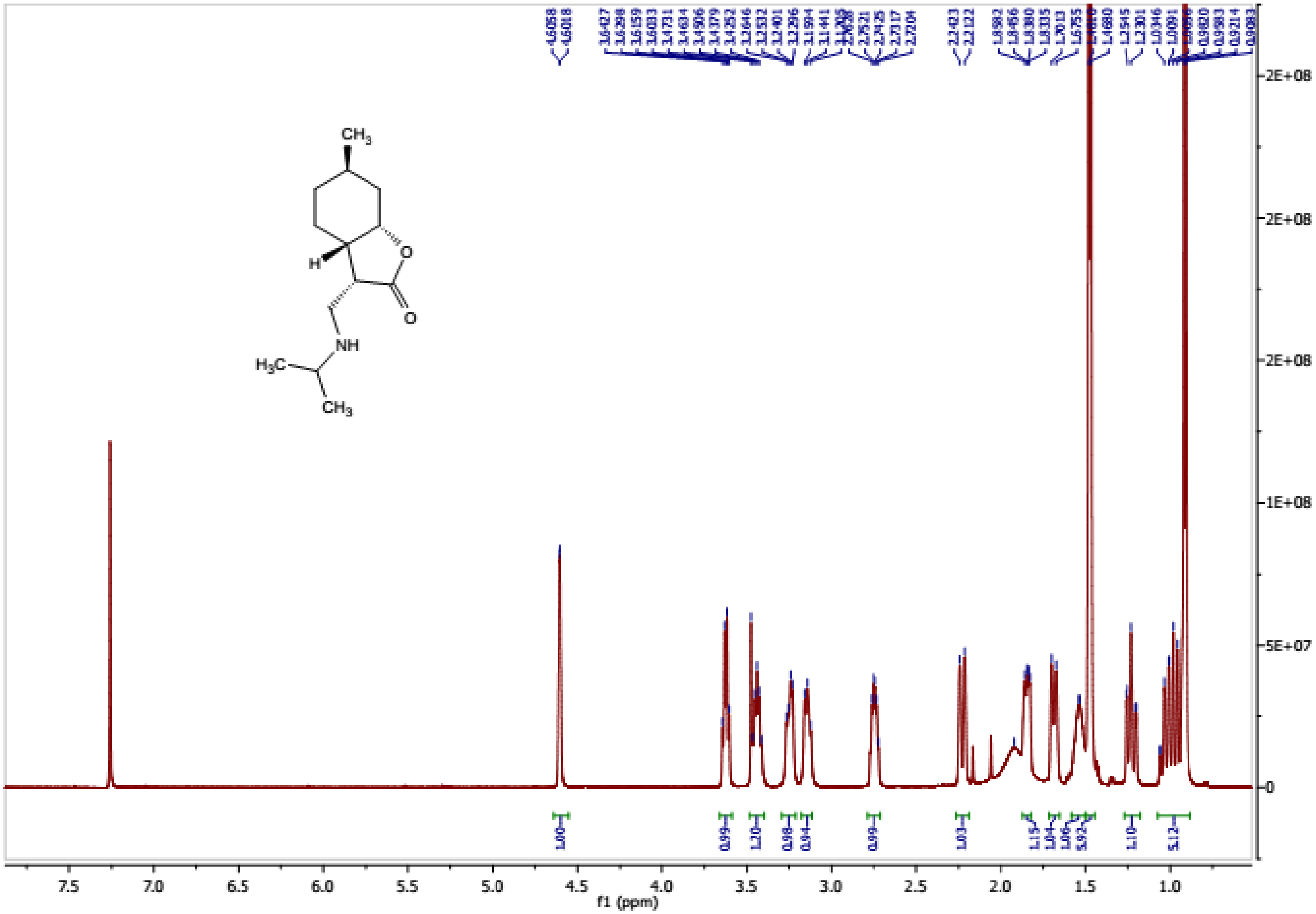

^{13}C-NMR of compound **8**

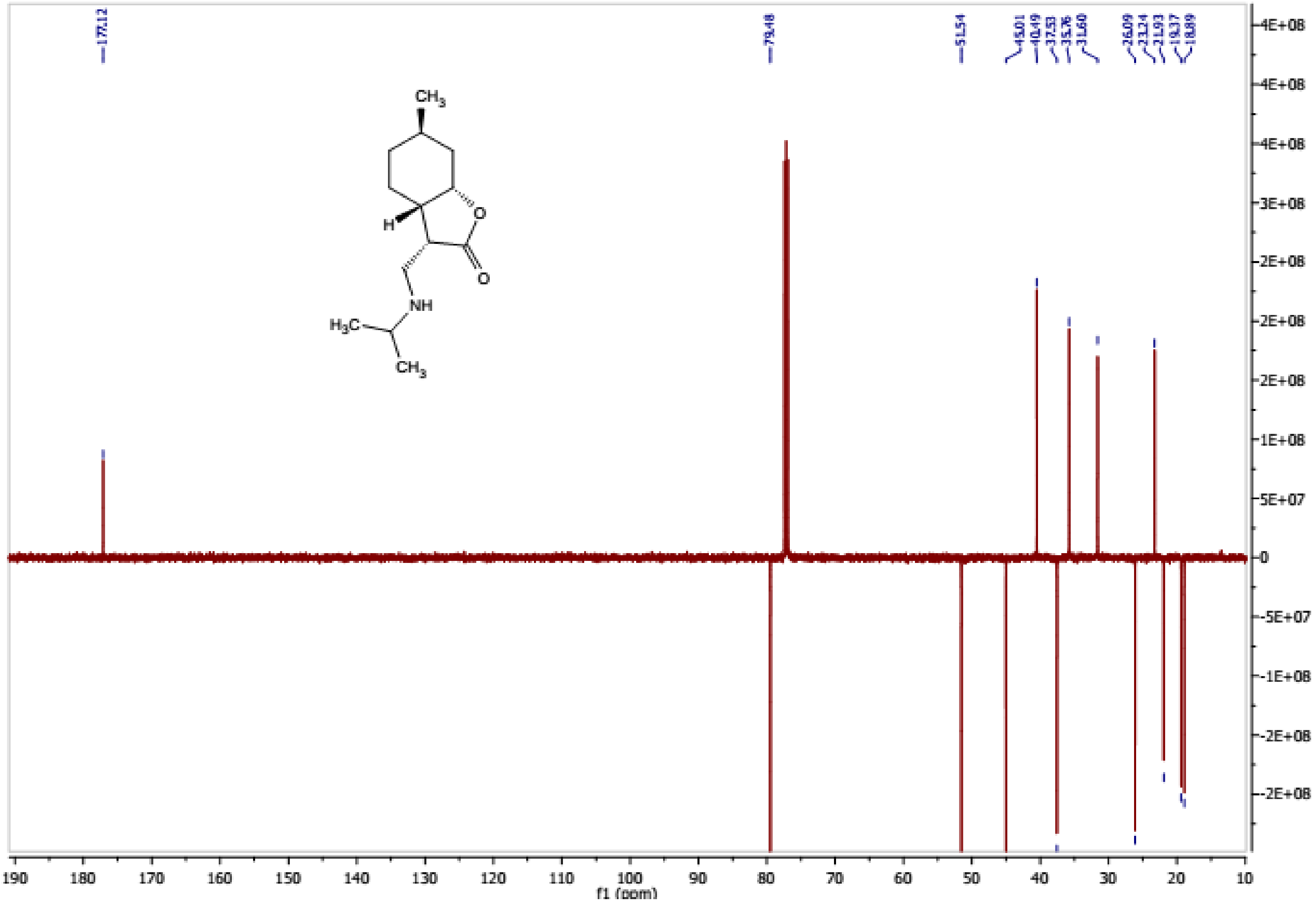

^{1}H-NMR of compound **9**

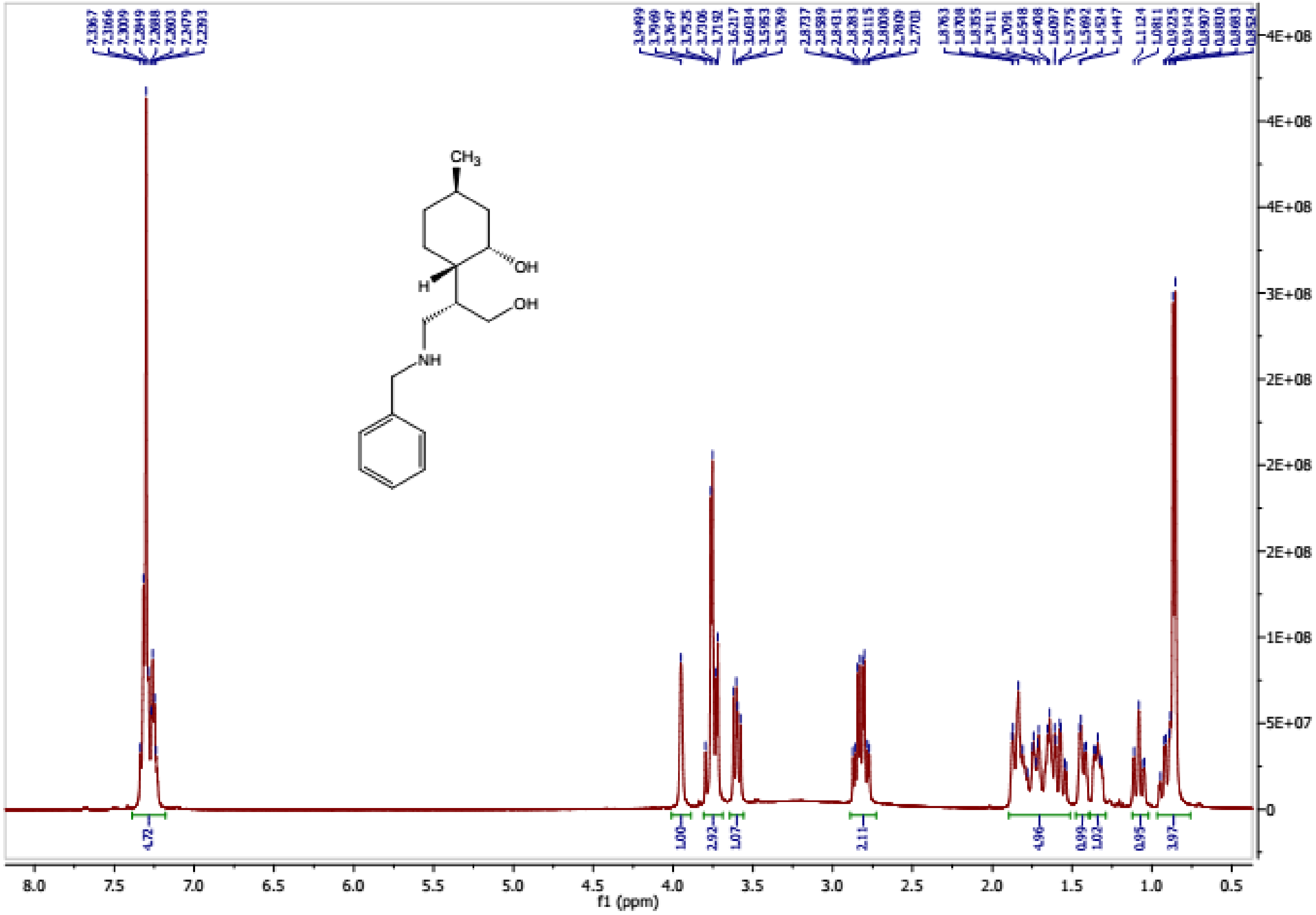

^{13}C-NMR of compound **9**

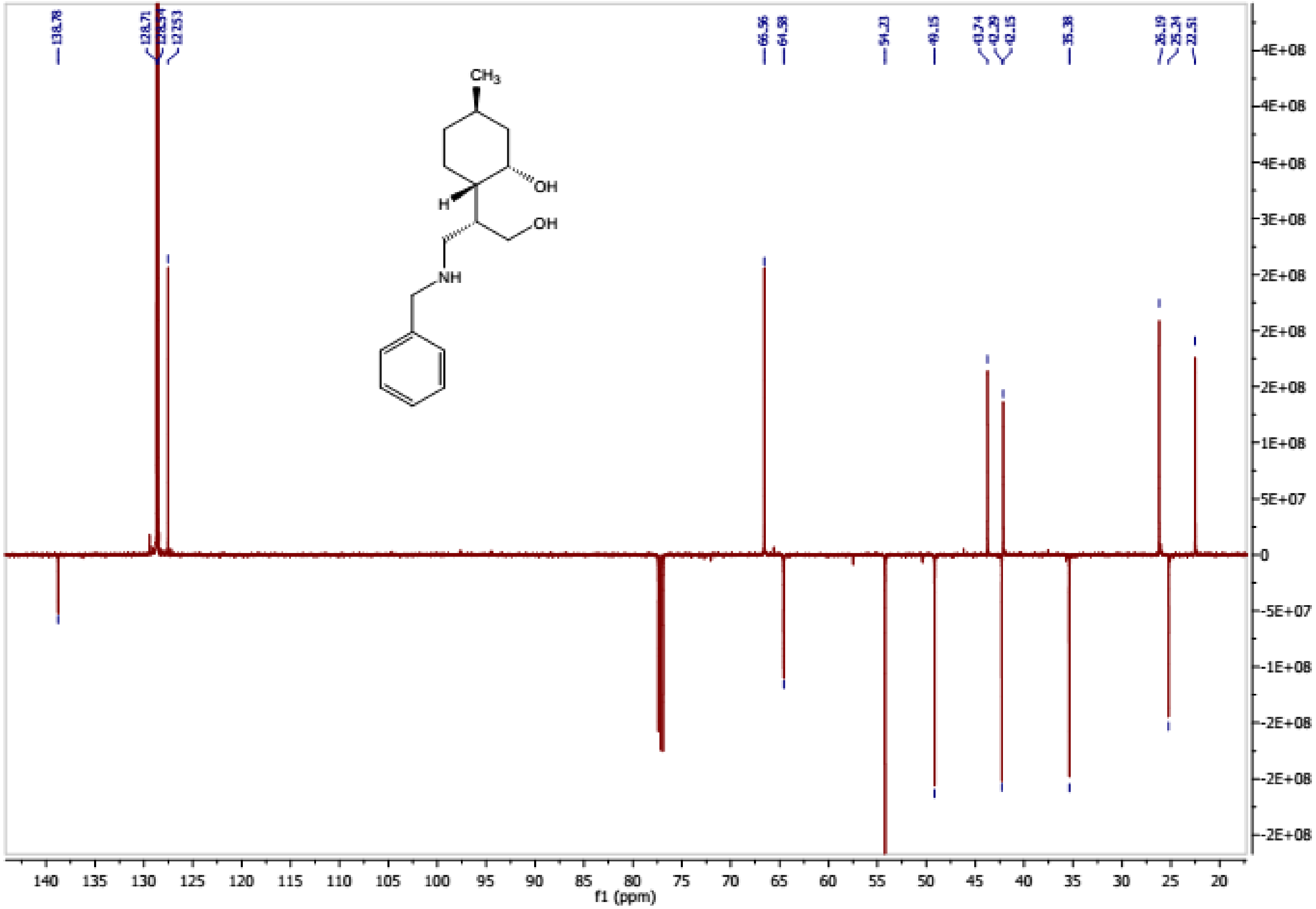

^{1}H-NMR of compound **10**

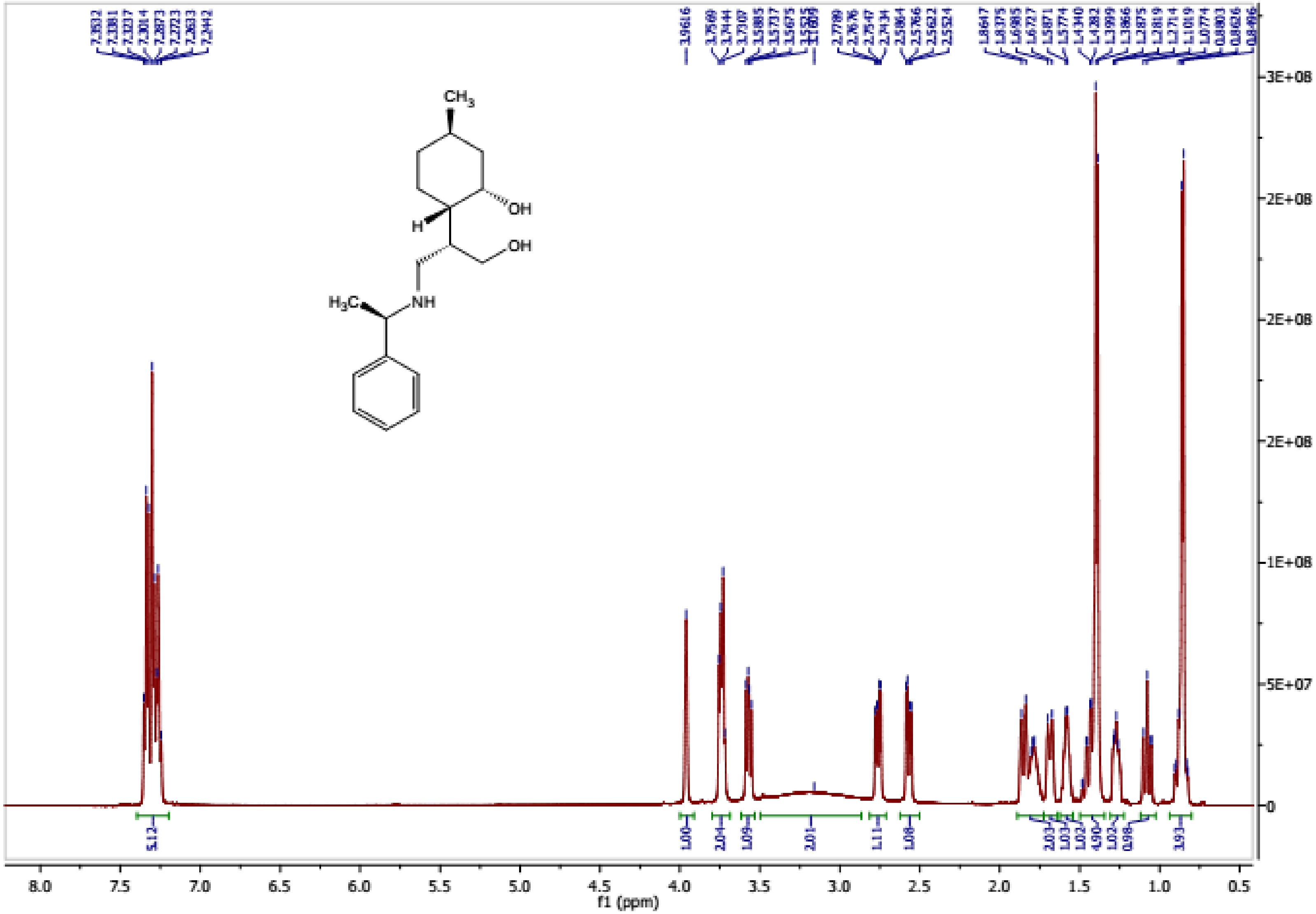

^{13}C-NMR of compound **10**

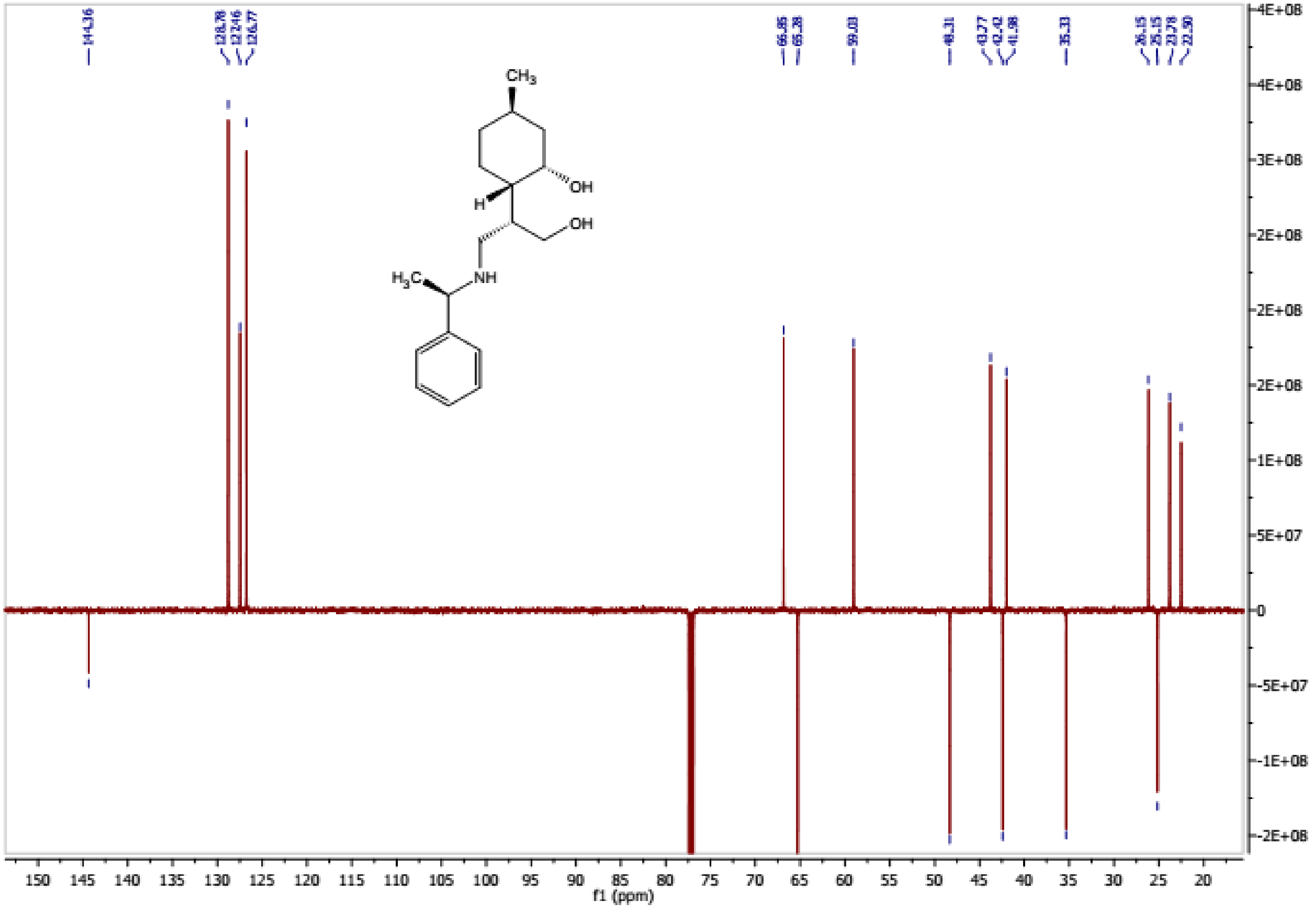

^{1}H-NMR of compound **11**

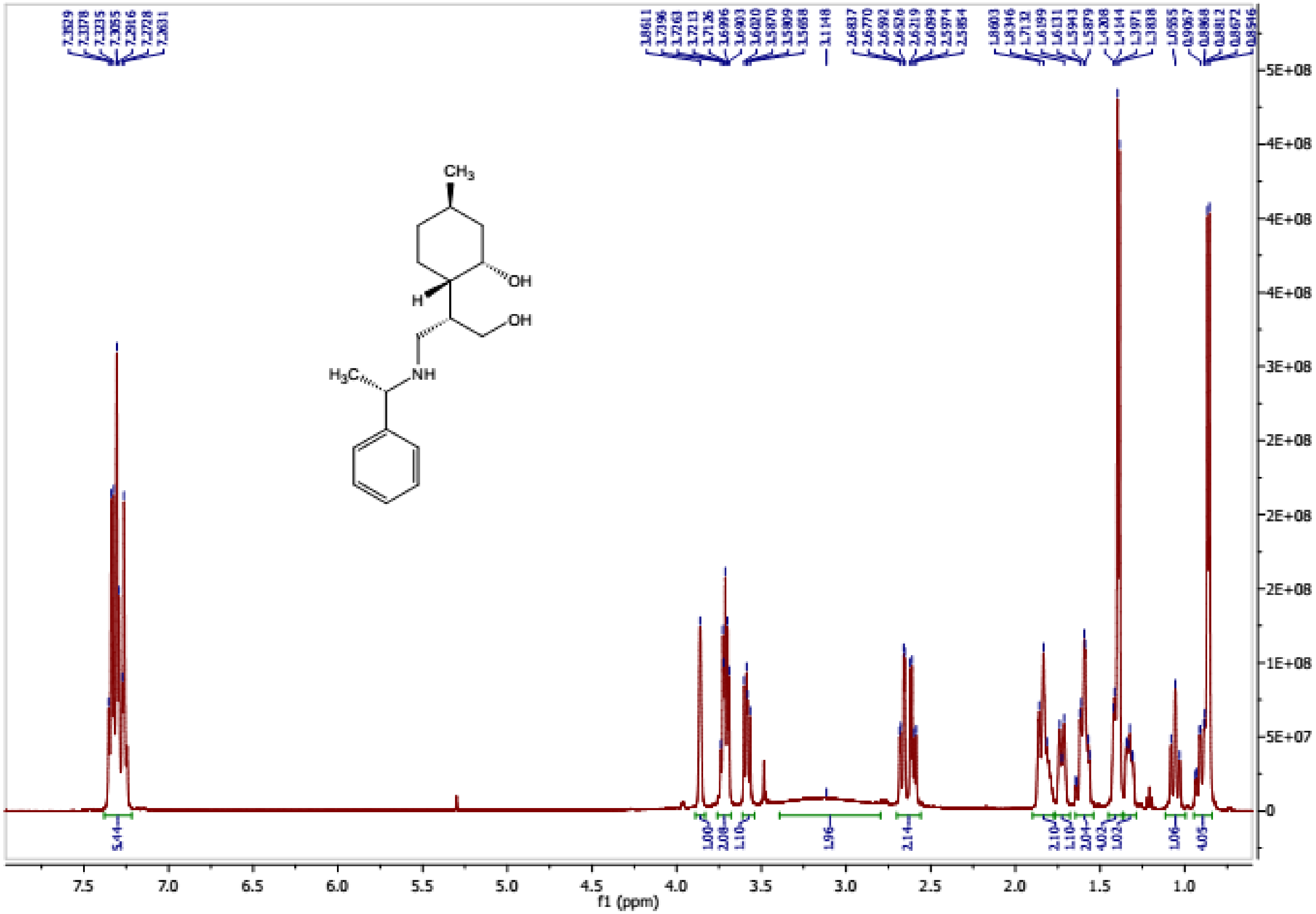

^{13}C-NMR of compound **11**

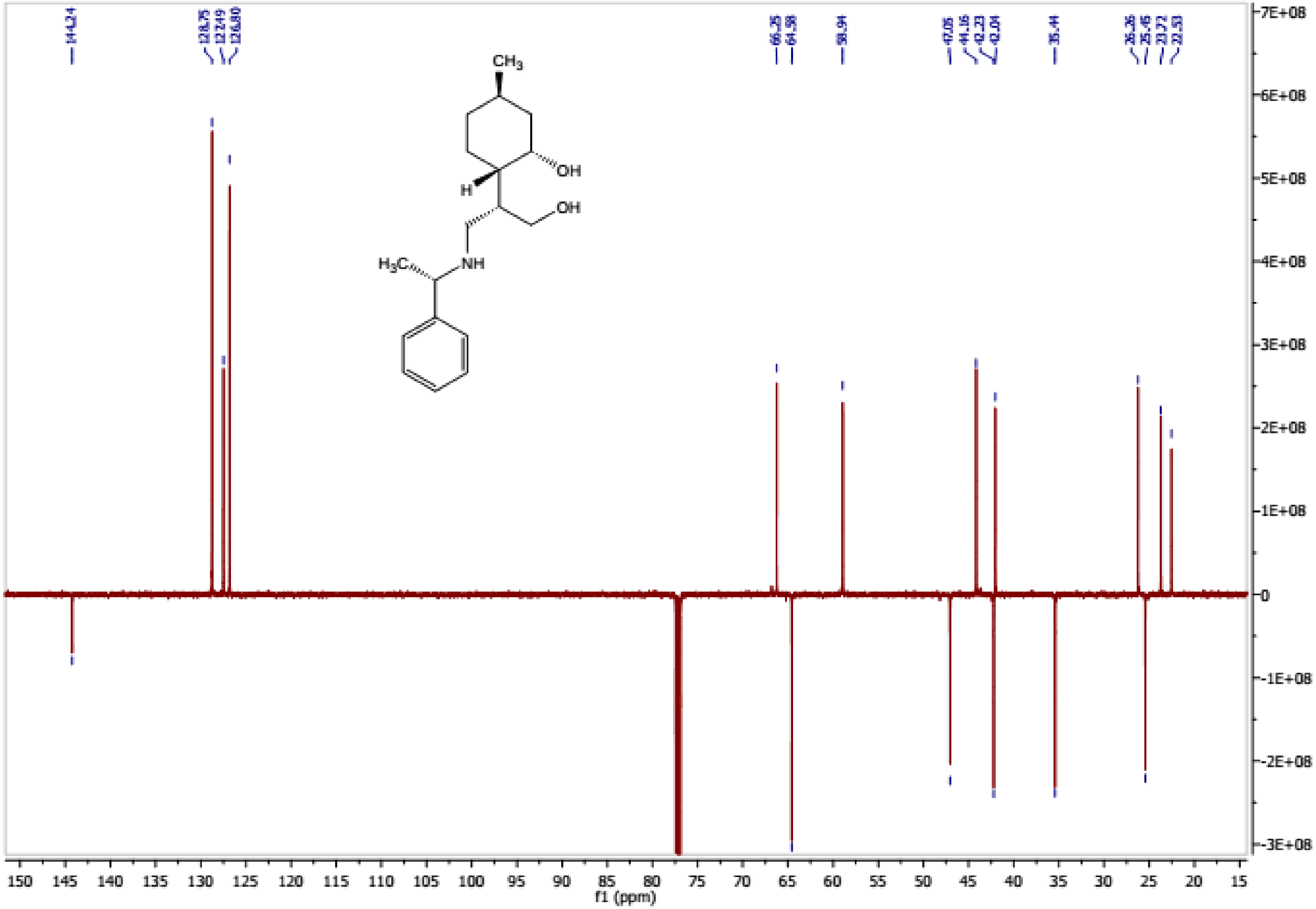

^{1}H-NMR of compound **12**

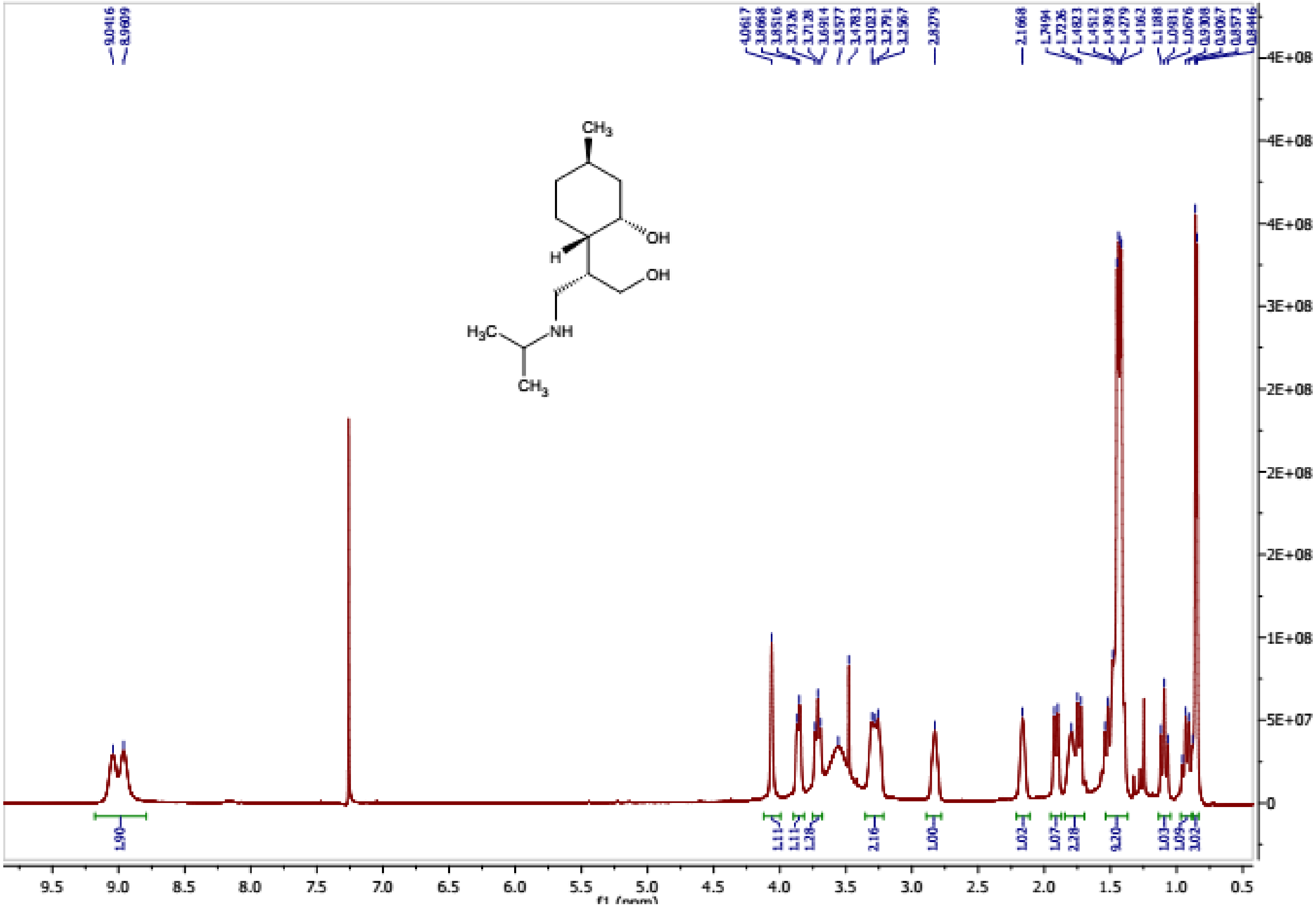

^{13}C-NMR of compound **12**

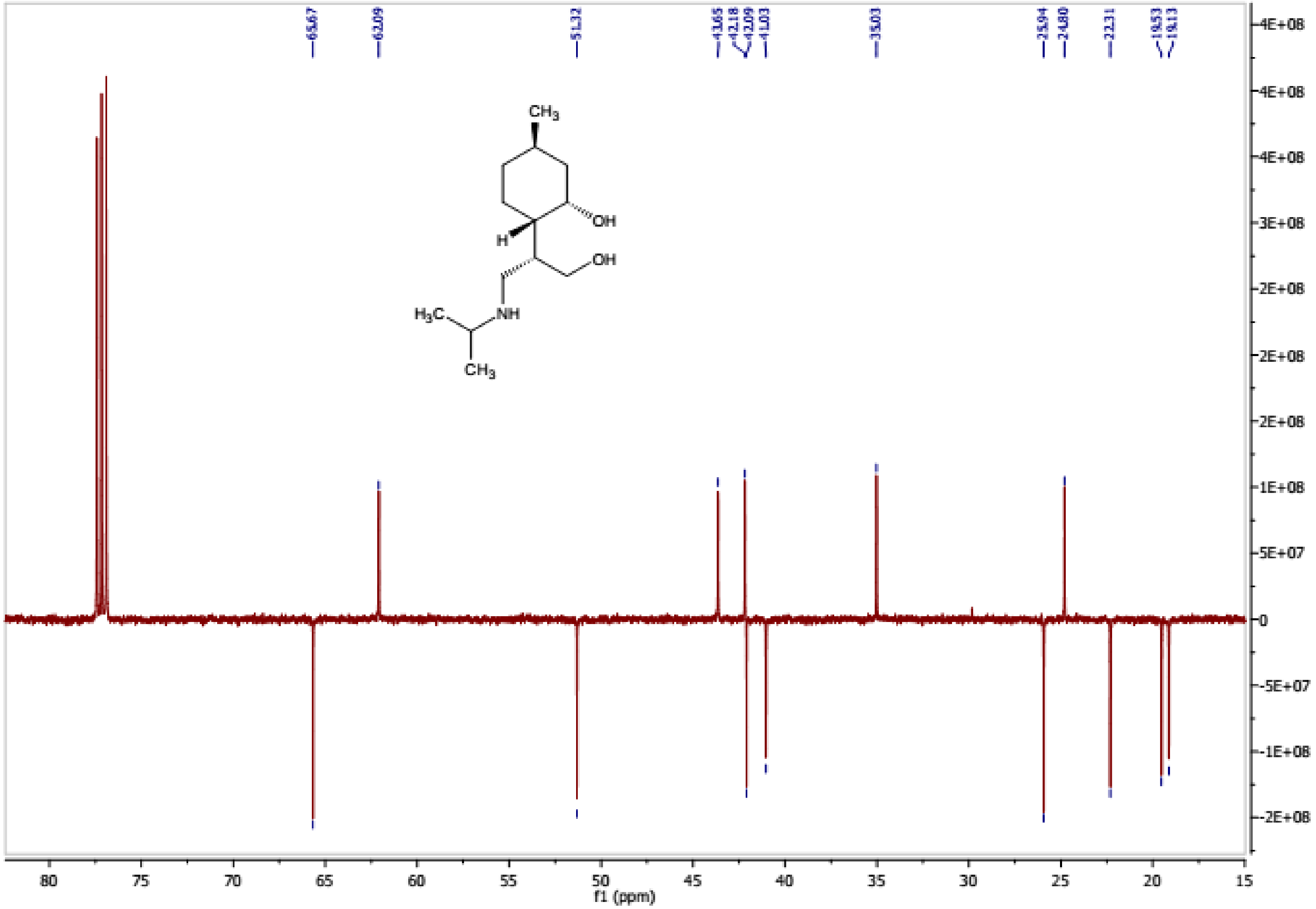

^{1}H-NMR of compound **13**

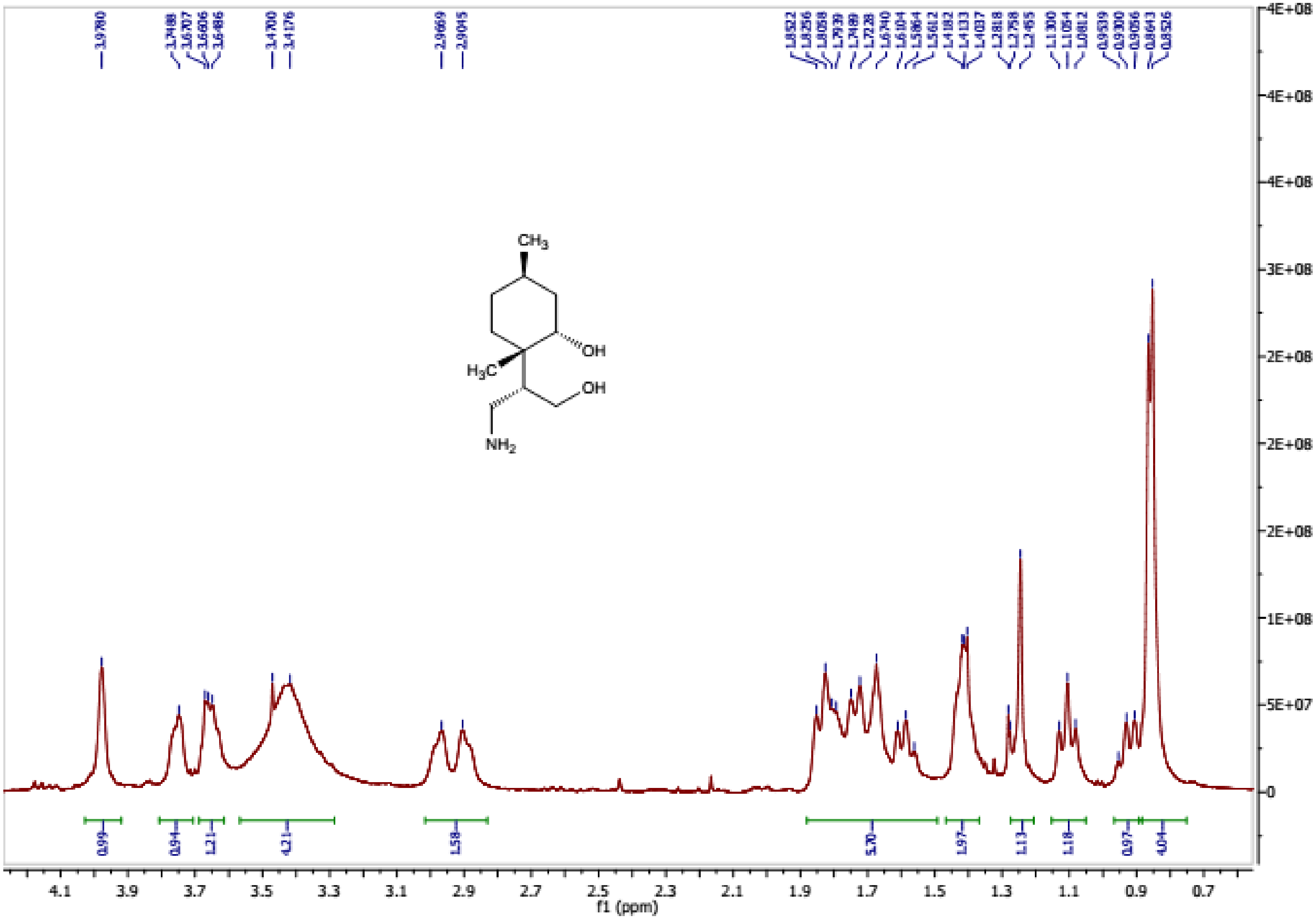

^{13}C-NMR of compound **13**

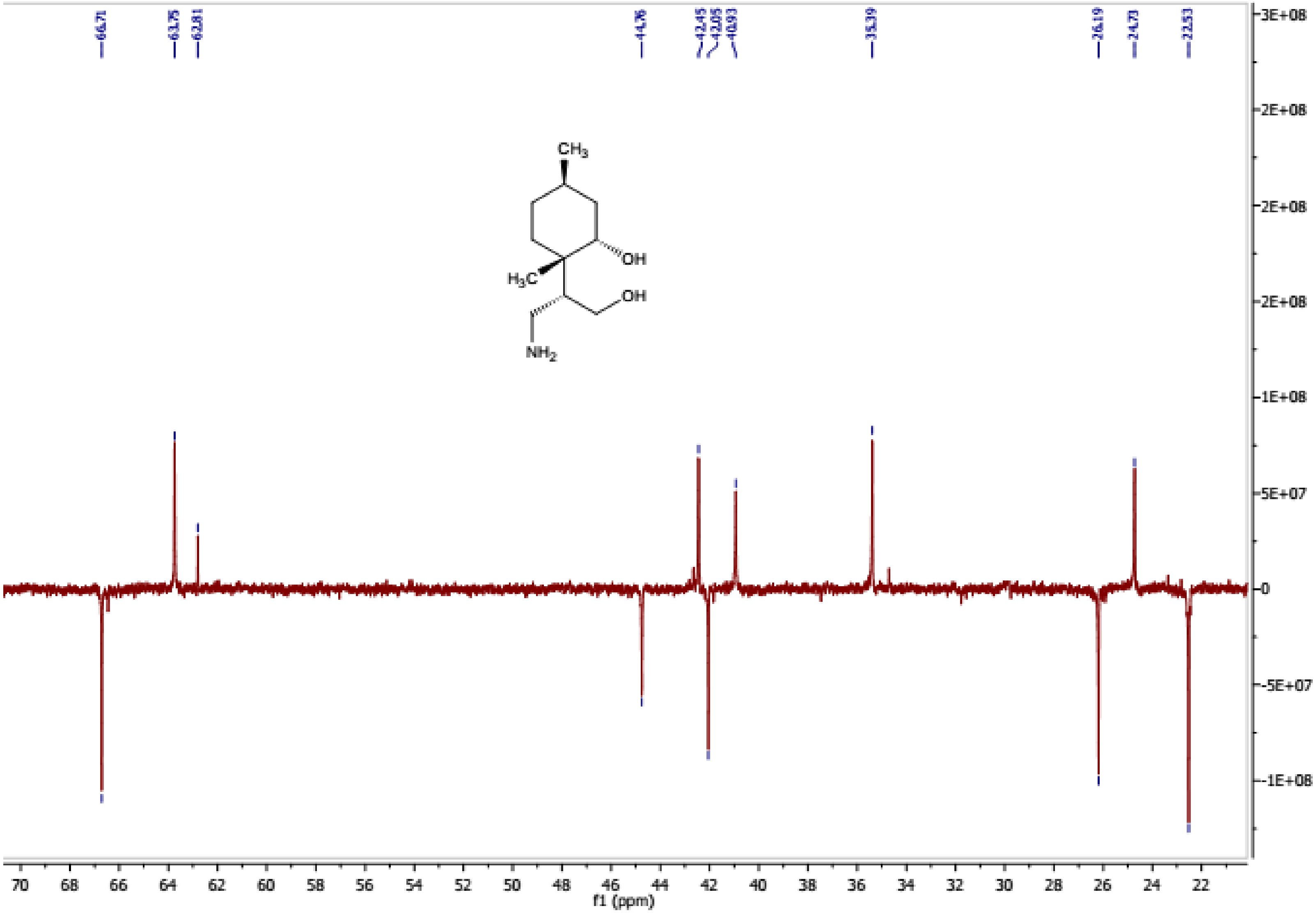

^{1}H-NMR of compound **14**

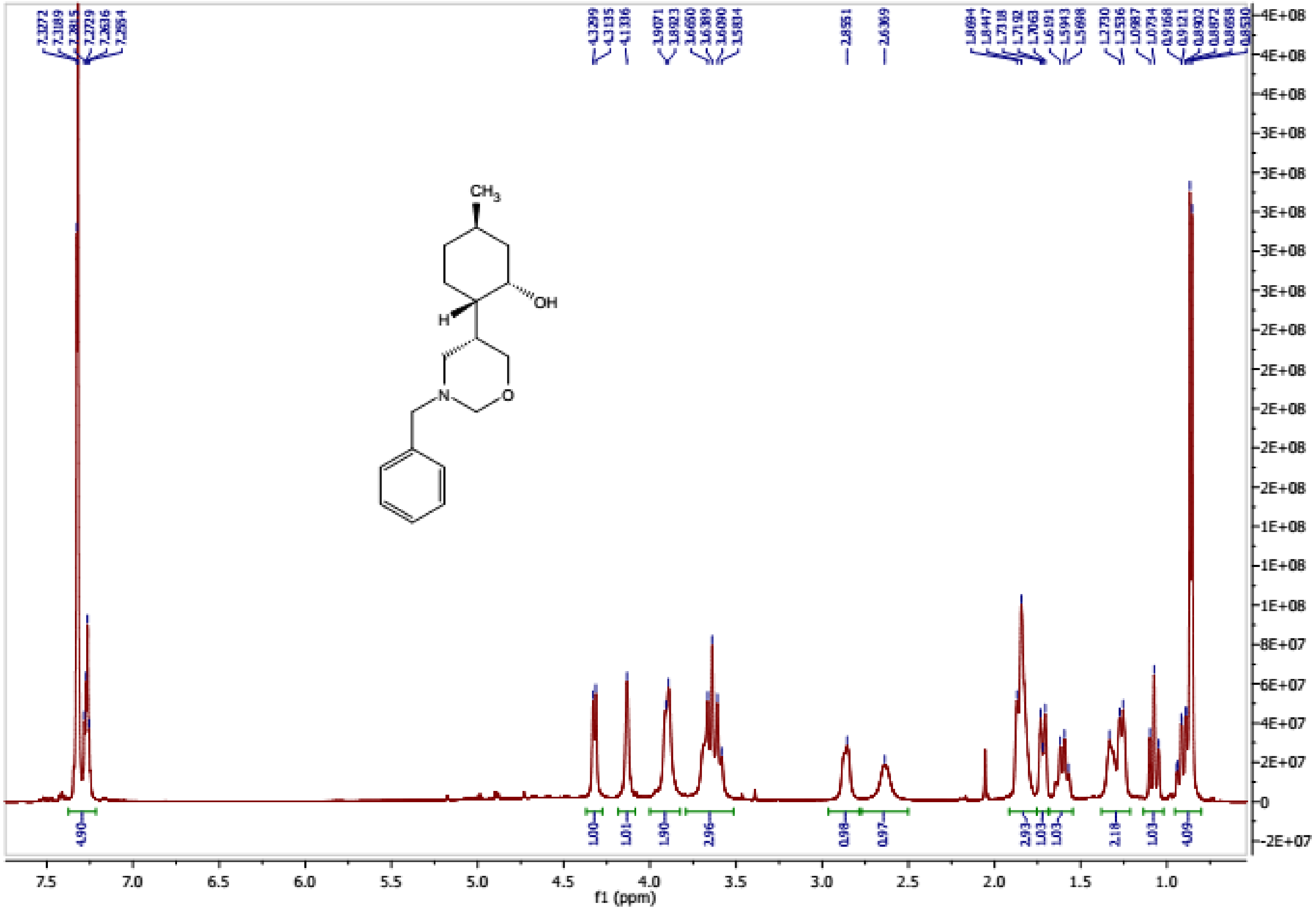

^{13}C-NMR of compound **14**

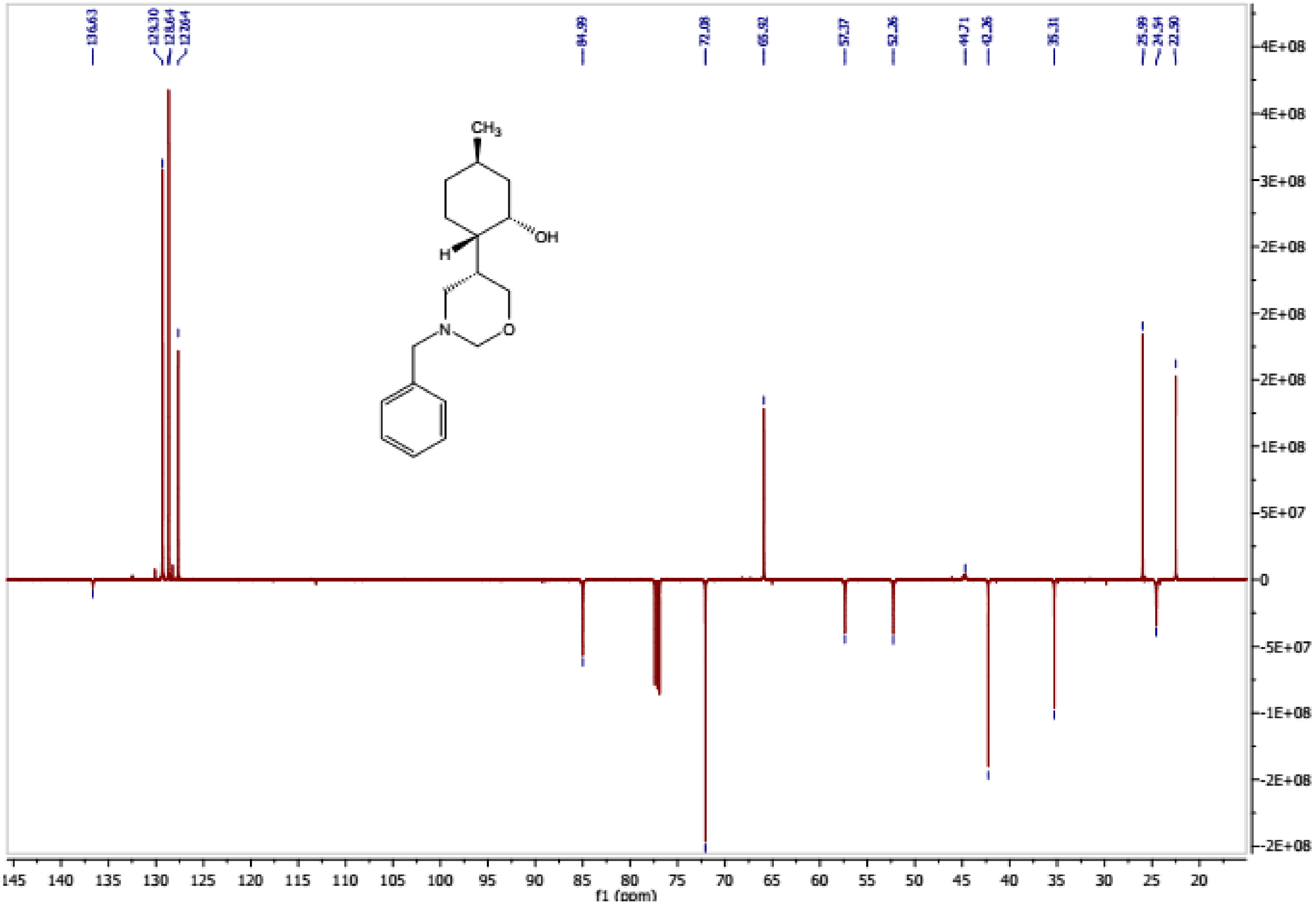

HSQC of compound **14**

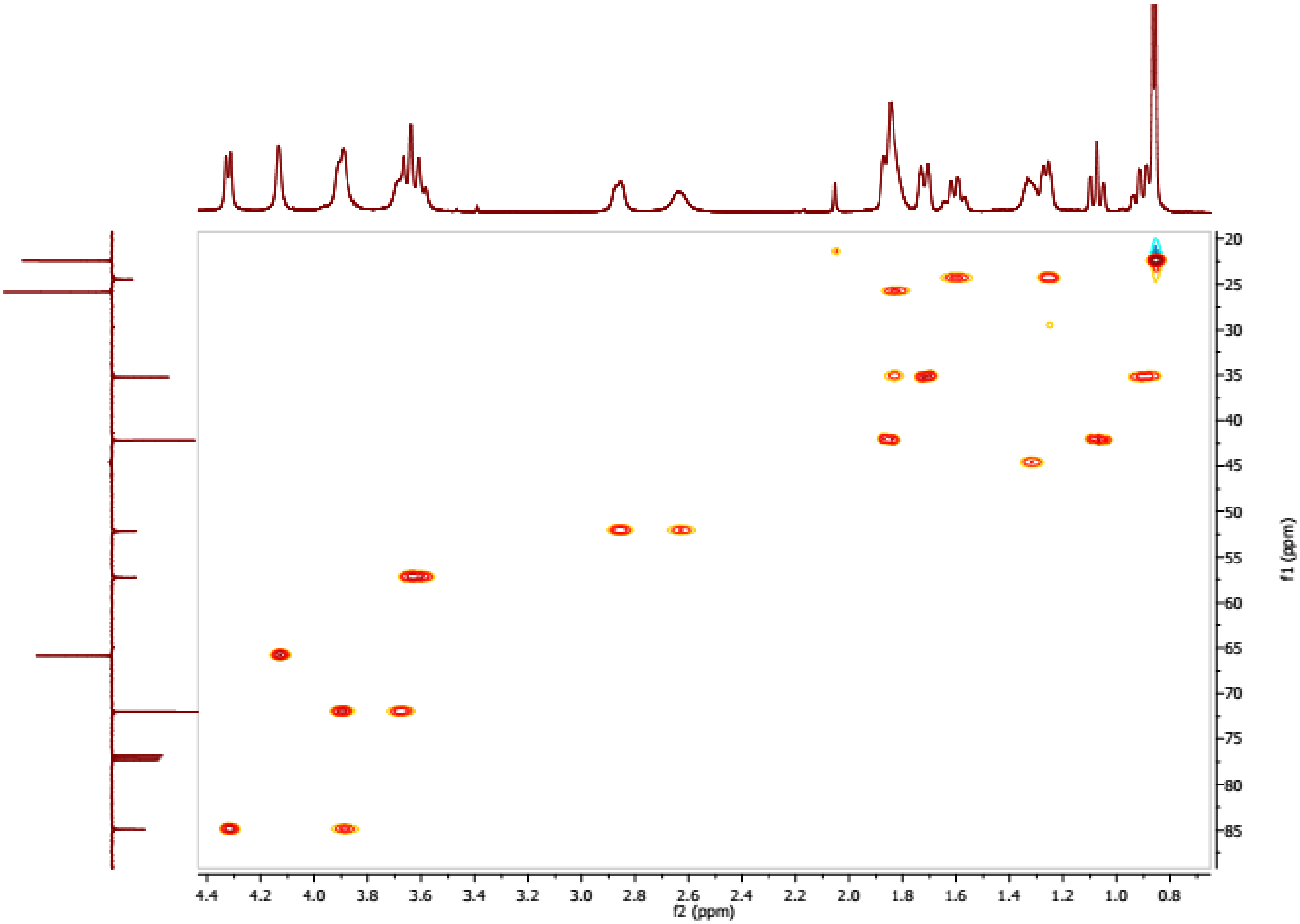

HMBC of compound **14**

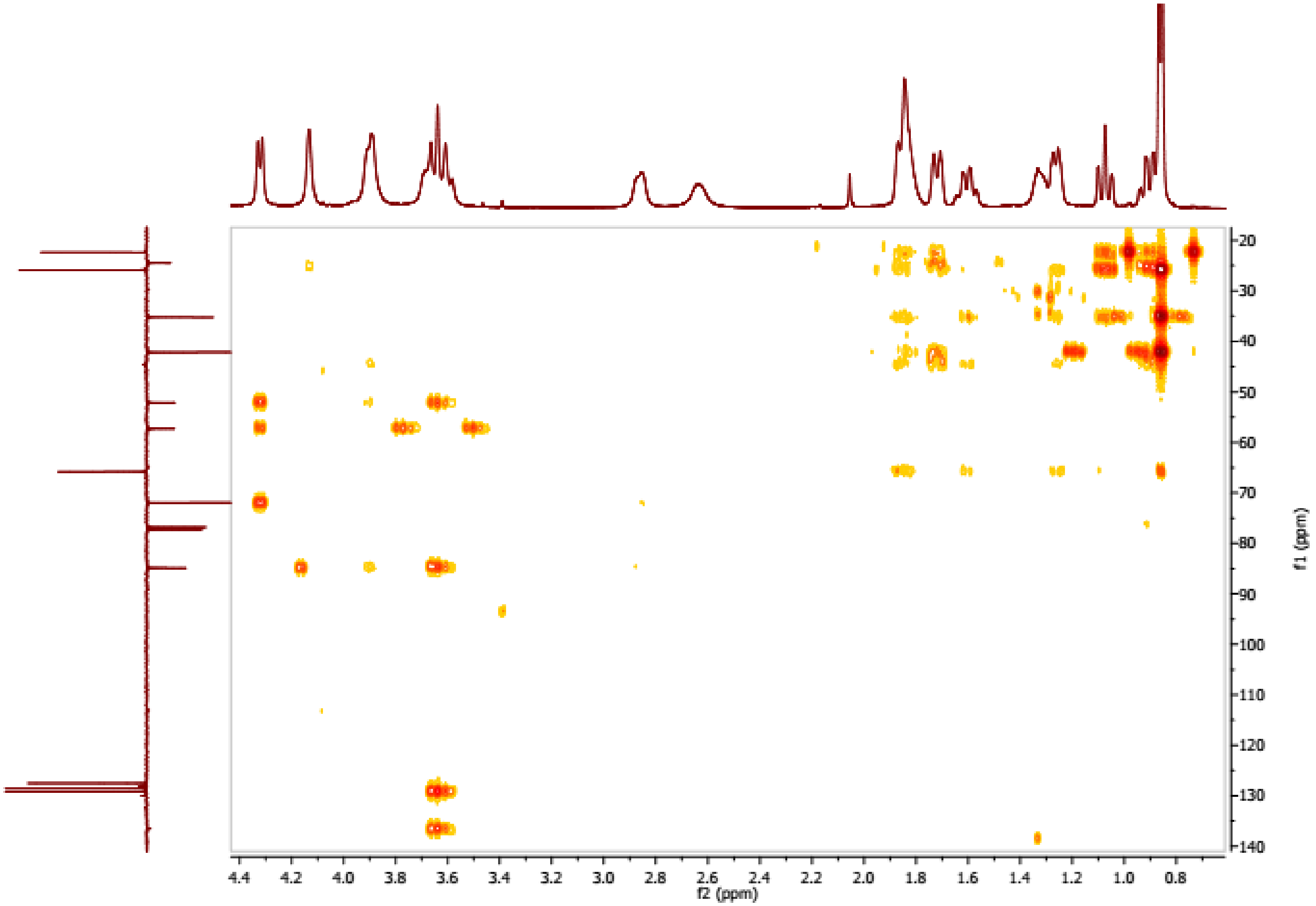

^{1}H-NMR of compound **15**

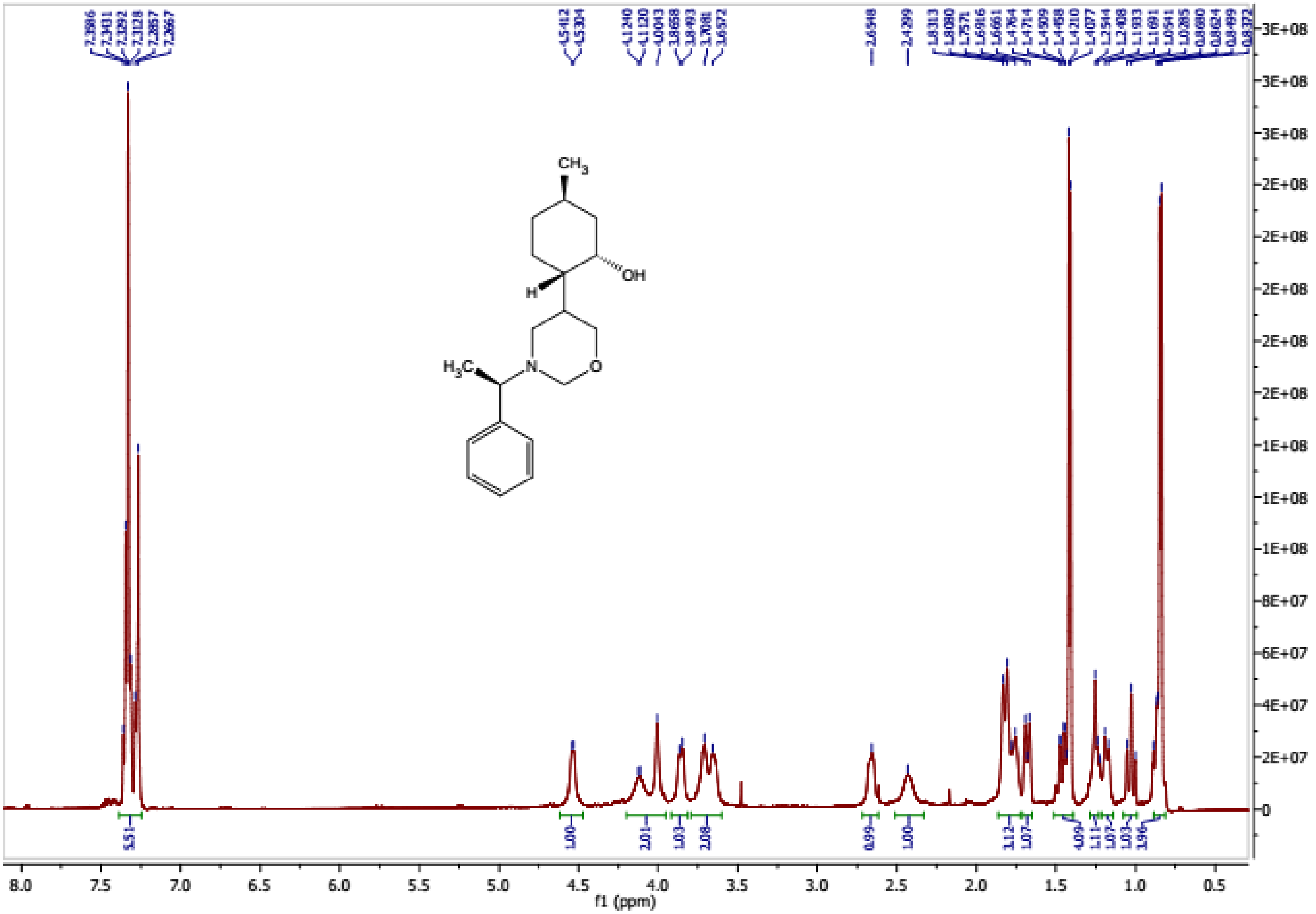

^{13}C-NMR of compound **15**

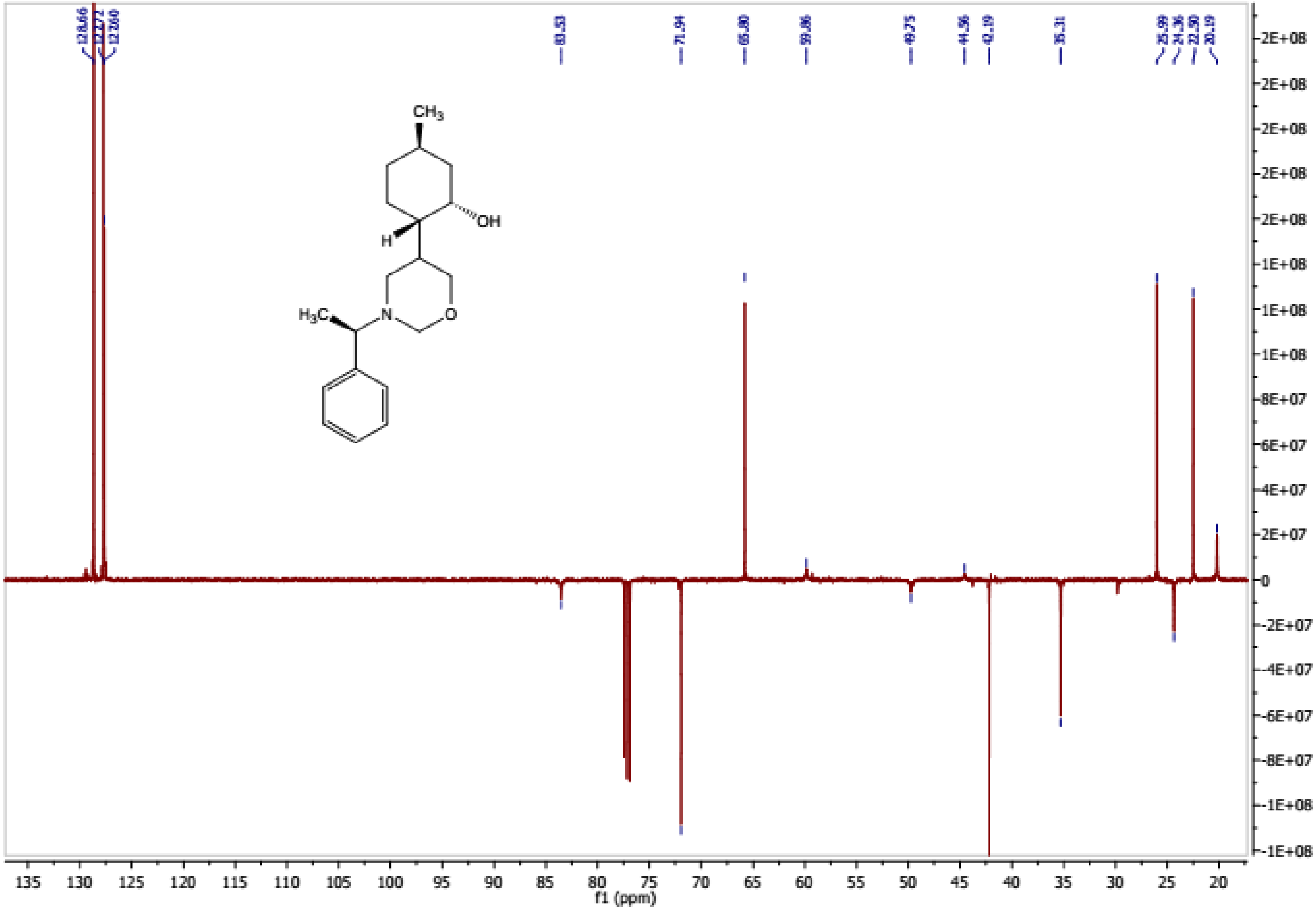

HSQC of compound **15**

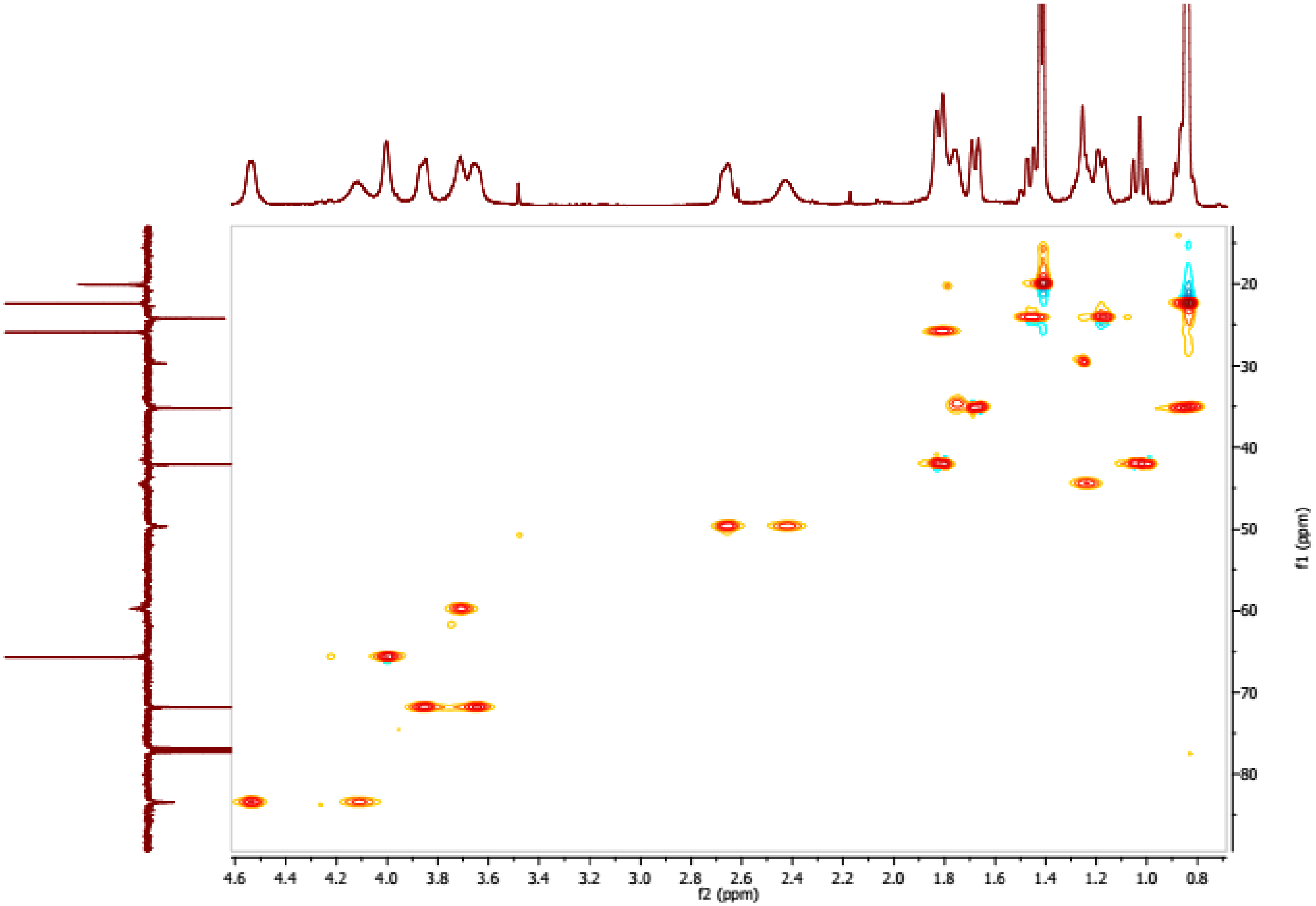

HMBC of compoun **15**

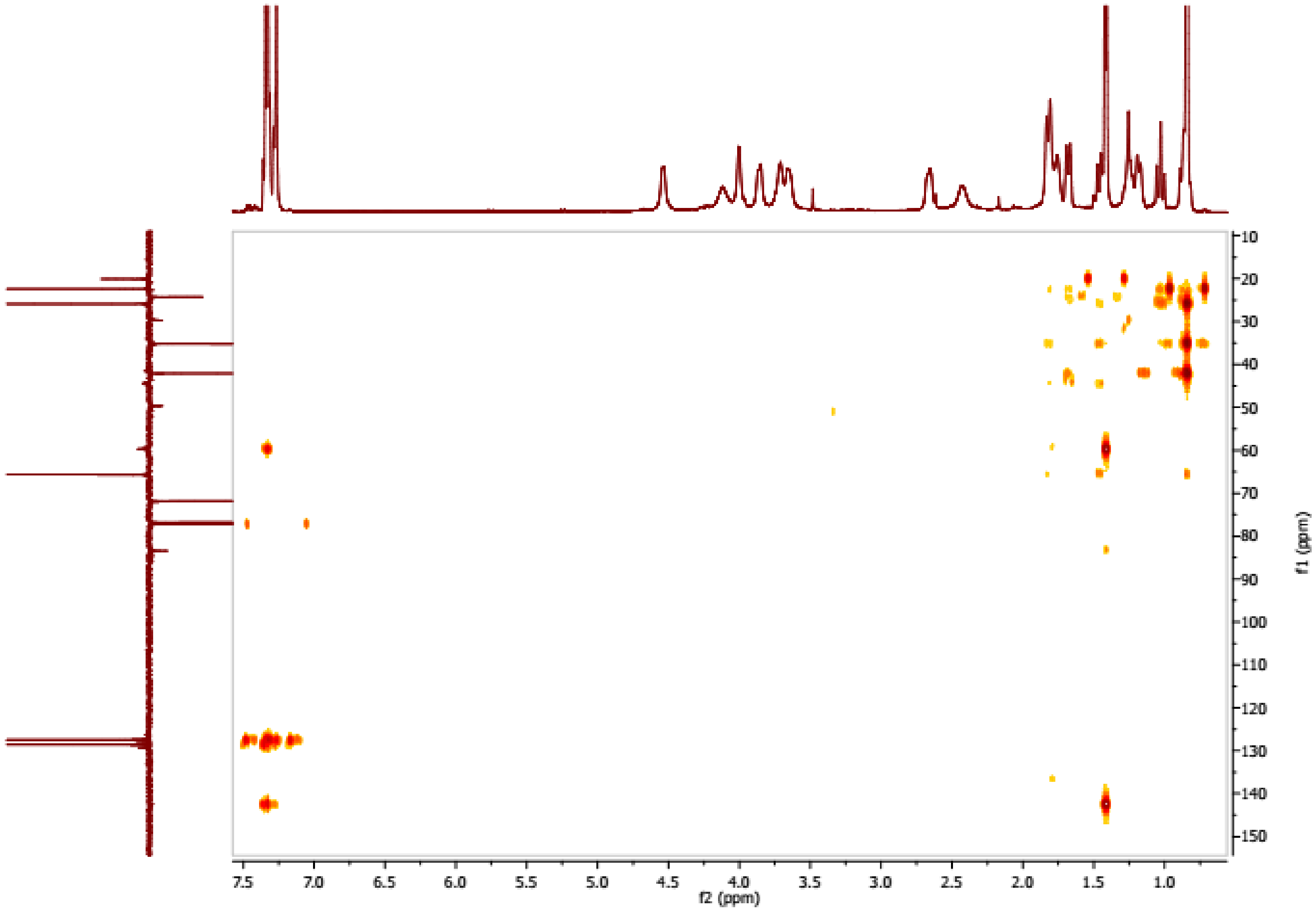

^{1}H-NMR of compound **16**

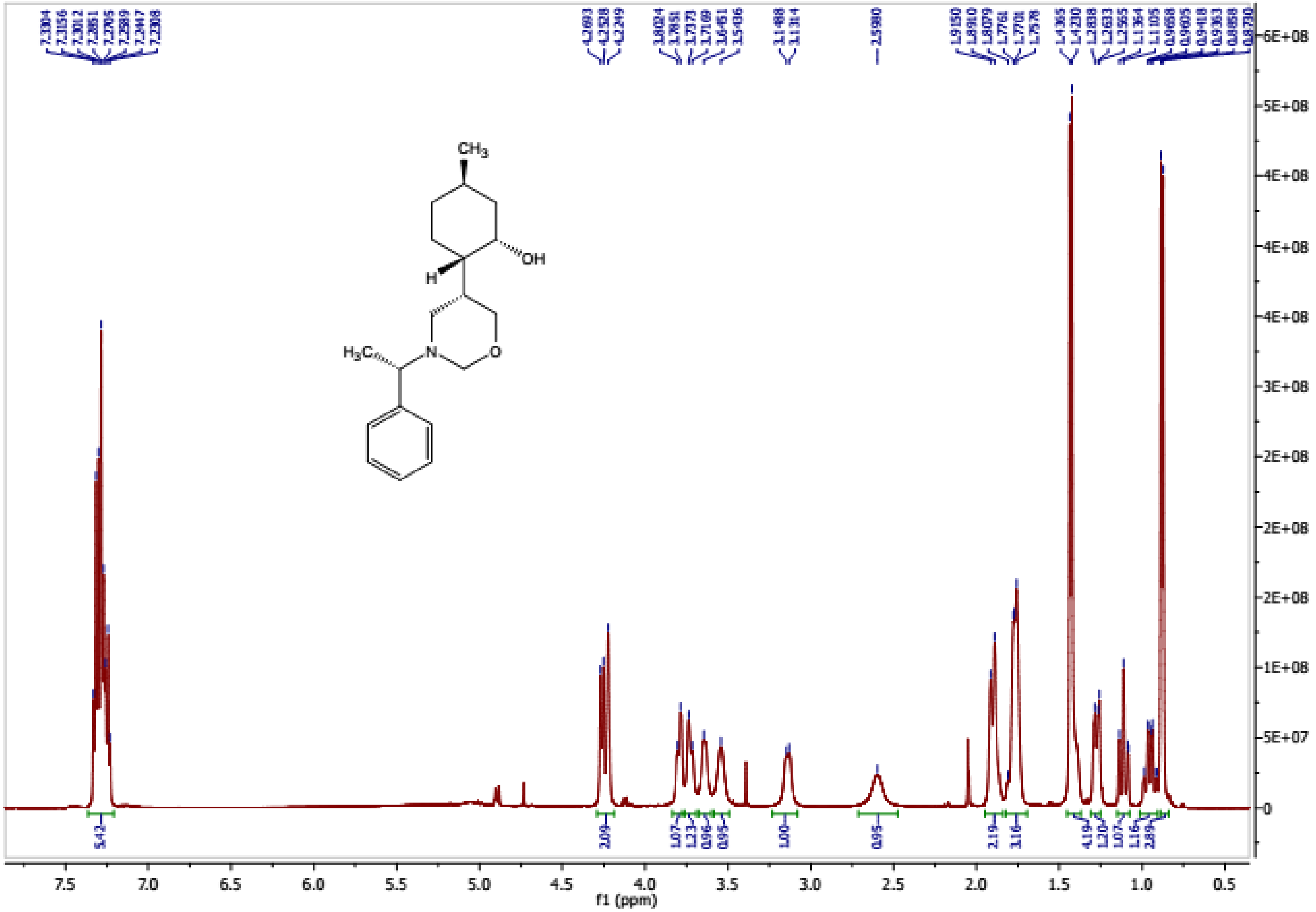

^{13}C-NMR of compound **16**

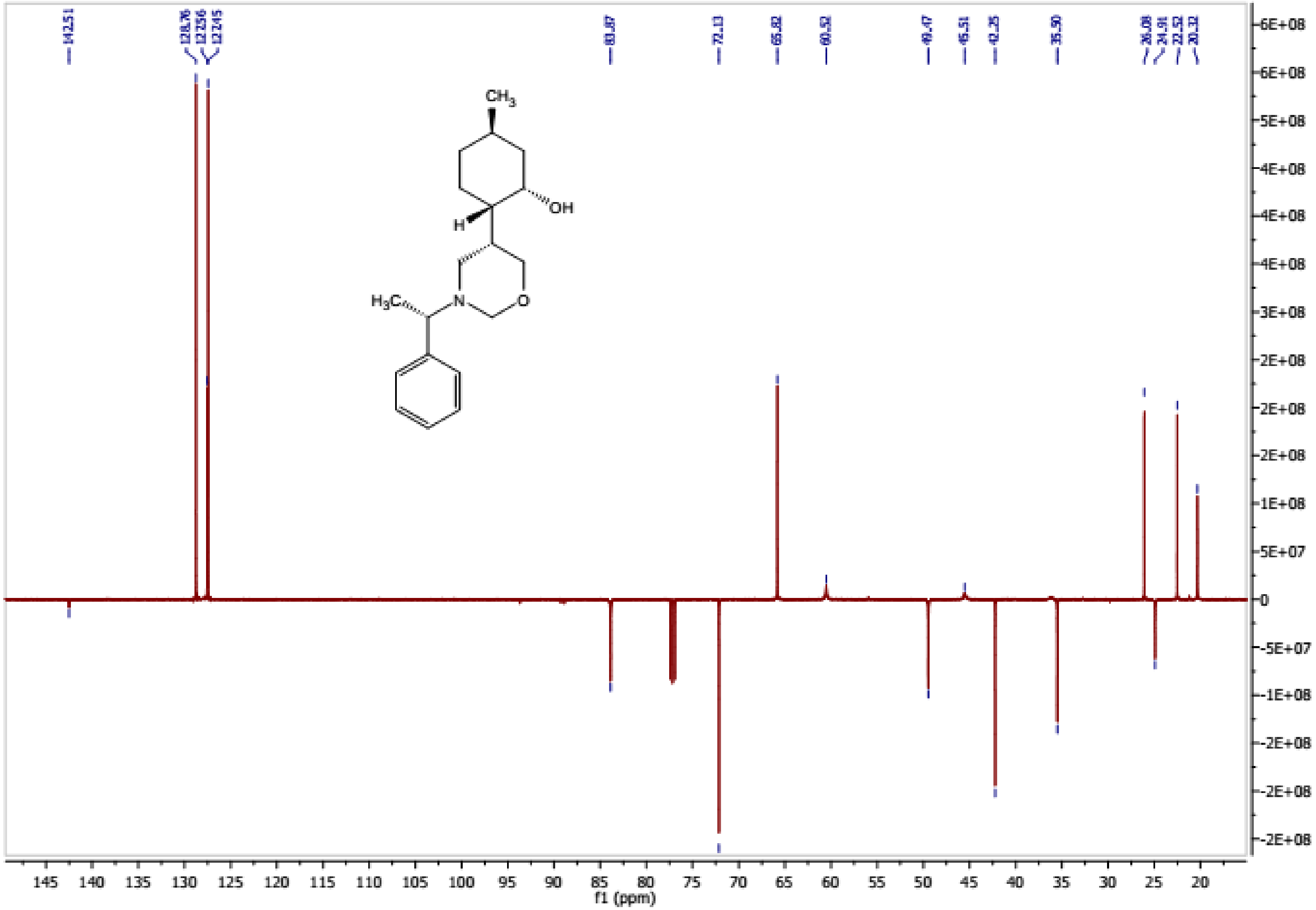

HSQC of coumpound **16**

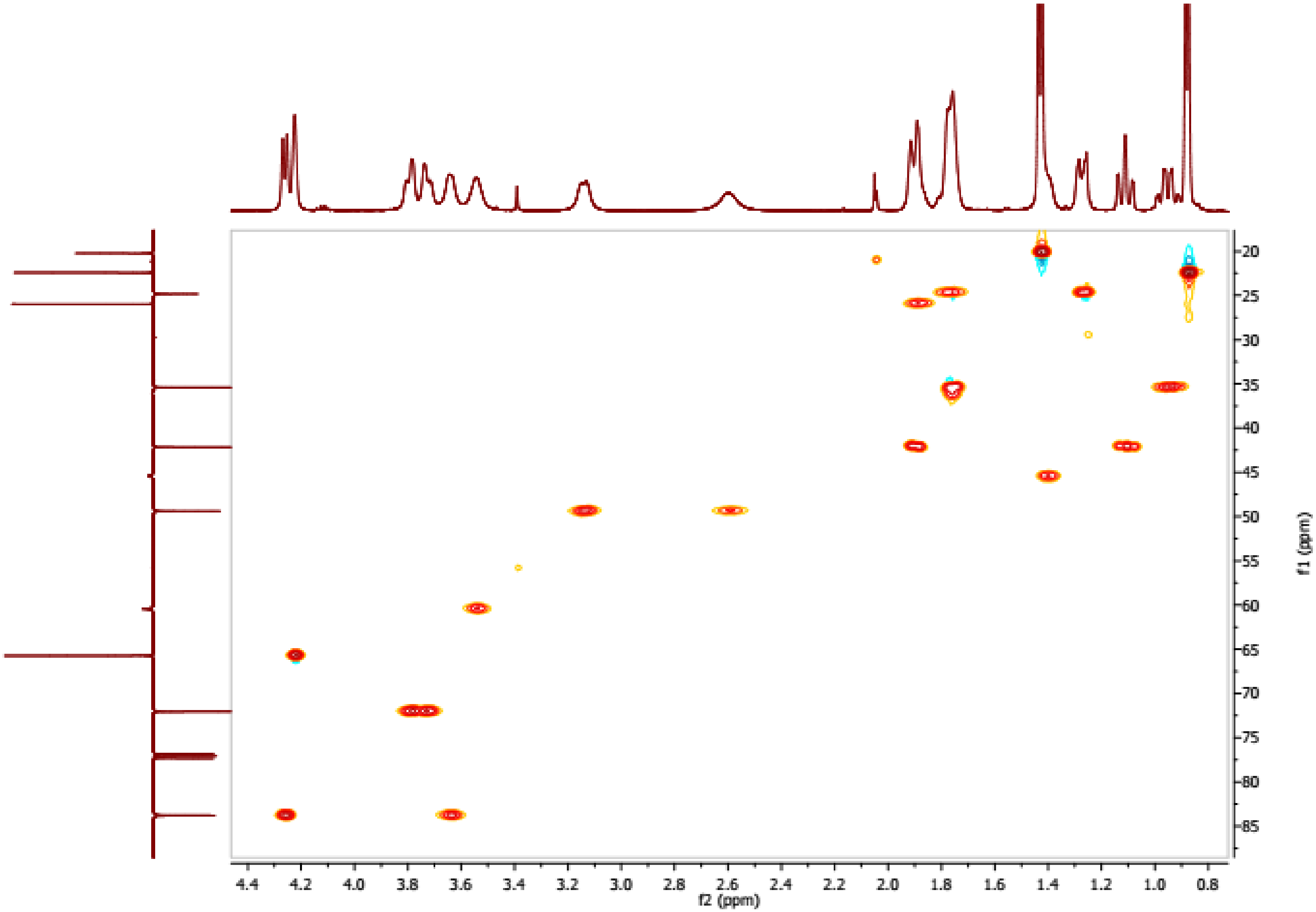

^{1}H-NMR of compound **17**

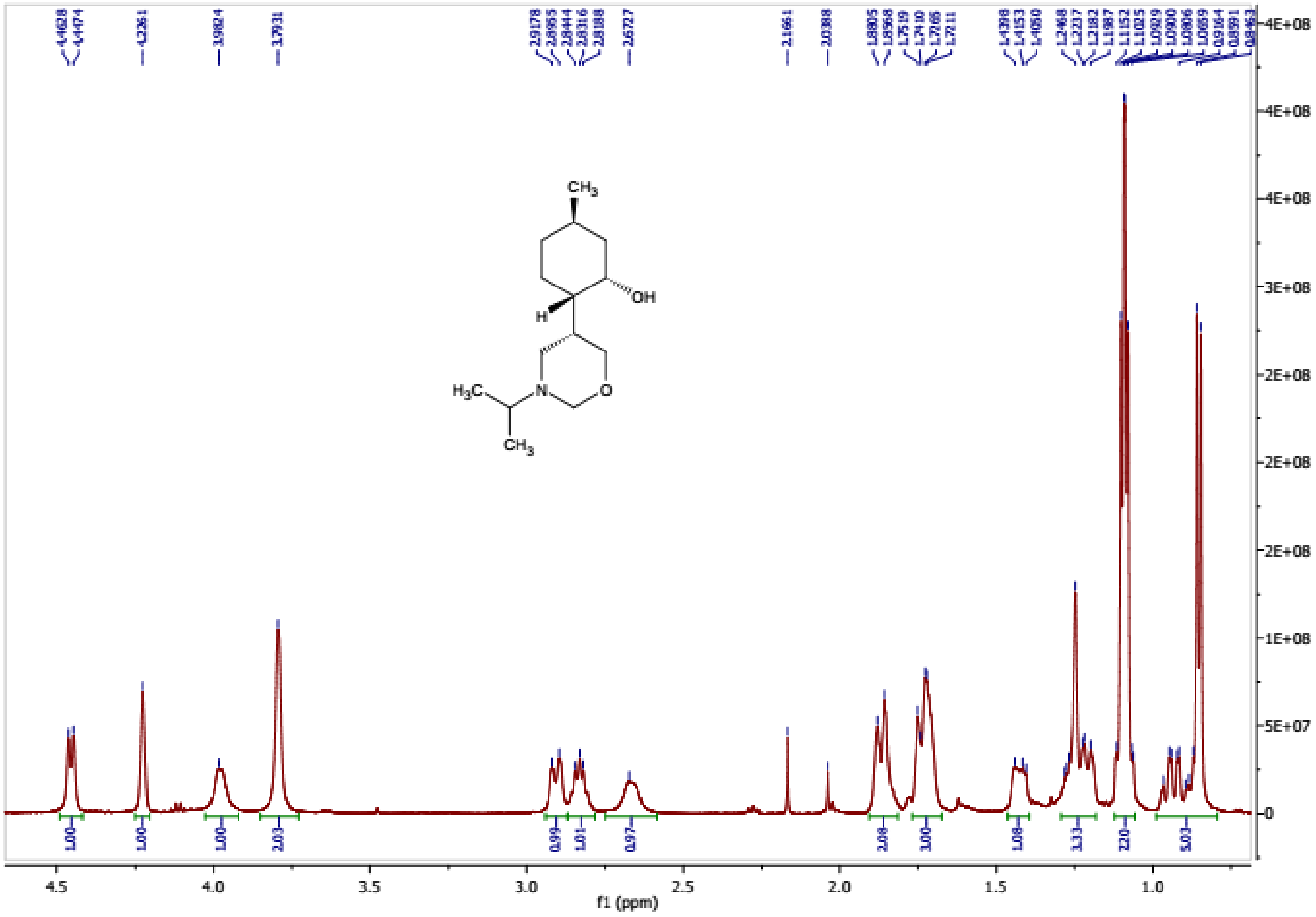

^{13}C-NMR of compound **17**

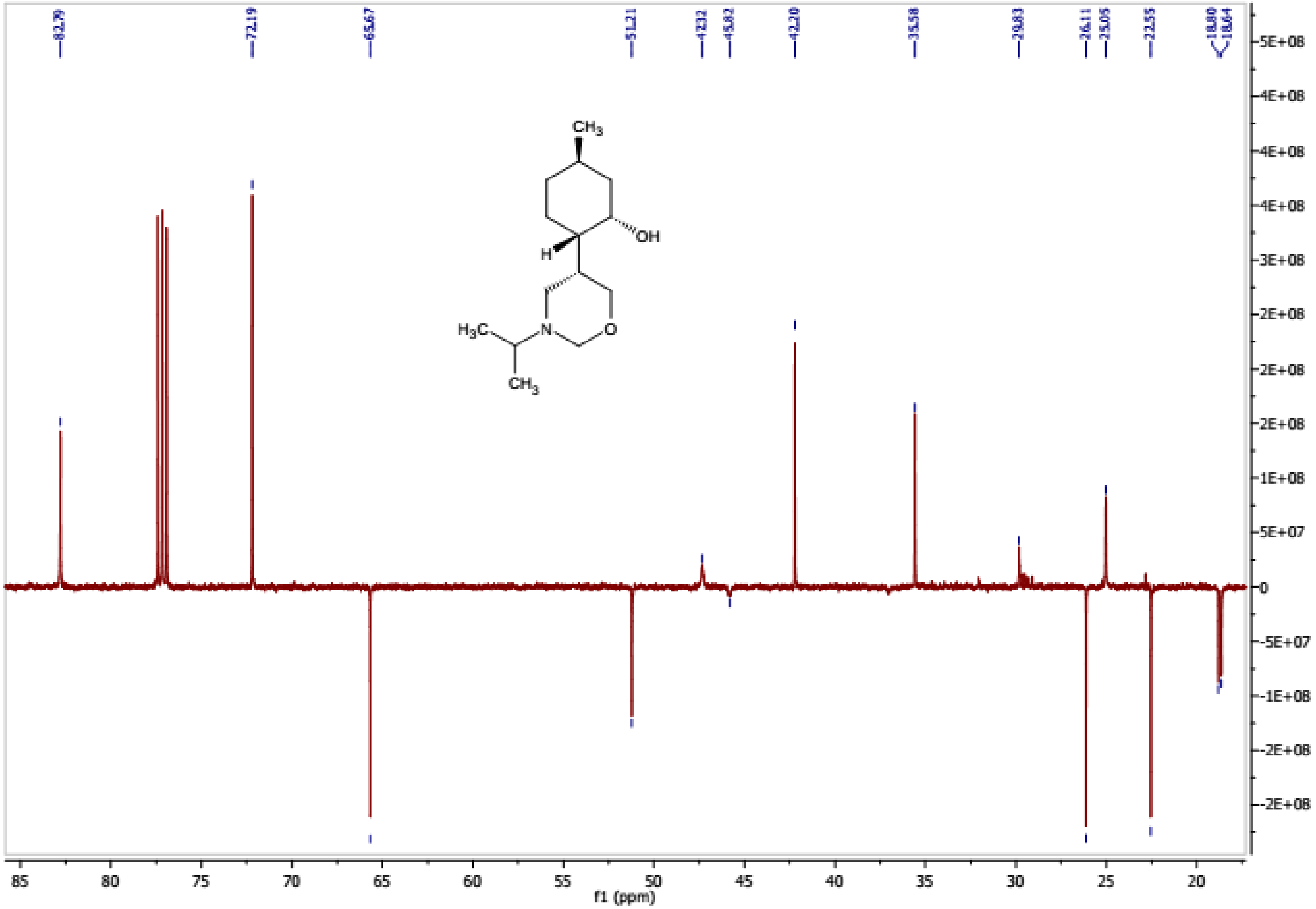

HSQC of compound **17**

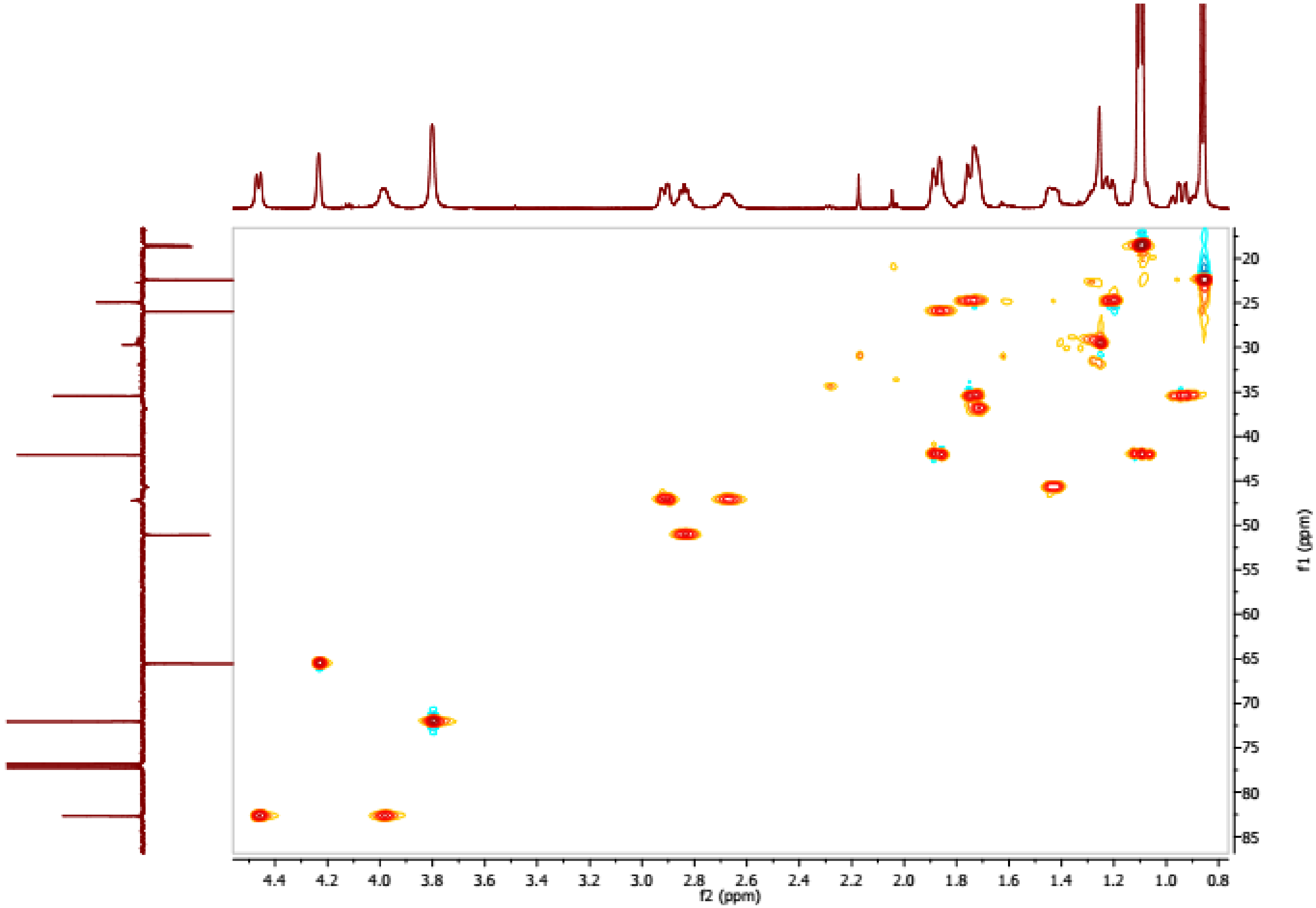

HMBC of compound **17**

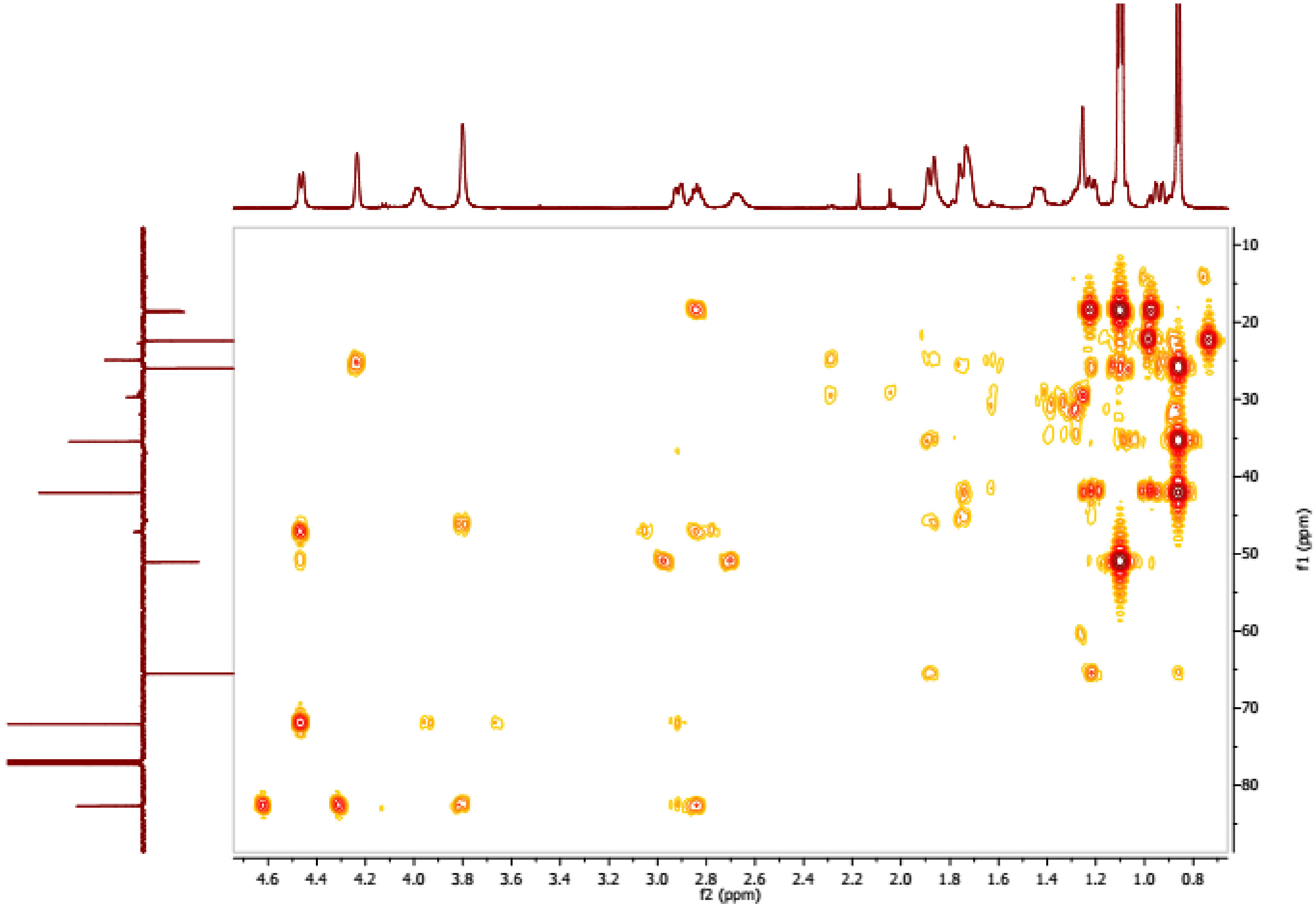

^{1}H-NMR of compound **18**

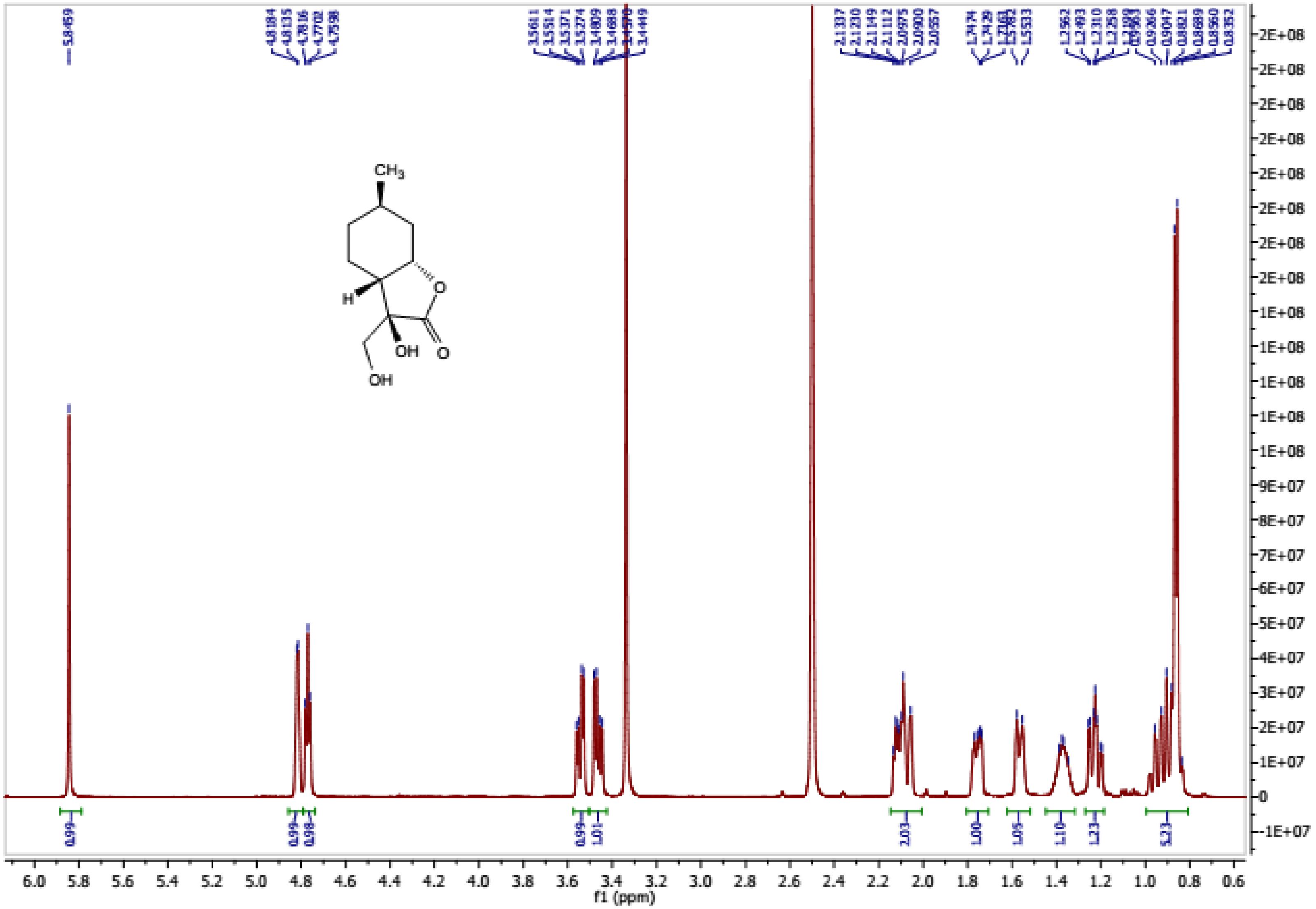

^{13}C-NMR of compound **18**

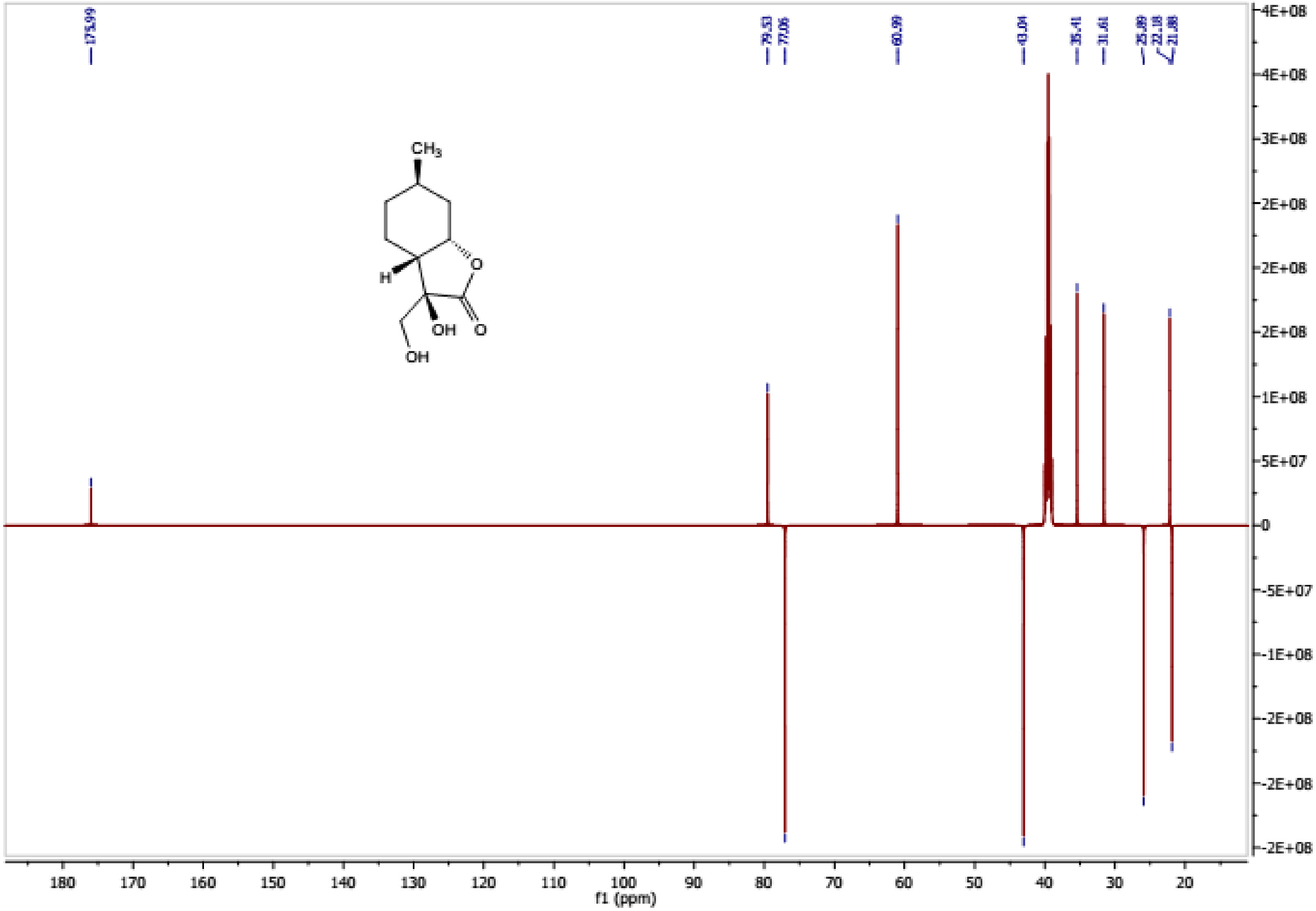

COSY of counpound **18**

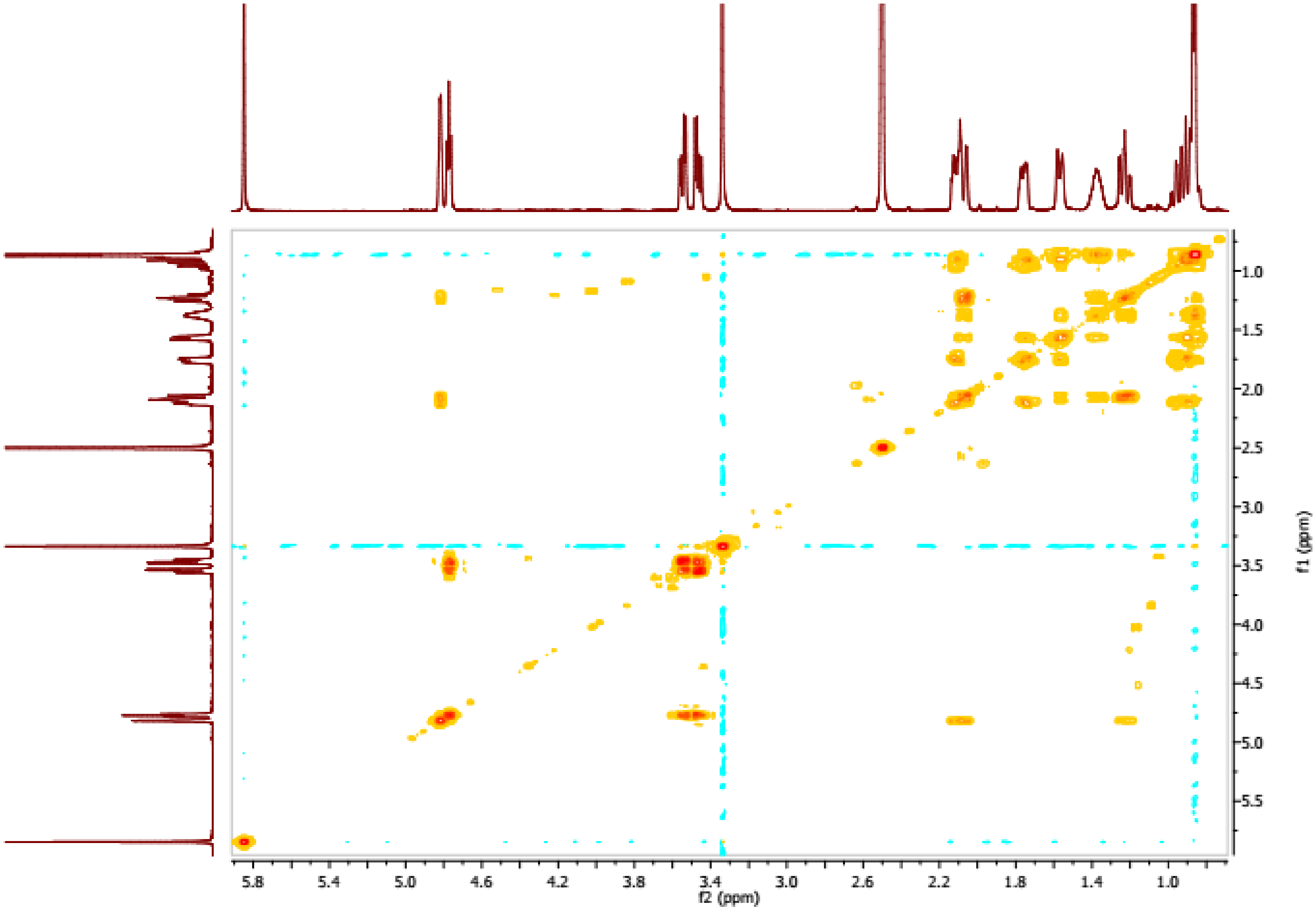

NOESY of compound **18**

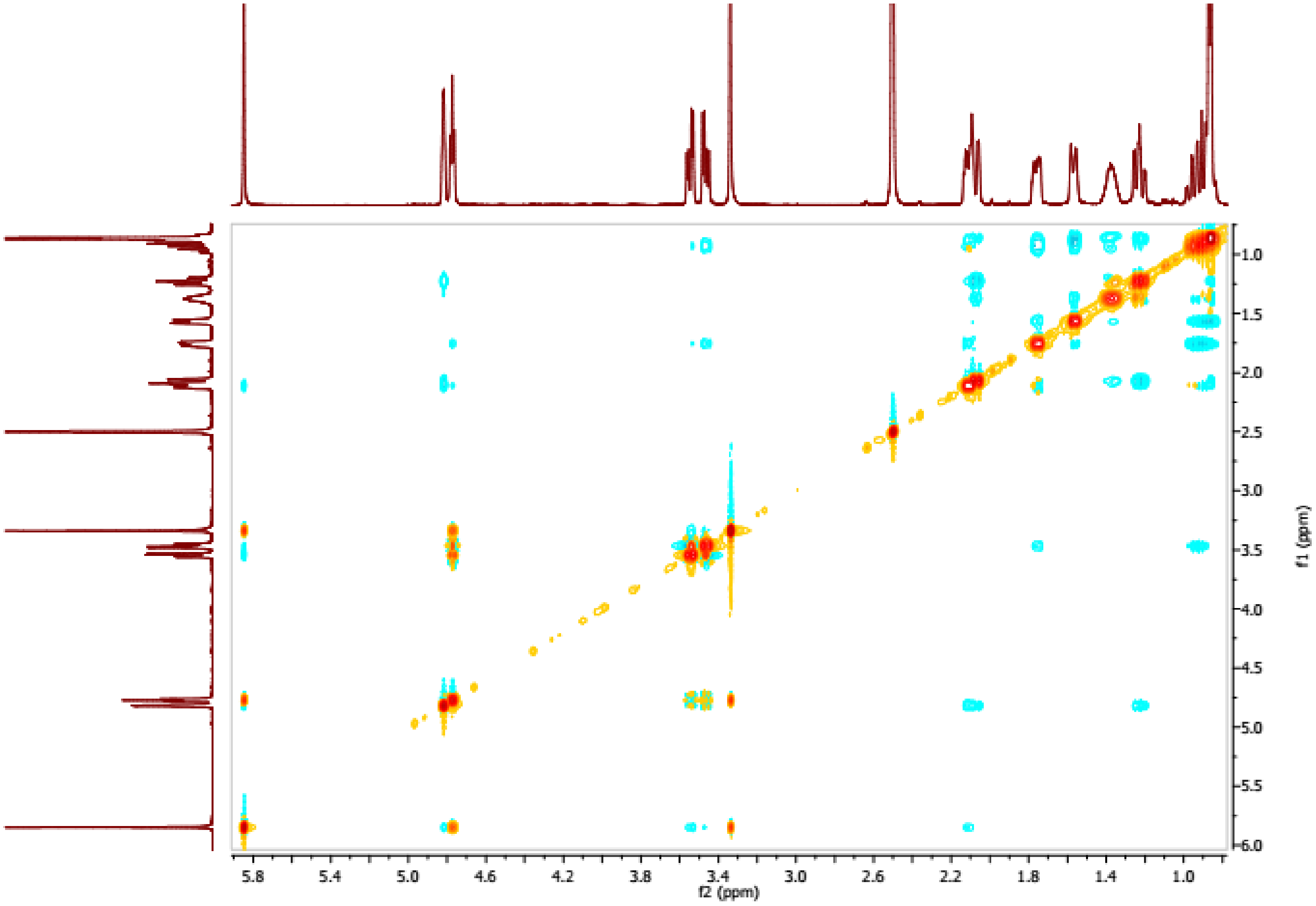

HSQC of compound **18**

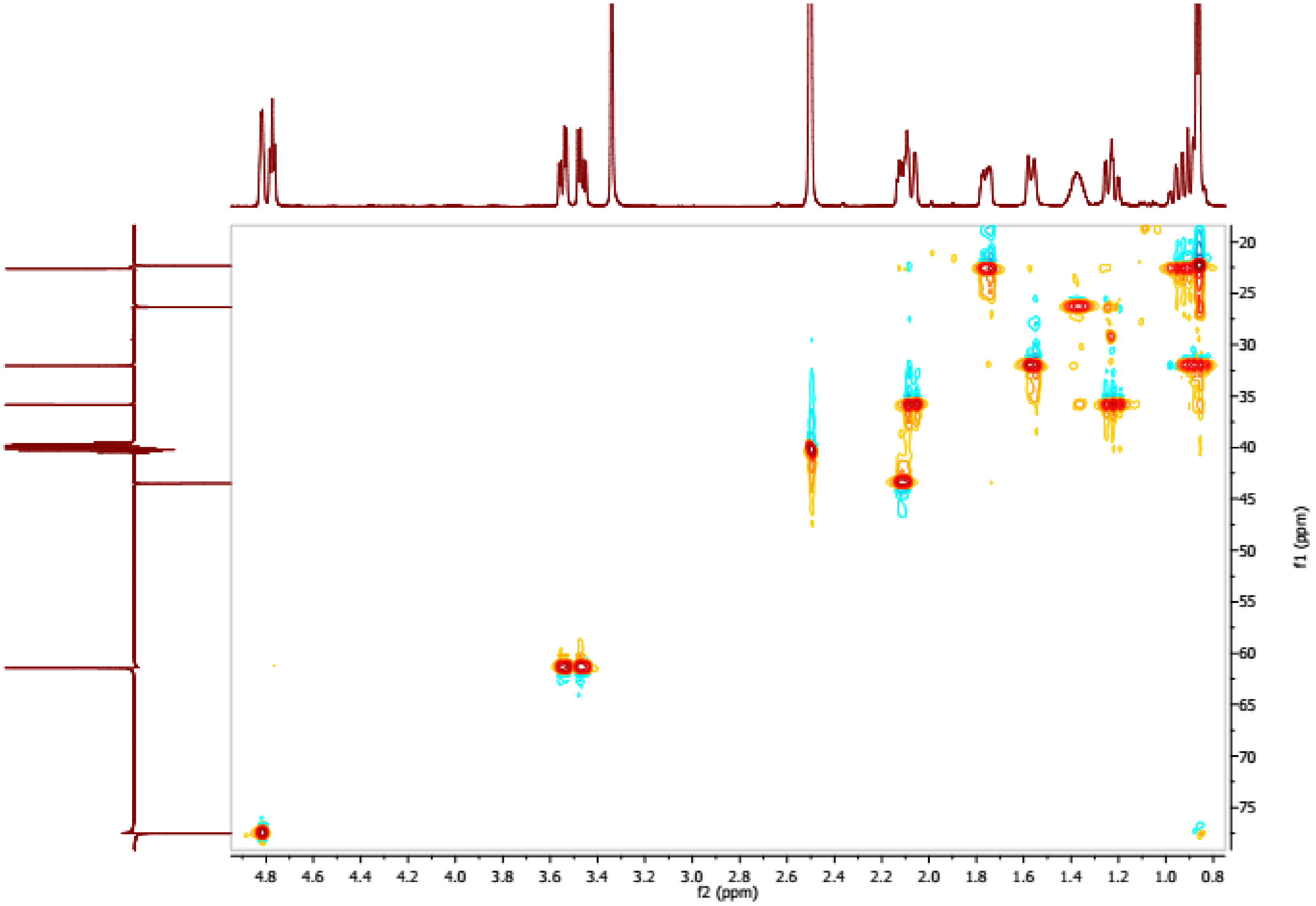

HMBC of compound **18**

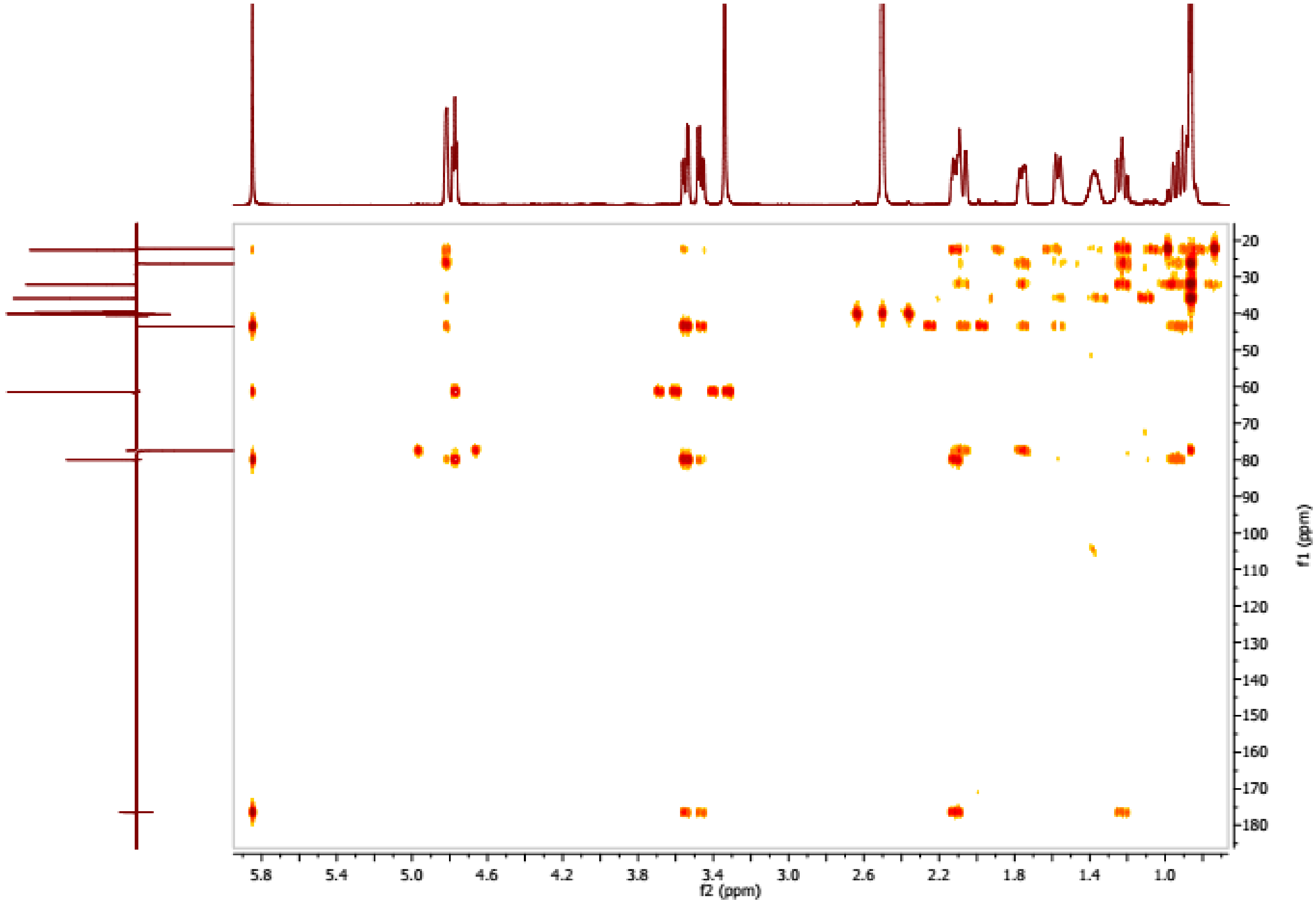

^{1}H-NMR of compound **19**

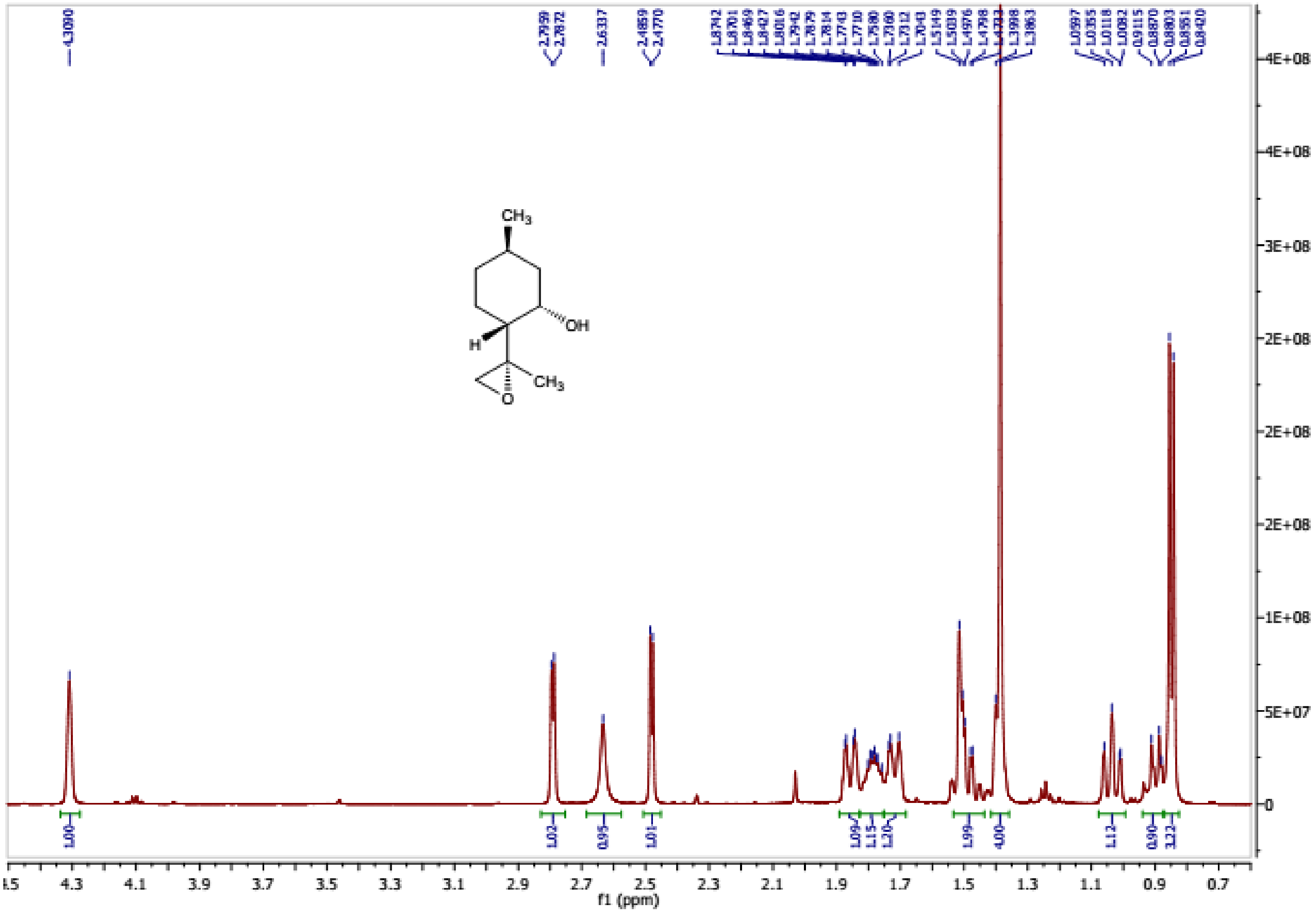

^{13}C-NMR of compound **19**

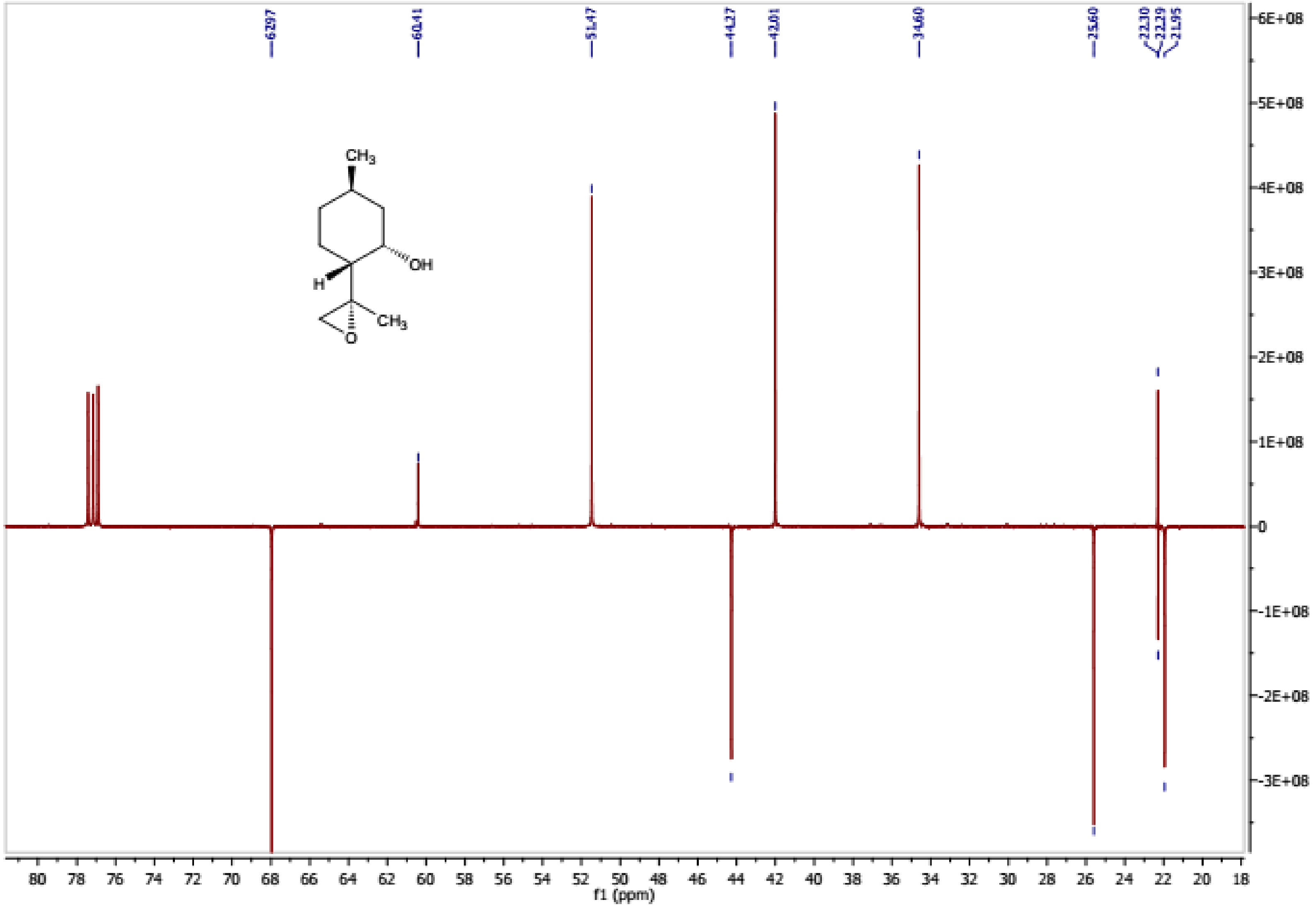

COSY of compound **20**

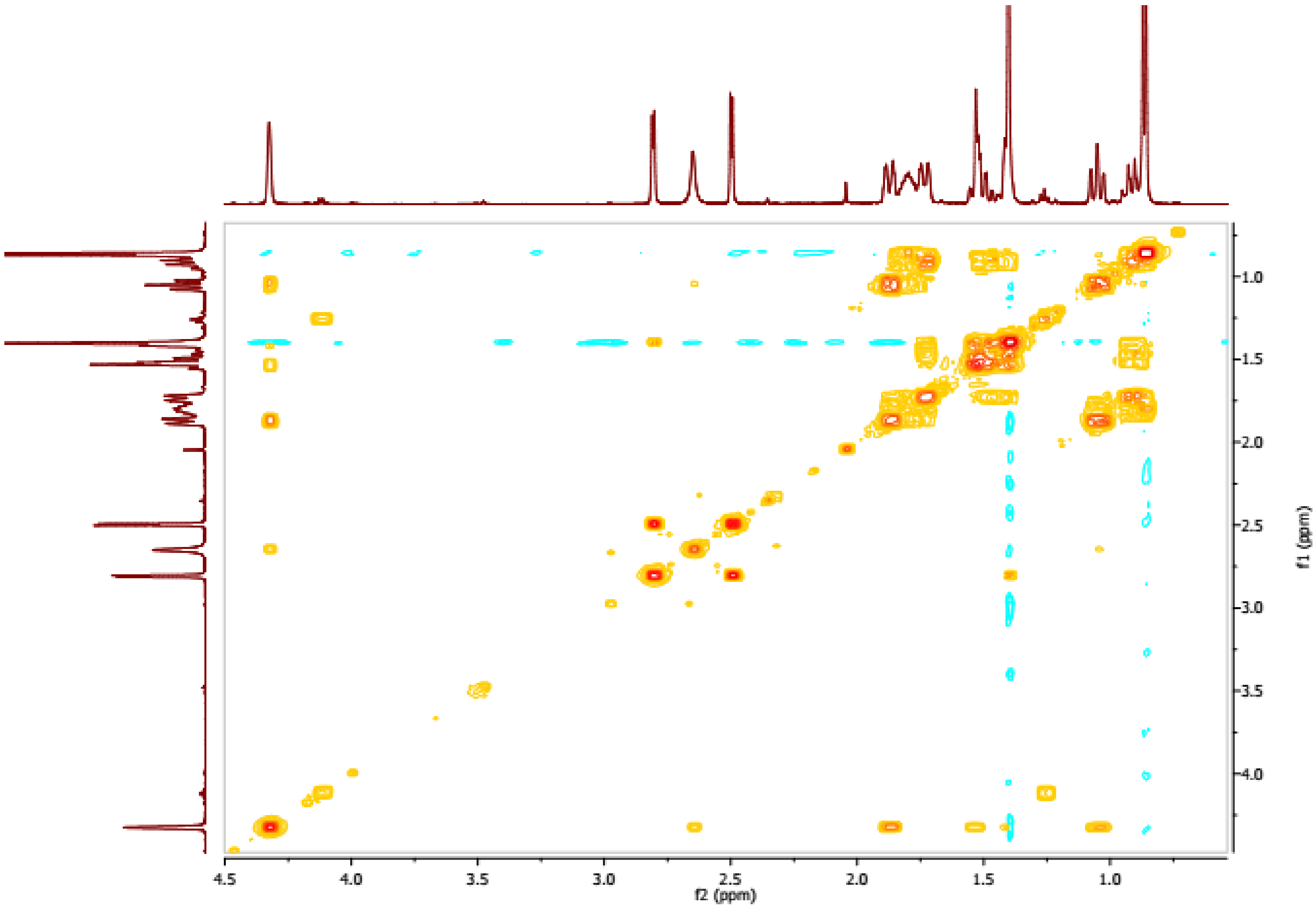

NOESY of compound **19**

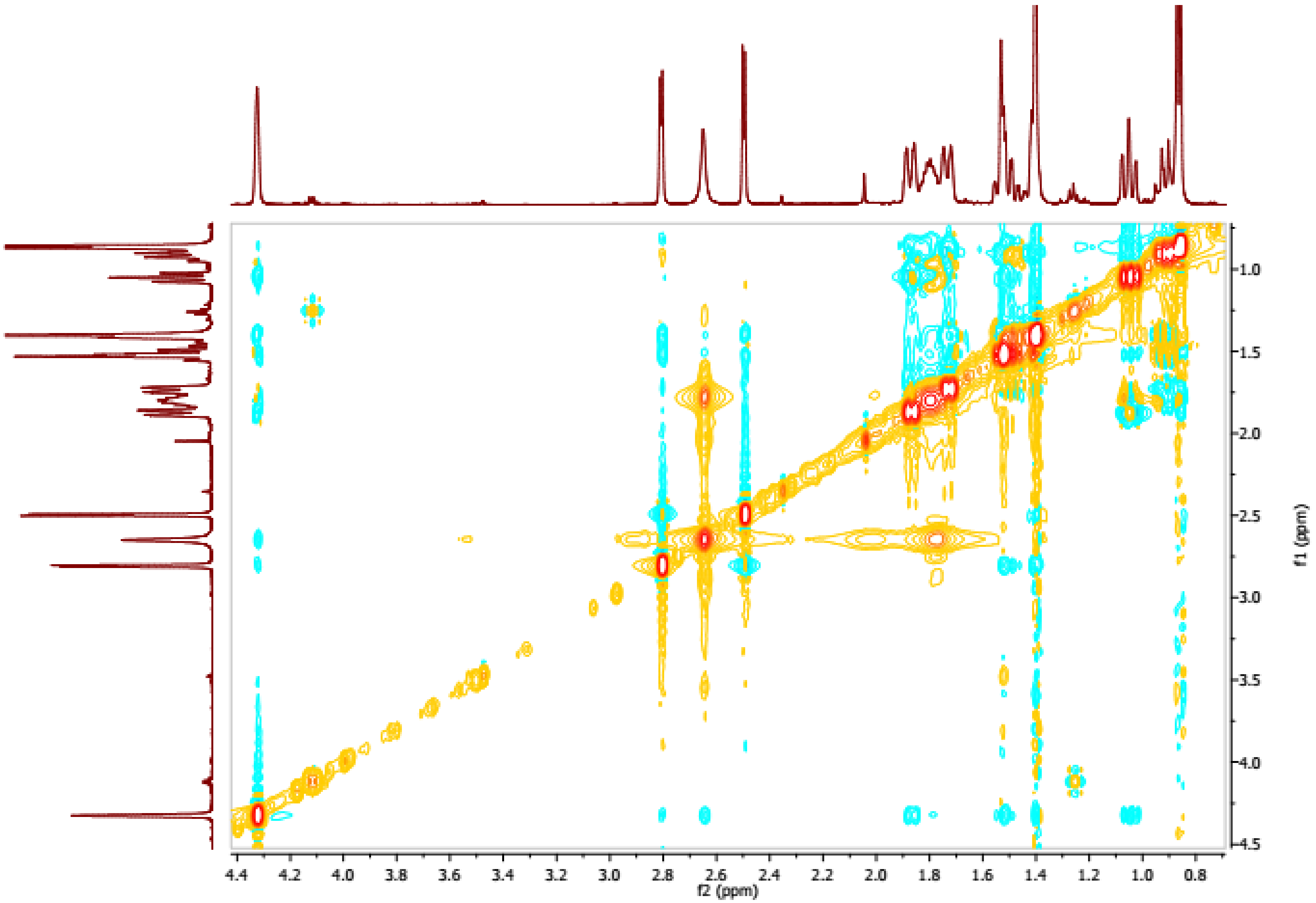

HSQC of compound **19**

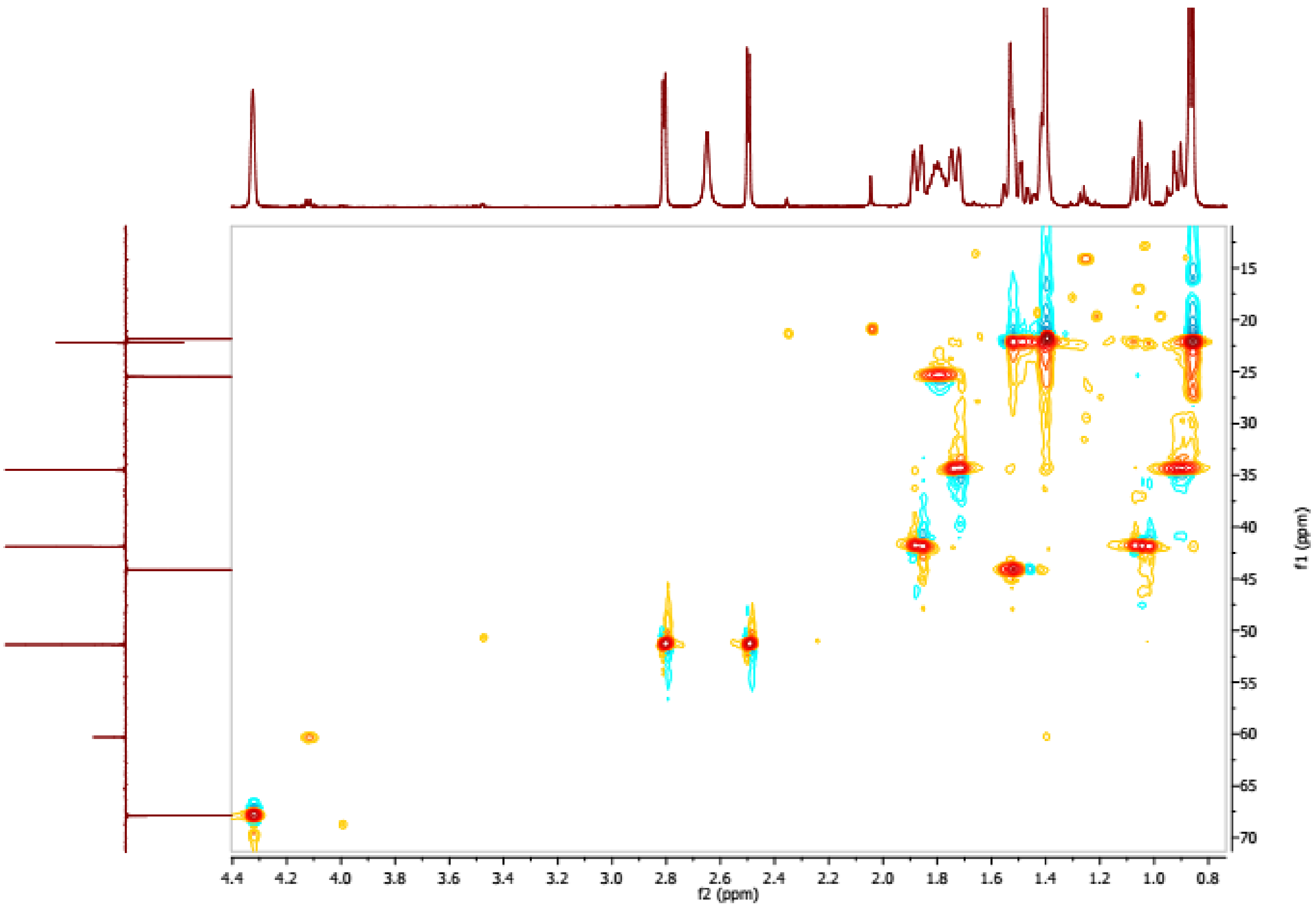

HMBC of compound **19**

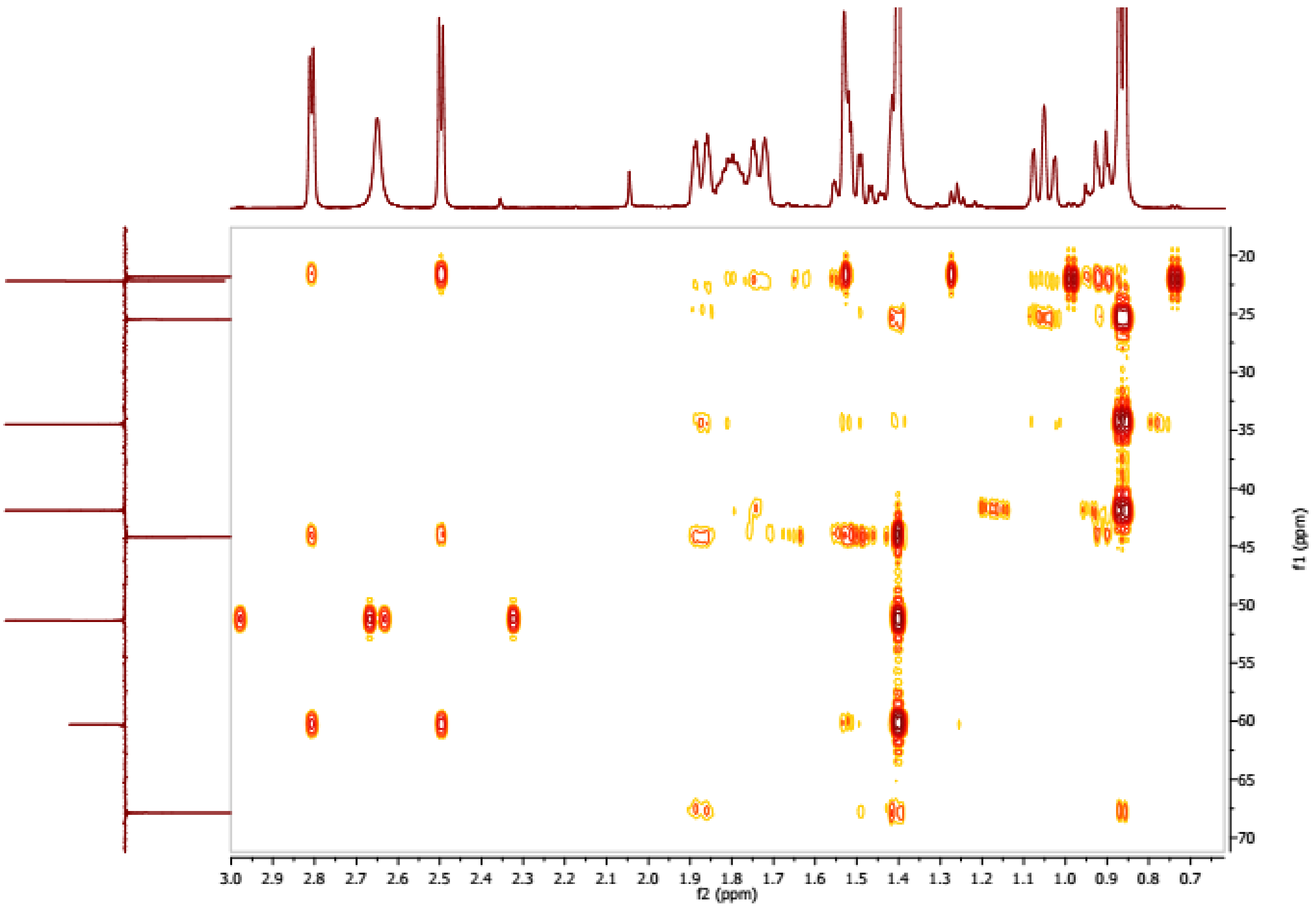

^{1}H-NMR of compound **20**

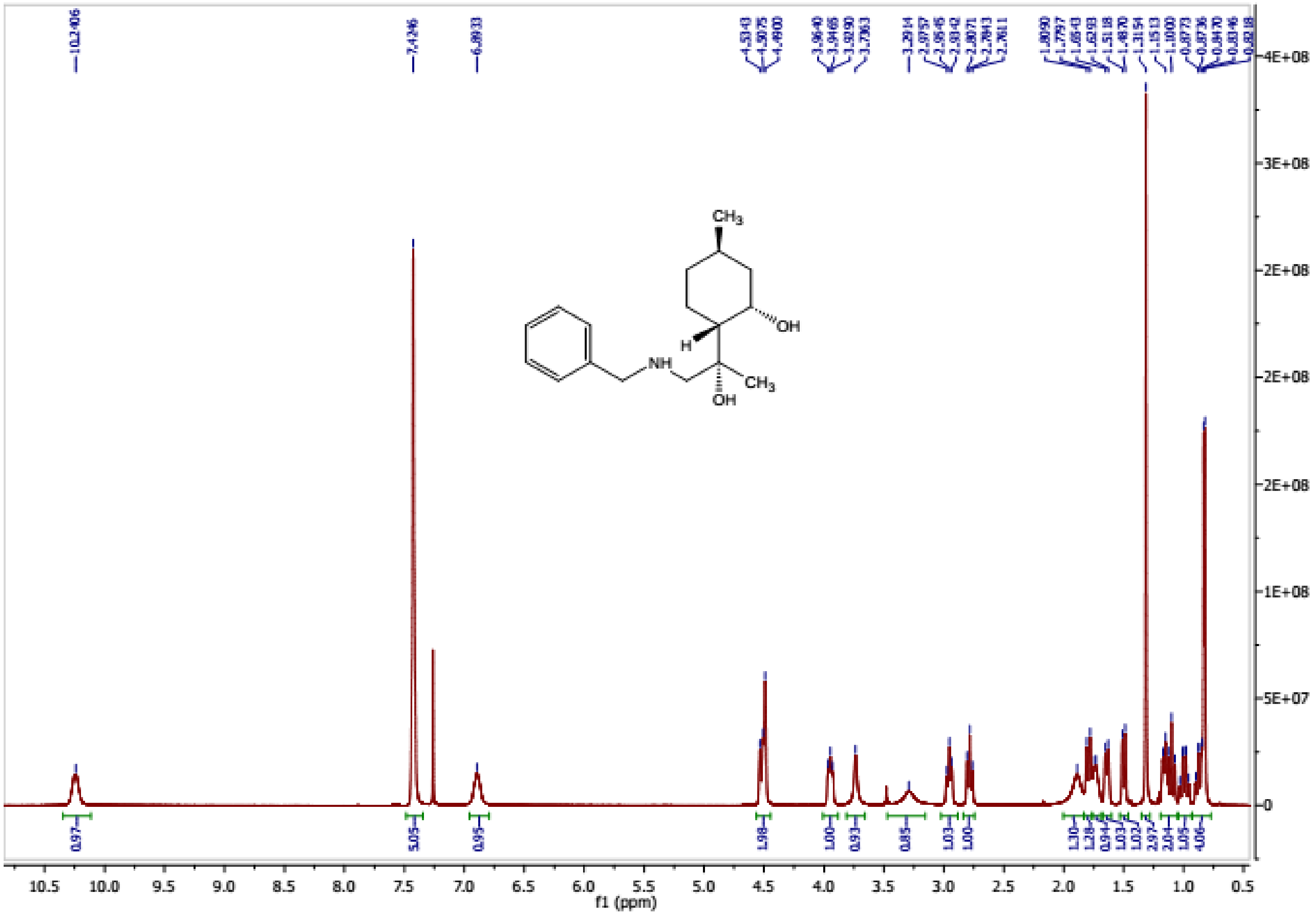

^{13}C-NMR of compound **20**

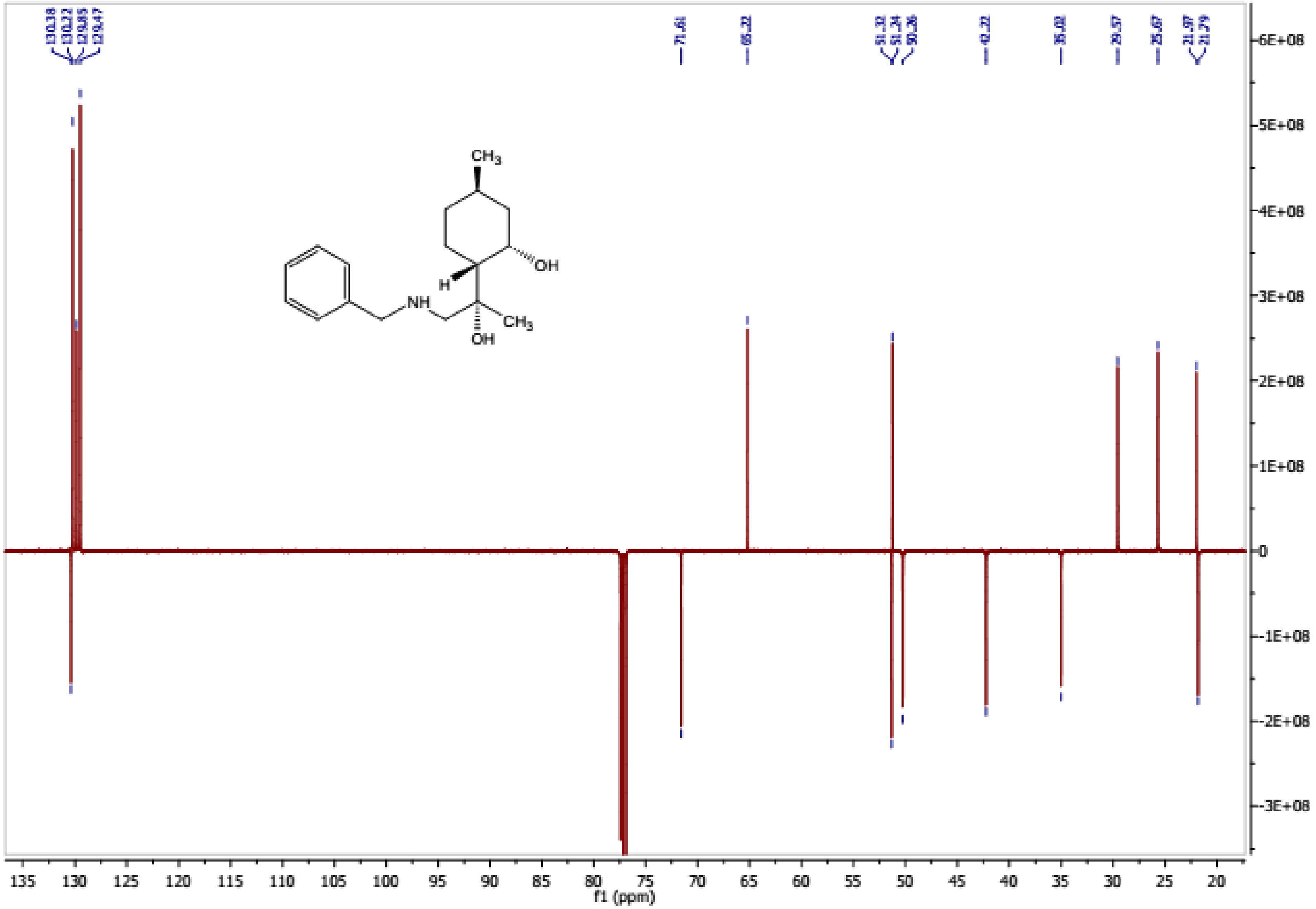

COSY of compound **20**

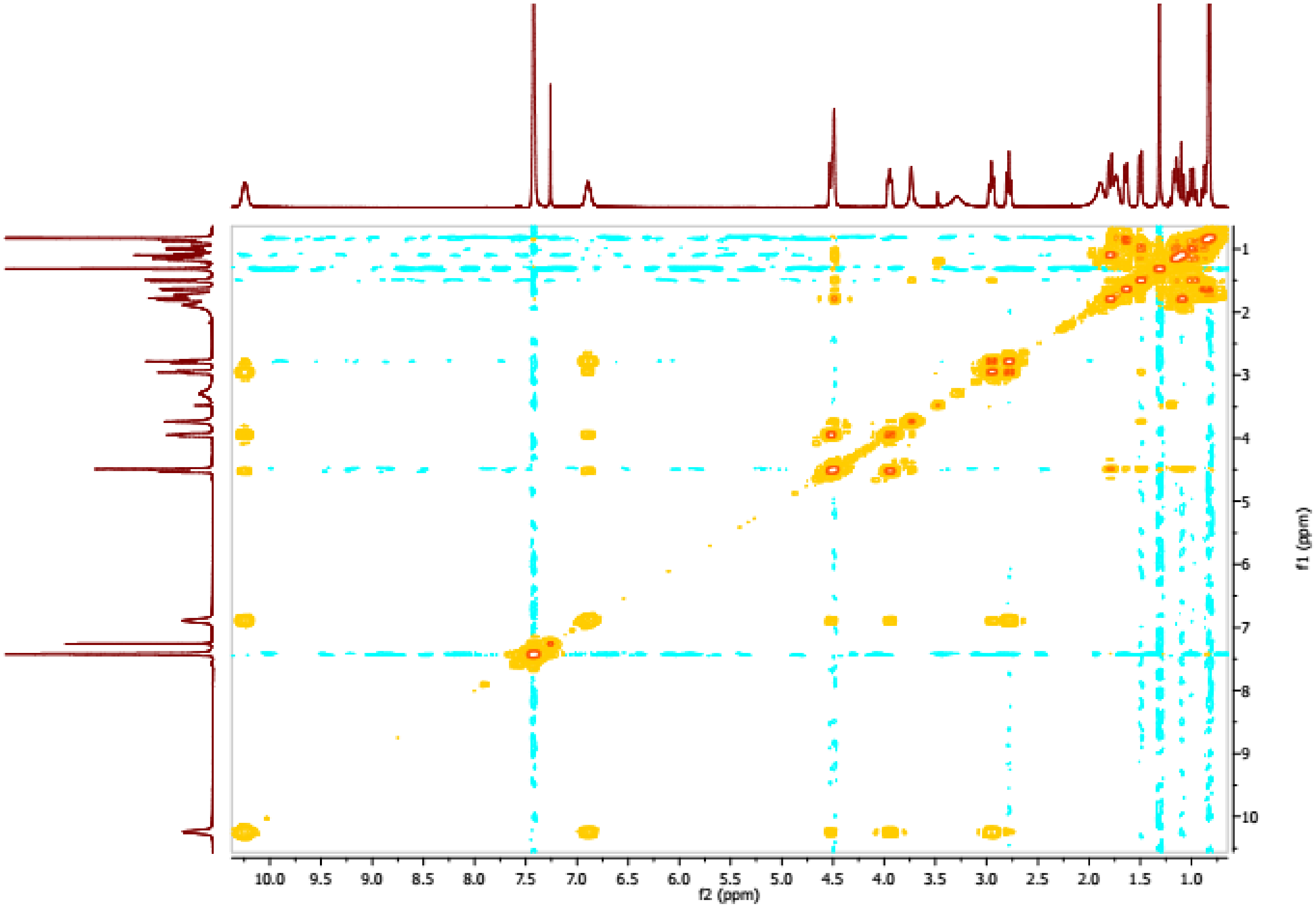

NOESY of compound **20**

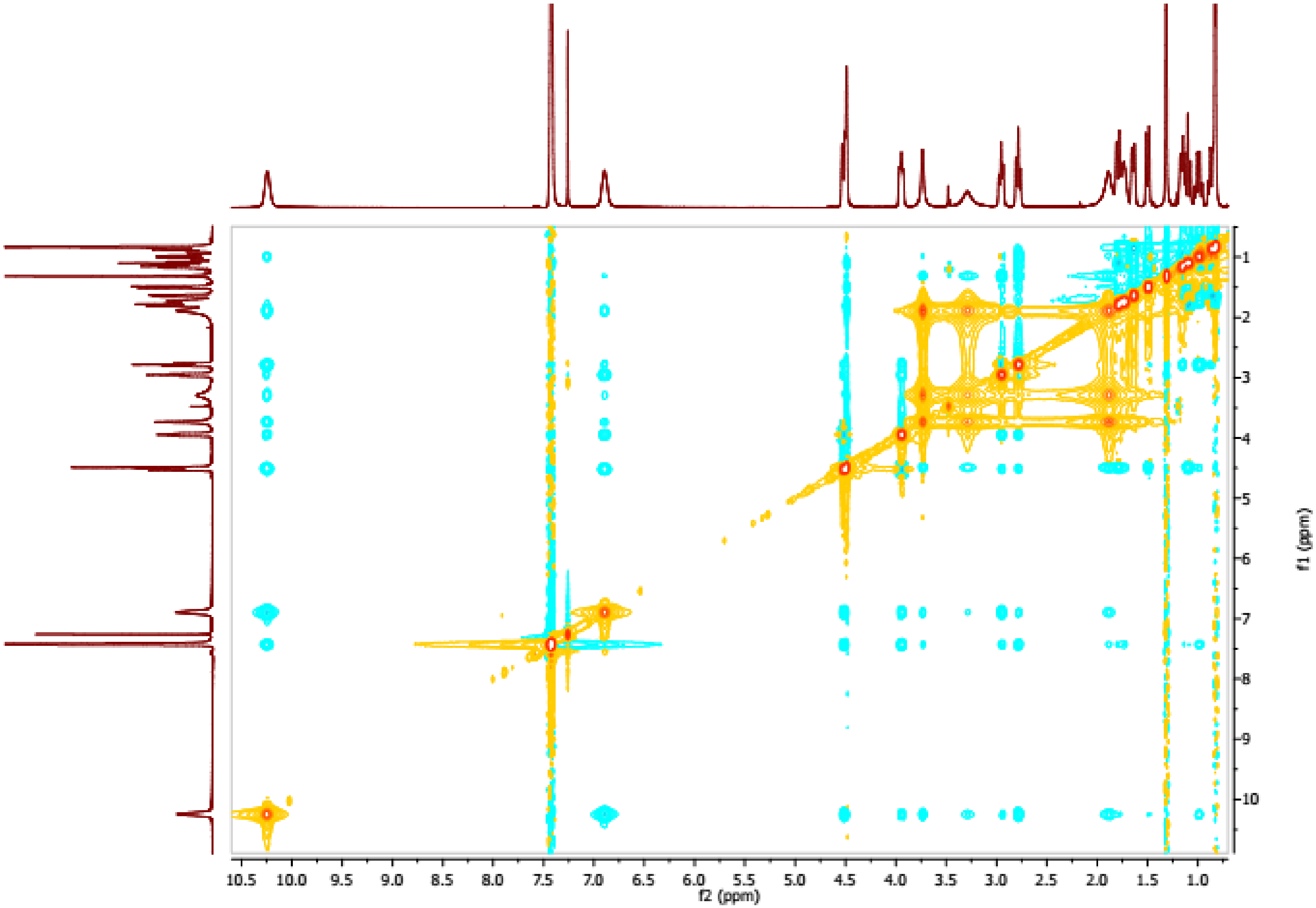

HSQC of compound **20**

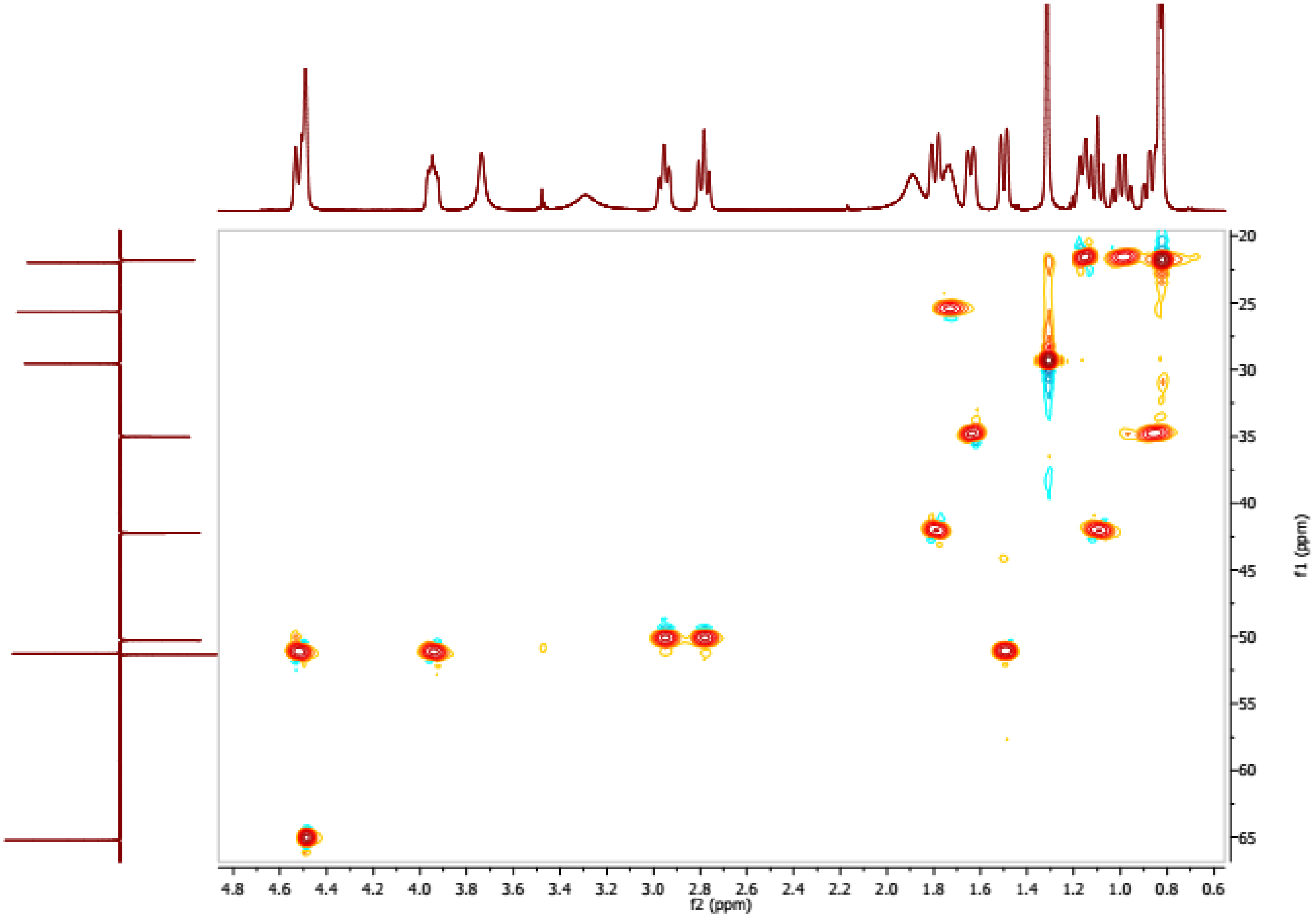

HMBC of compound **20**

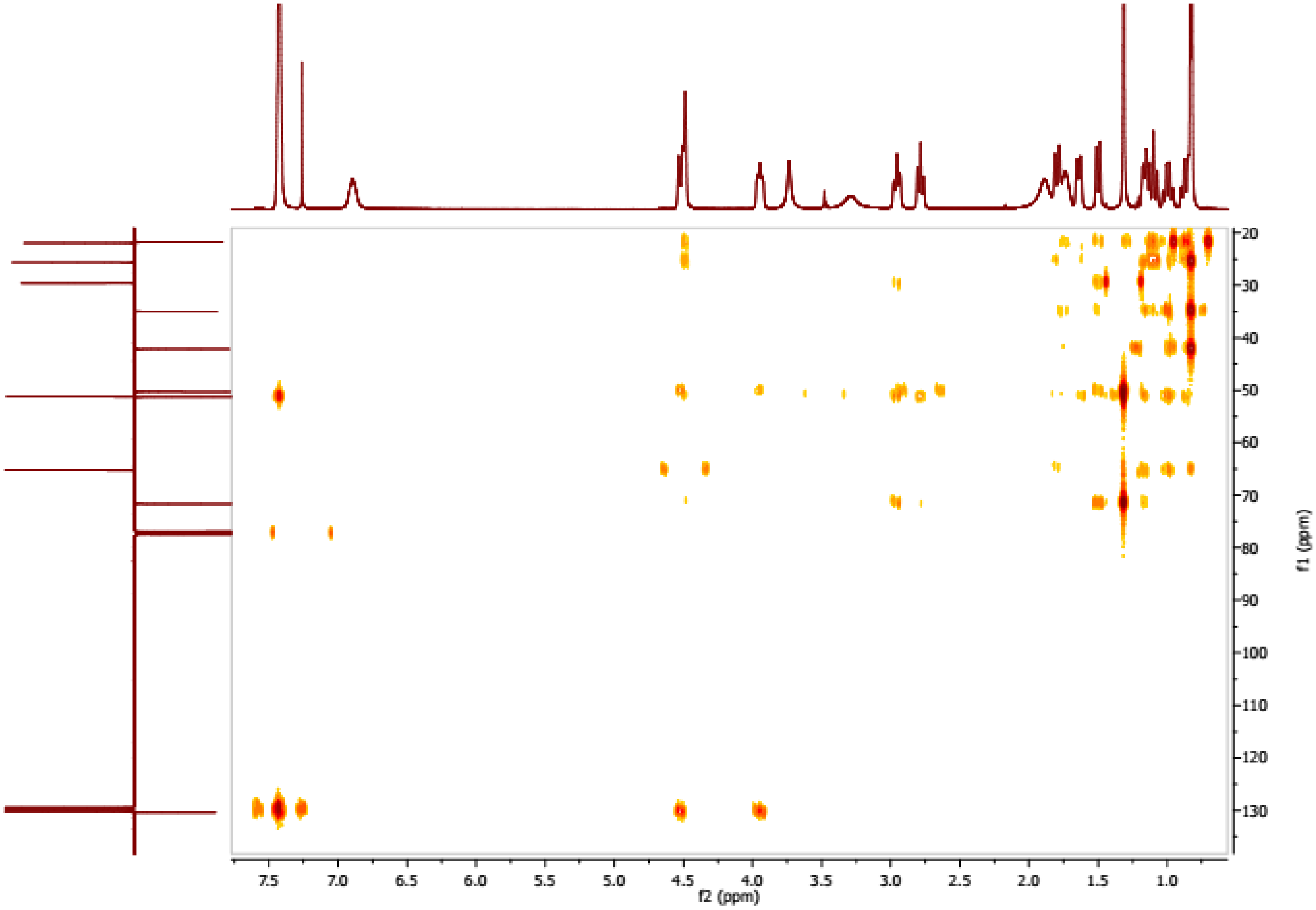

^{1}H-NMR of compound **21**

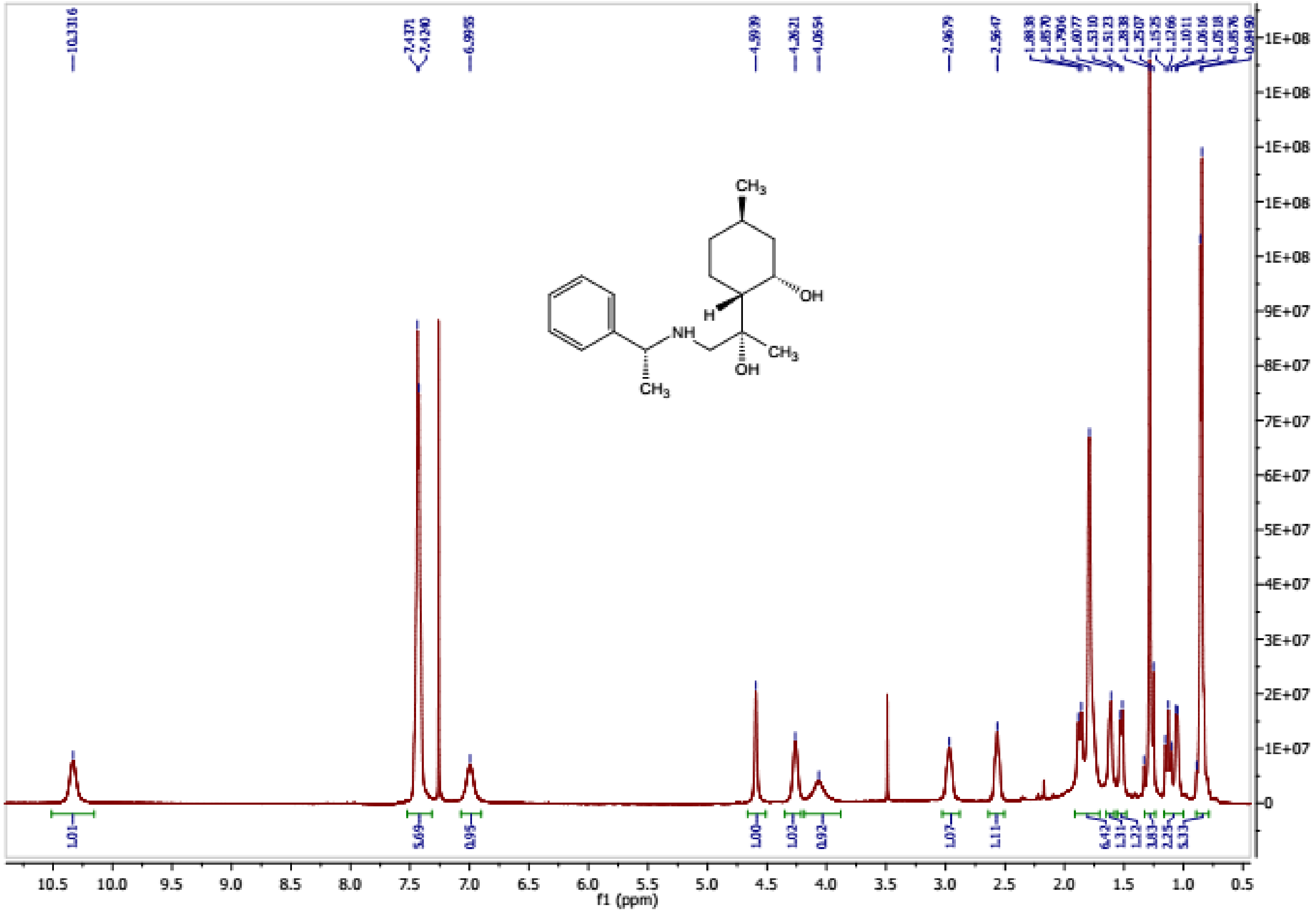

^{13}C-NMR of compound **21**

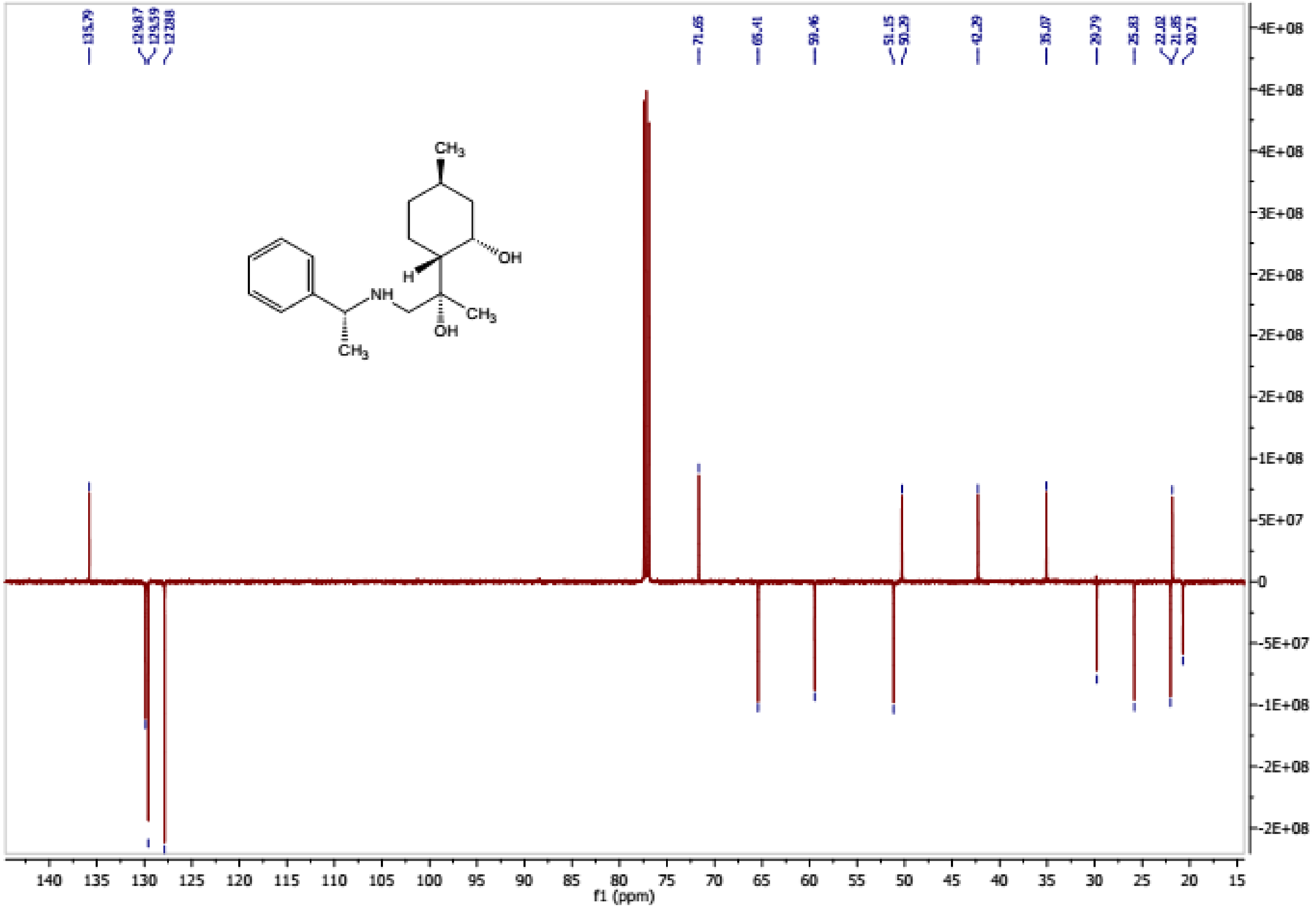

COSY of compound **21**

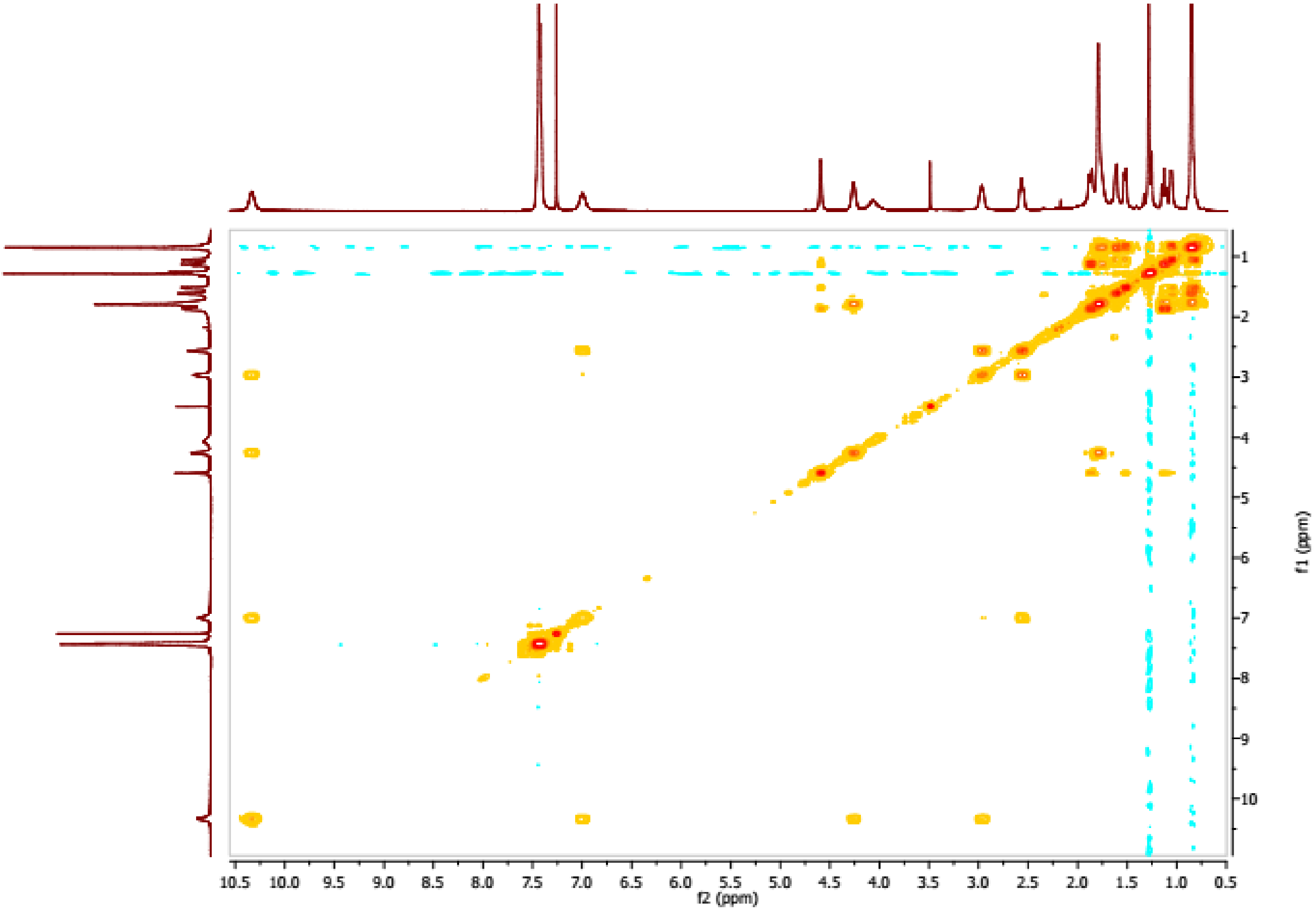

NOESY of compound **21**

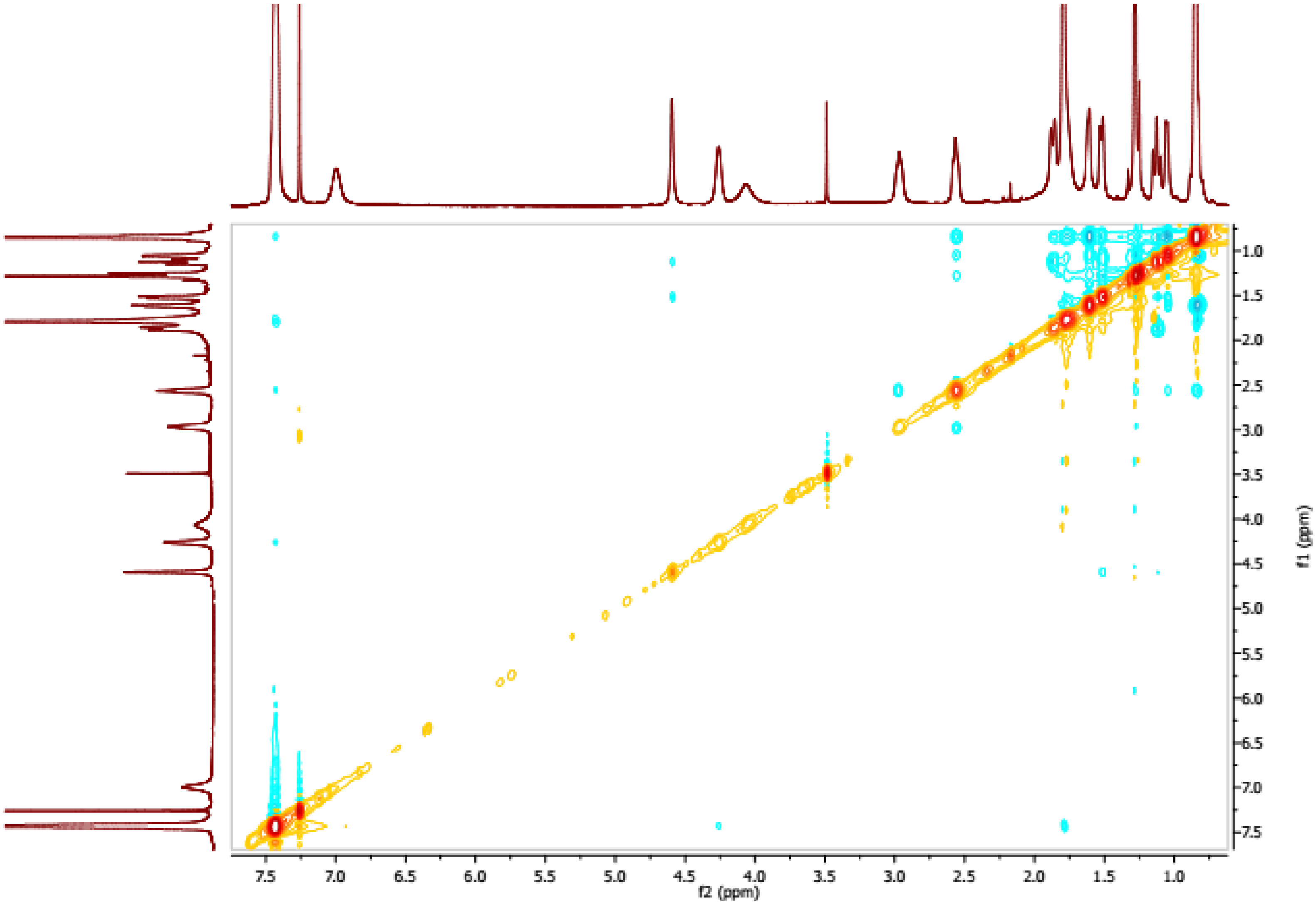

HSQC of compound **21**

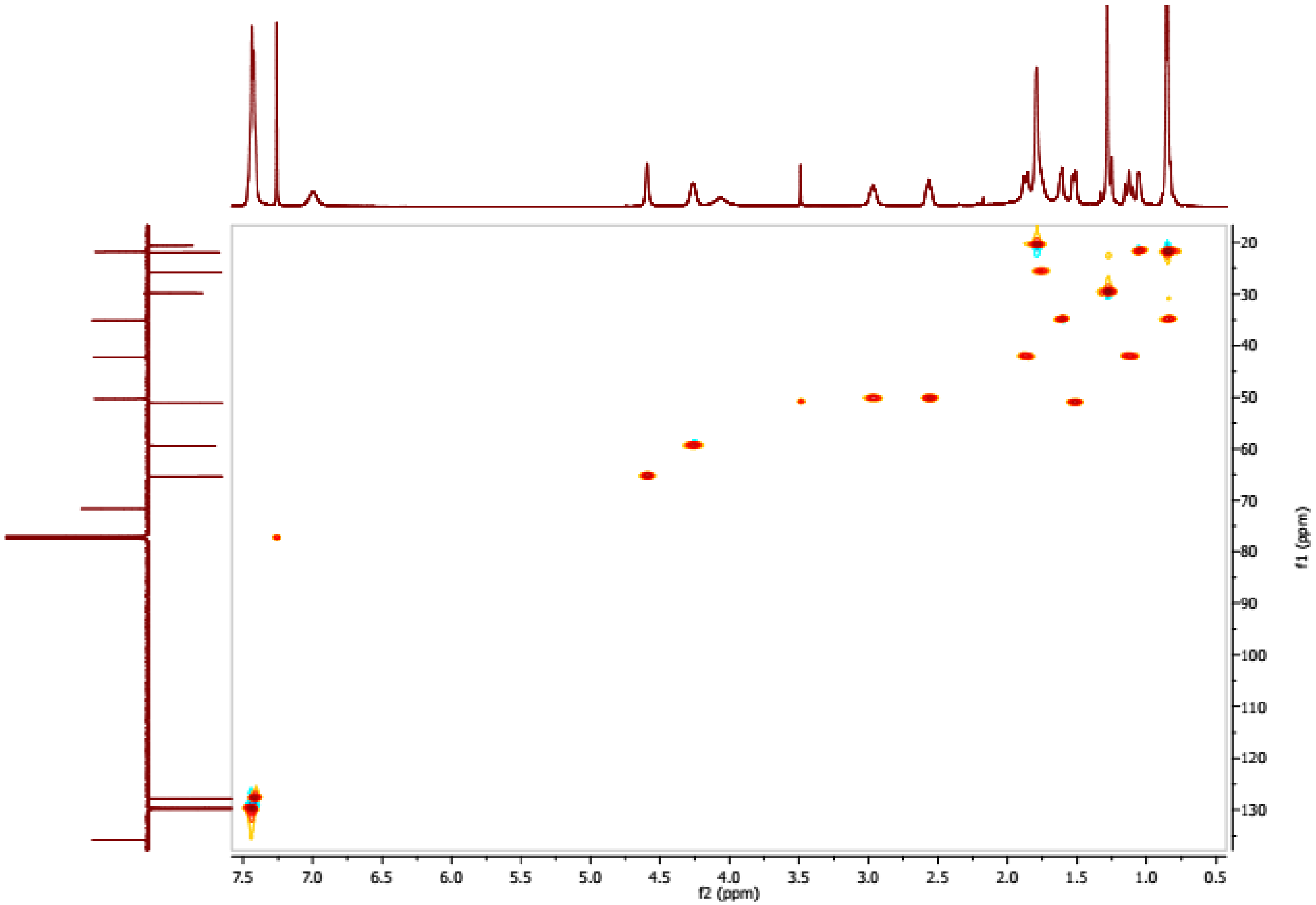

HMBC of compound **21**

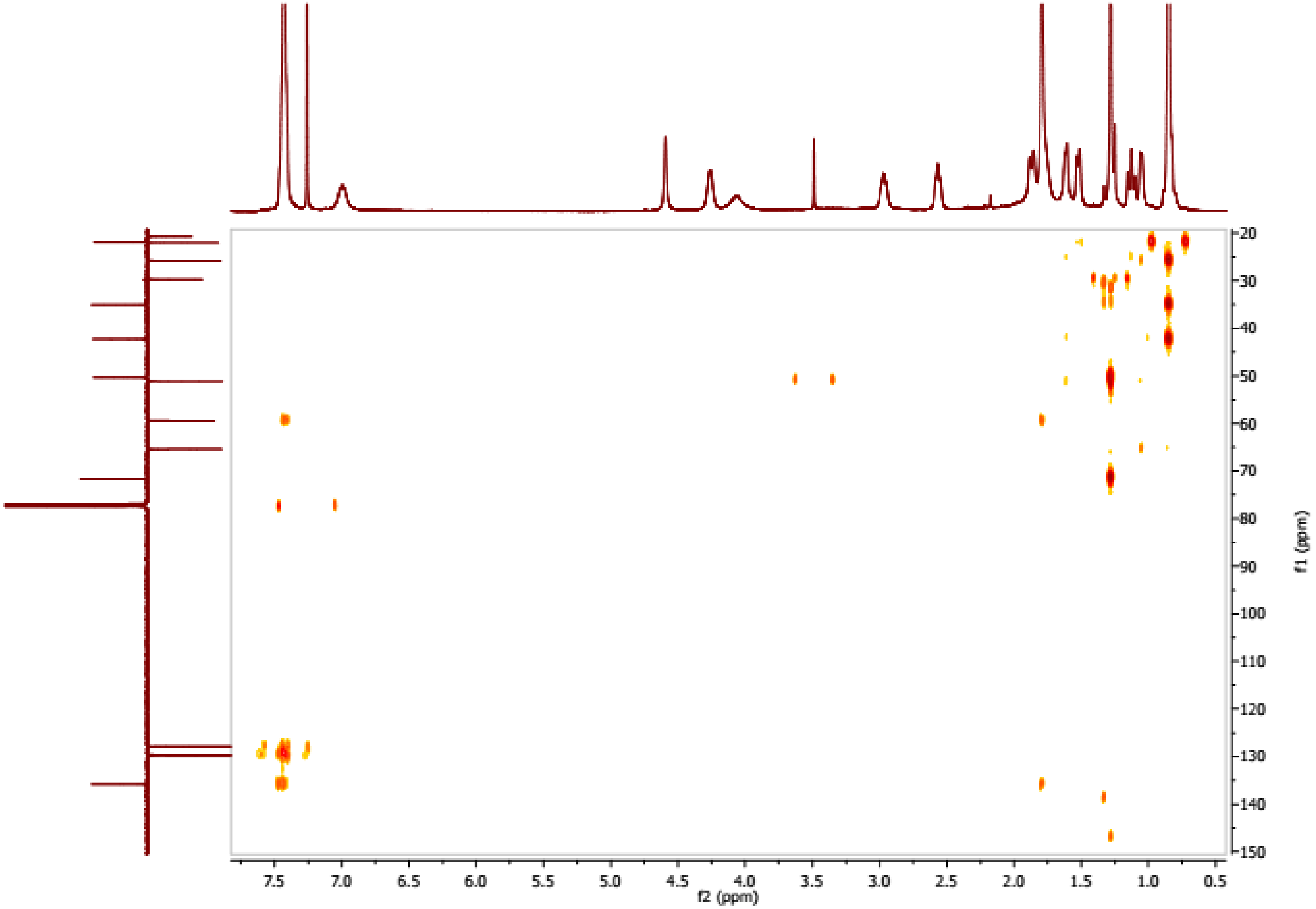

^{1}H-NMR of compound **22**

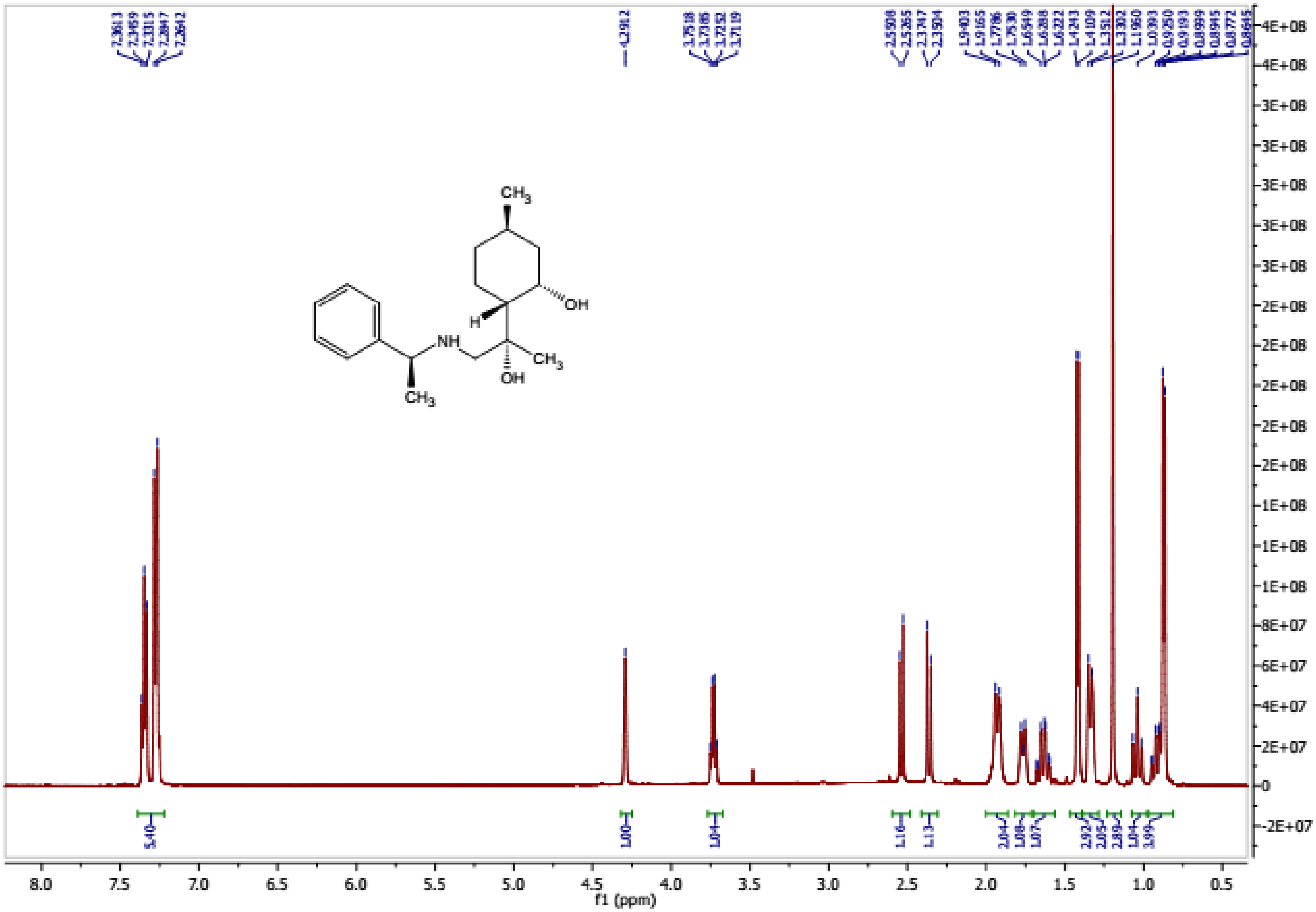

^{13}C-NMR of compound **22**

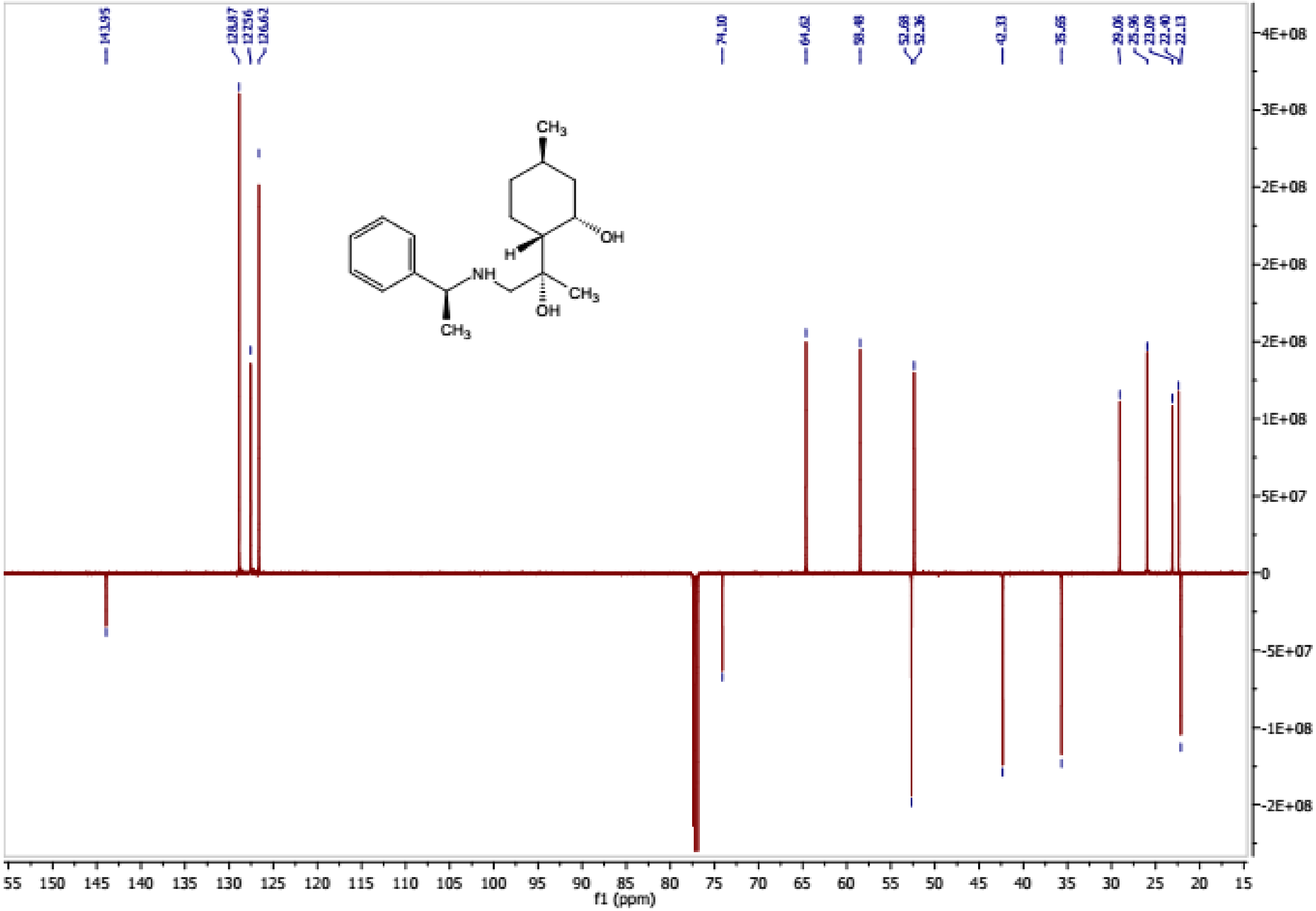

COSY of compound **22**

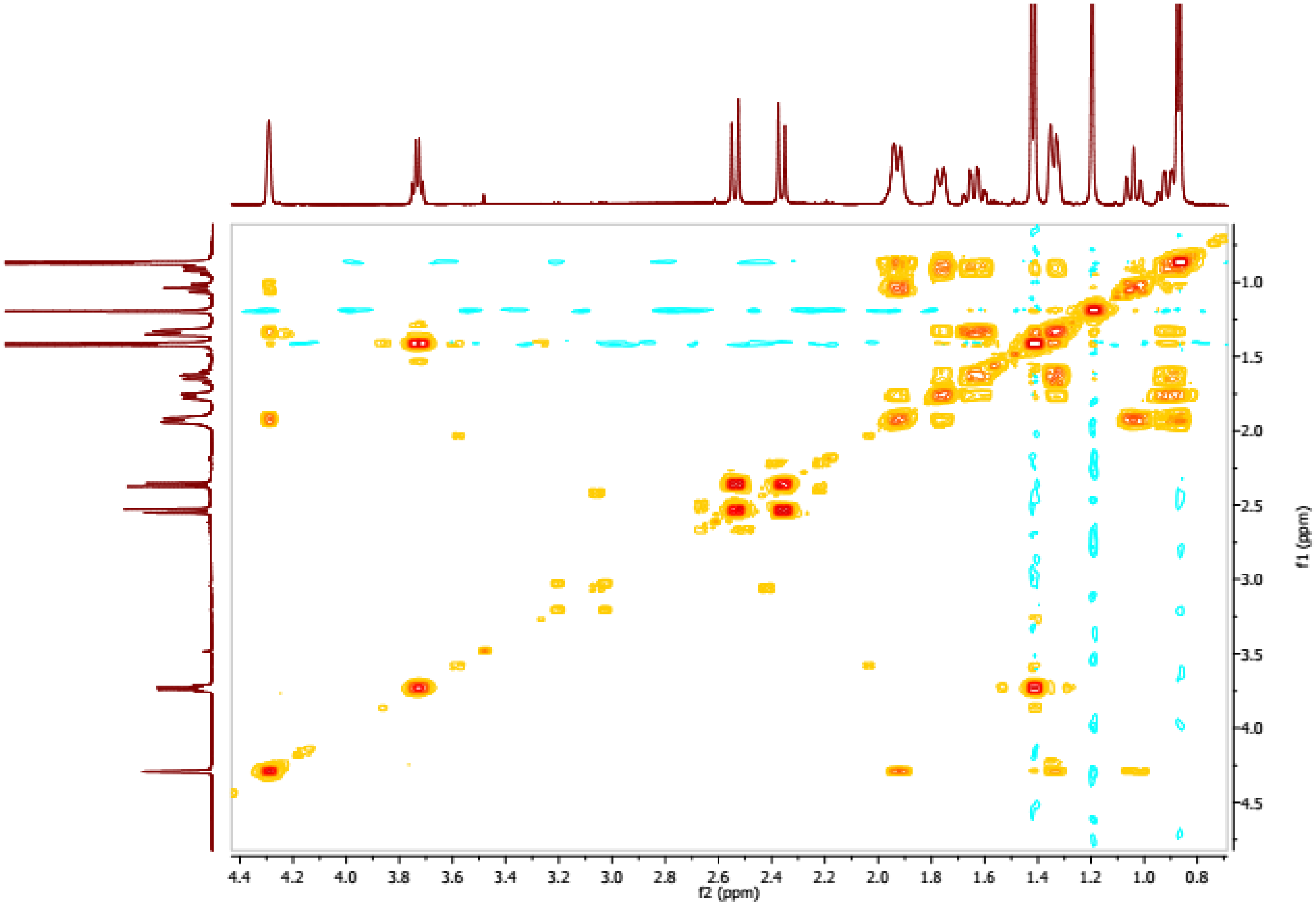

NOESY of compound **22**

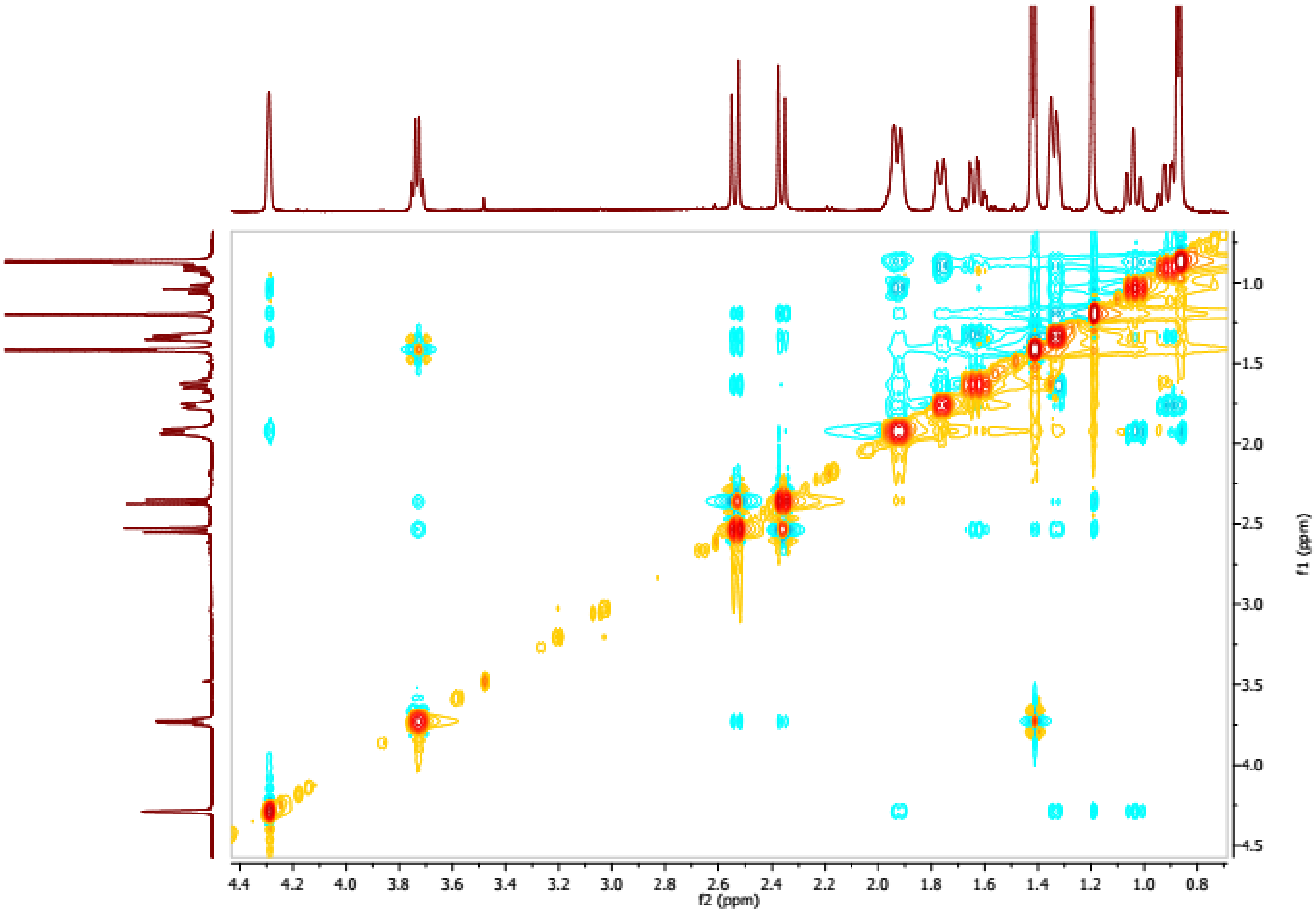

HMBC of compound **22**

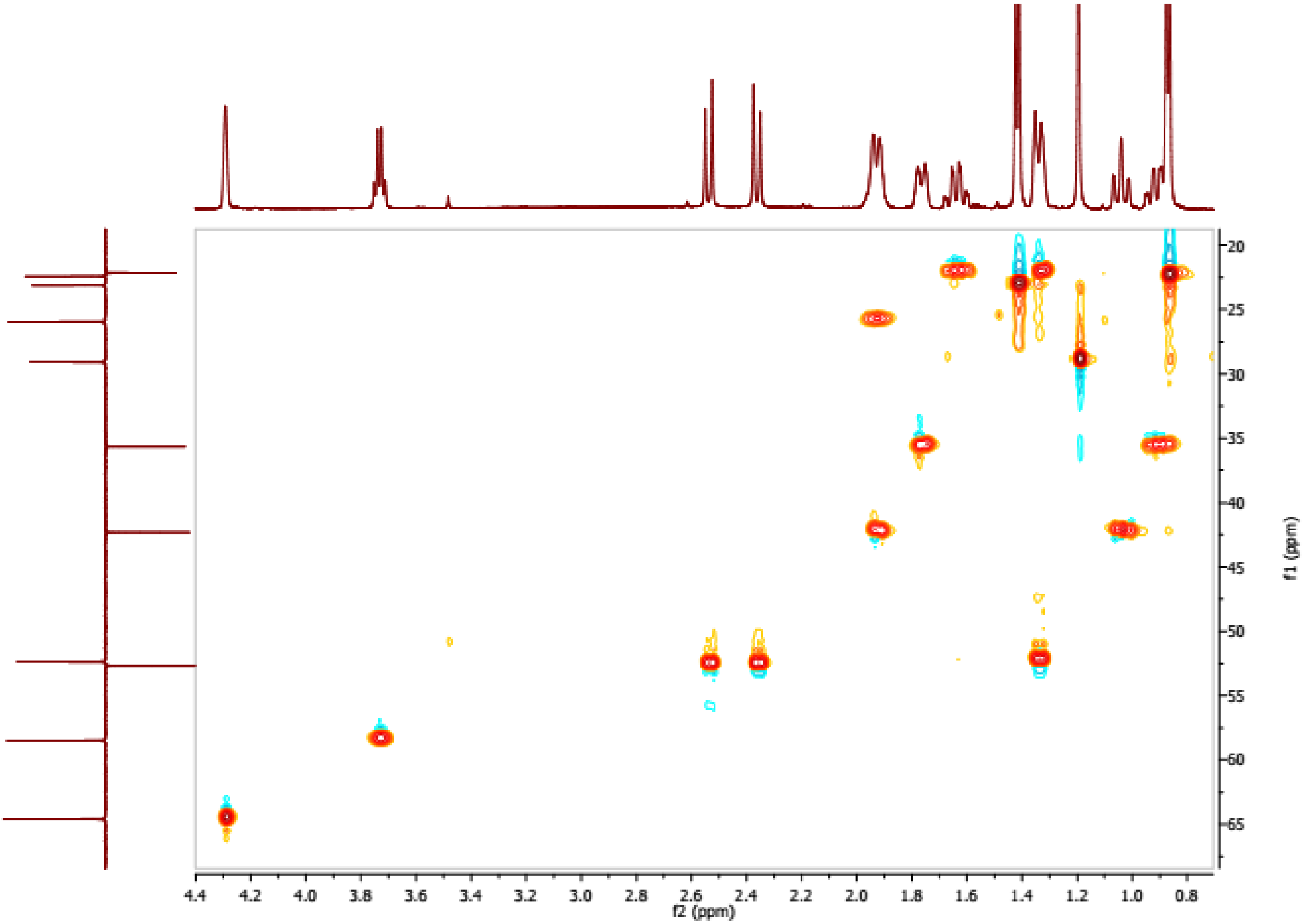

HMBC of compound **22**

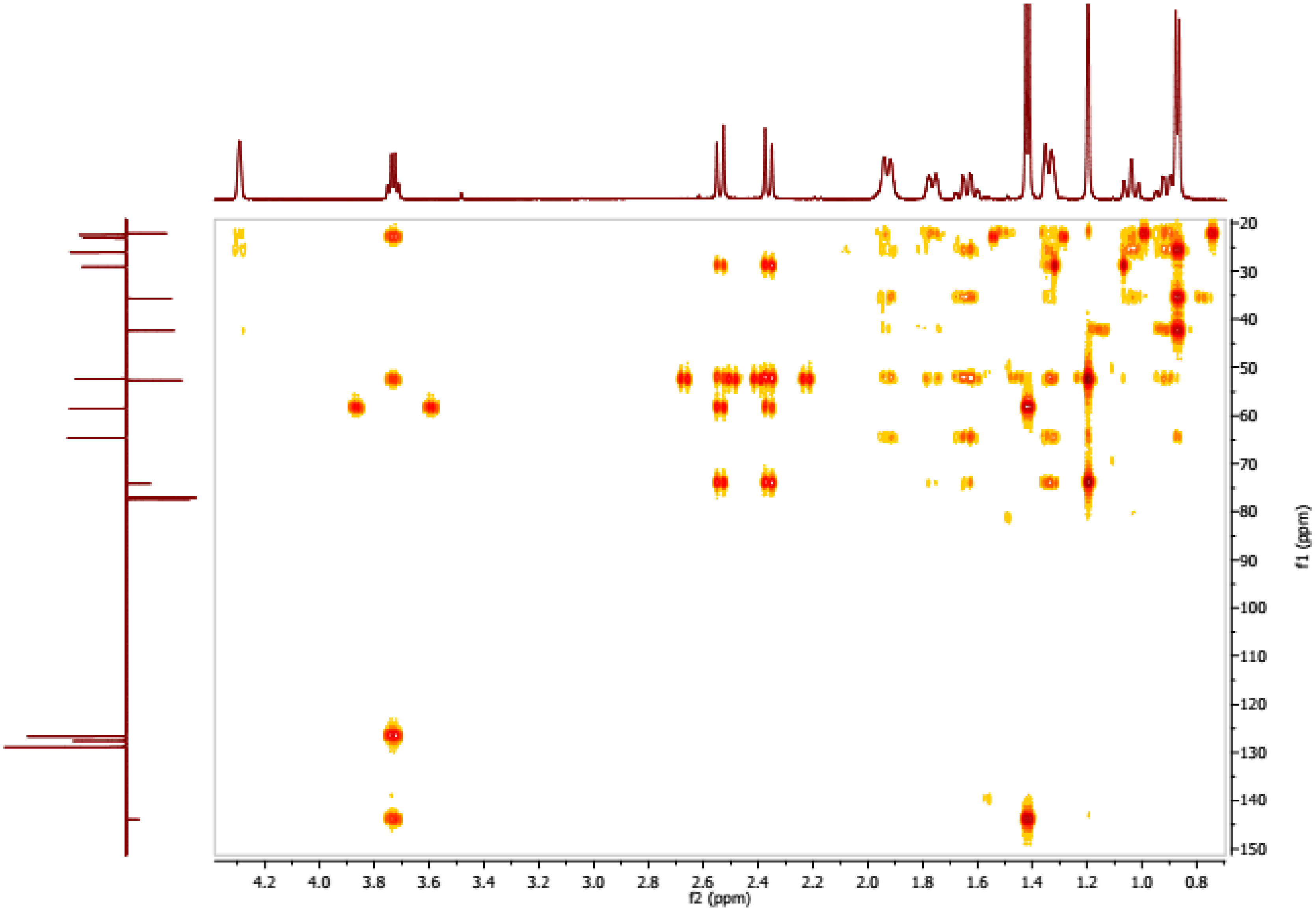

^{1}H-NMR of compound **23**

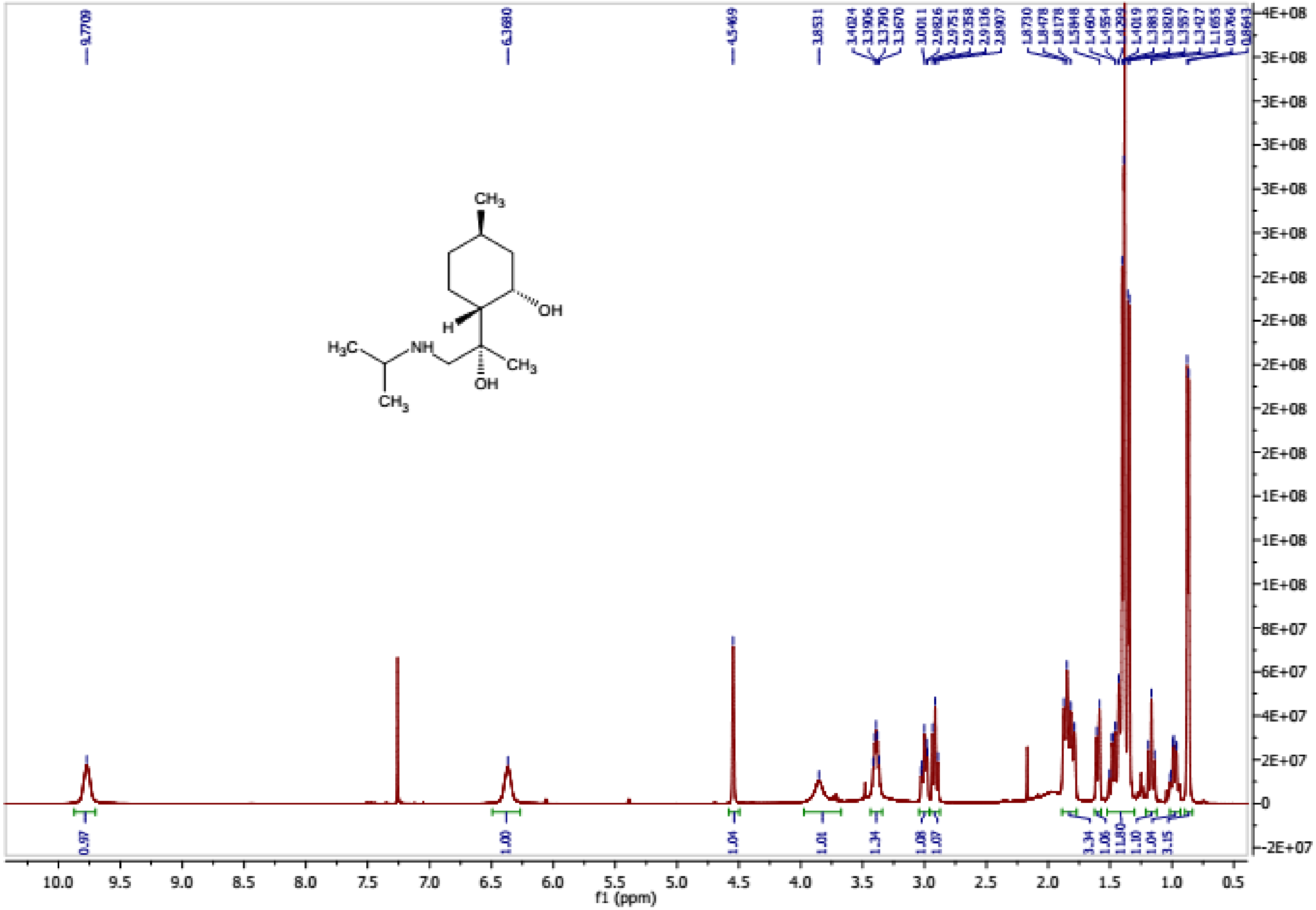

^{13}C-NMR of compound **23**

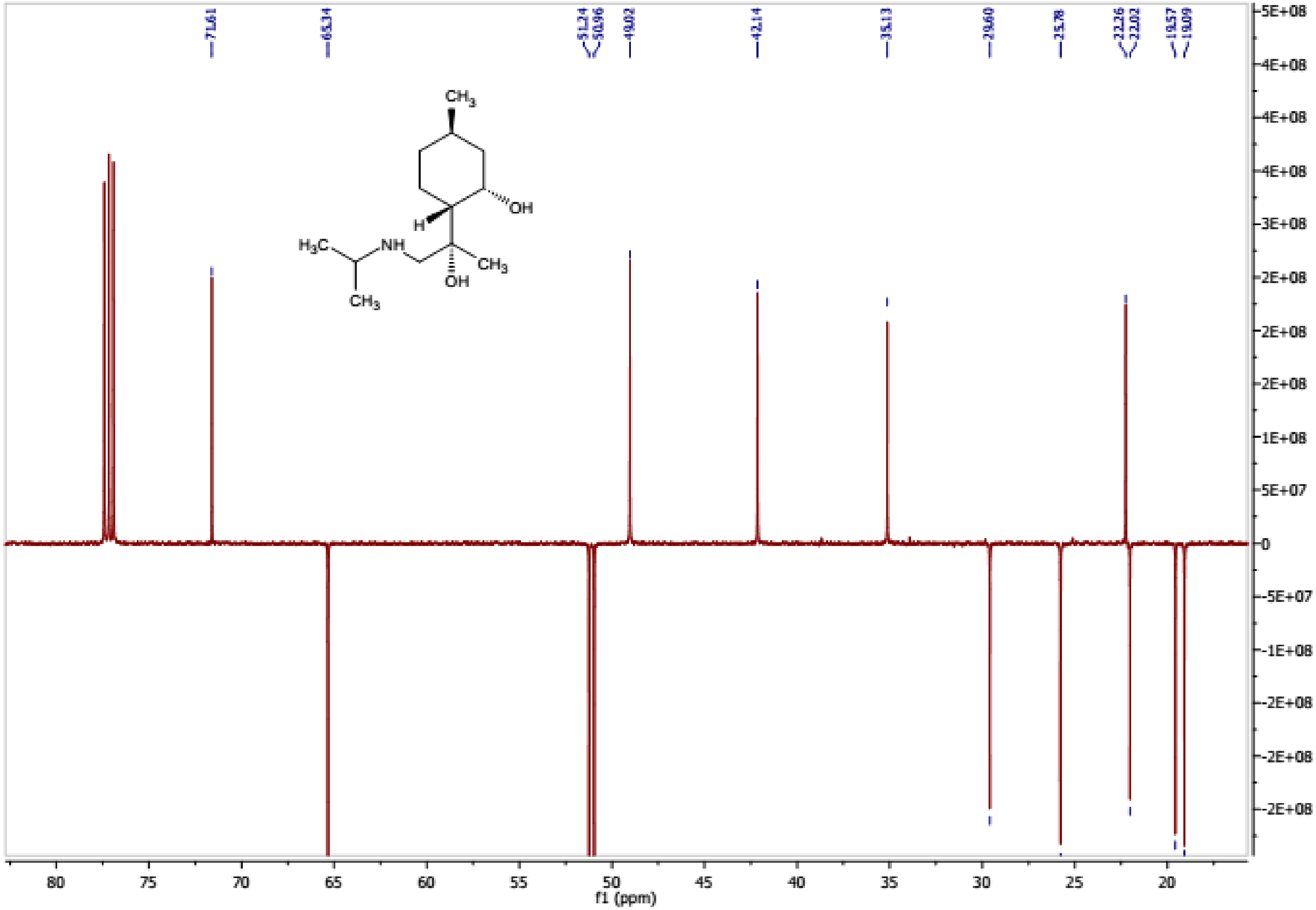

COSY of compound **23**

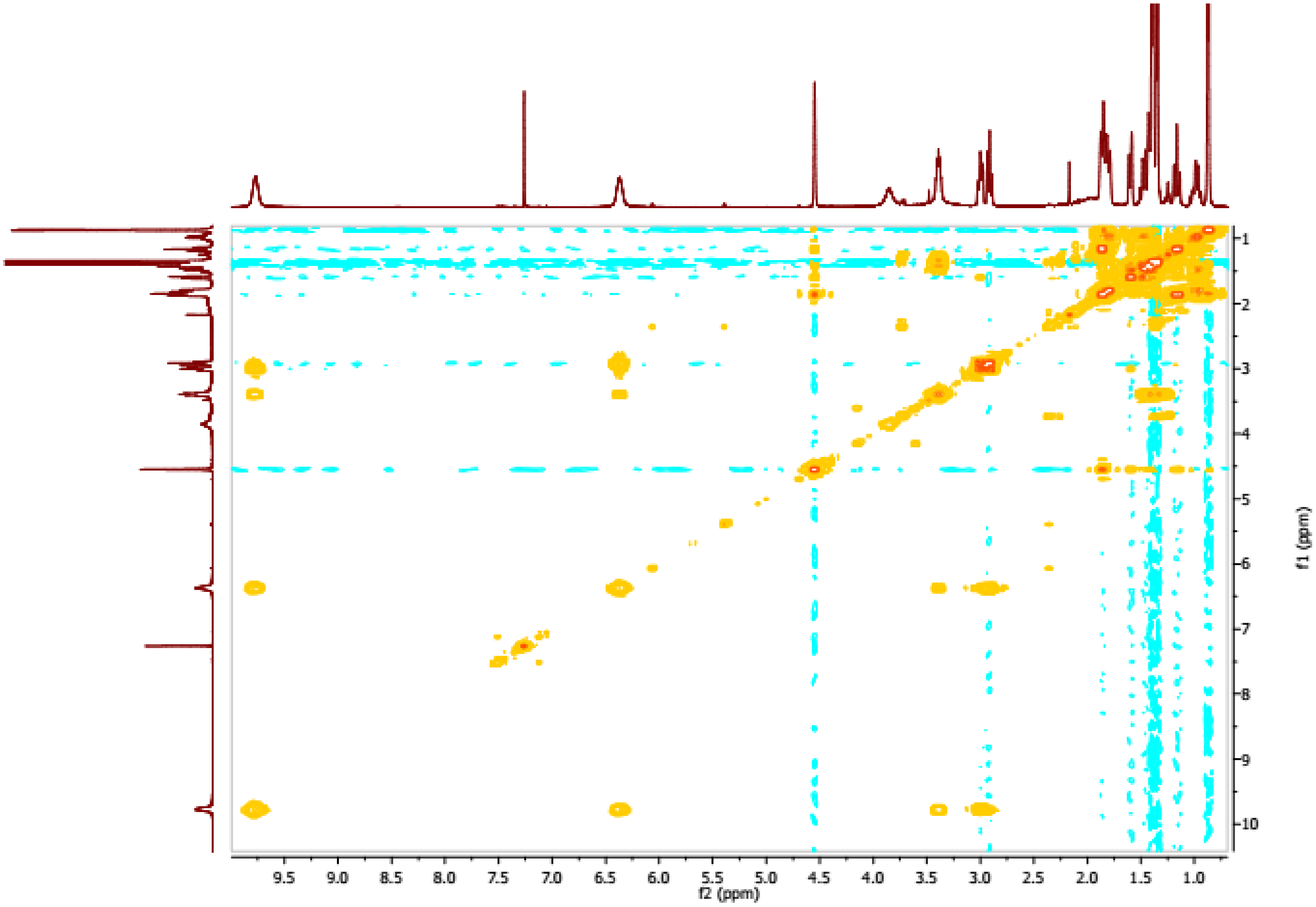

NOESY of compound **23**

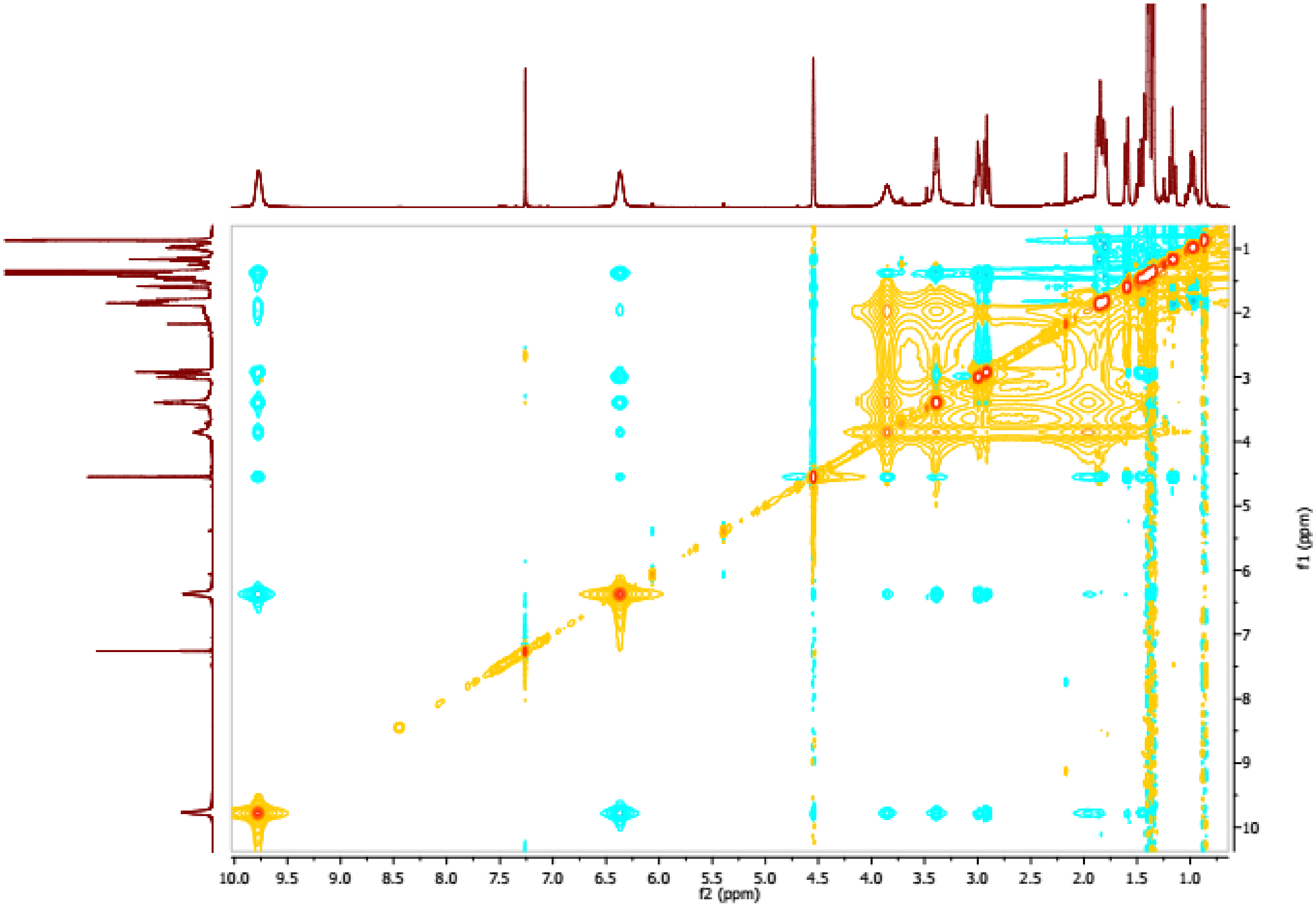

HSQC of compound **23**

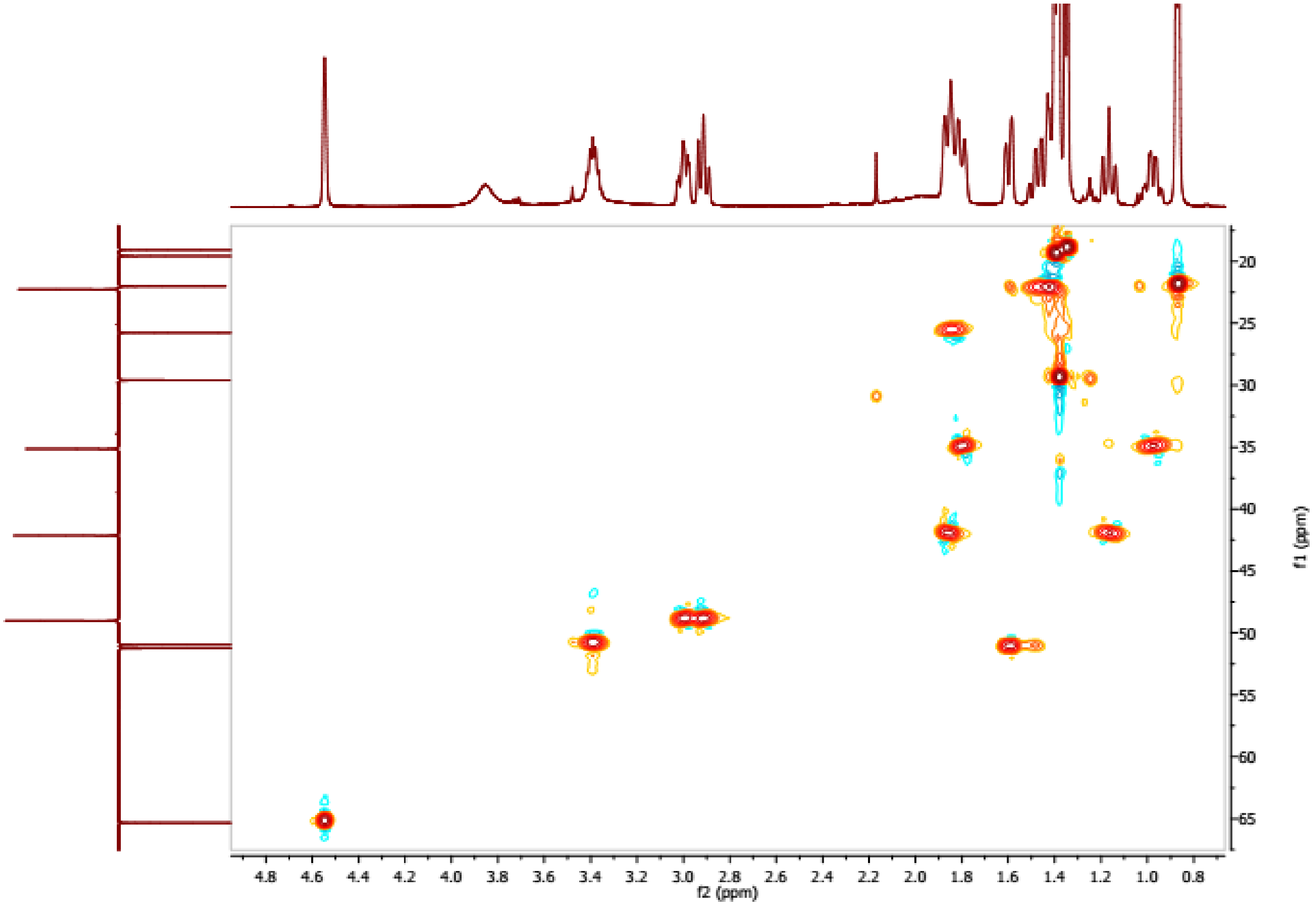

HMBC of compound **23**

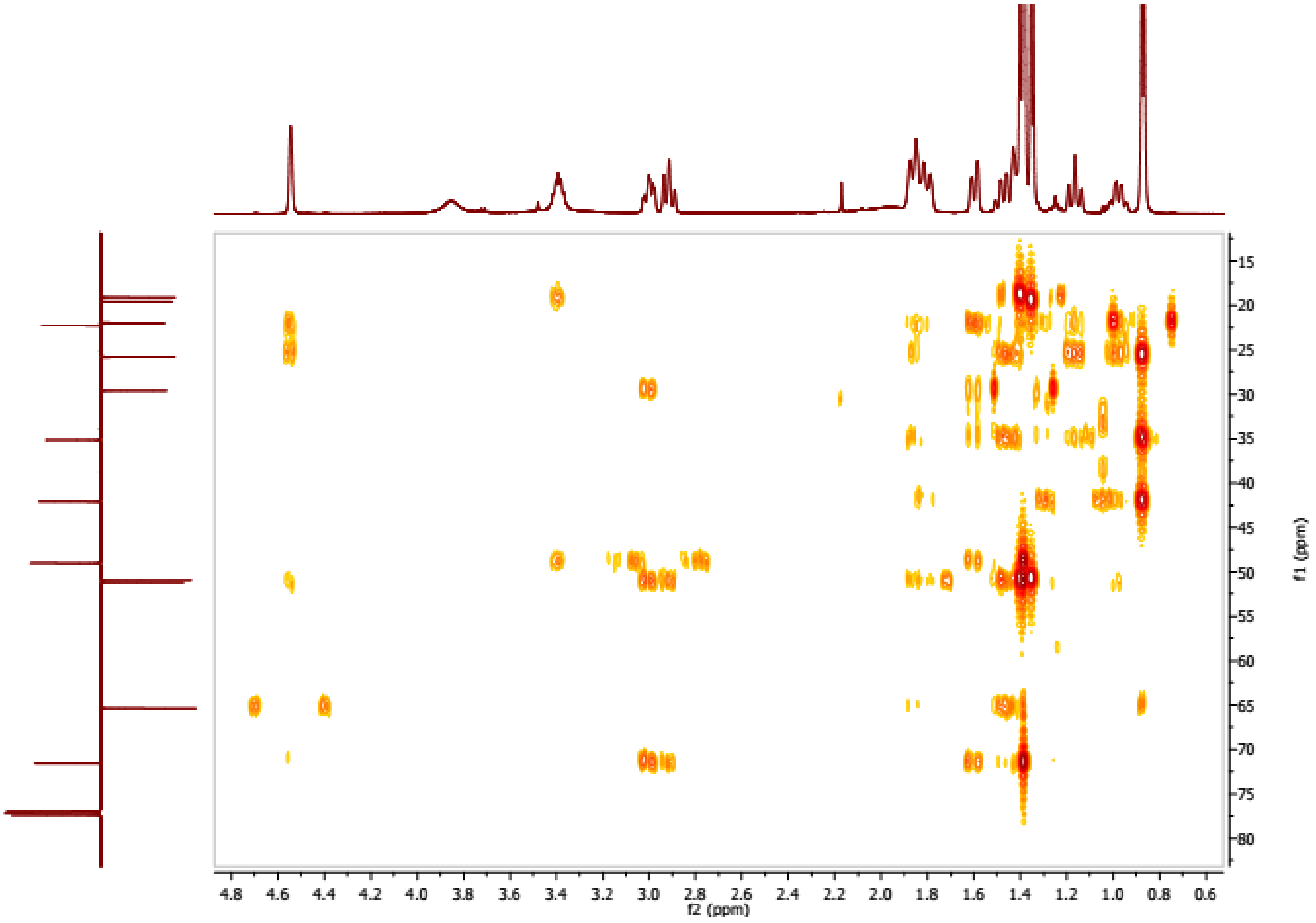

^{1}H-NMR of compound **24**

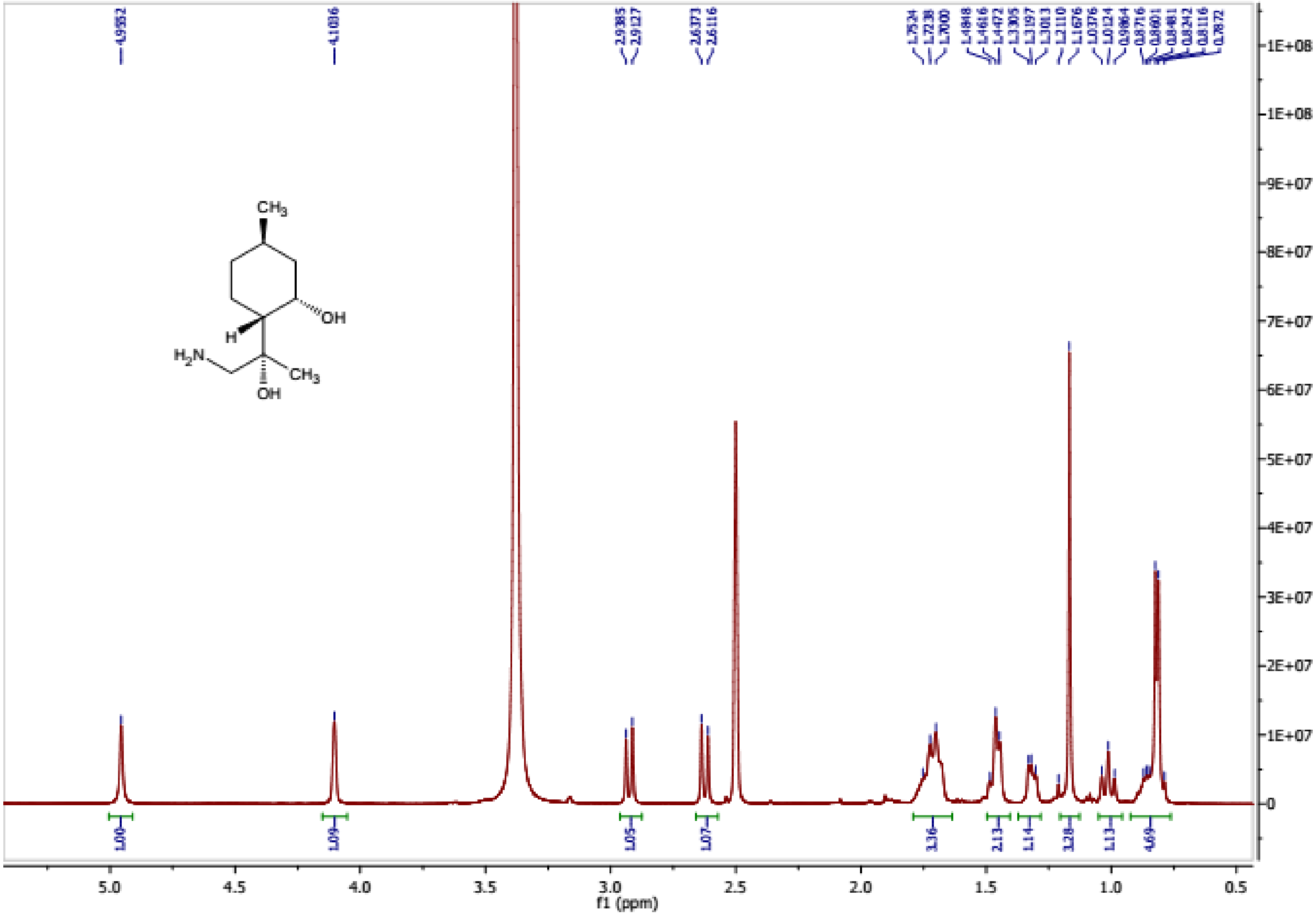

^{13}C-NMR of compound **24**

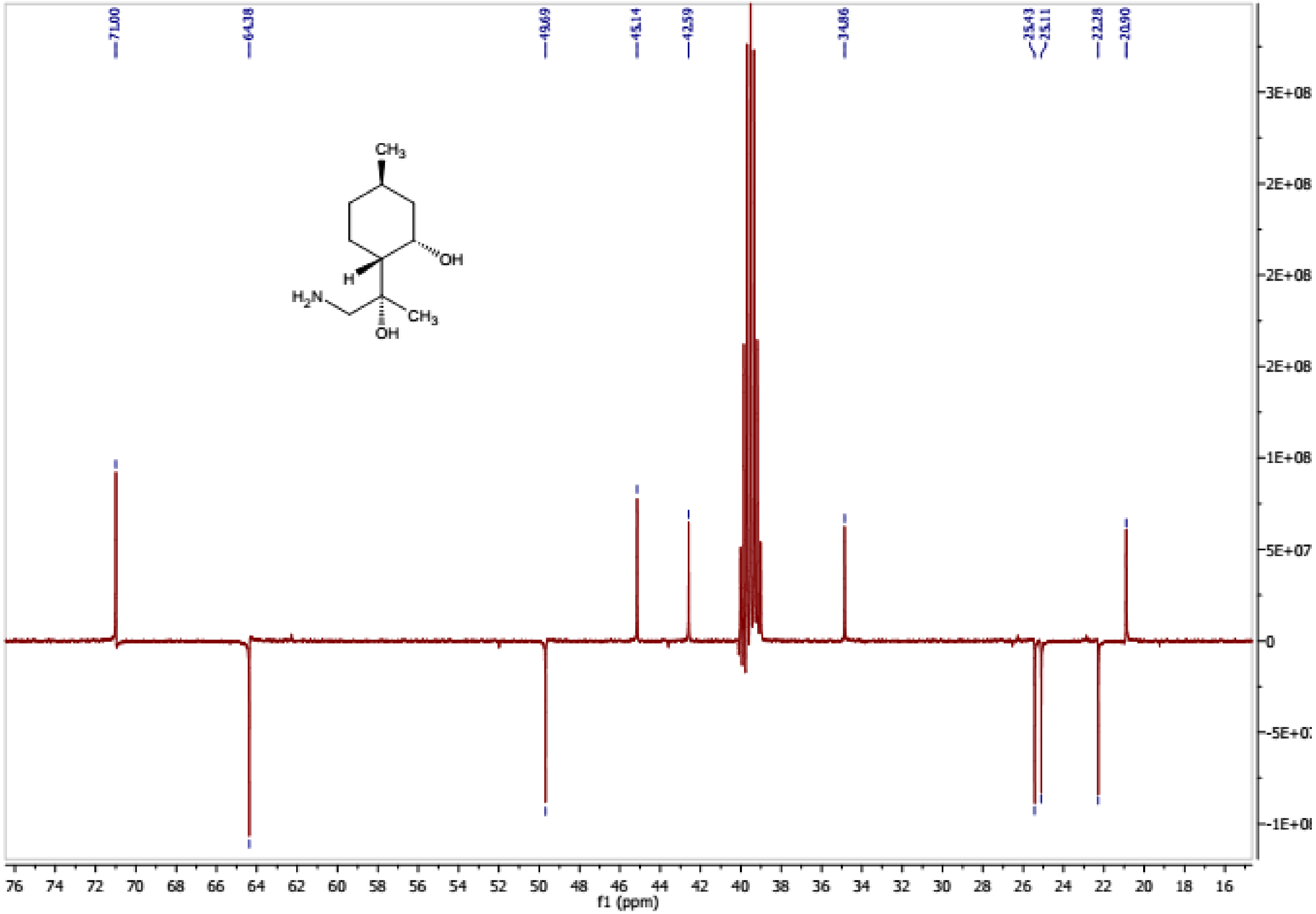

^{1}H-NMR of compound **25**

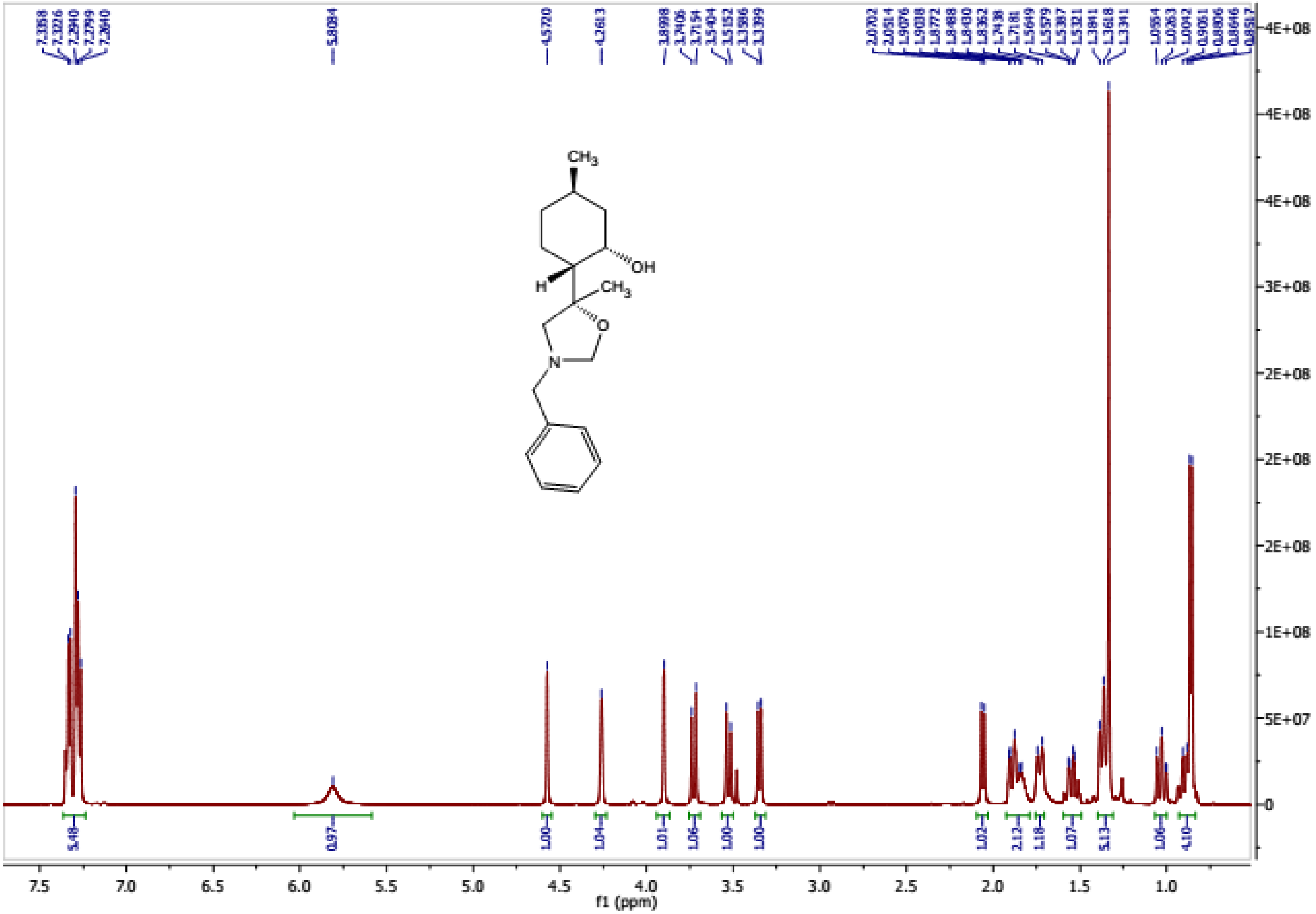

^{13}C-NMR of compound **25**

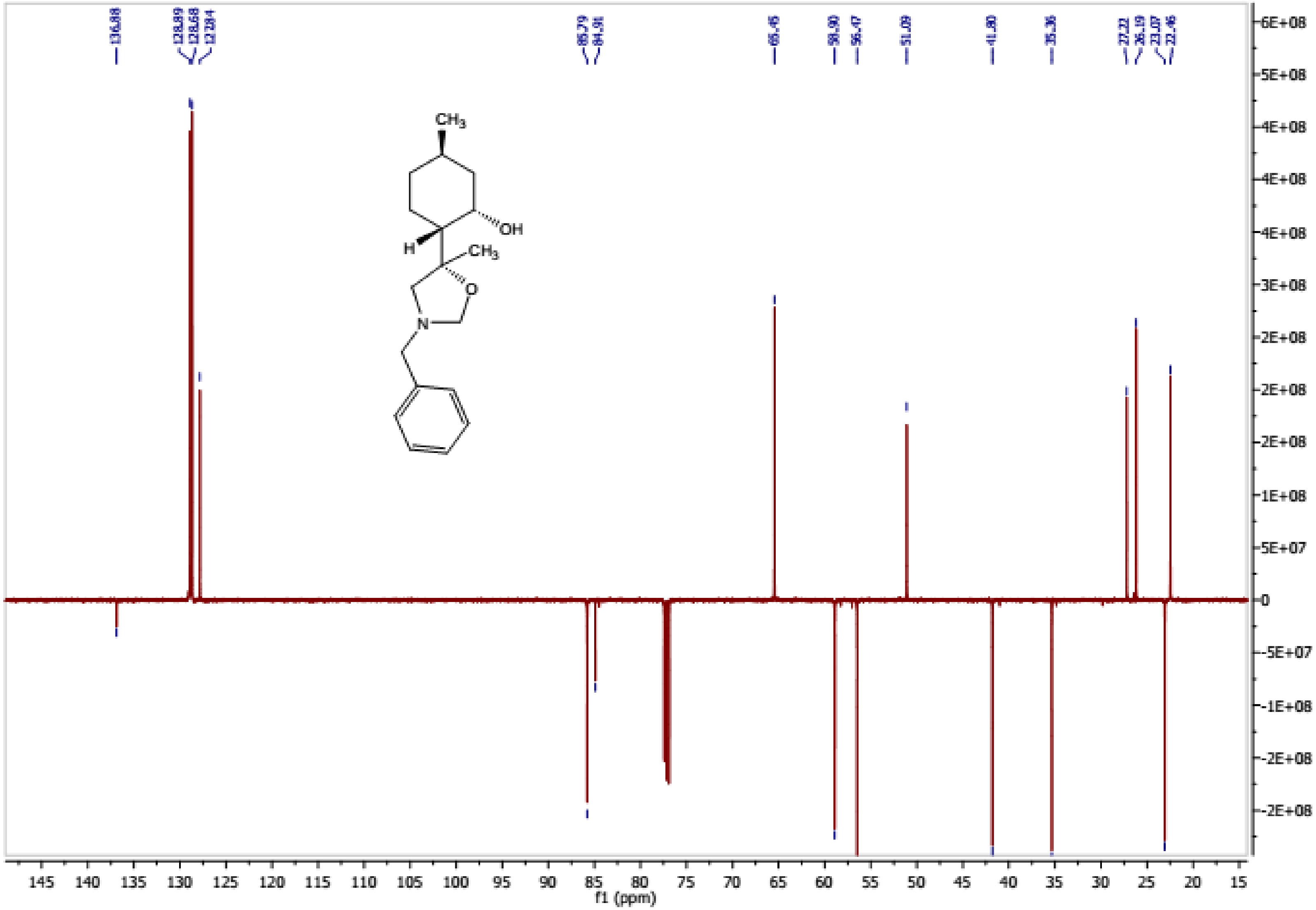

HSQC of compound **25**

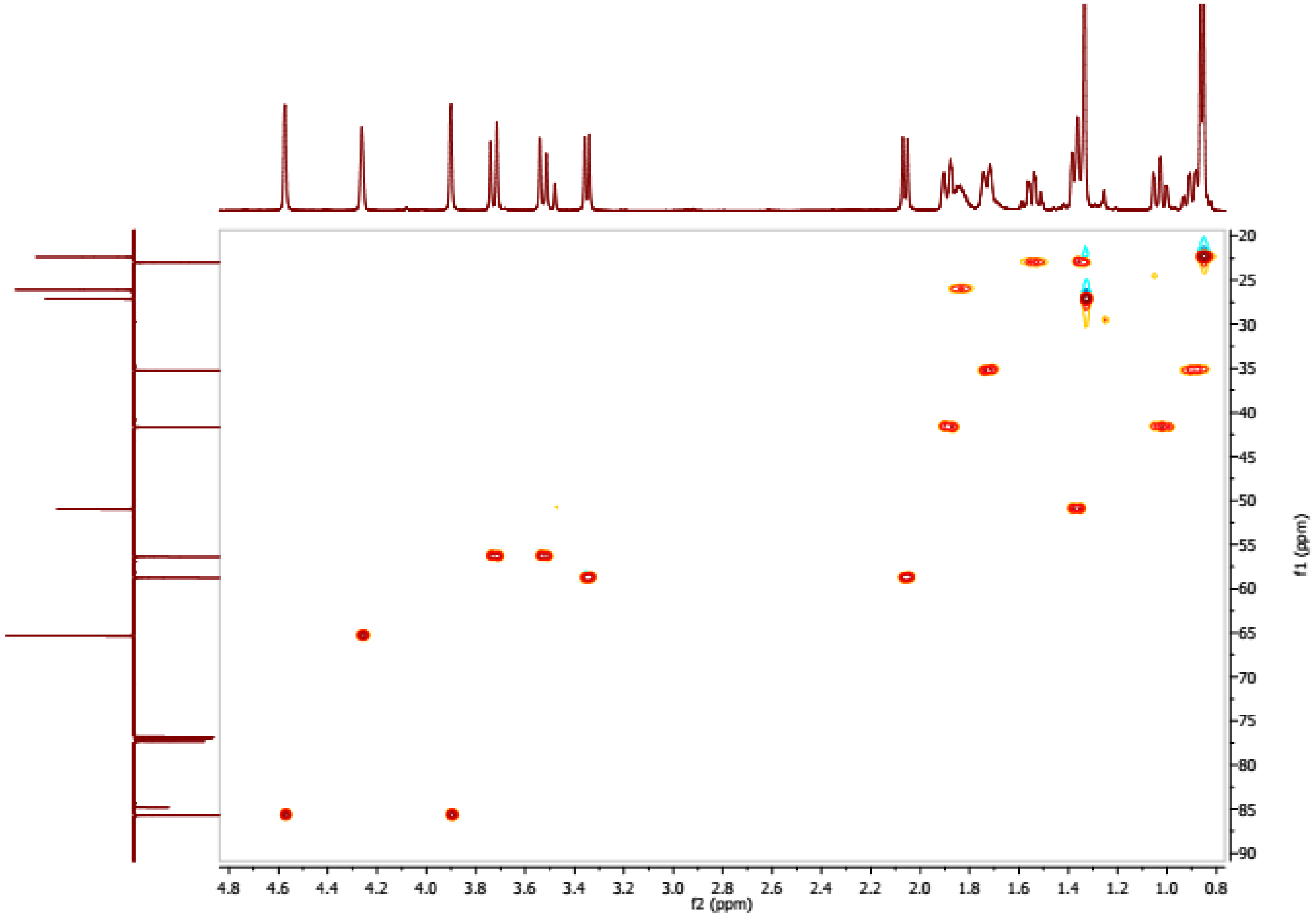

HMBC of compound **25**

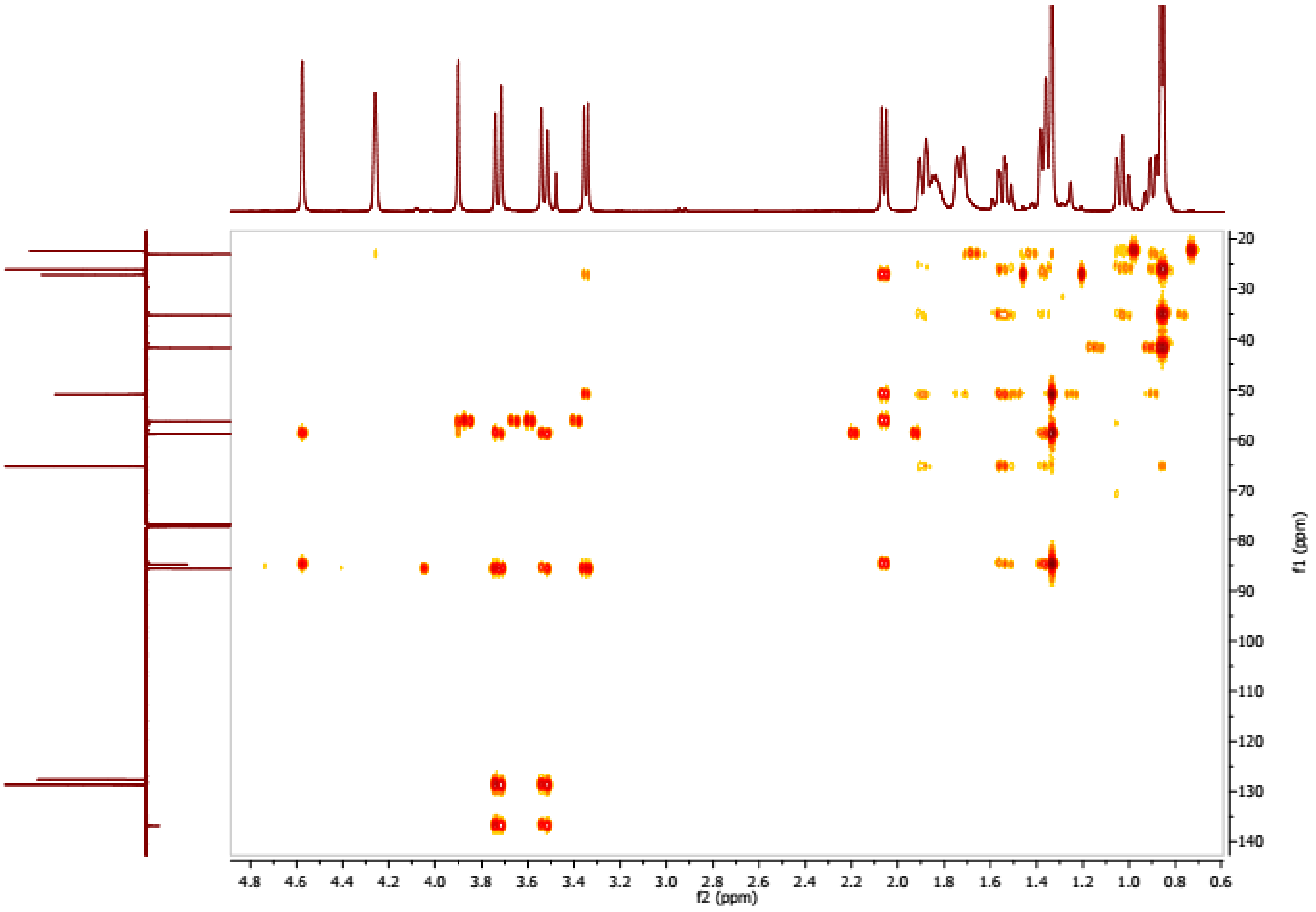

^{1}H-NMR of compound **26**

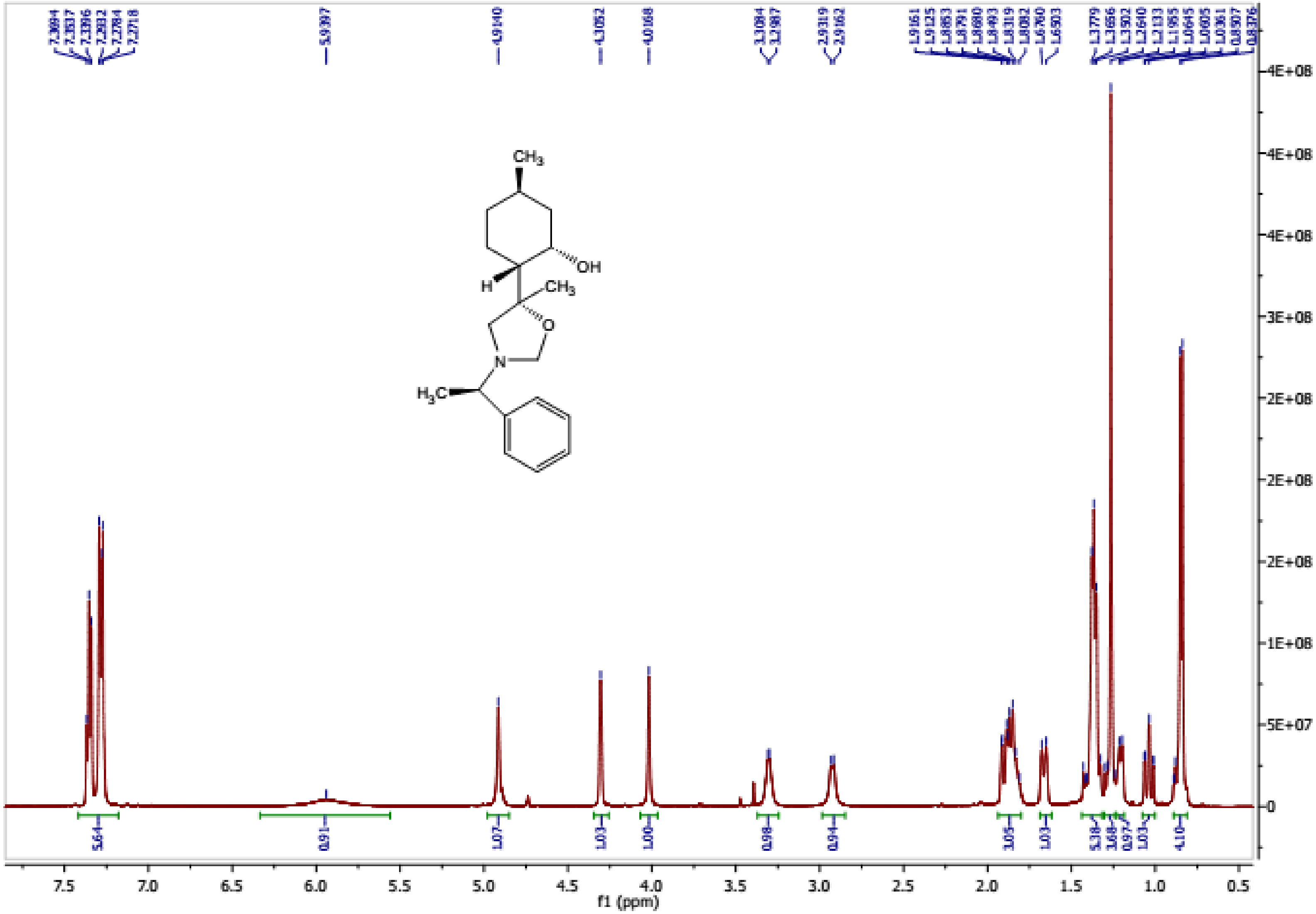

^{13}C-NMR of compound **26**

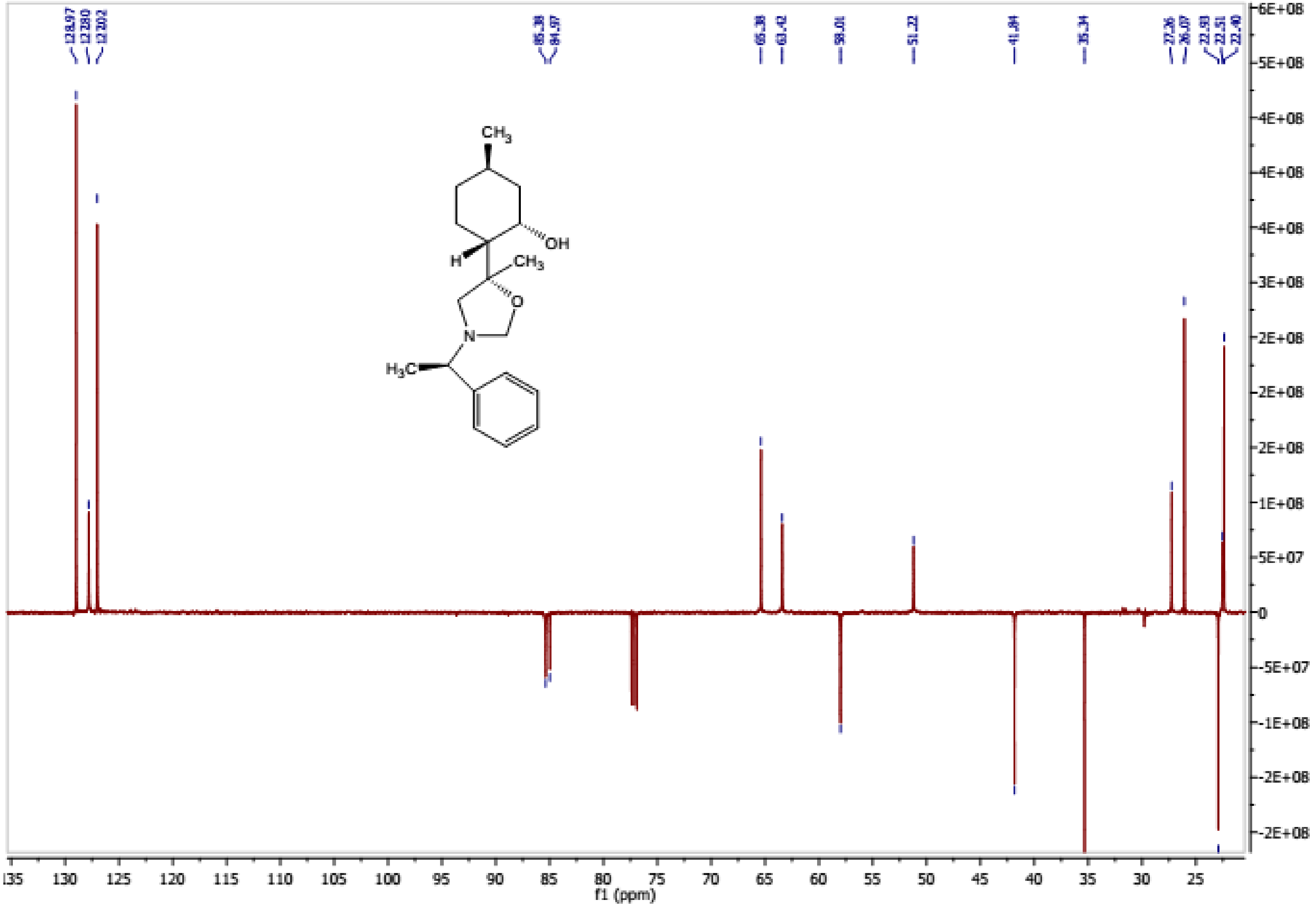

HSQC of compound **26**

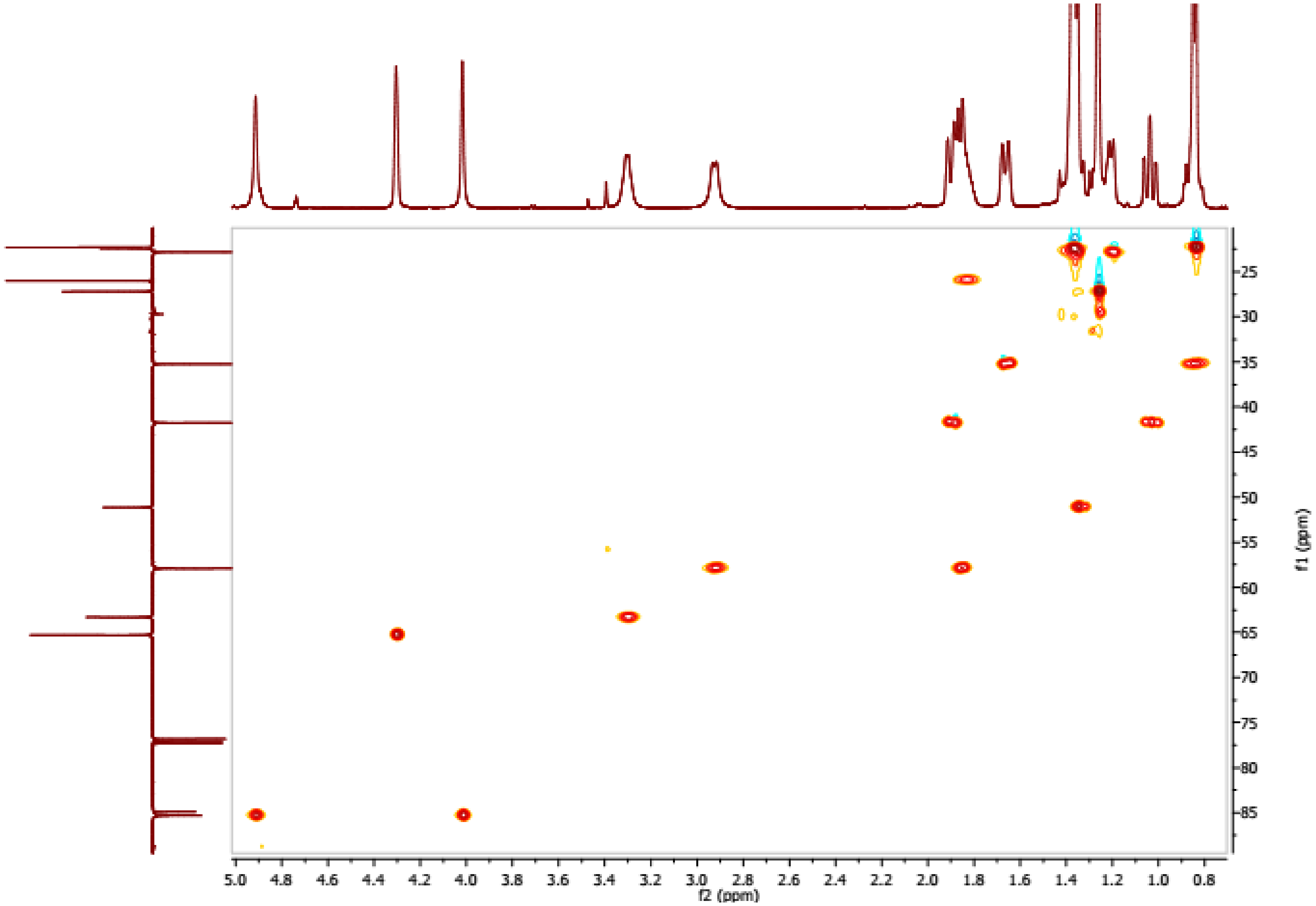

HMBC of compound **26**

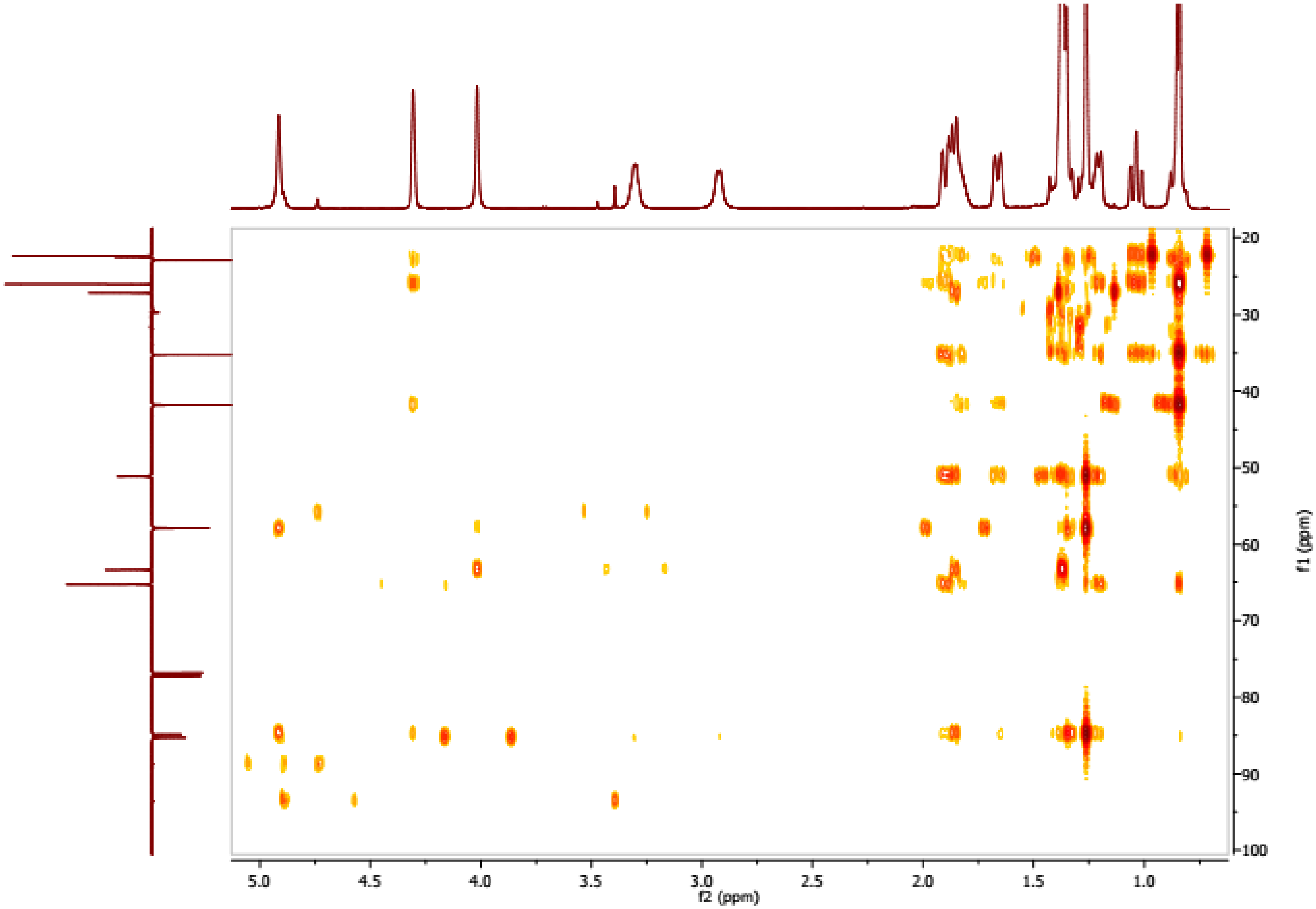

^{1}H-NMR of compound **27**

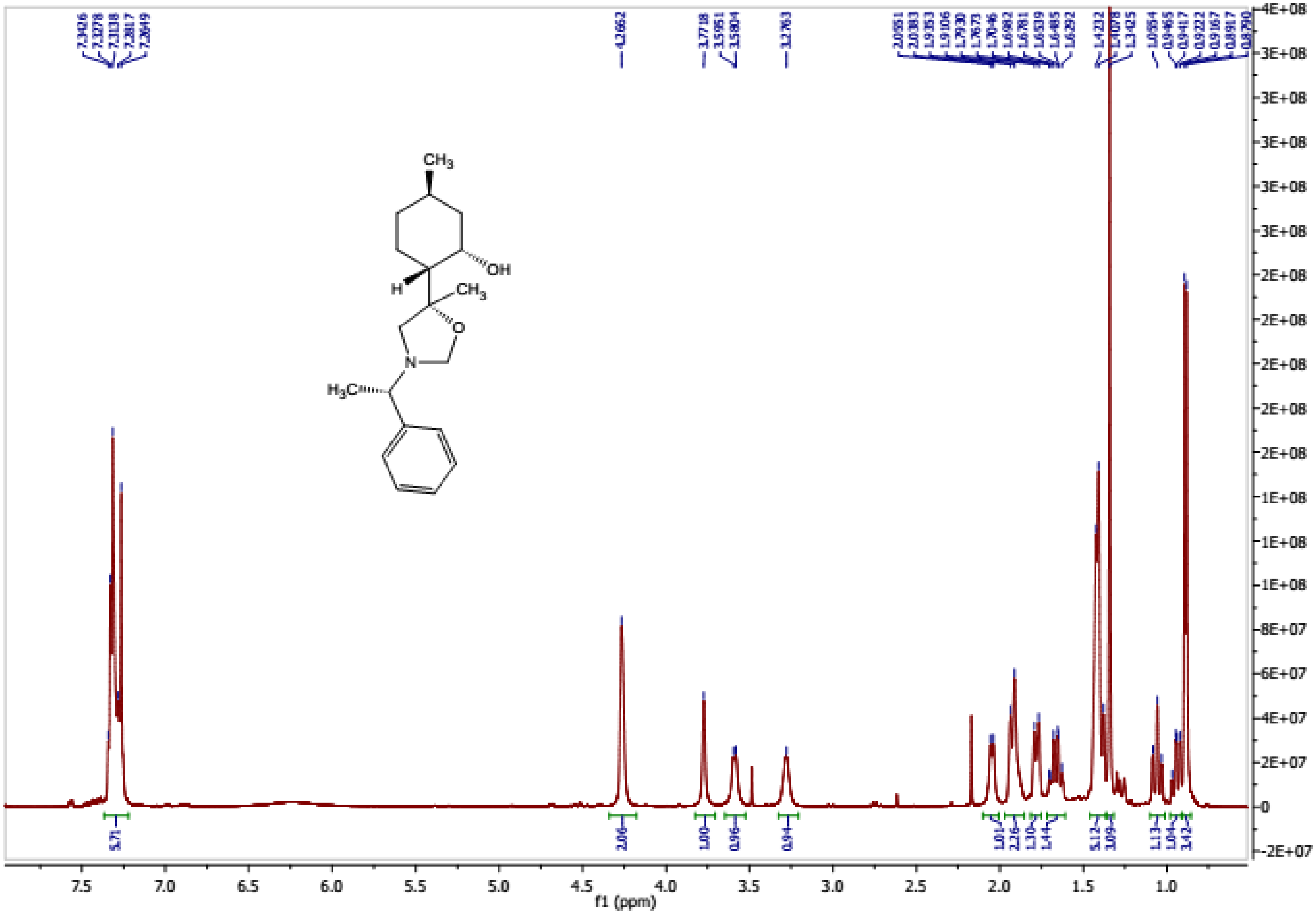

^{13}C-NMR of compound **27**

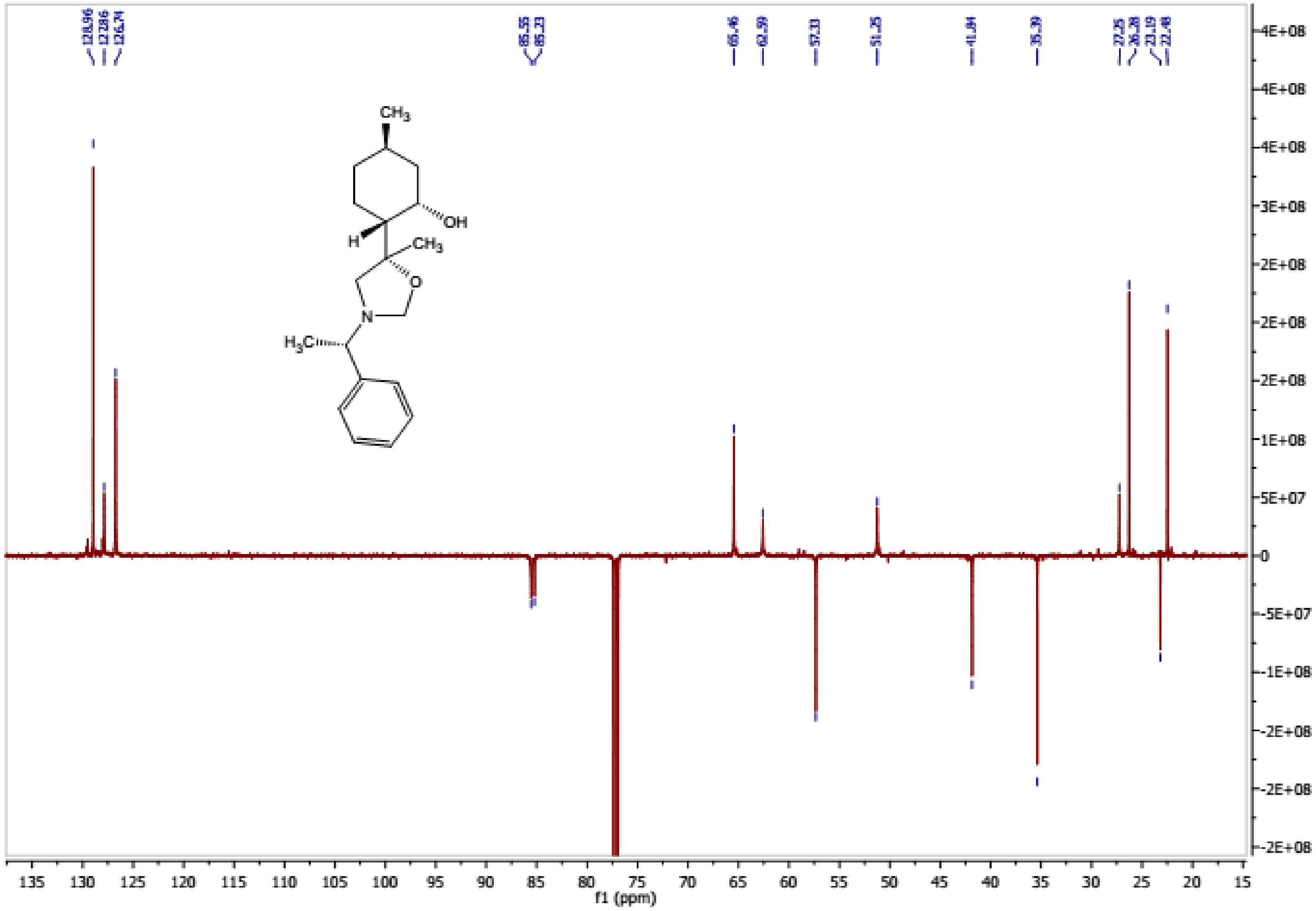

HSQC of compound **27**

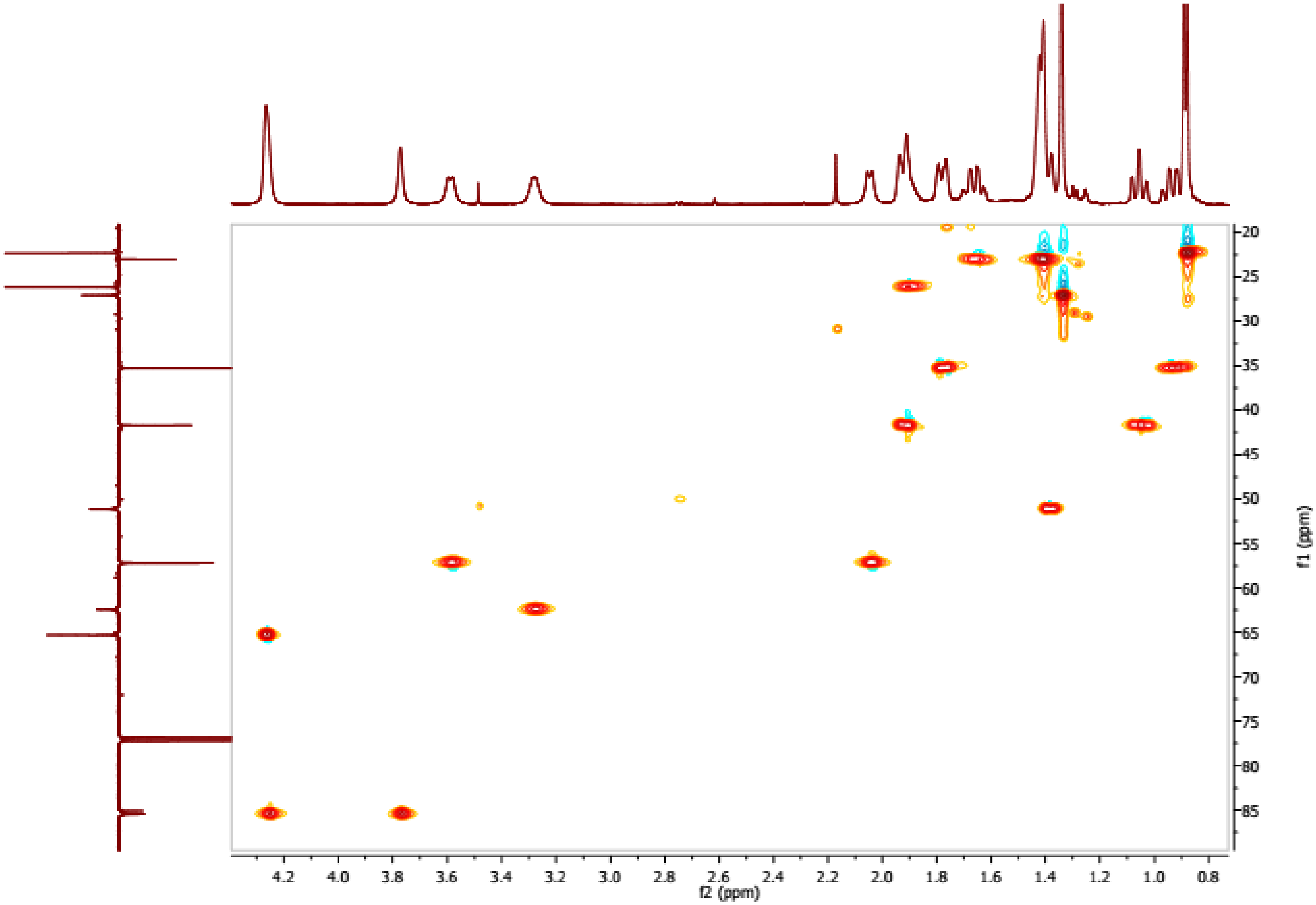

HMBC of compound **27**

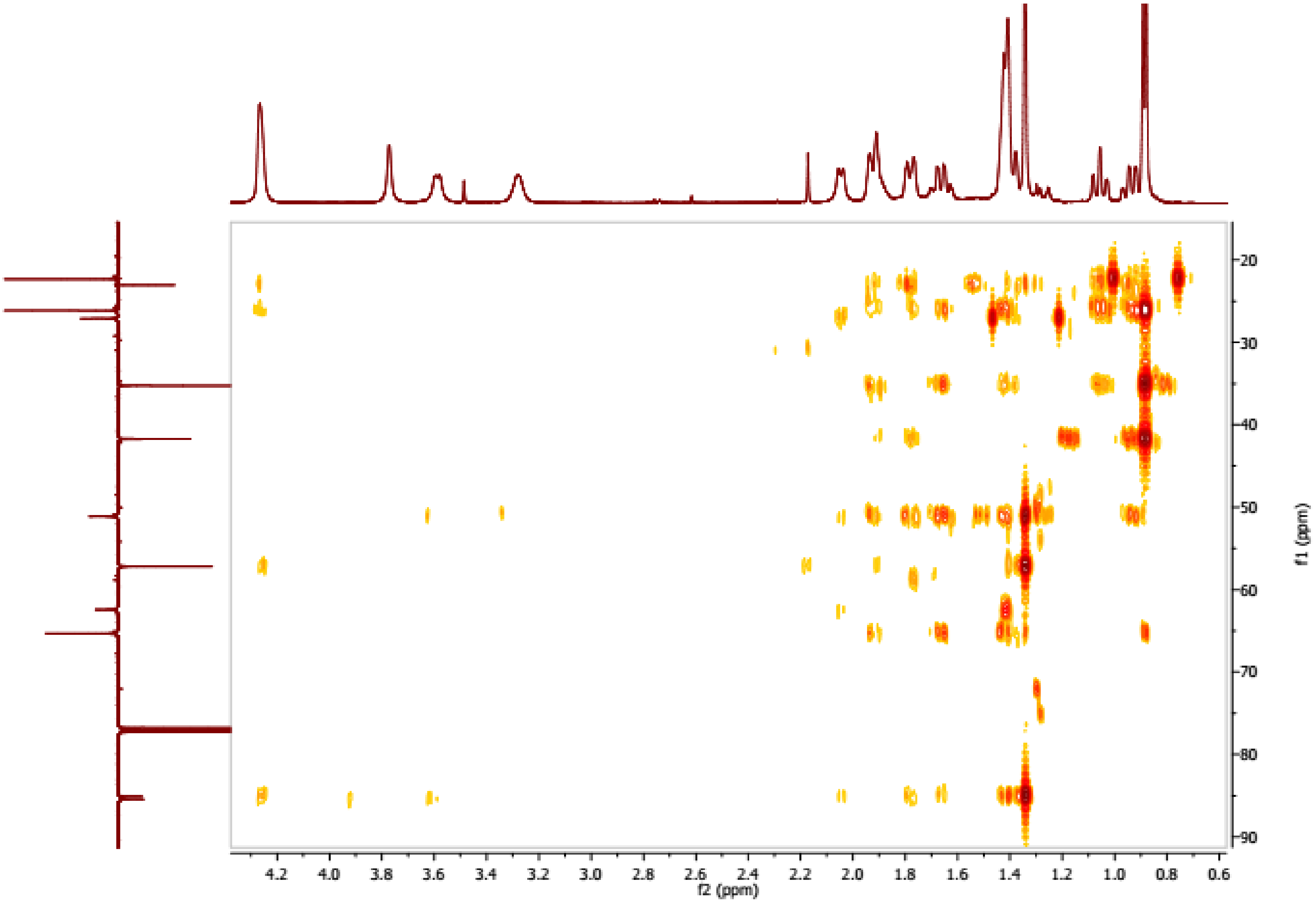

^{1}H-NMR of compound **28**

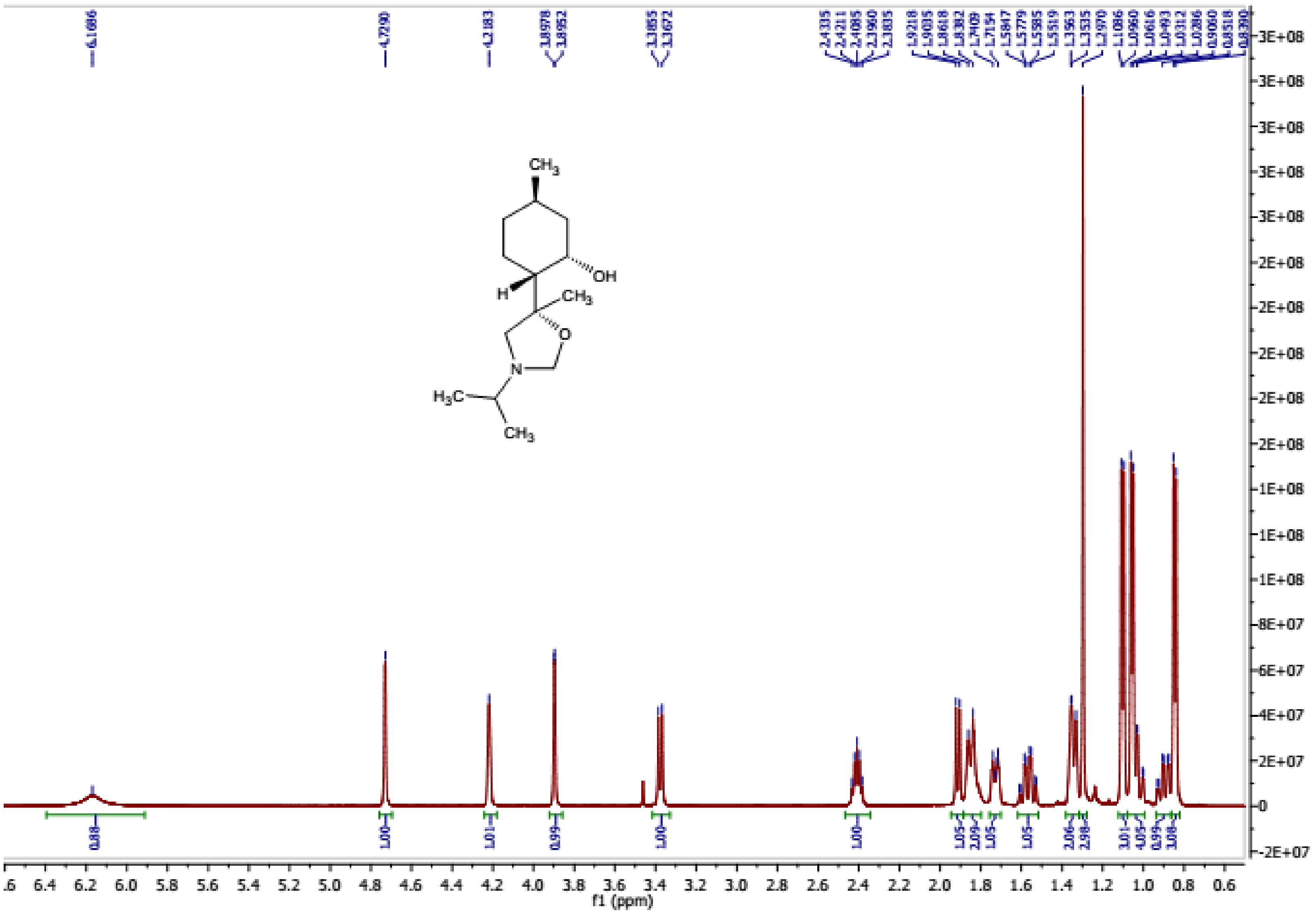

^{13}C-NMR of compound **28**

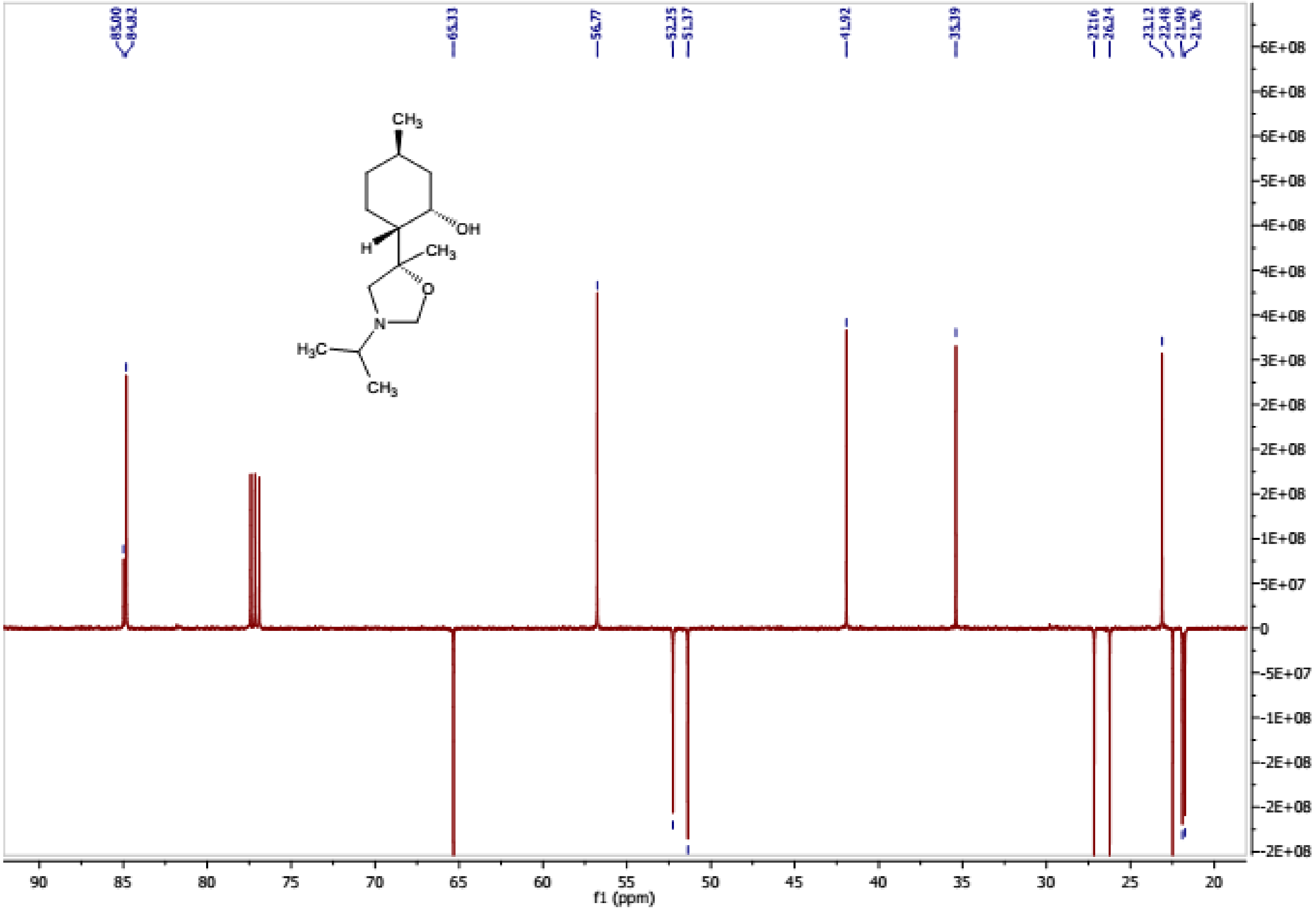

HSQC of compound **28**

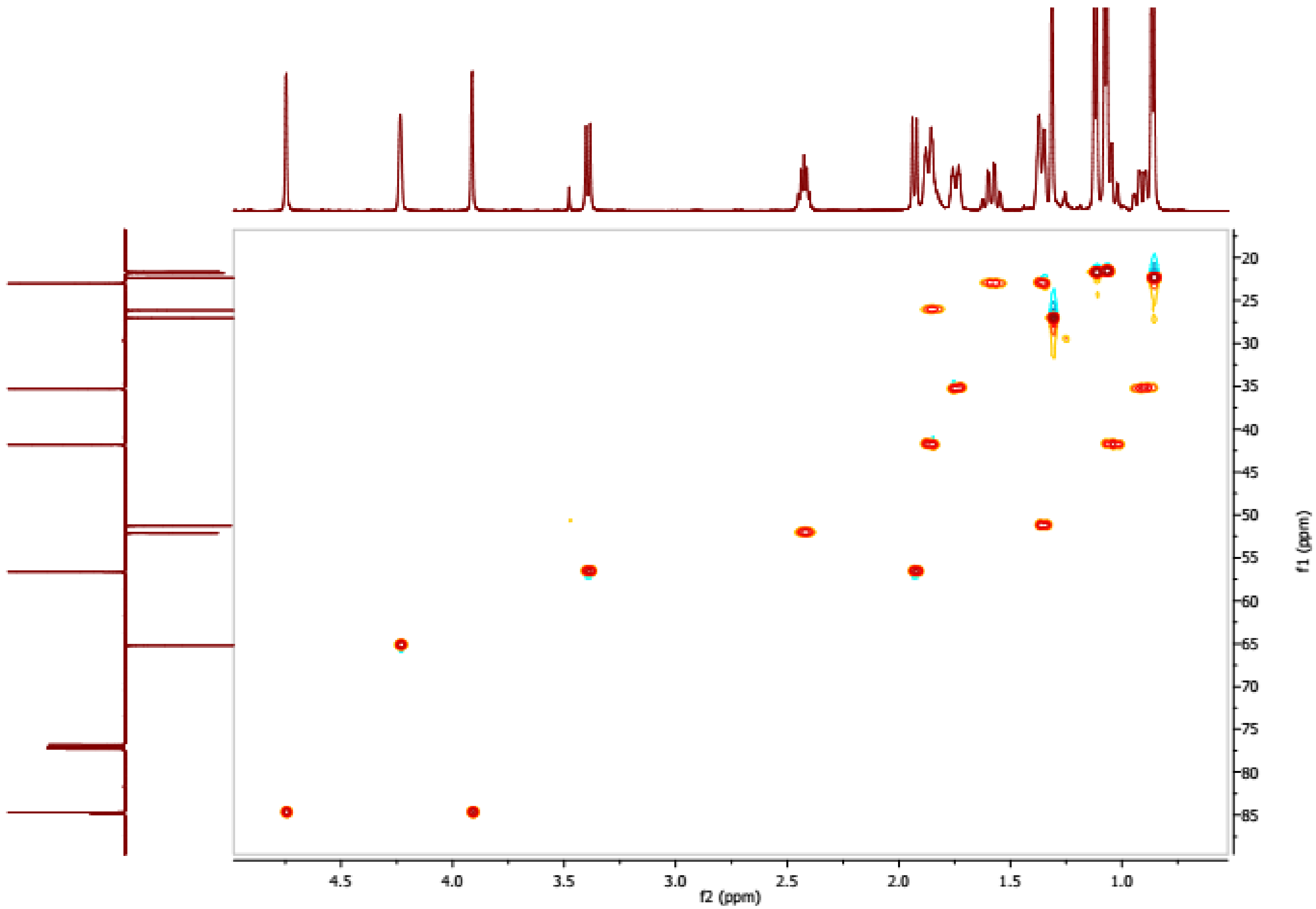

HMBC of compound **28**

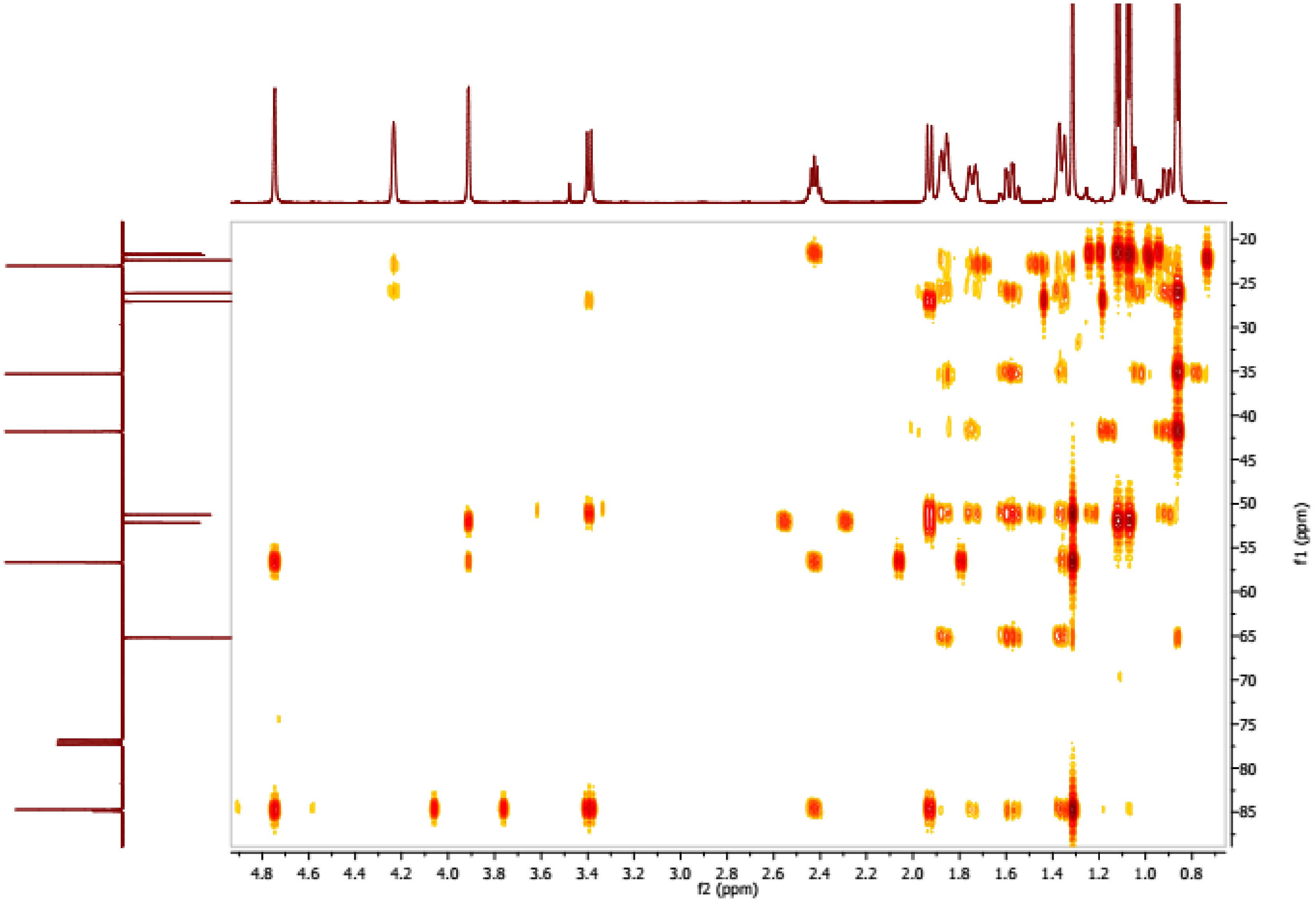

^{1}H-NMR of compound **29**

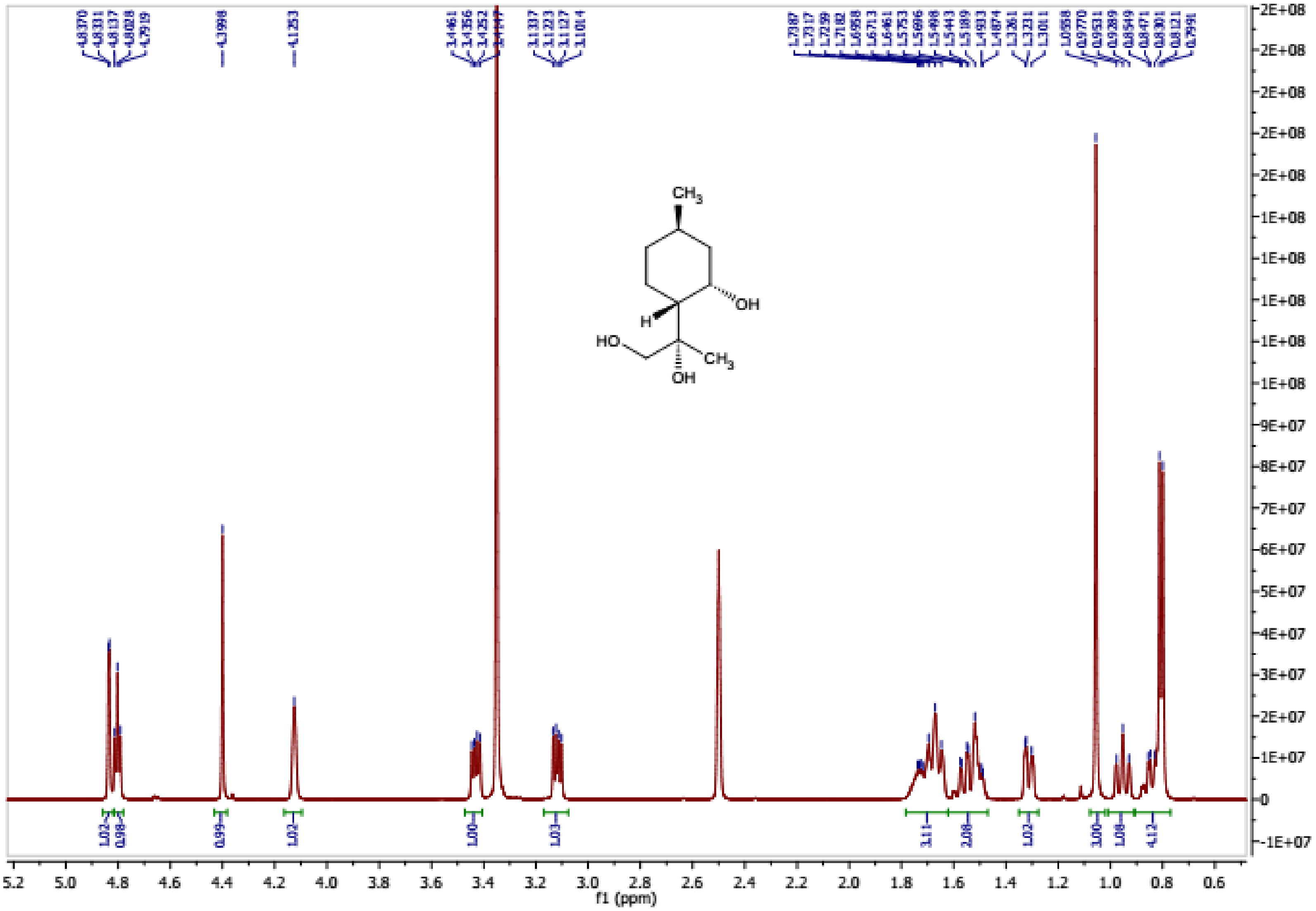

^{13}C-NMR of compound **29**

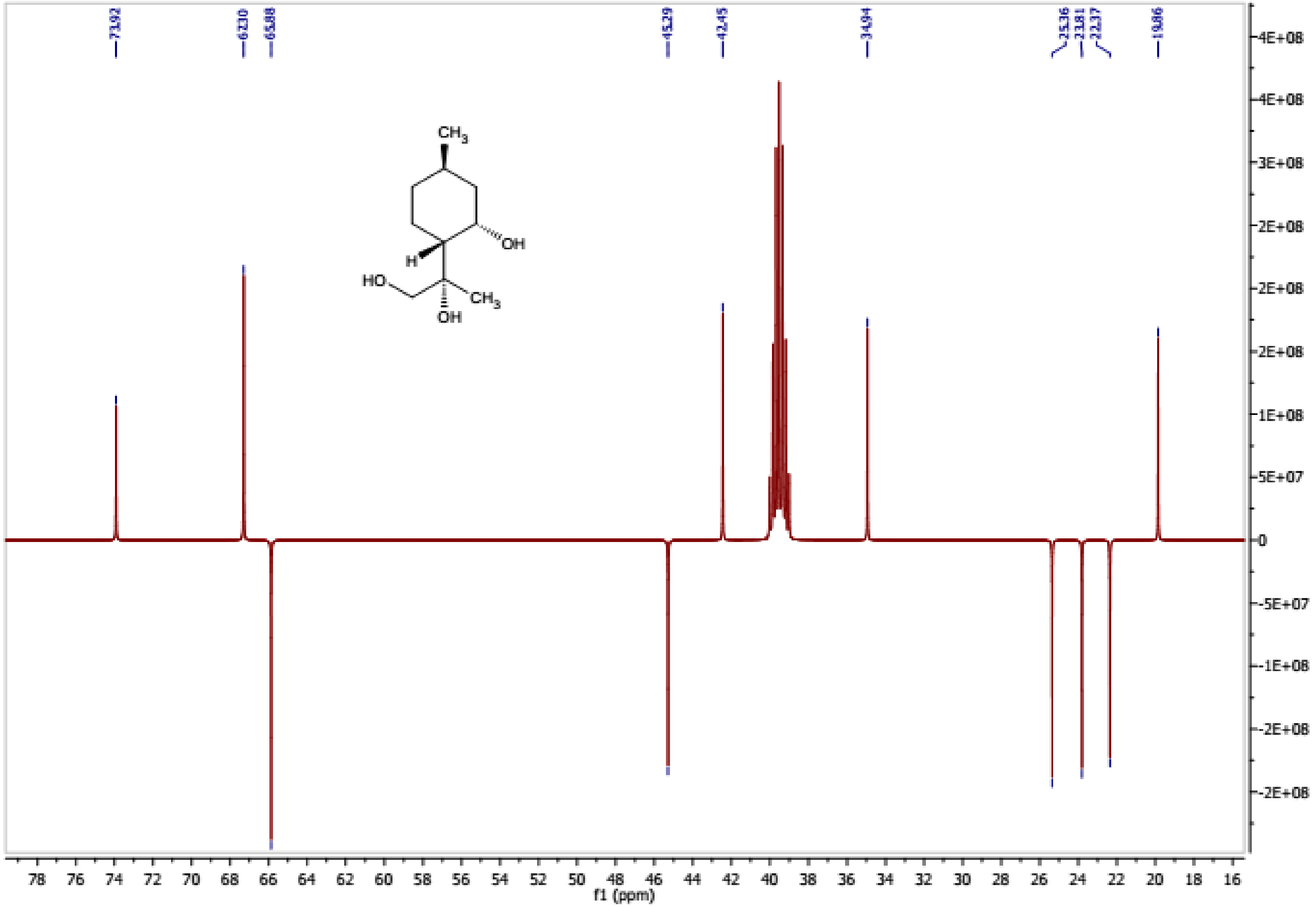

COSY of compound **29**

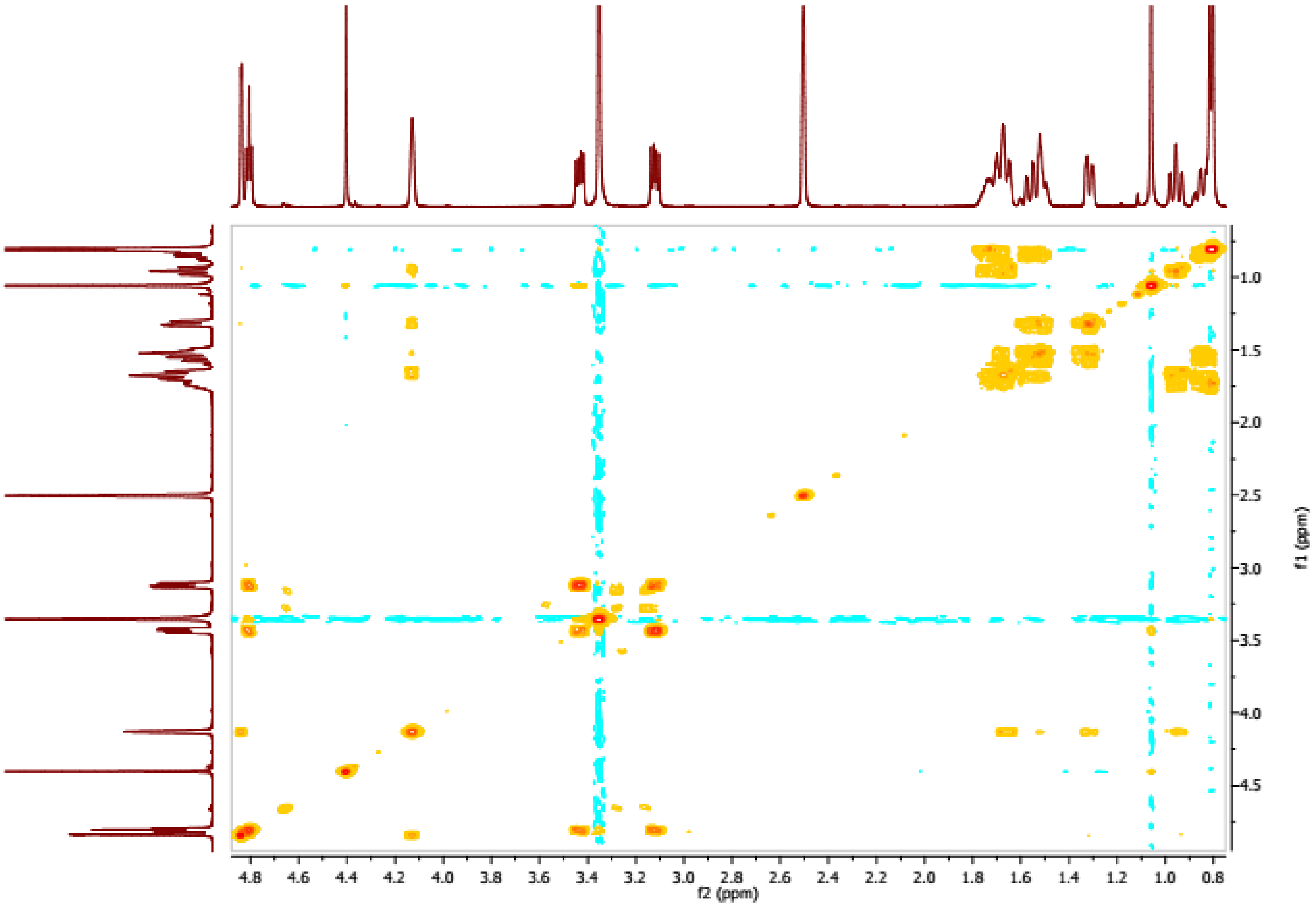

NOESY of compound **29**

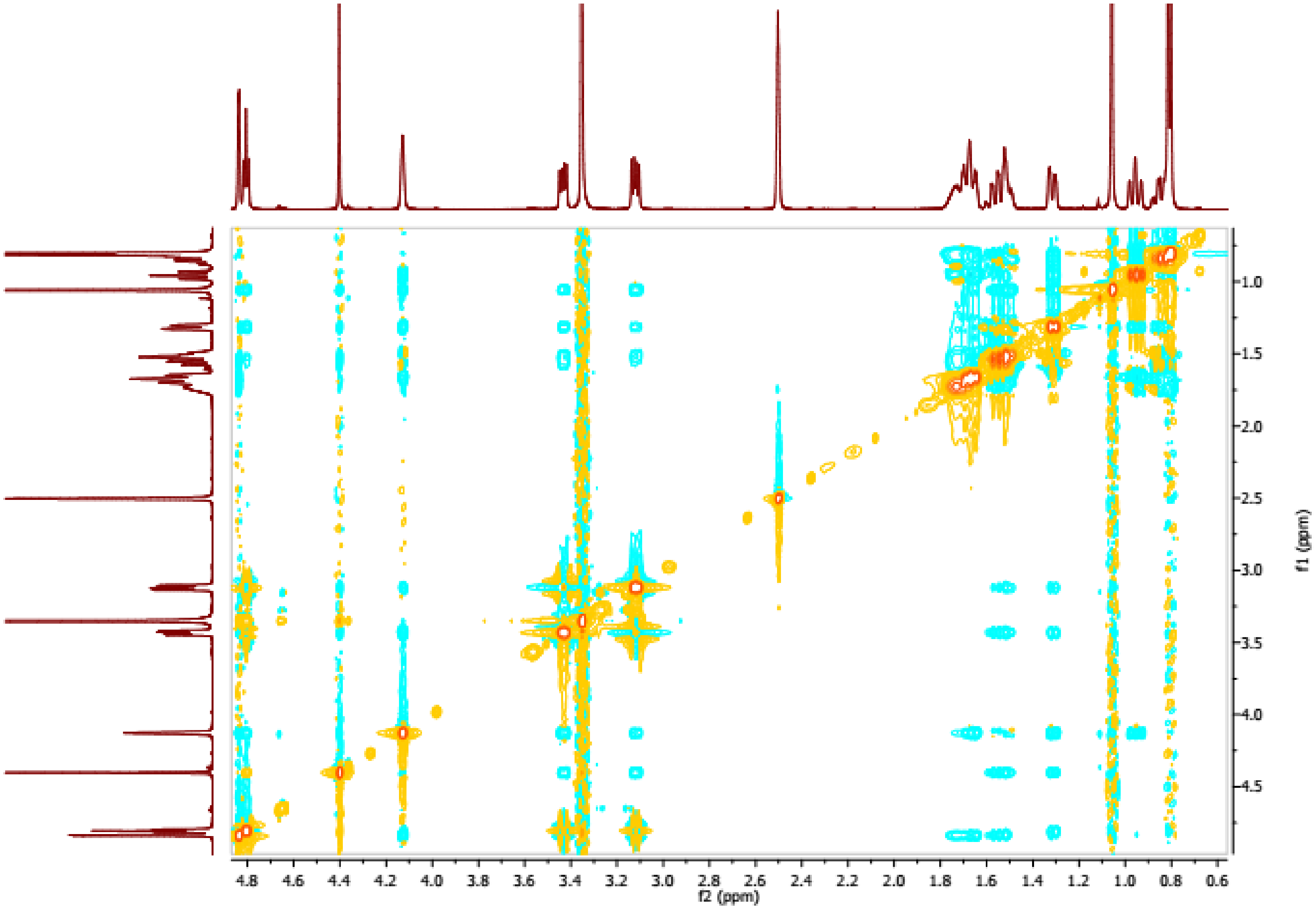

HSQC of compound **29**

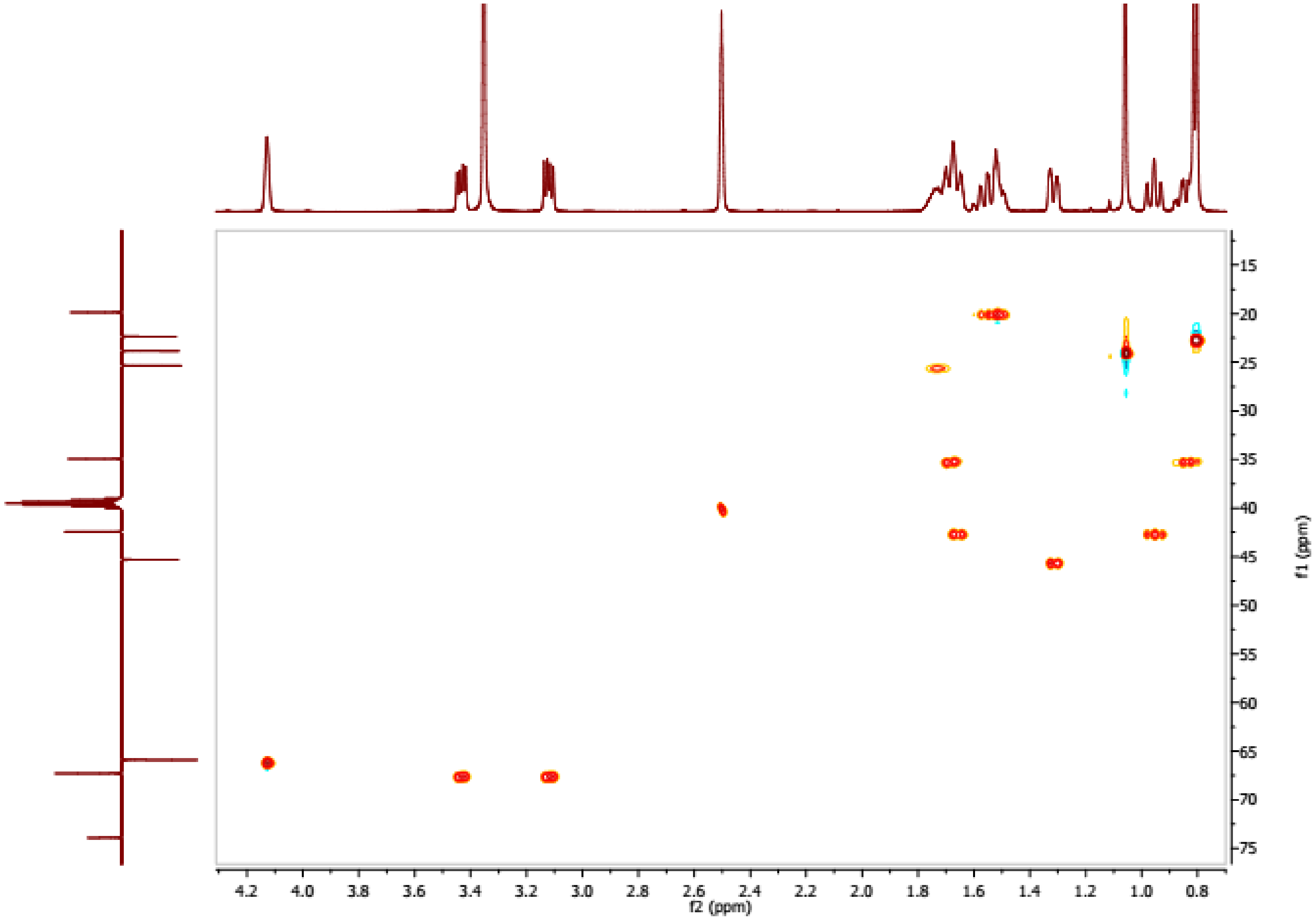

HMBC of compound **29**

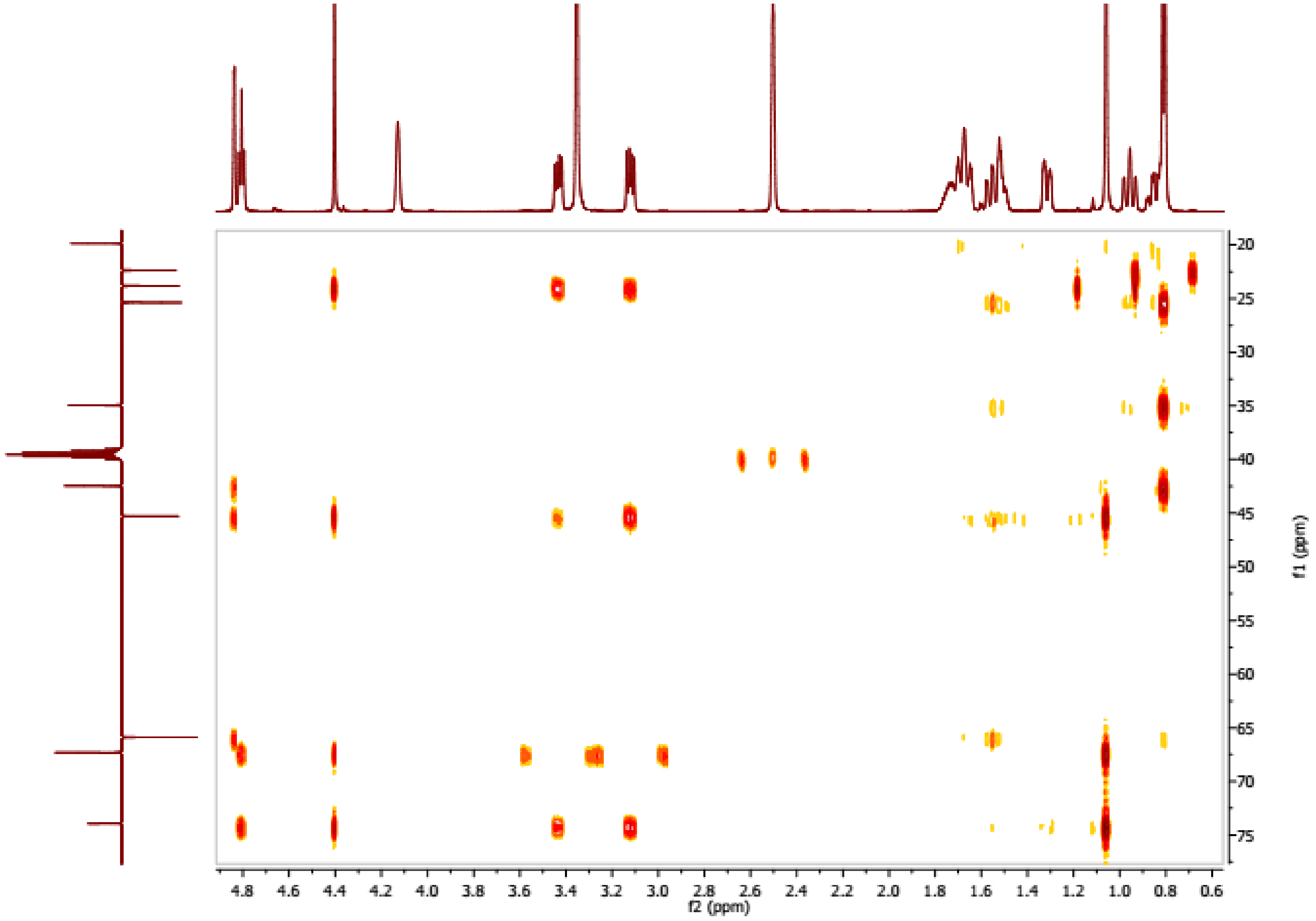

^{1}H-NMR of compound **30**

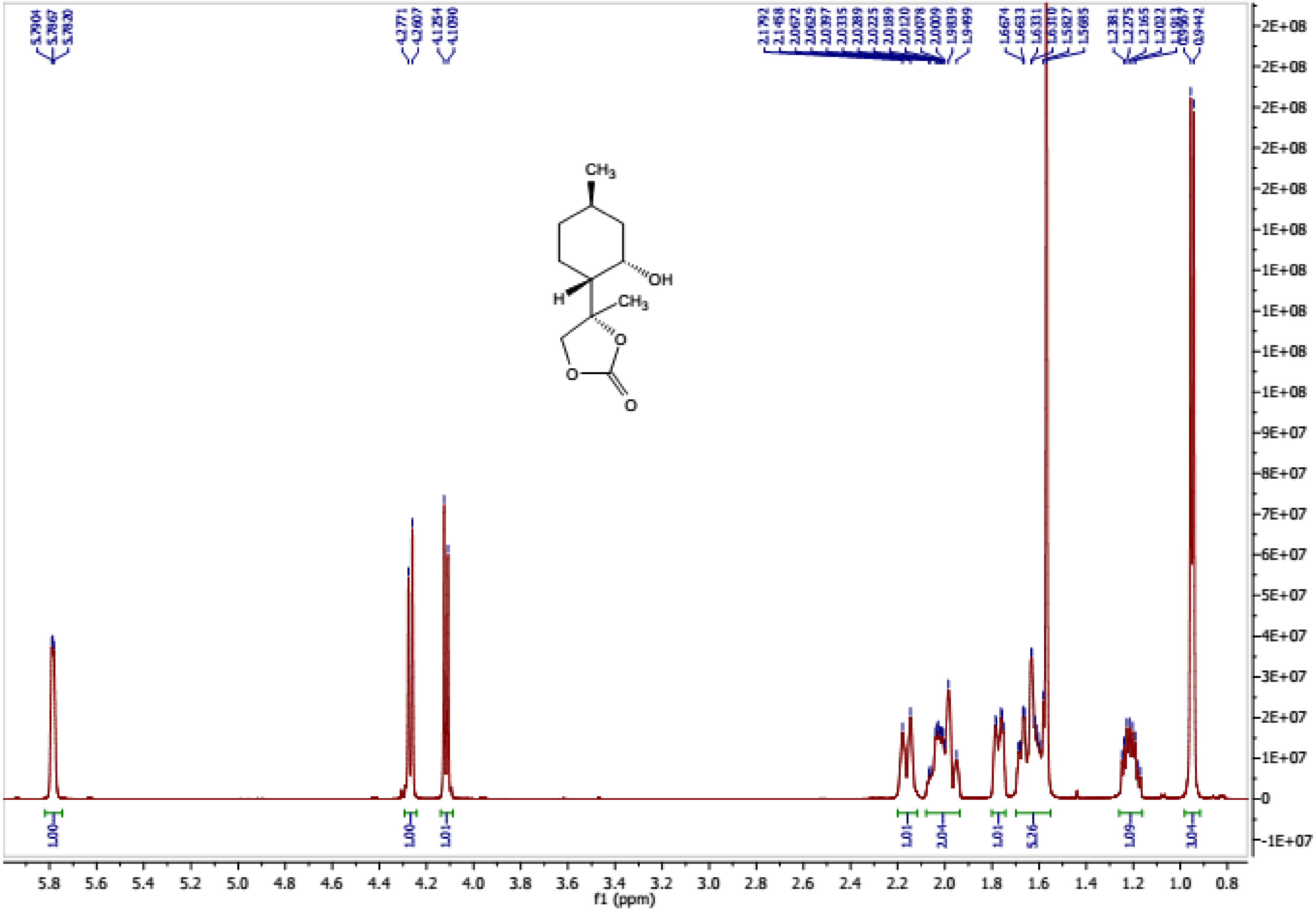

^{13}C-NMR of compound **30**

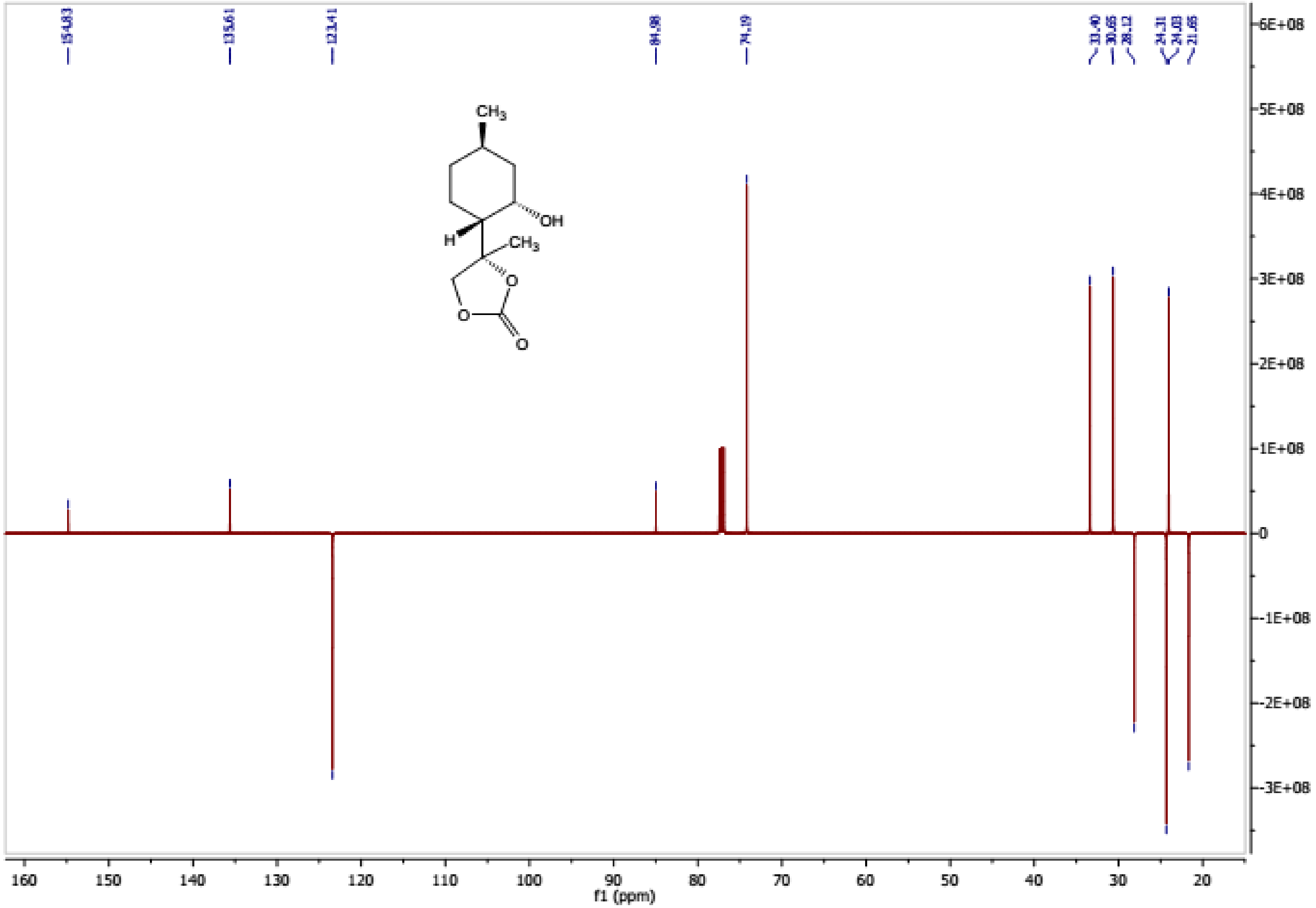

COSY of compound **30**

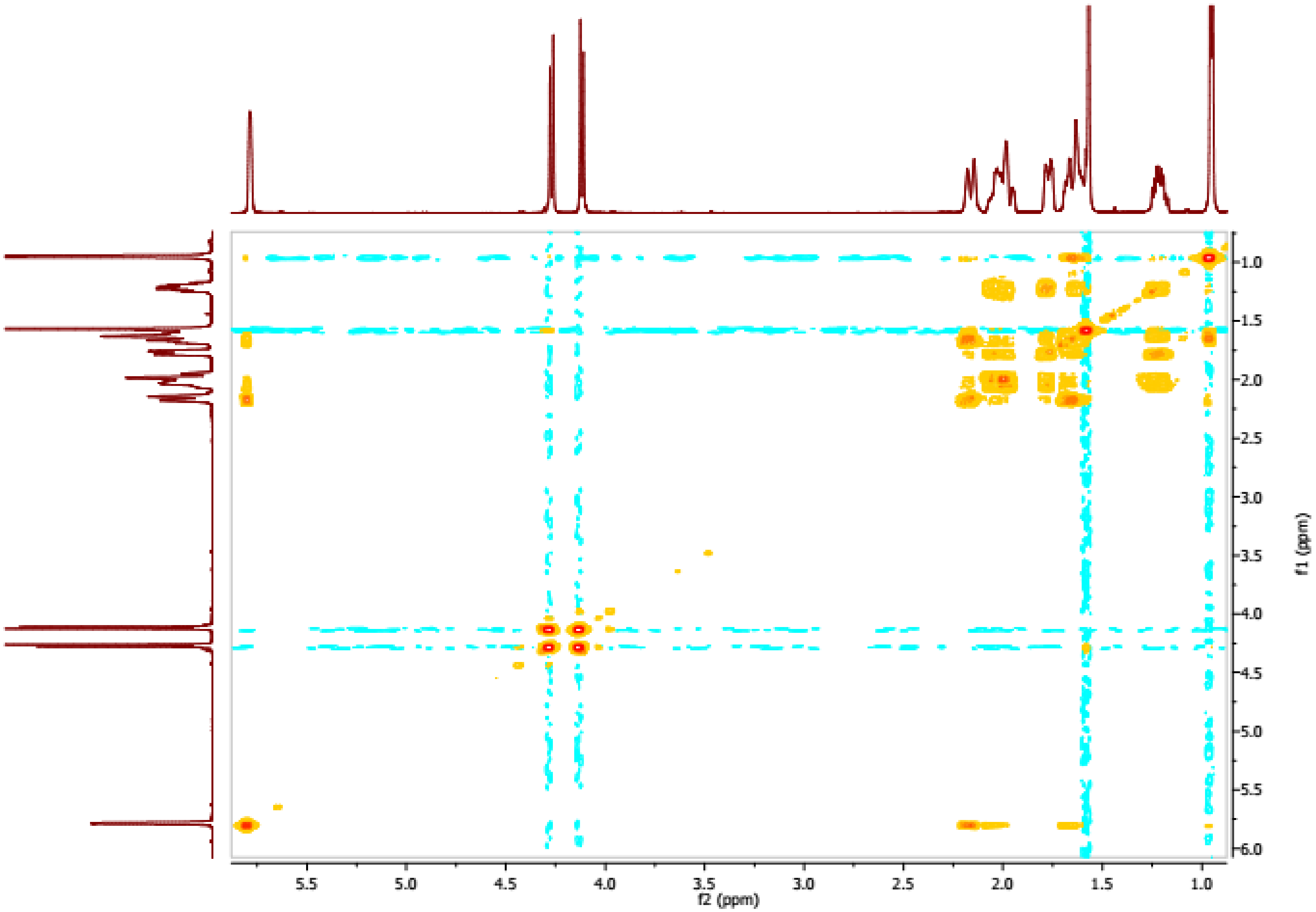

NOESY of compound **30**

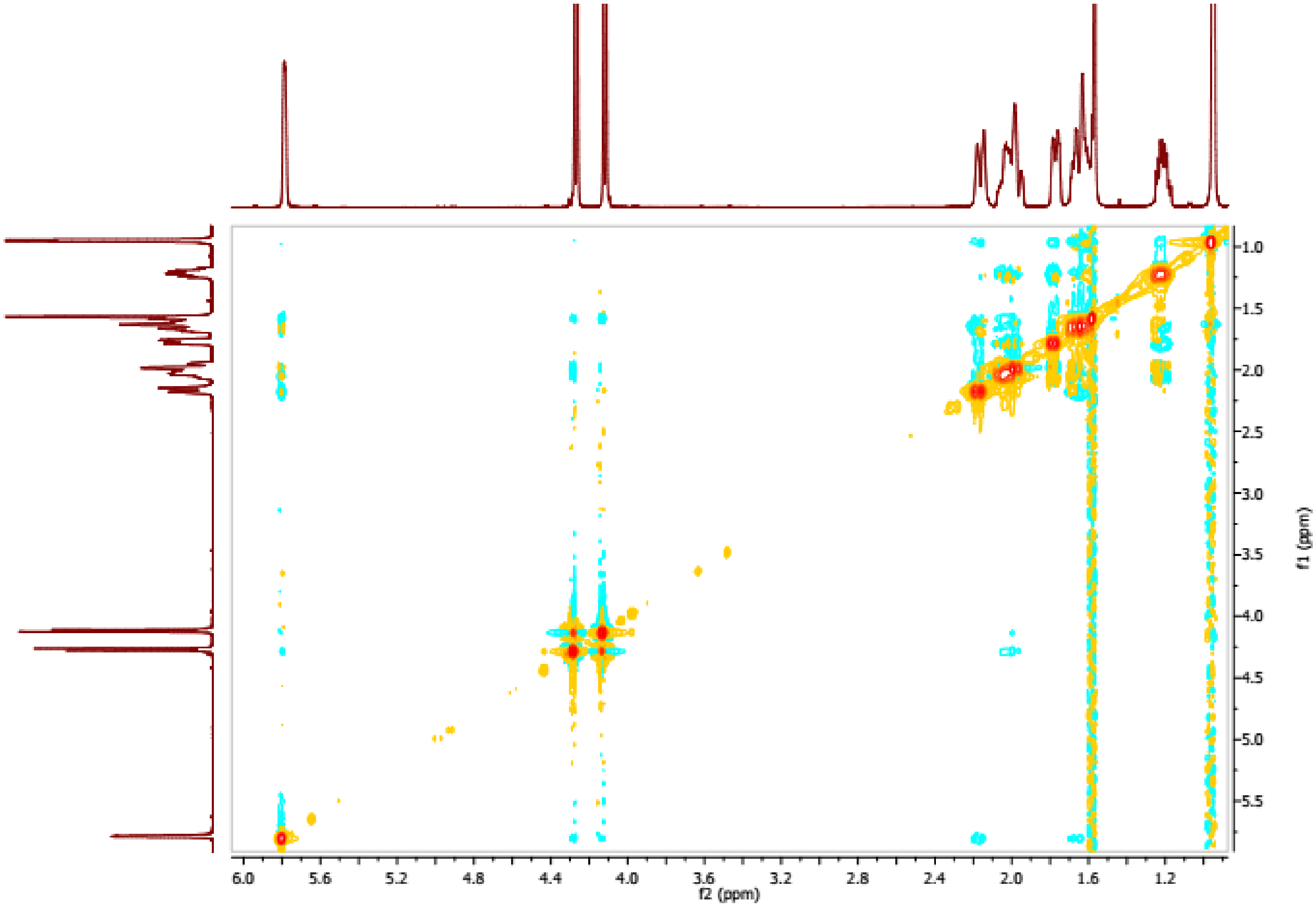

HSQC of compound **30**

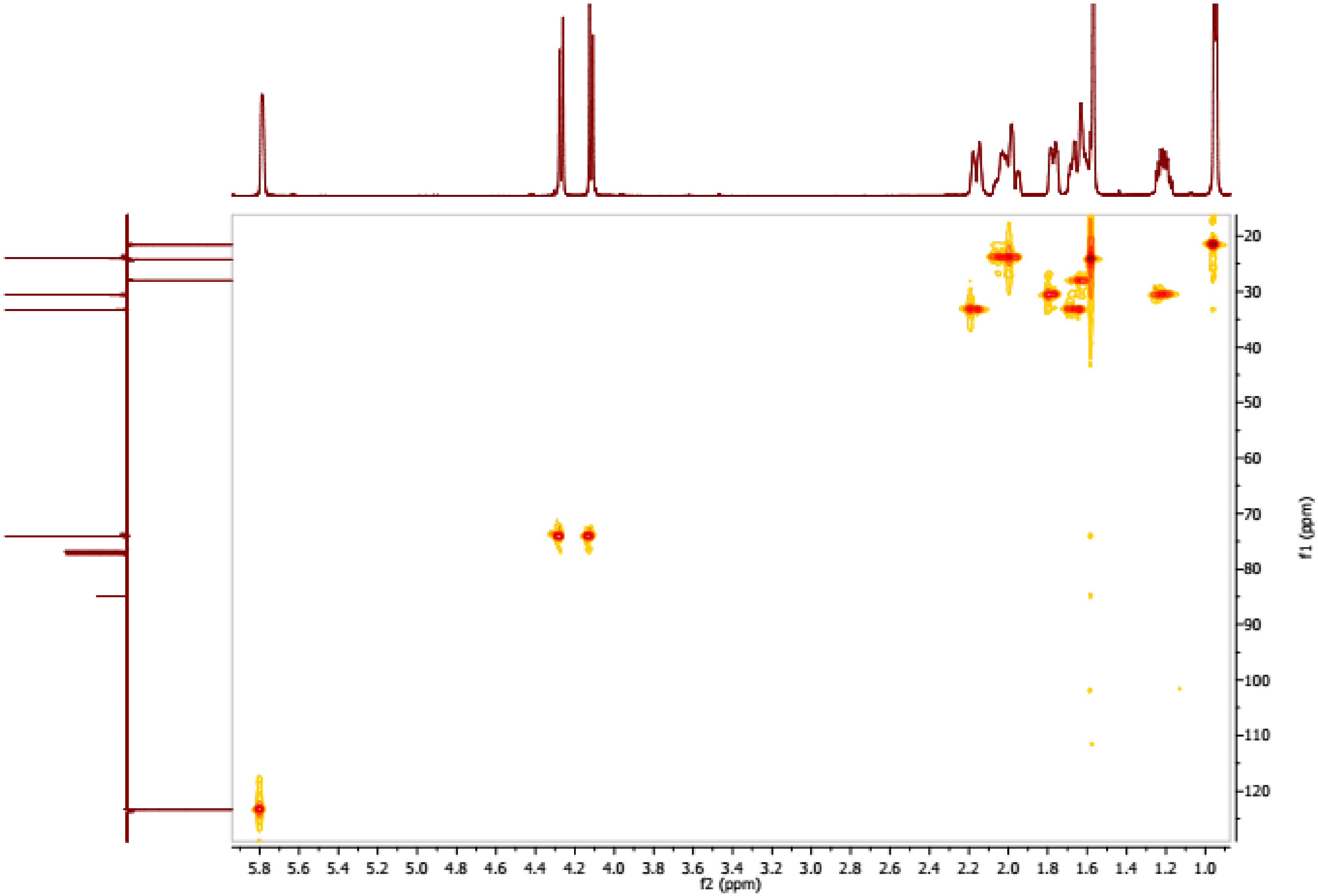

HMBC of compound **30**

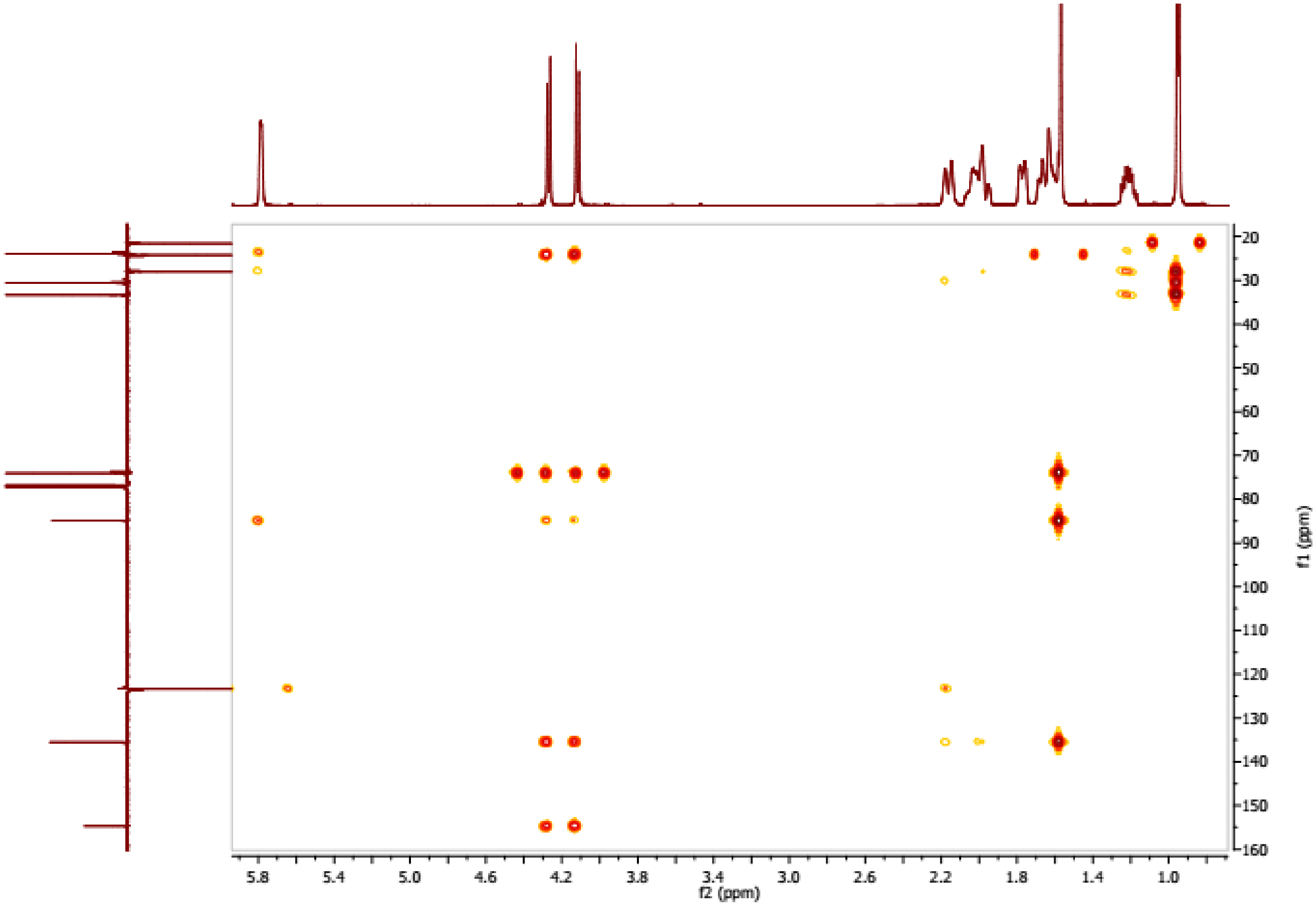

^{1}H-NMR of compound **31**

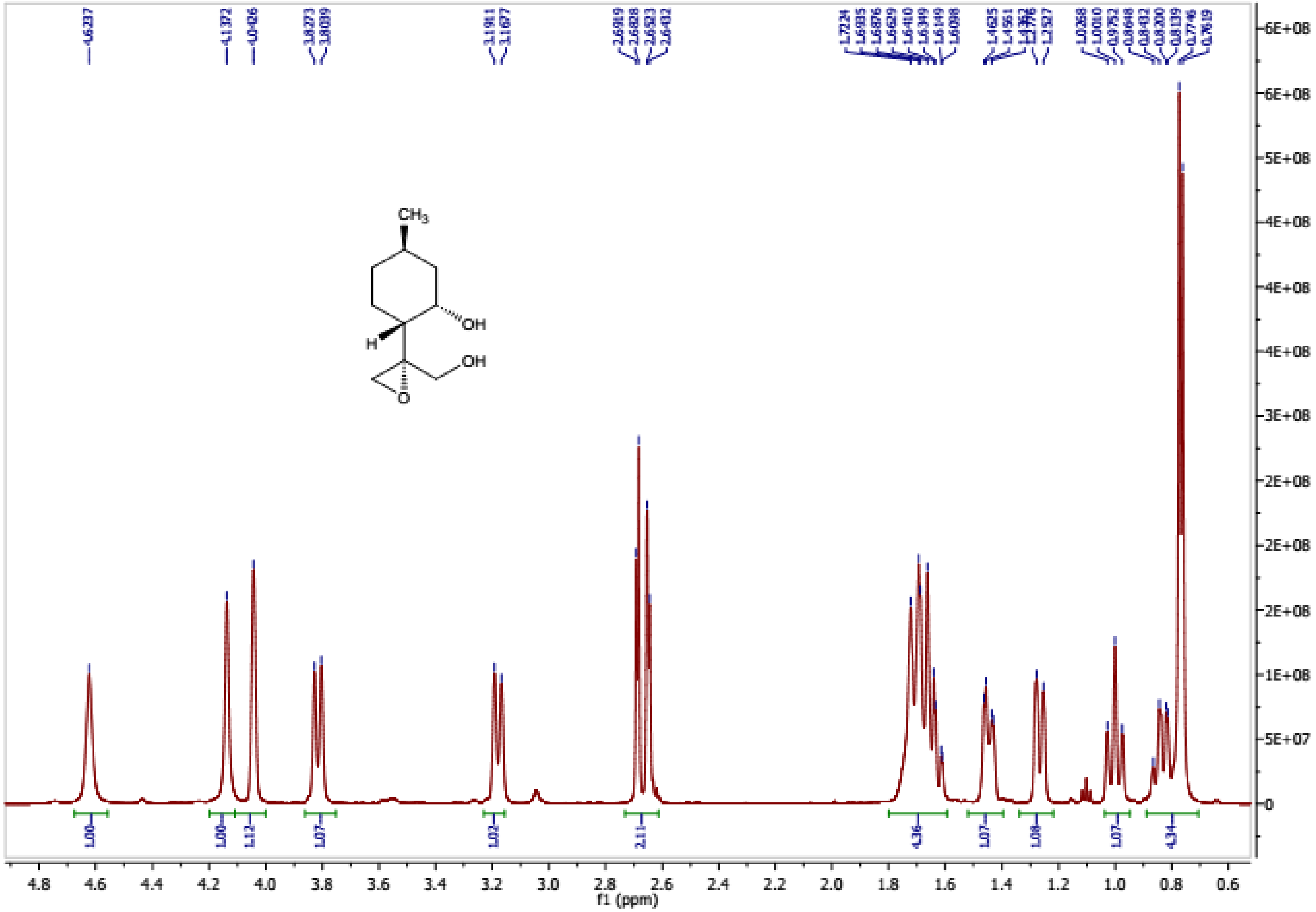

^{13}C-NMR of compound **31**

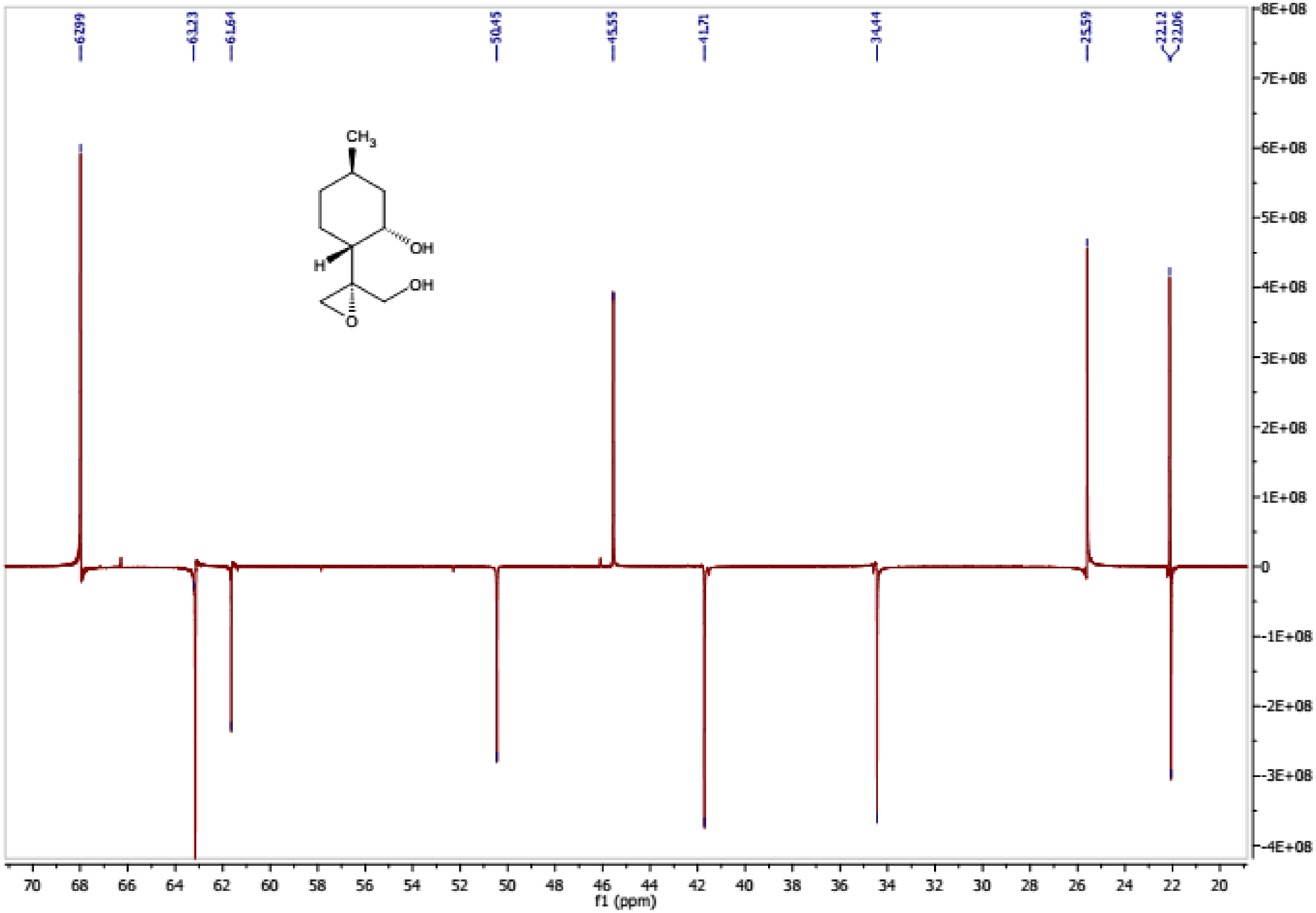

COSY os compound **31**

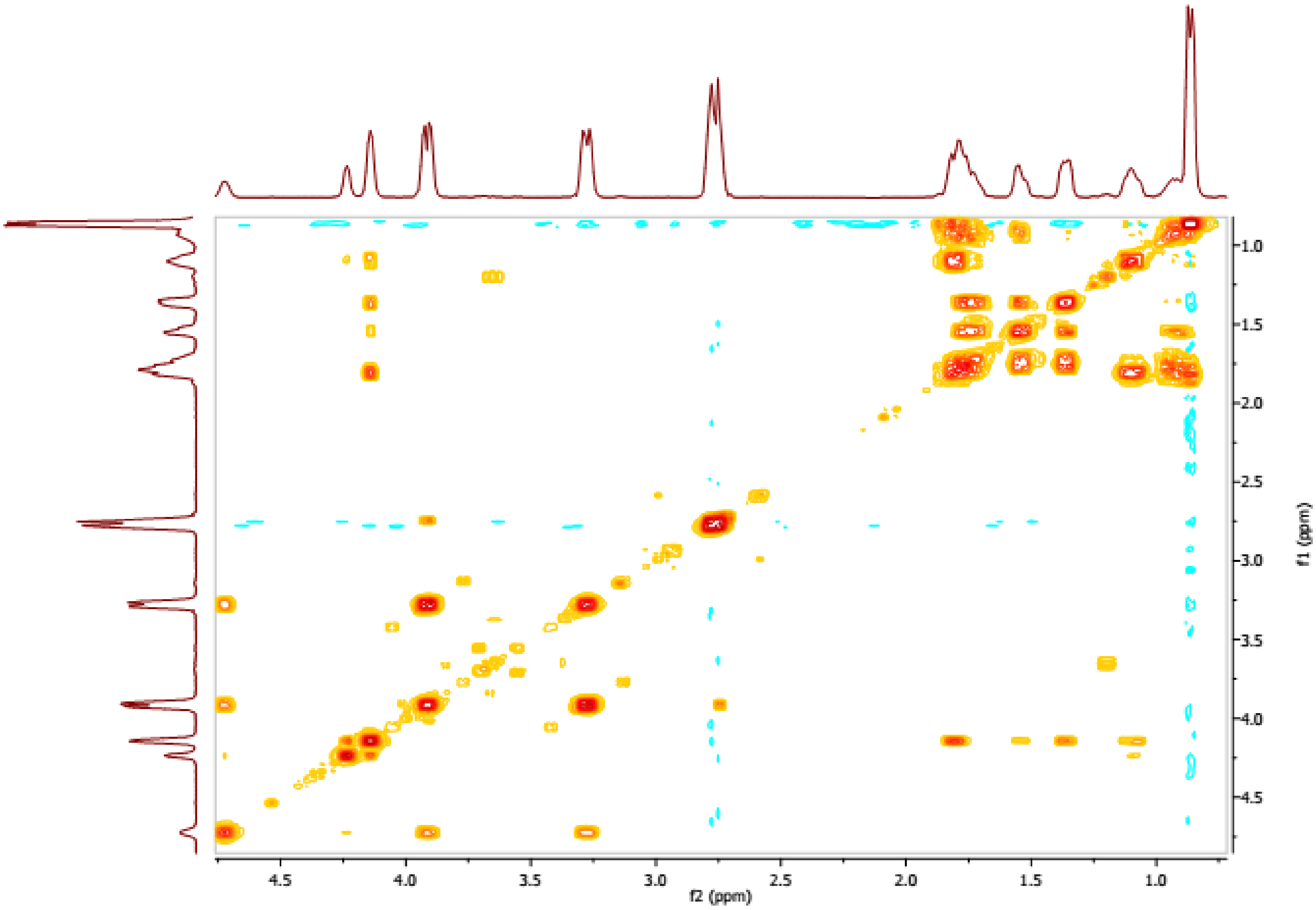

NOESY of compound **31**

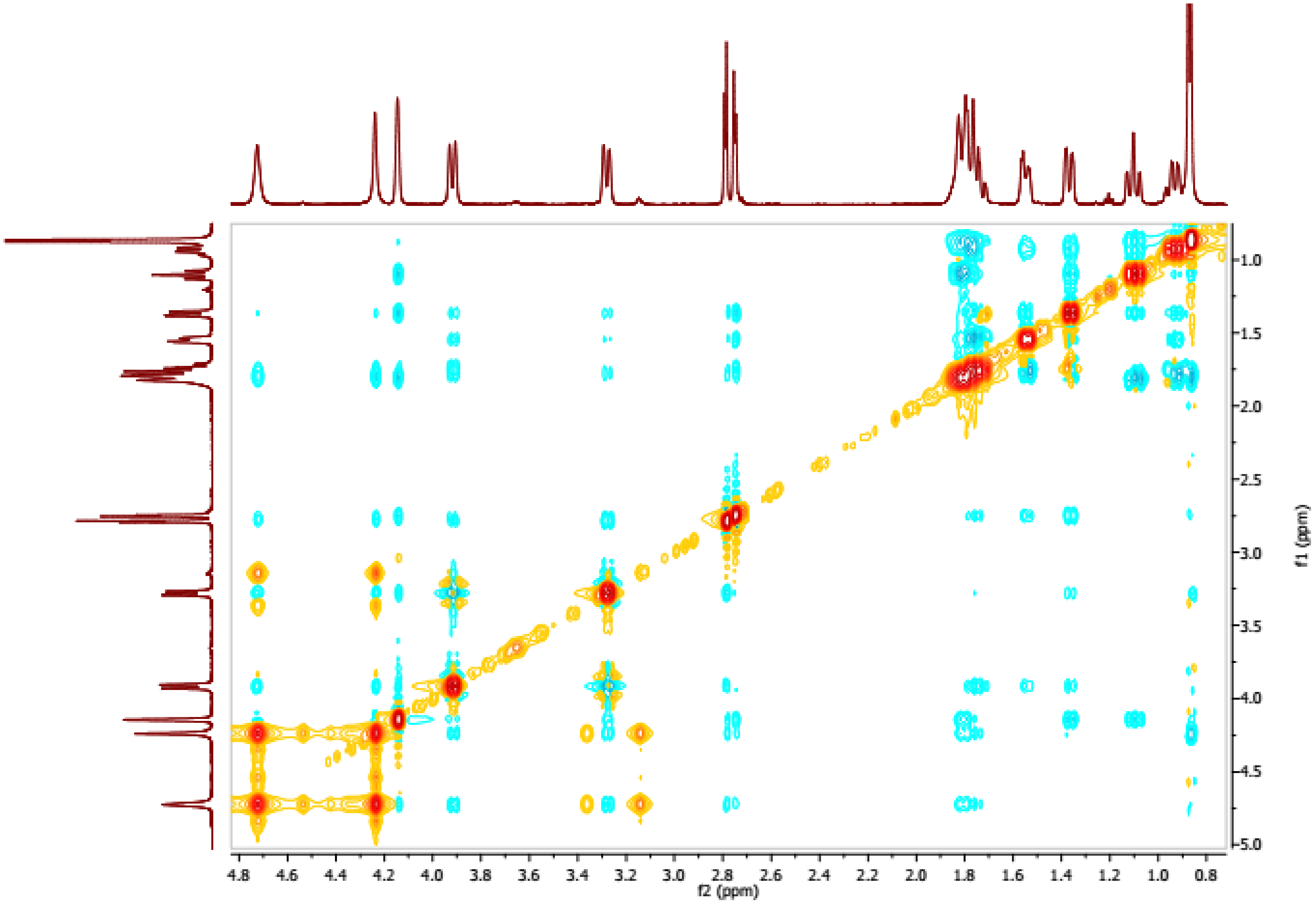

HSQC of compound **31**

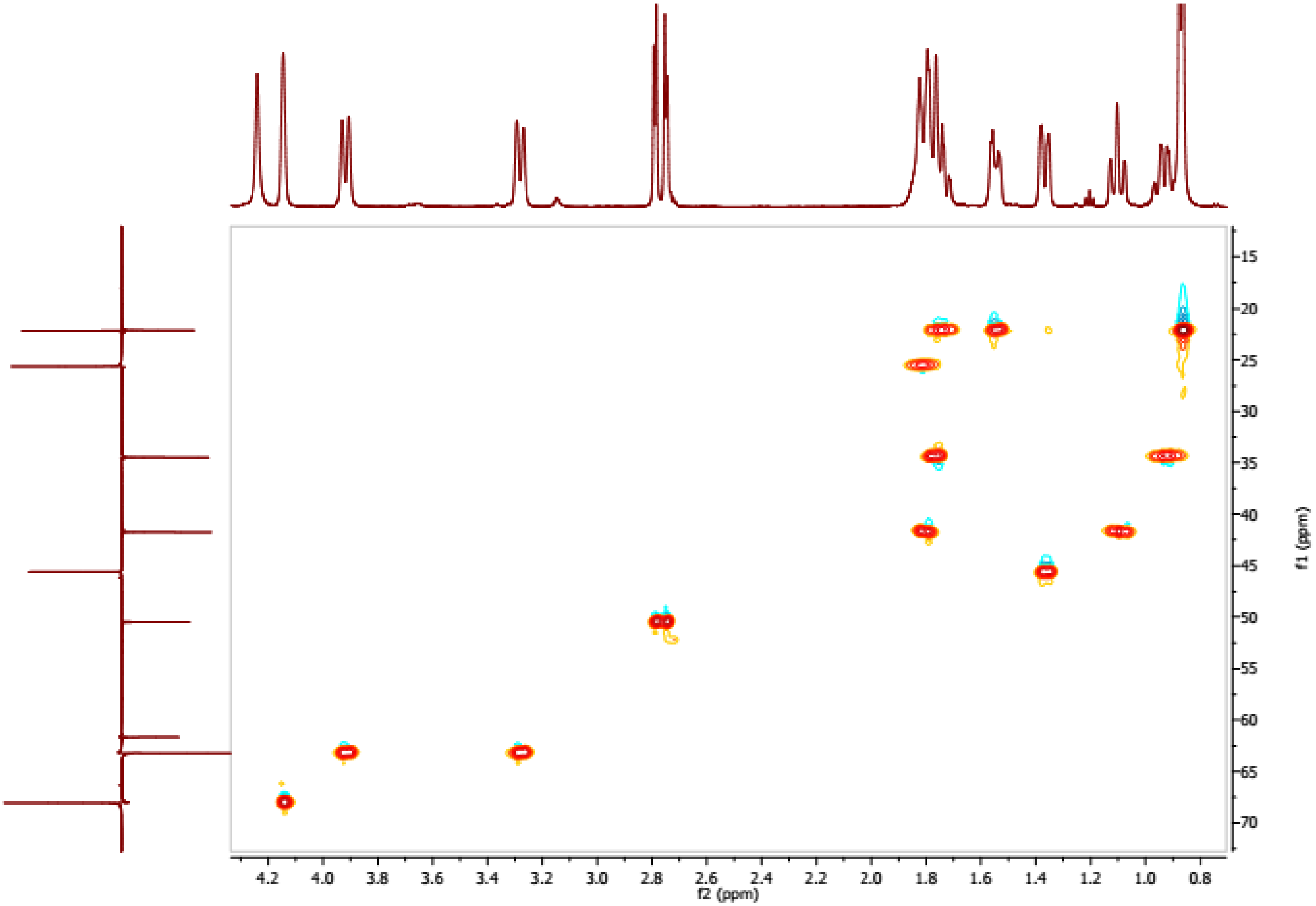

HMBC of compound **31**

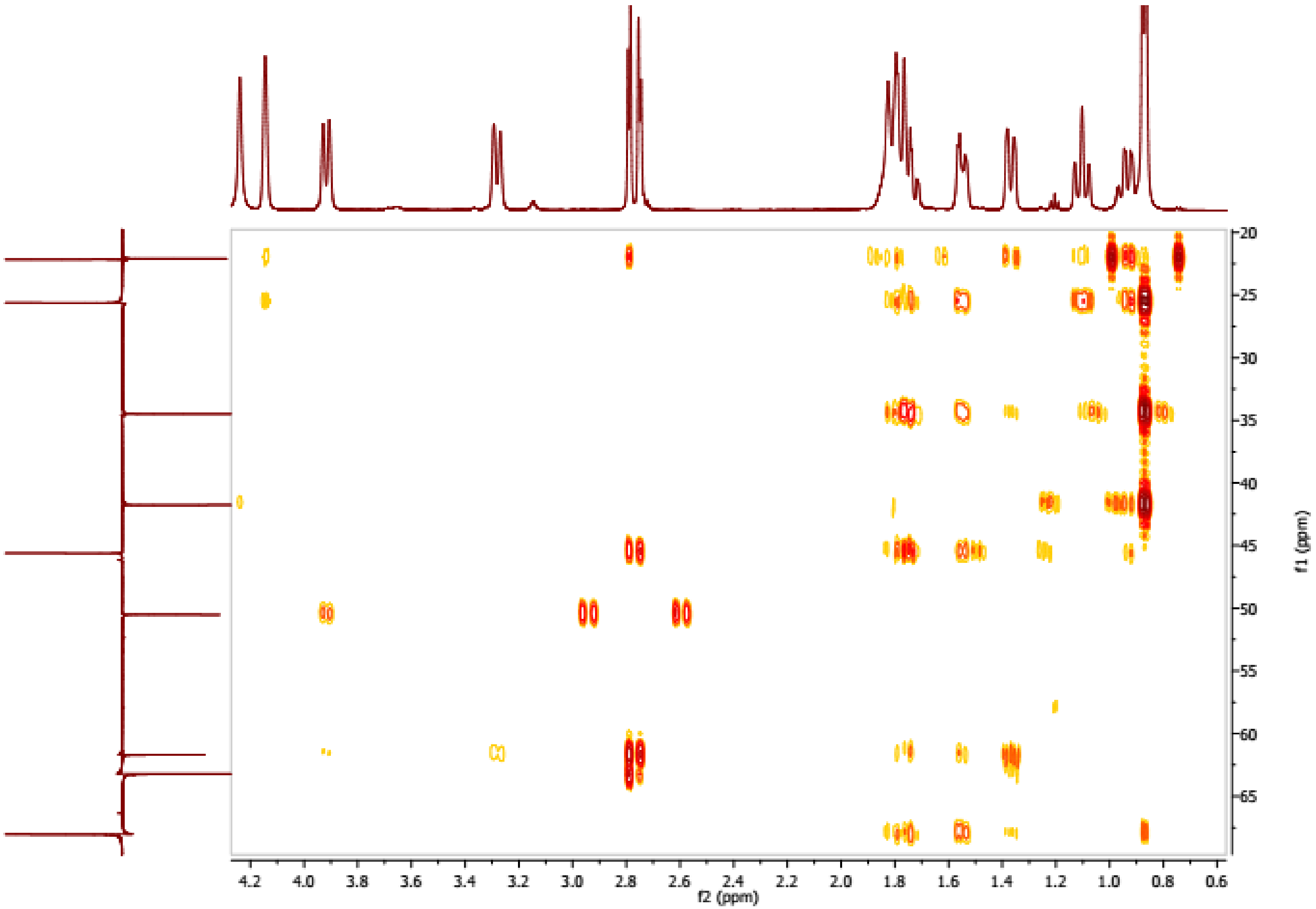

^{1}H-NMR of compound **32**

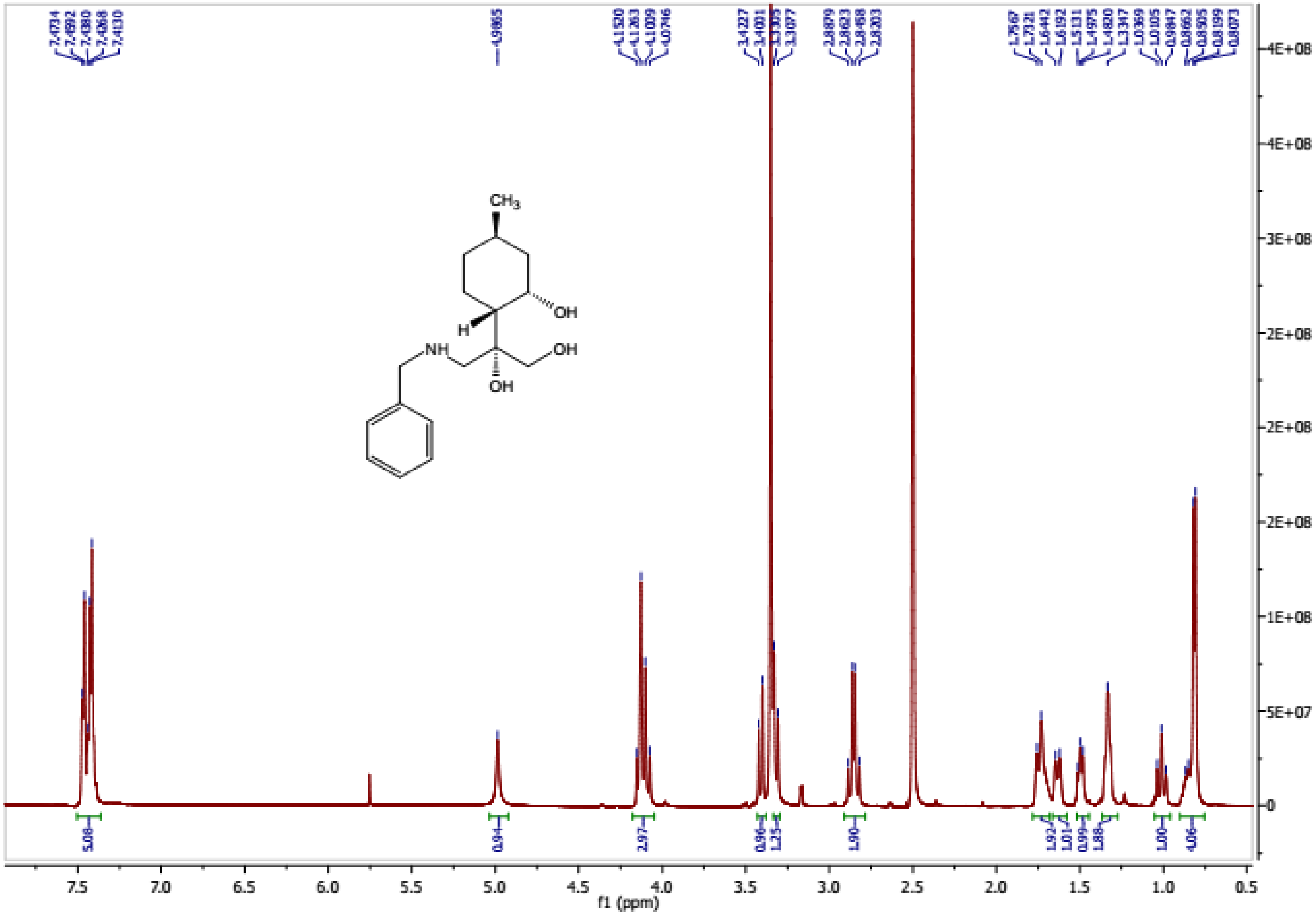

^{13}C-NMR of compound **32**

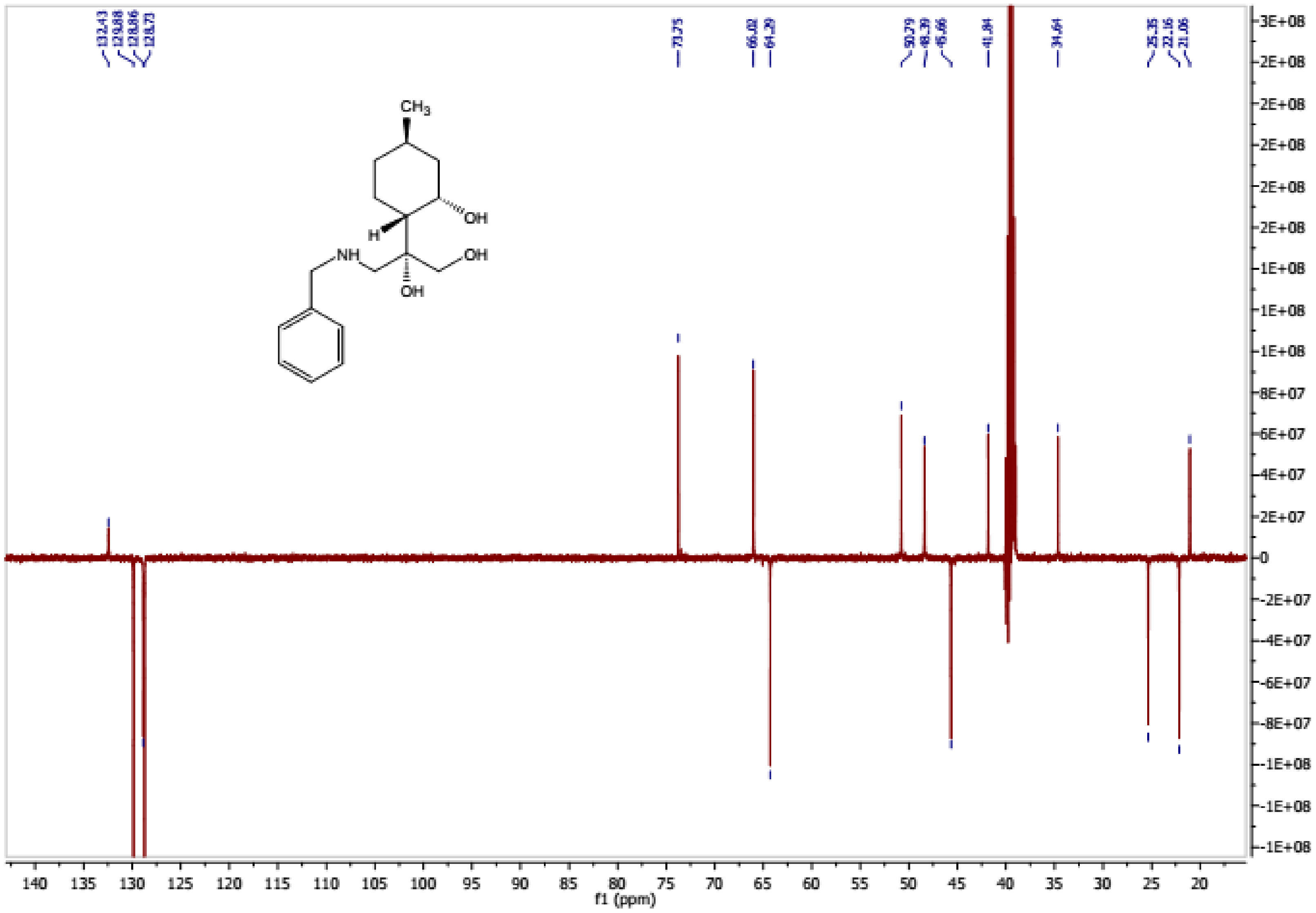

COSY of compound **32**

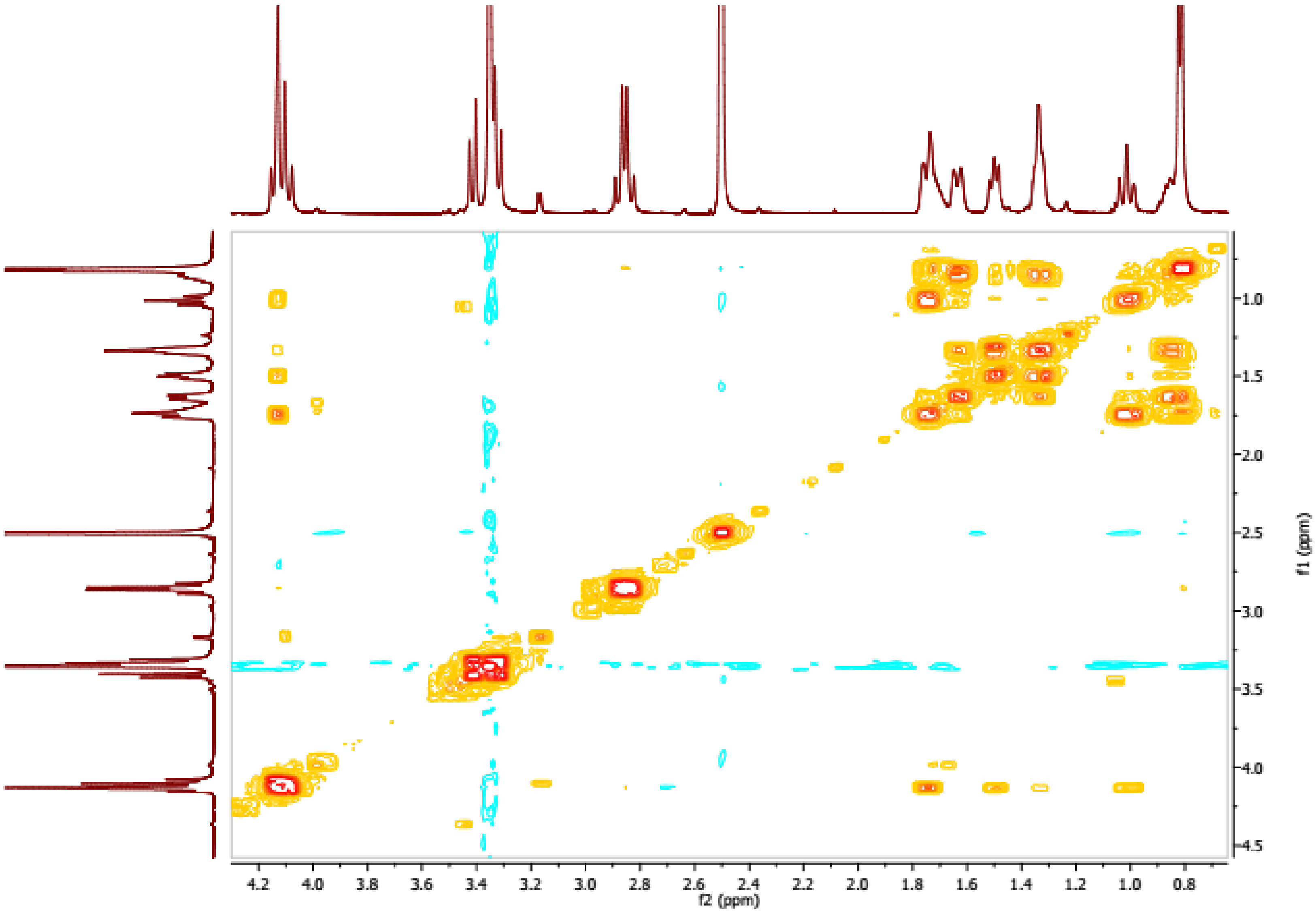

NOESY of compound **32**

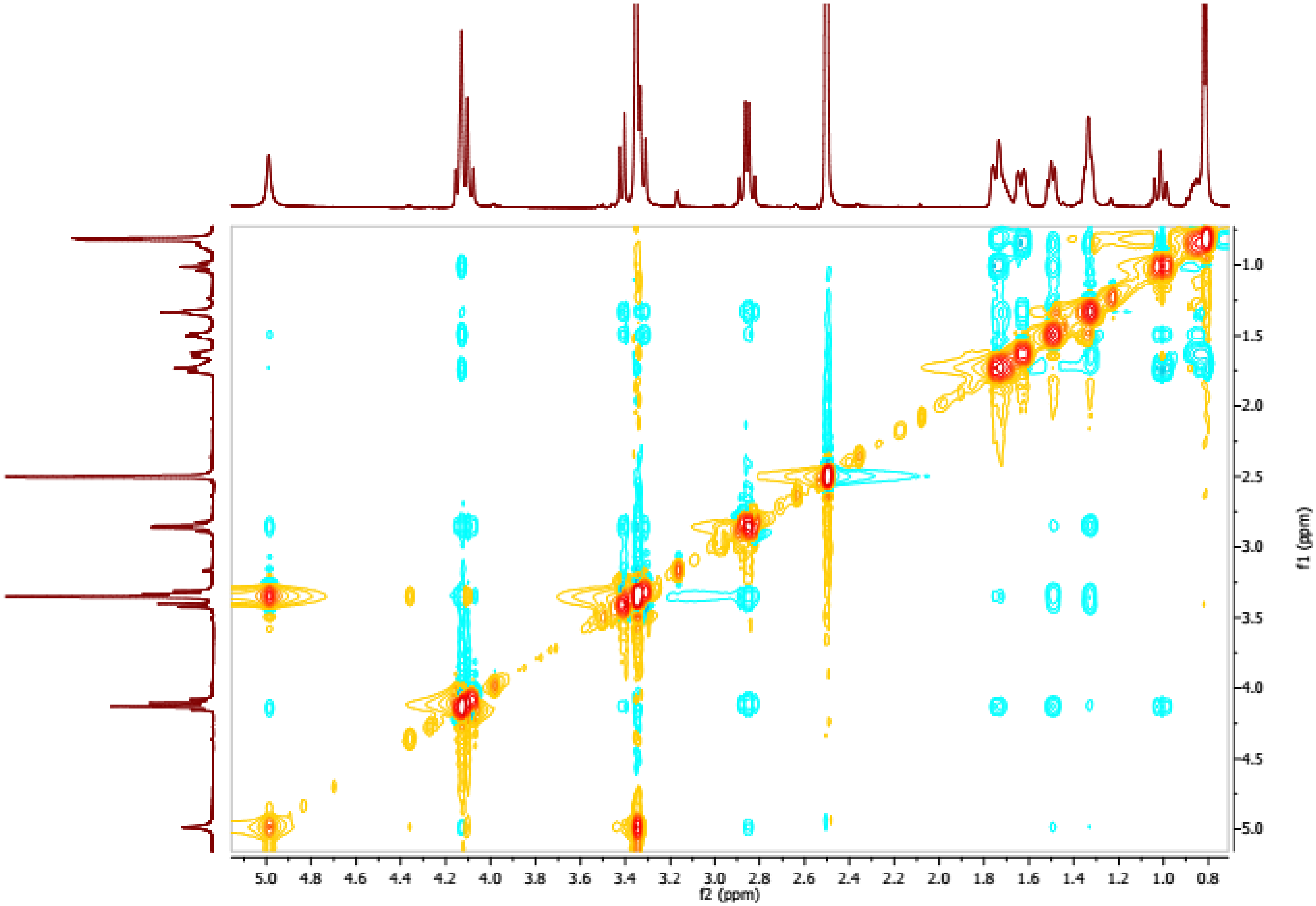

HSQC of compound **32**

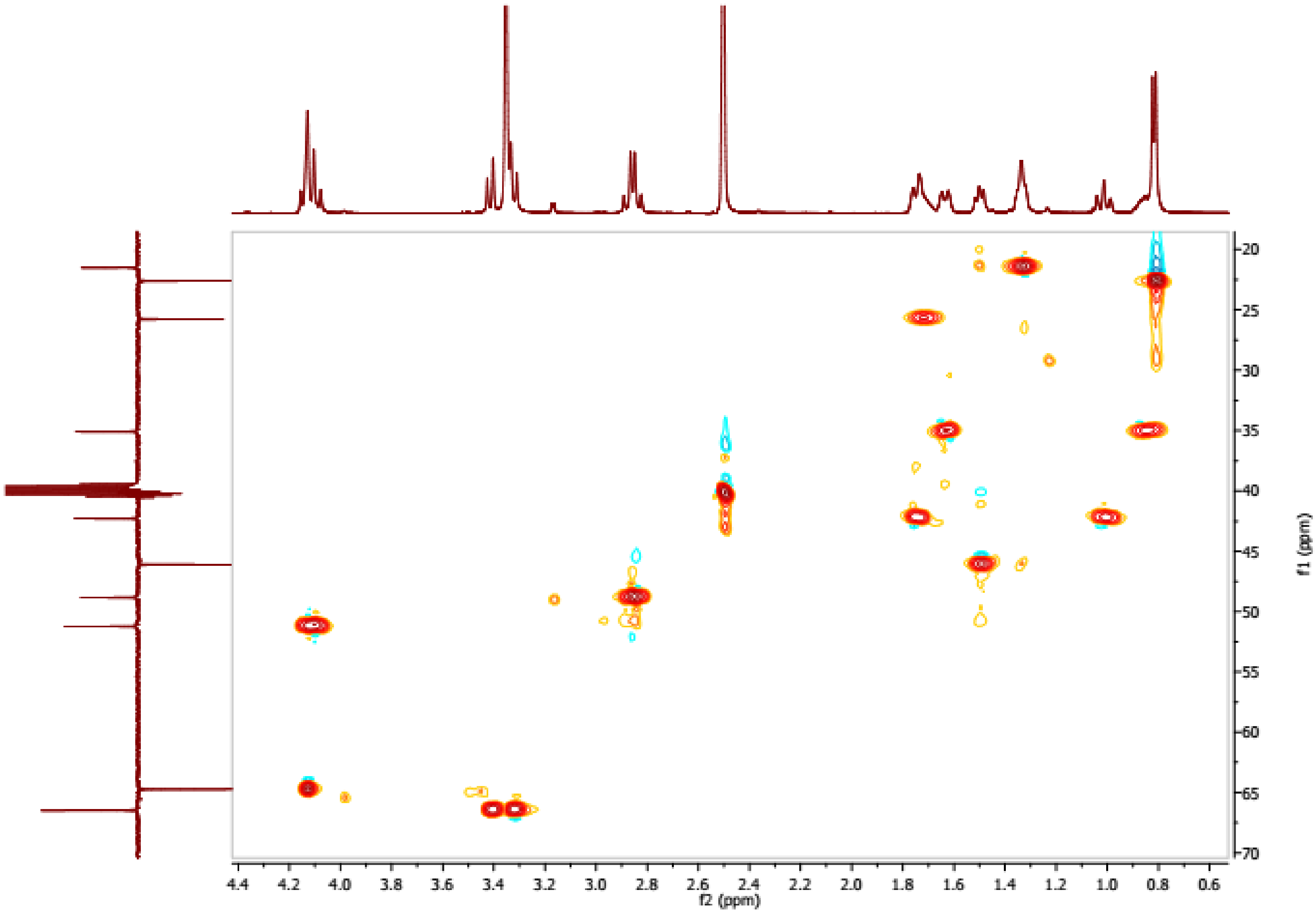

HMBC of compound **32**

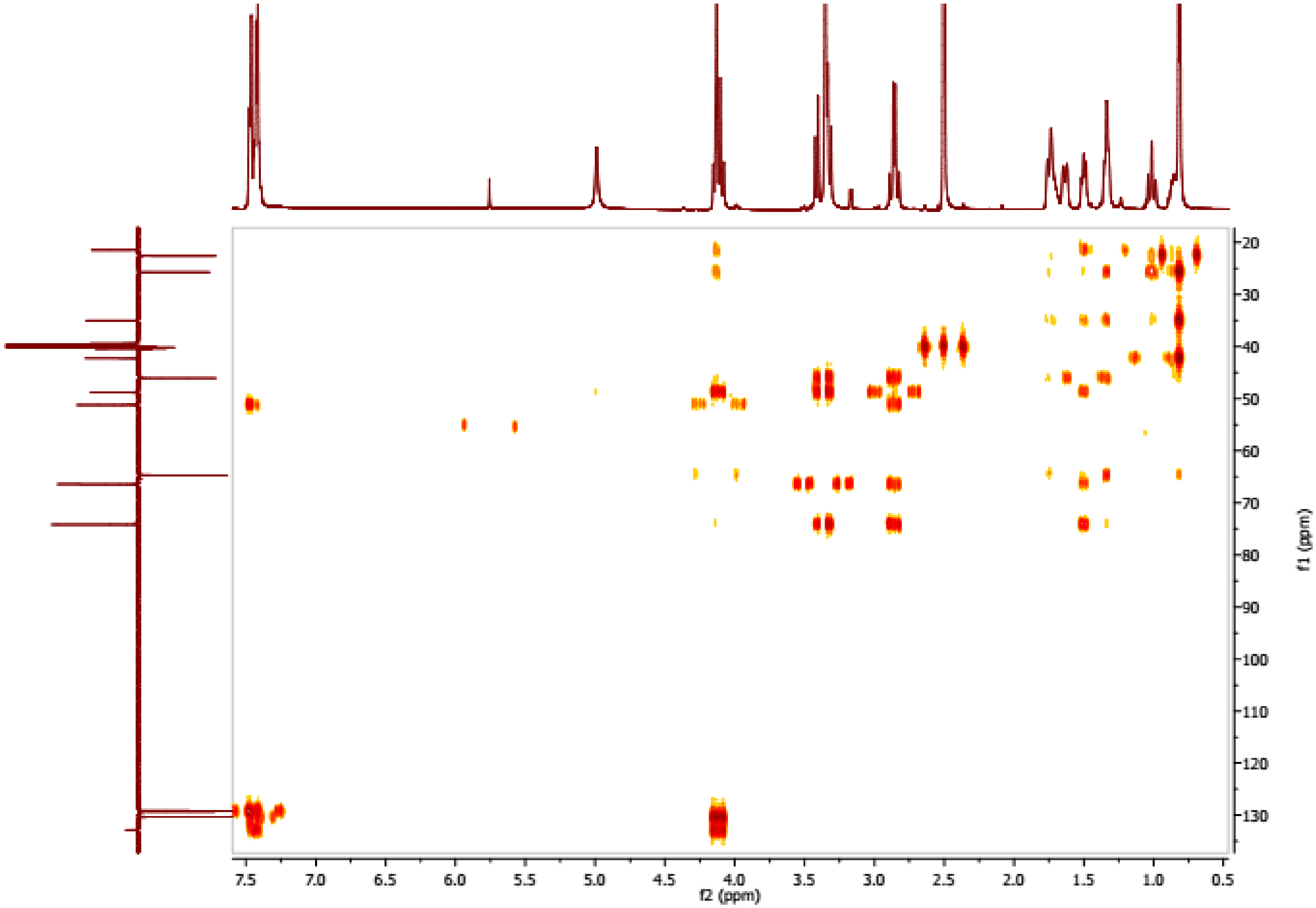

^{1}H-NMR of compound **33**

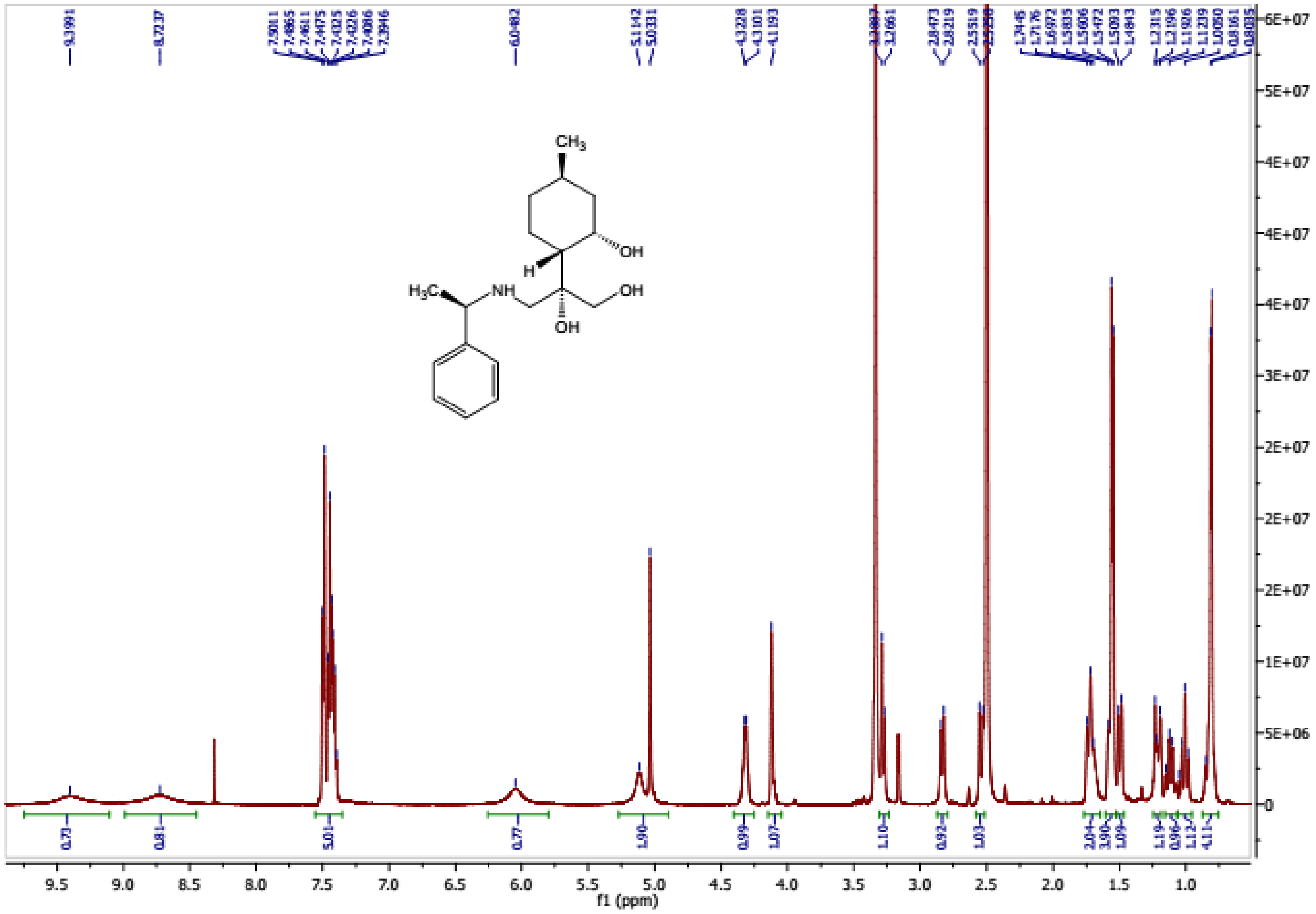

^{13}C-NMR of compound **33**

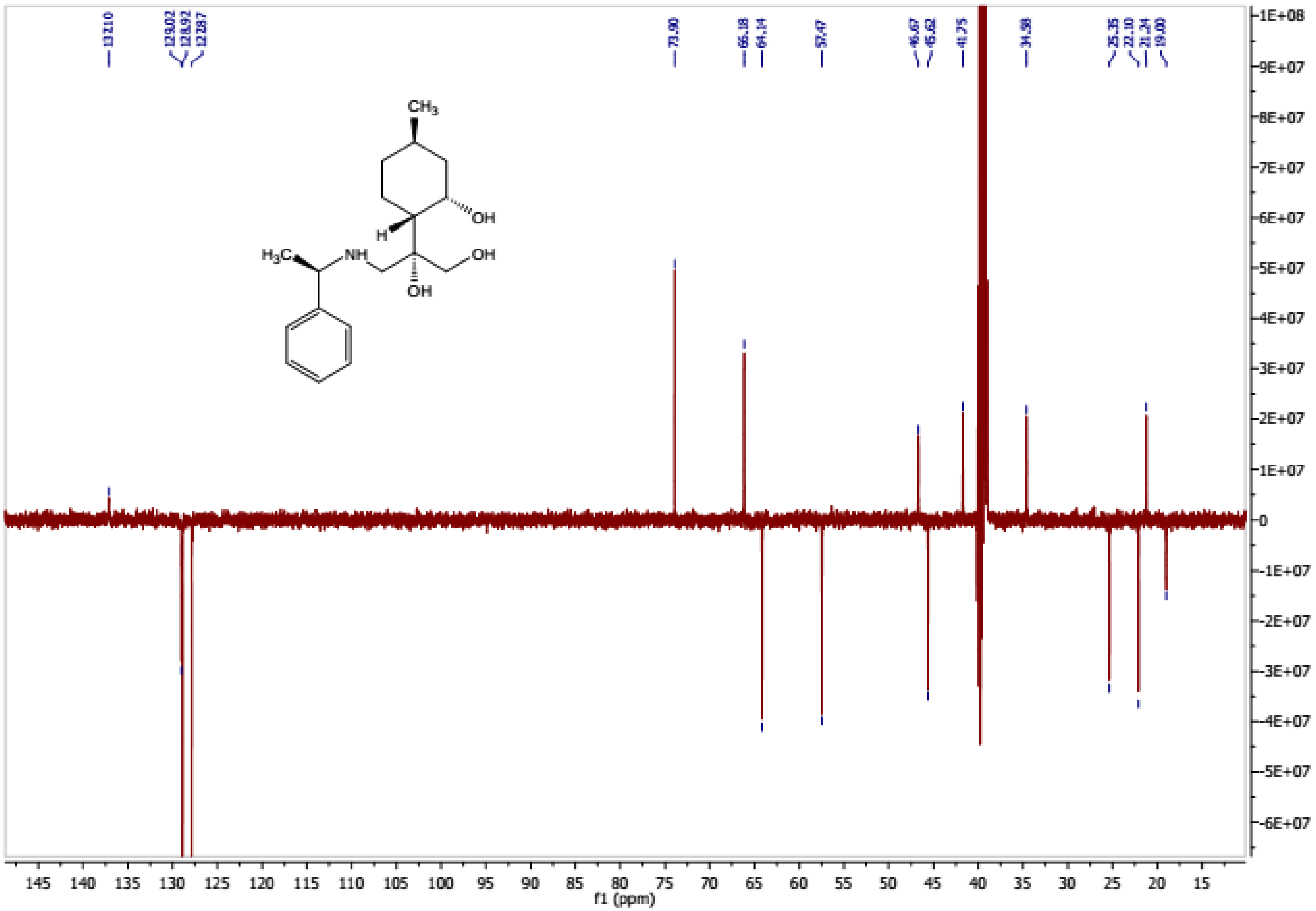

COSY of compound **33**

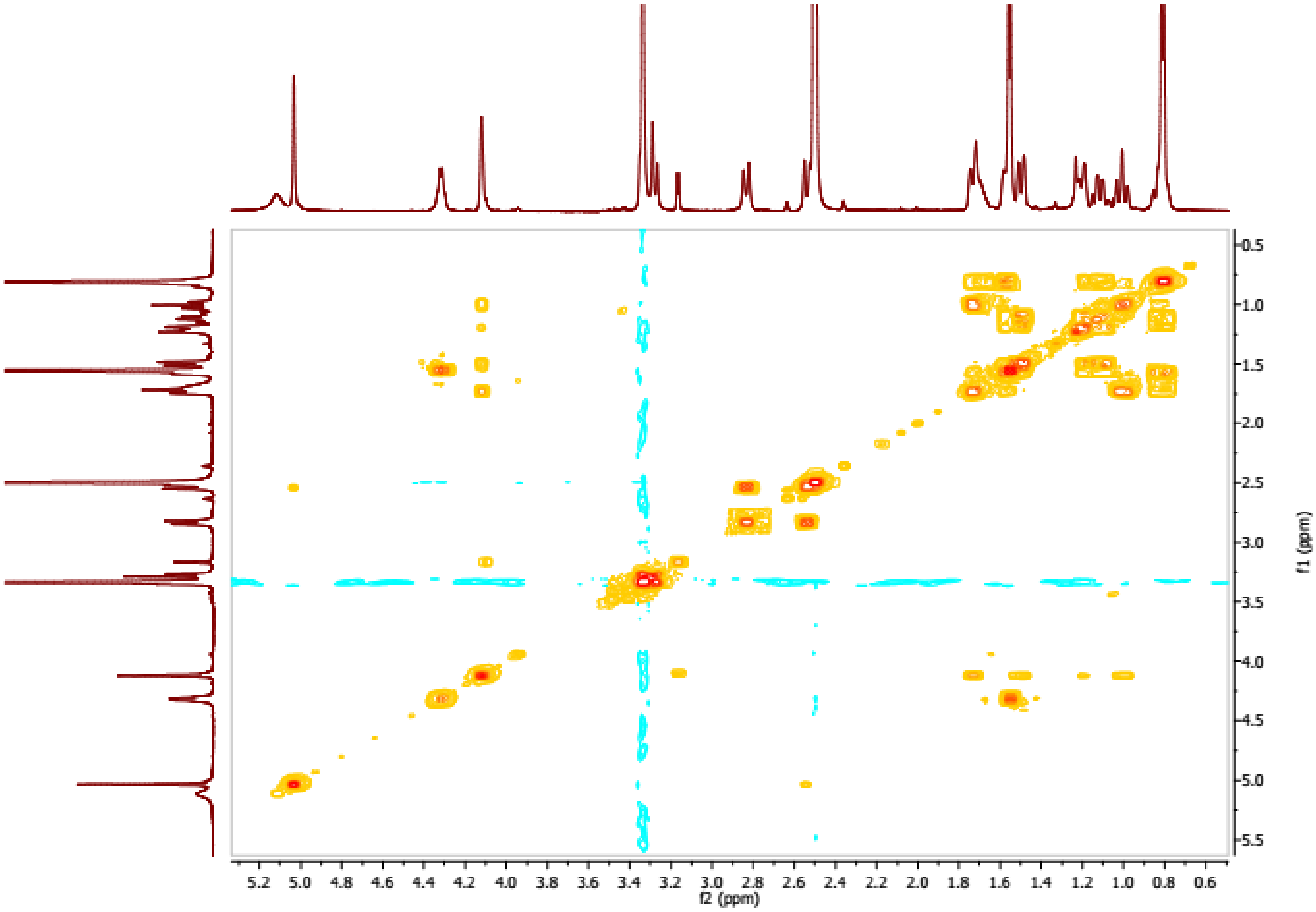

NOESY of compound **33**

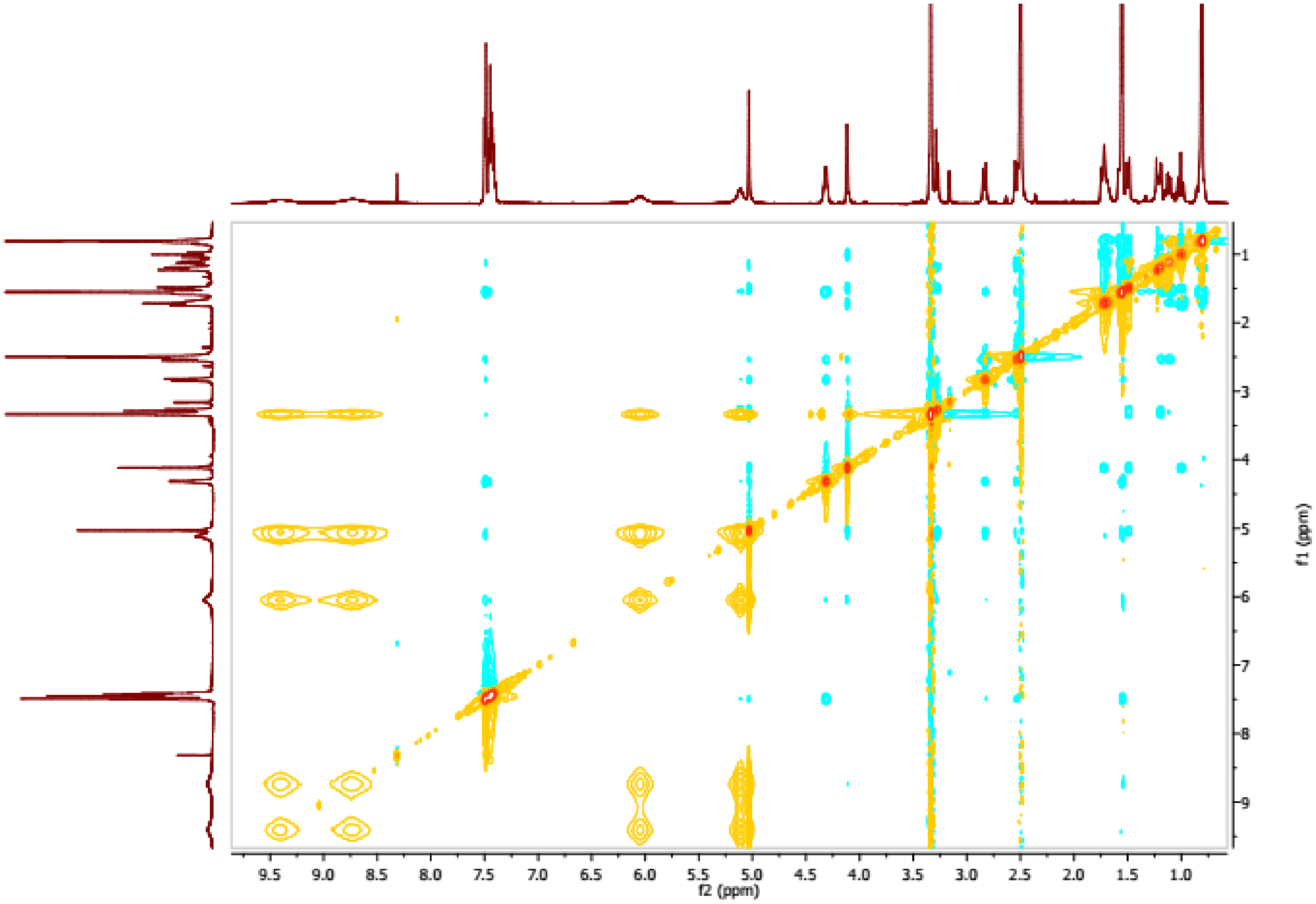

HSQC of compound **33**

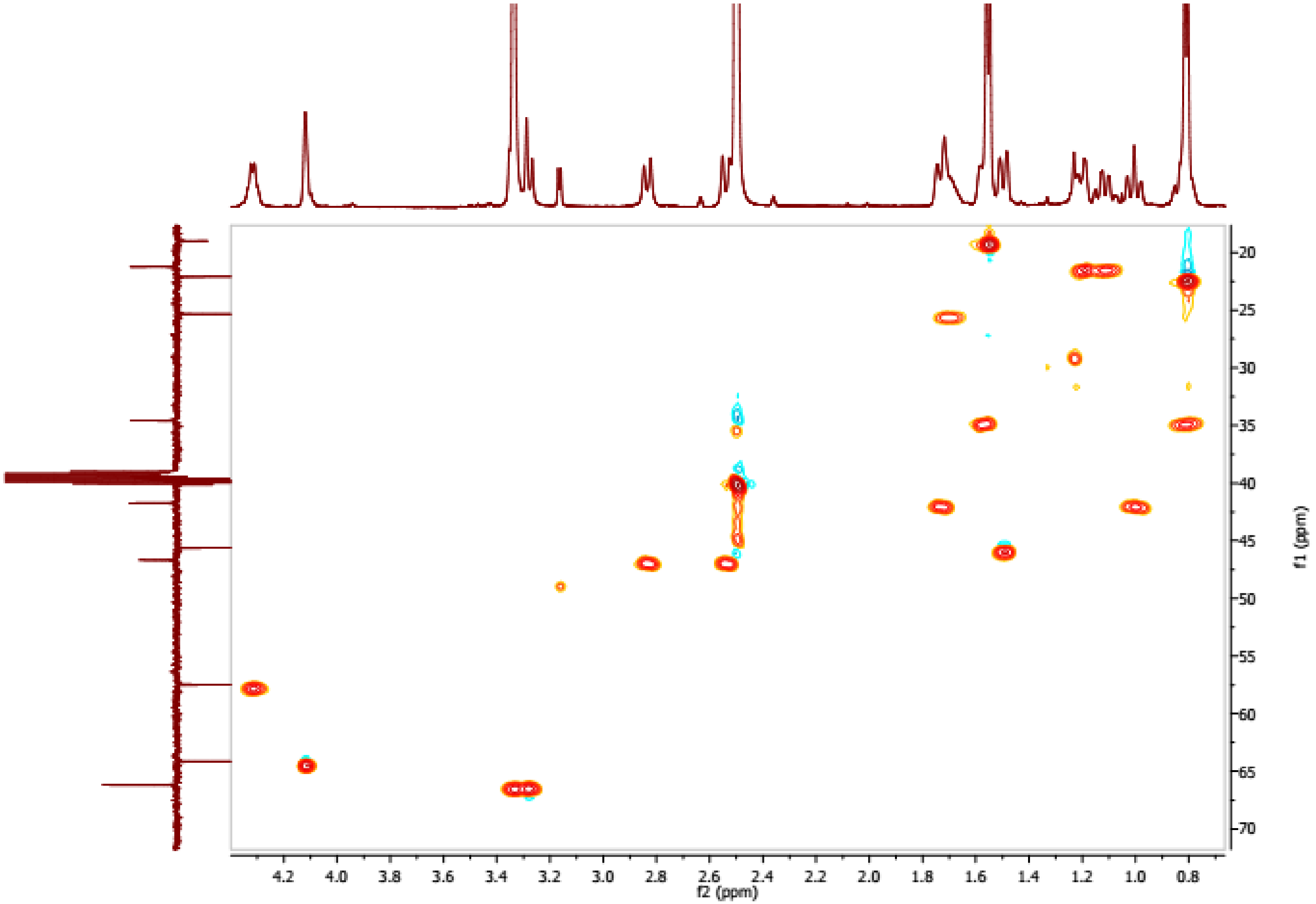

HMBC of compound **33**

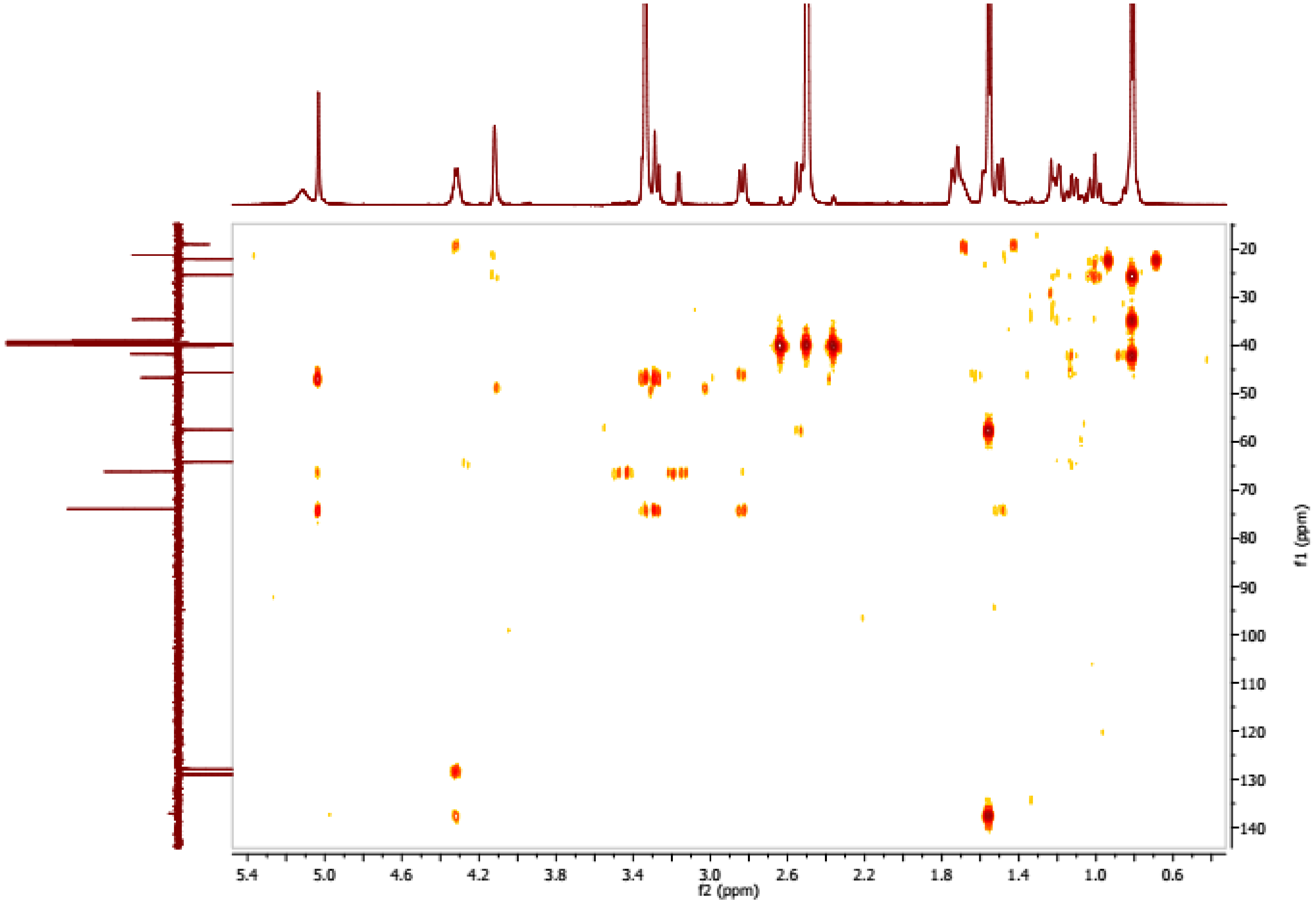

^{1}H-NMR of compound **34**

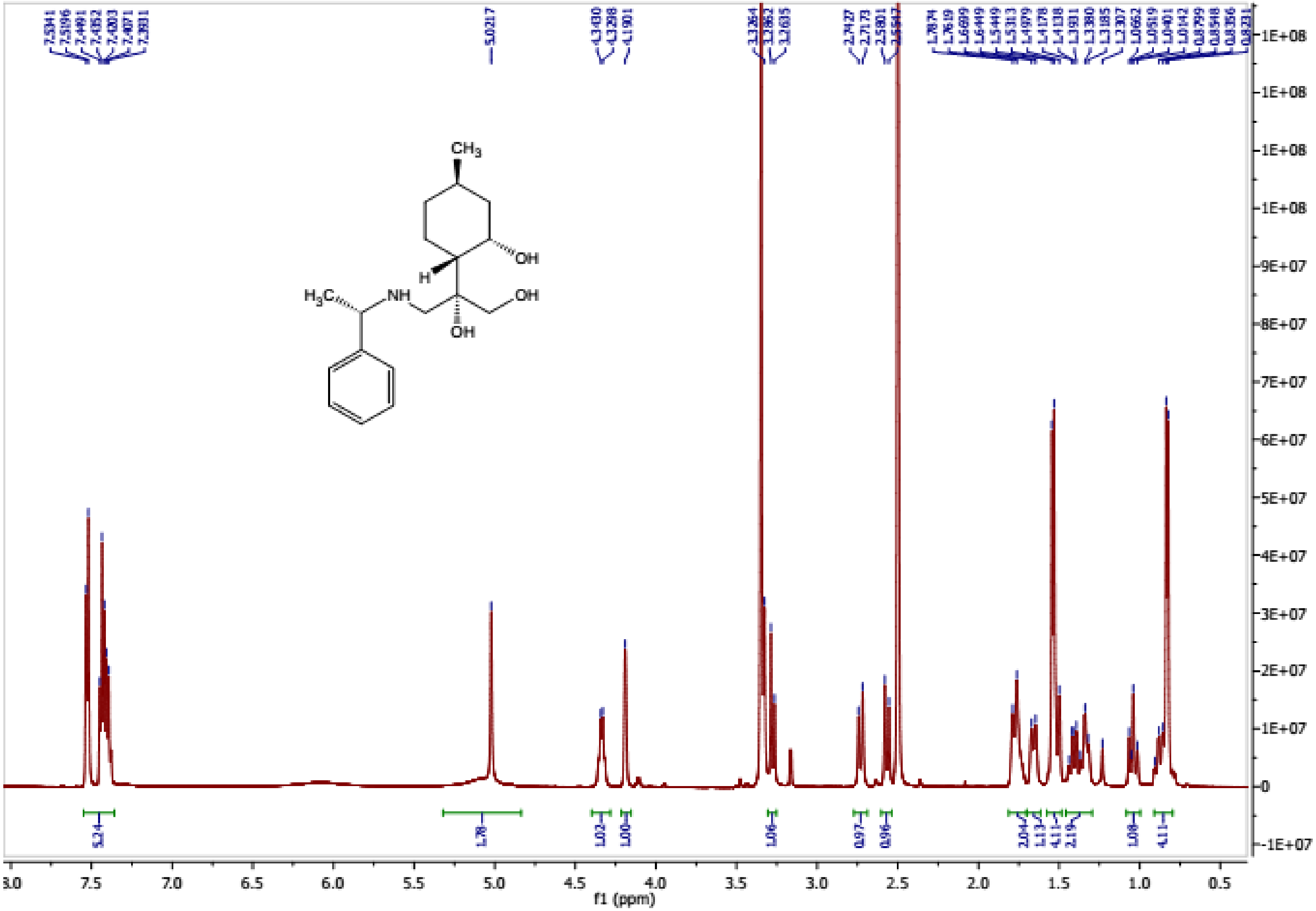

^{13}C-NMR of compound **34**

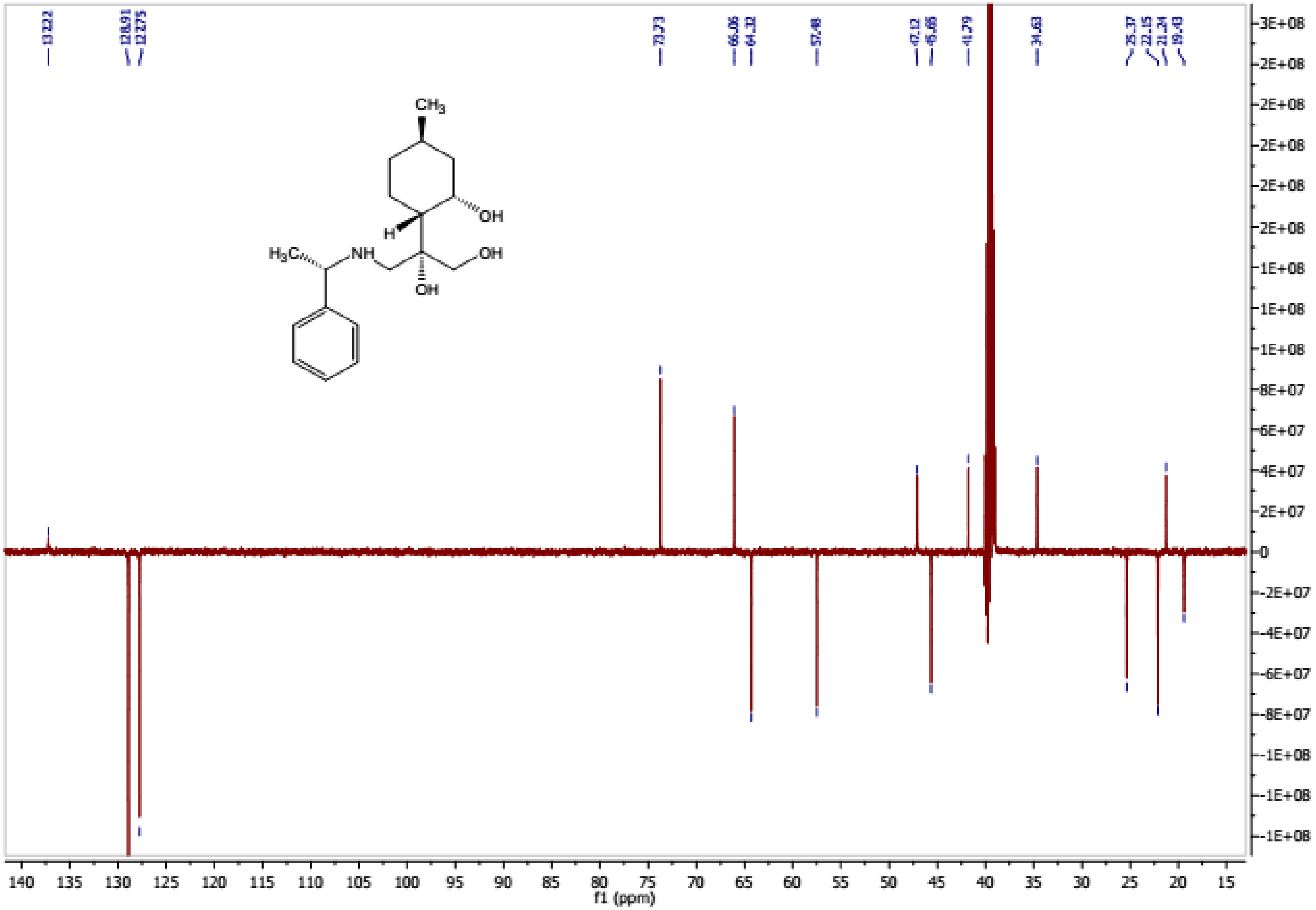

COSY of compound **34**

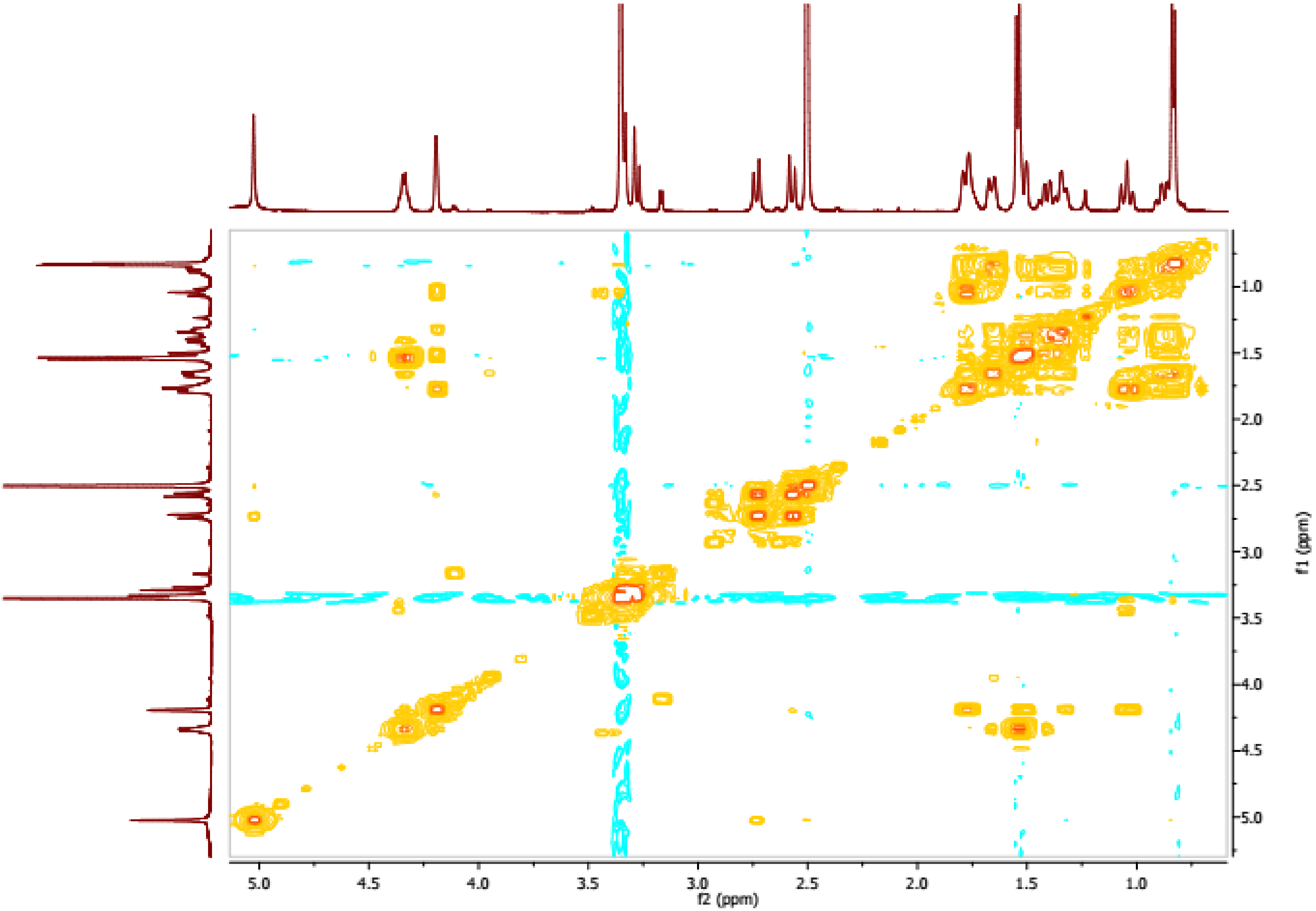

NOESY of compound **34**

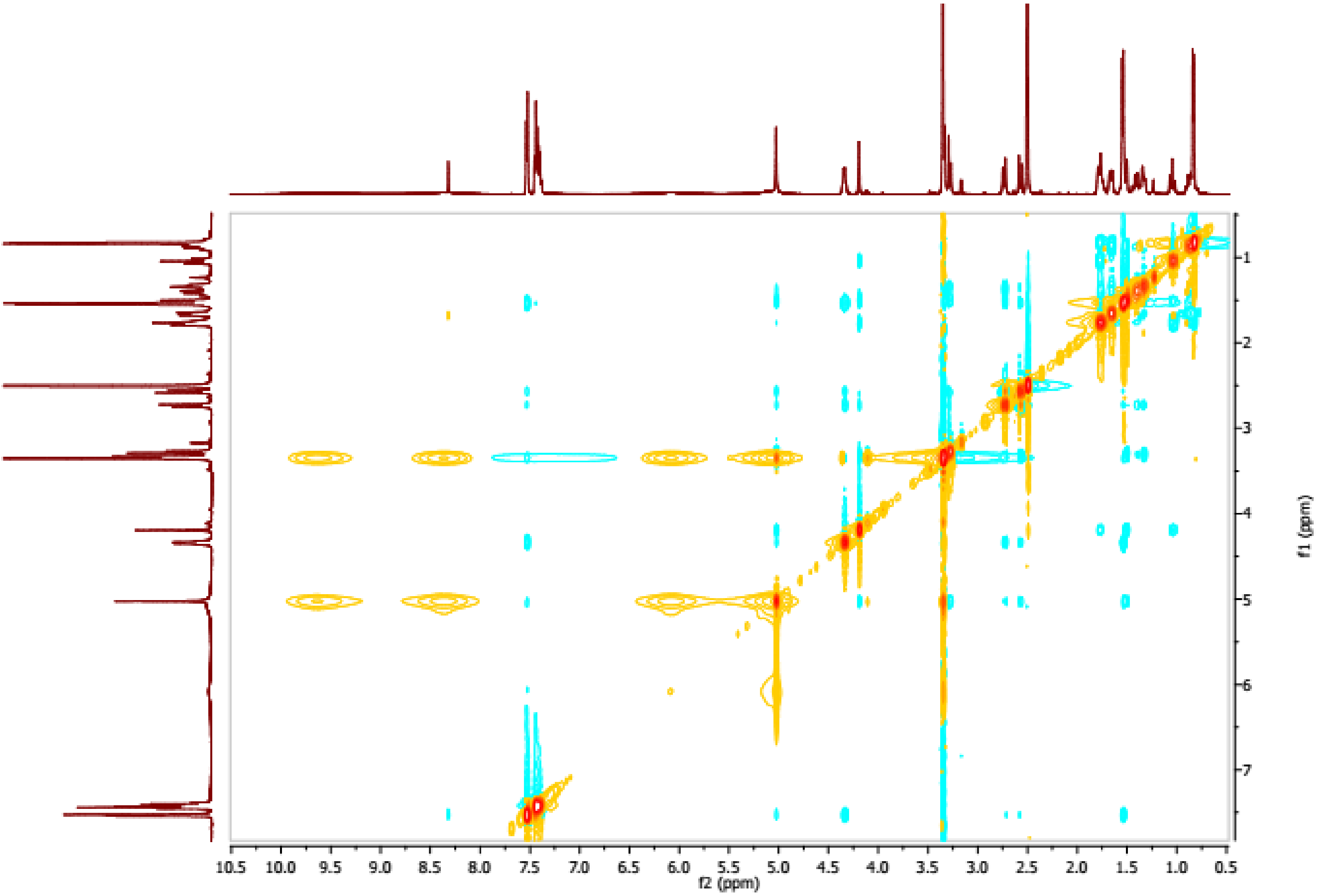

HSQC of compound **34**

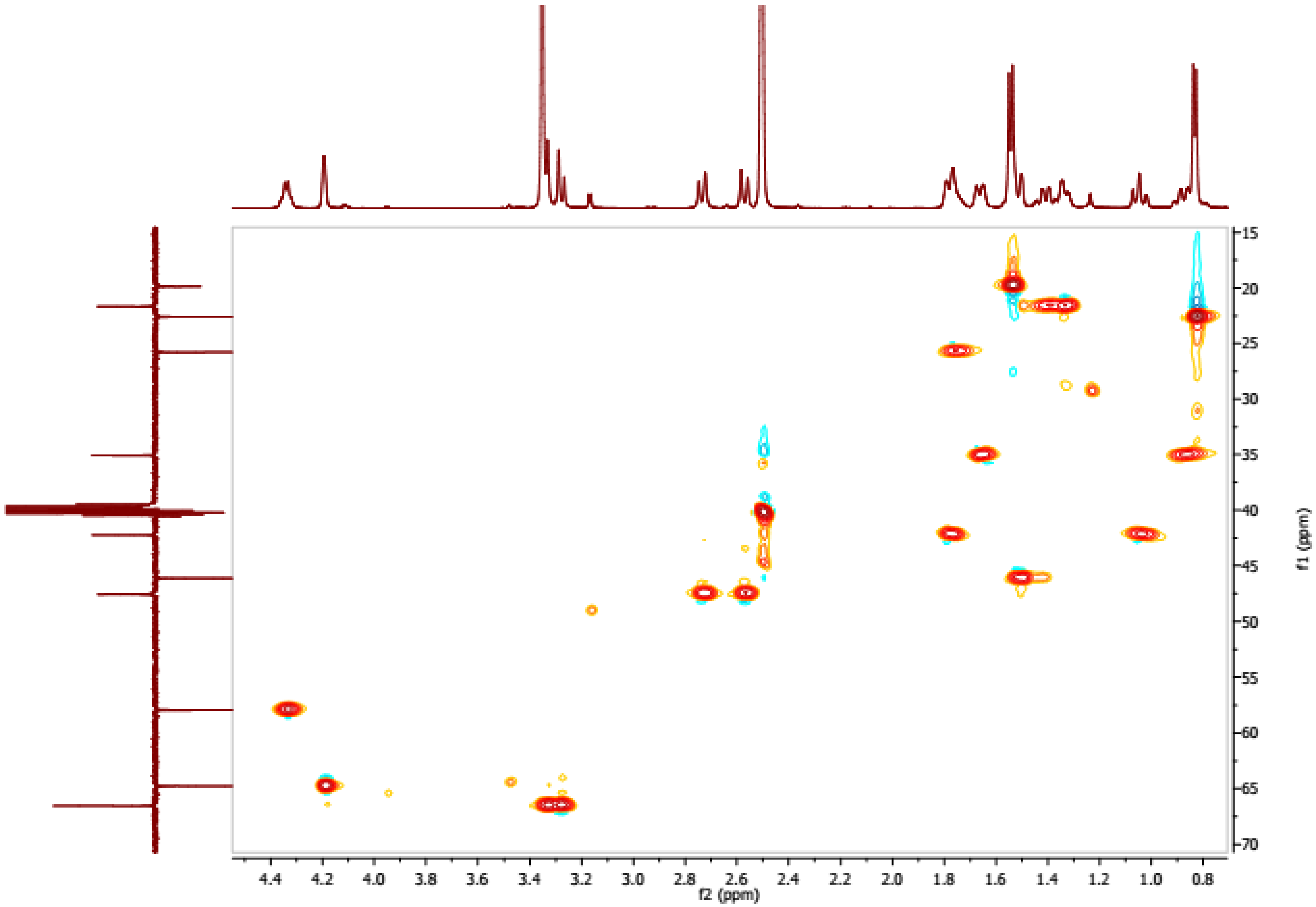

HMBC of compound **34**

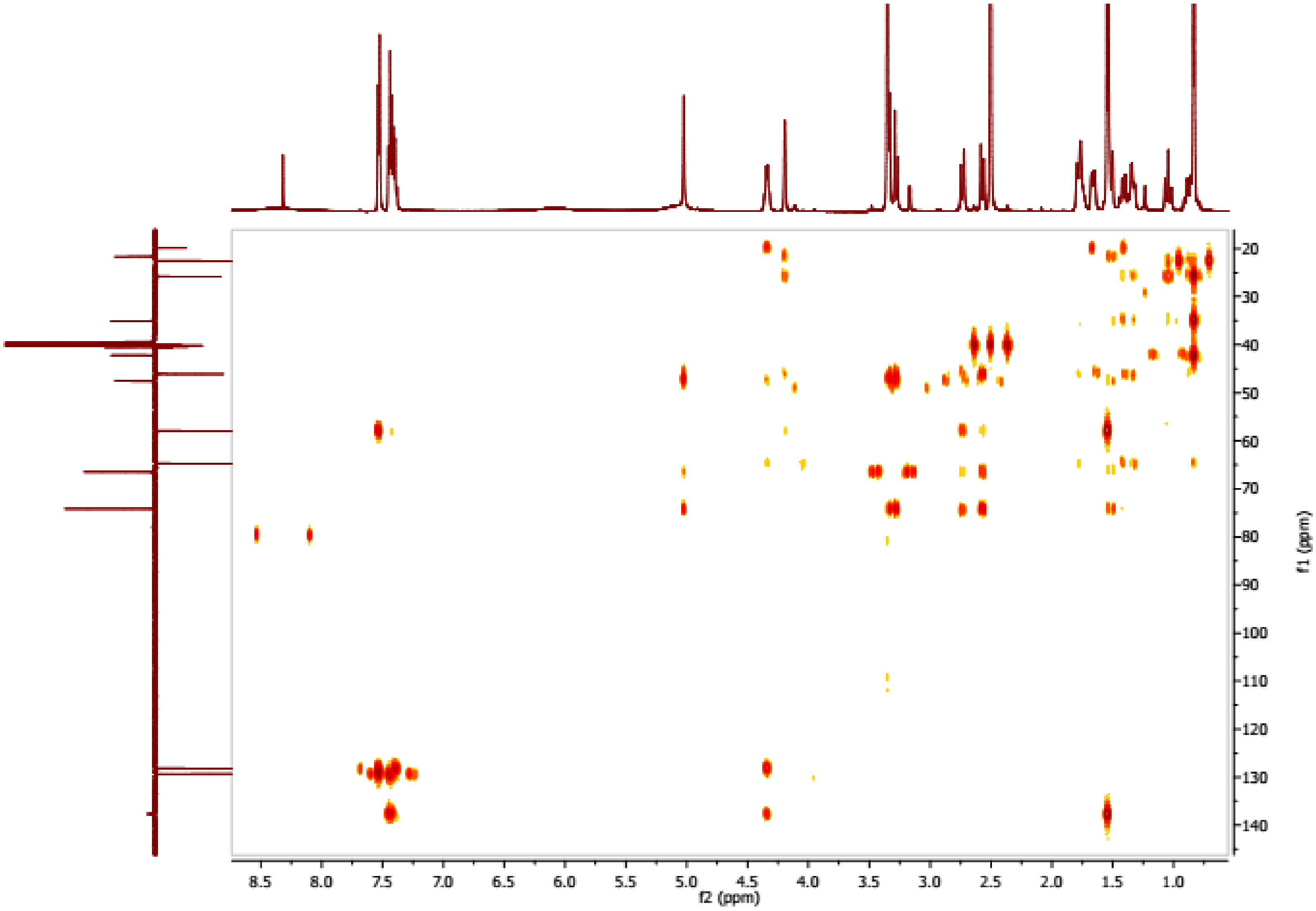

^{1}H-NMR of compound **35**

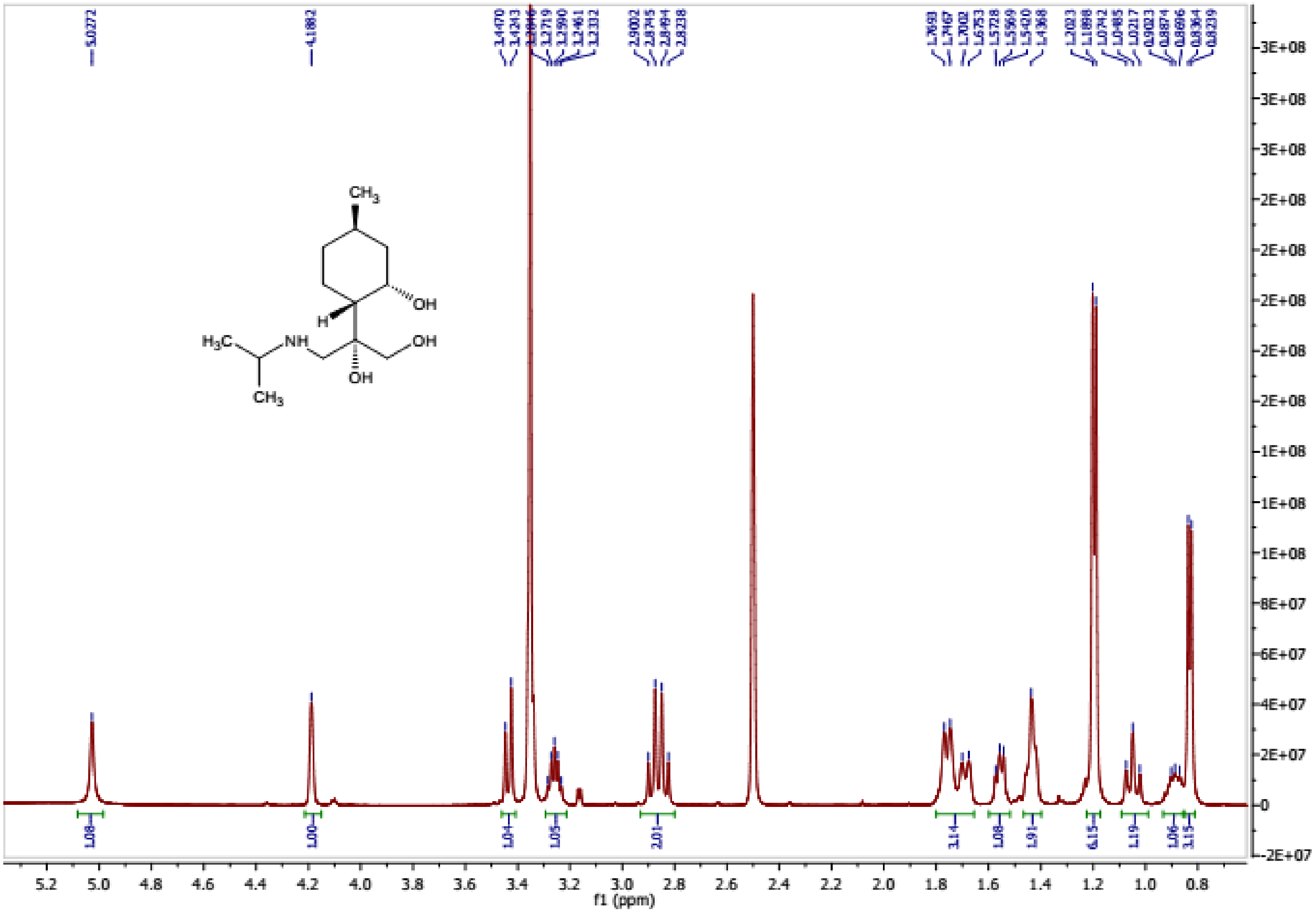

^{13}C-NMR of compound **35**

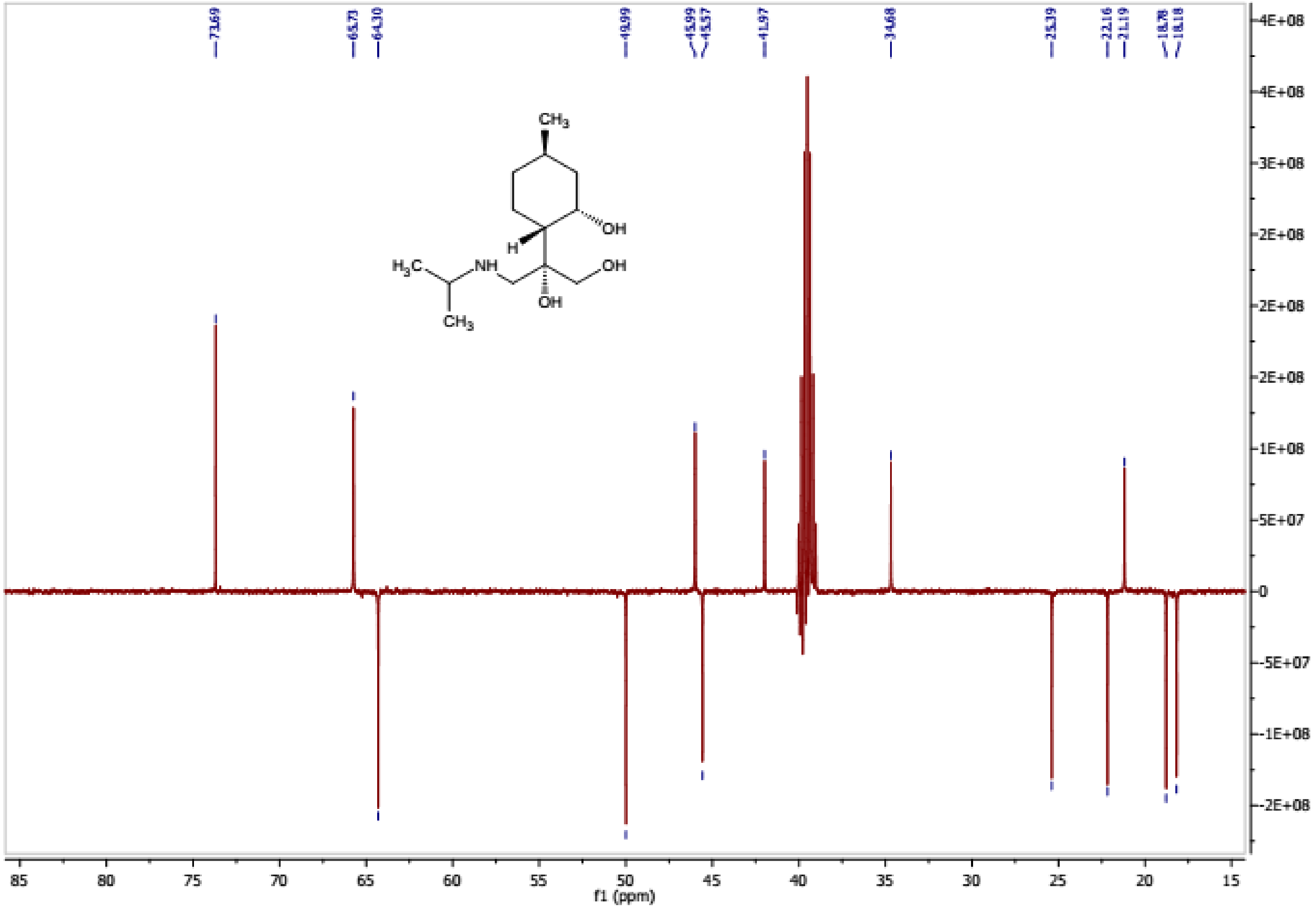

COSY of compound **35**

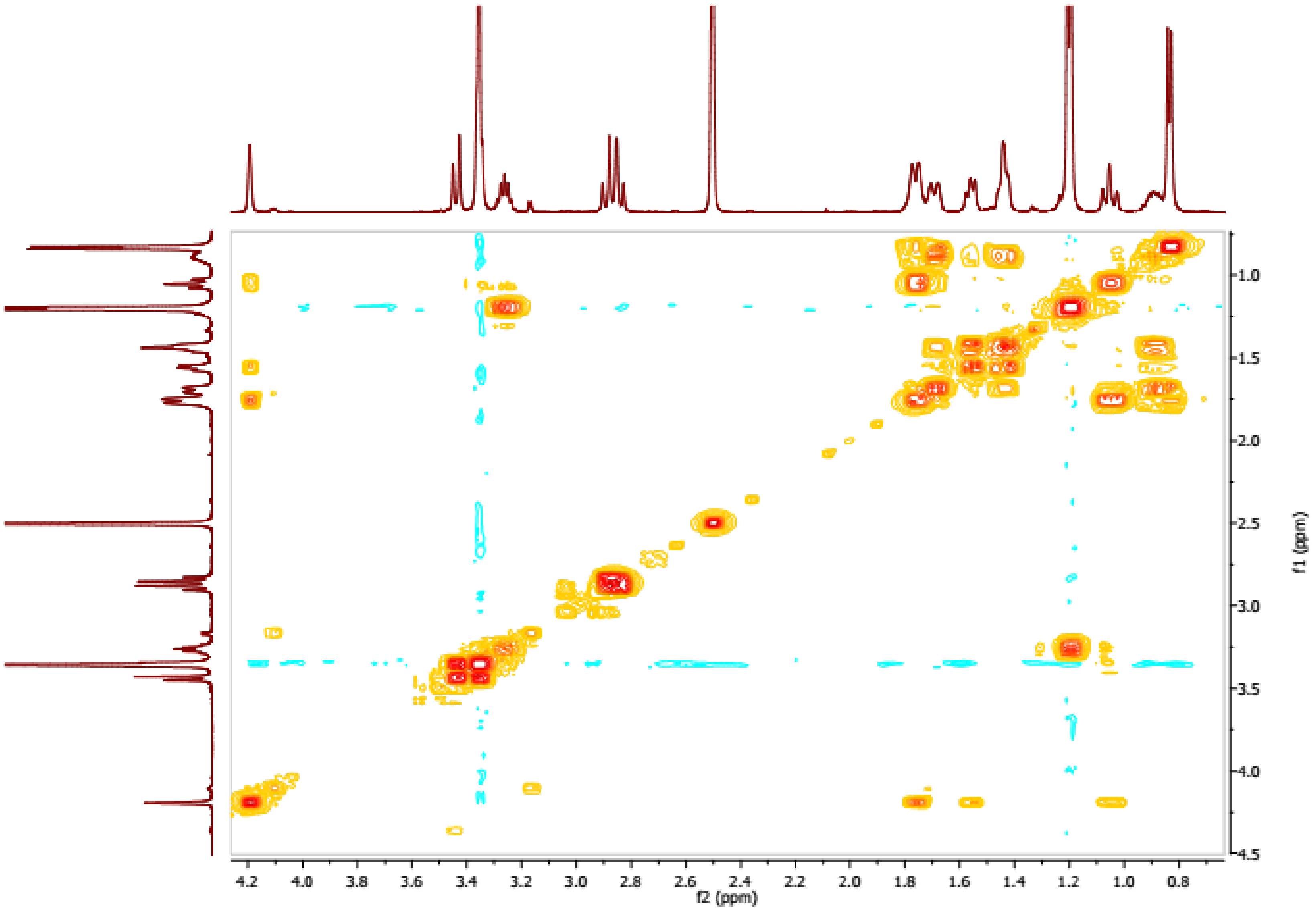

NOESY of compound **34**

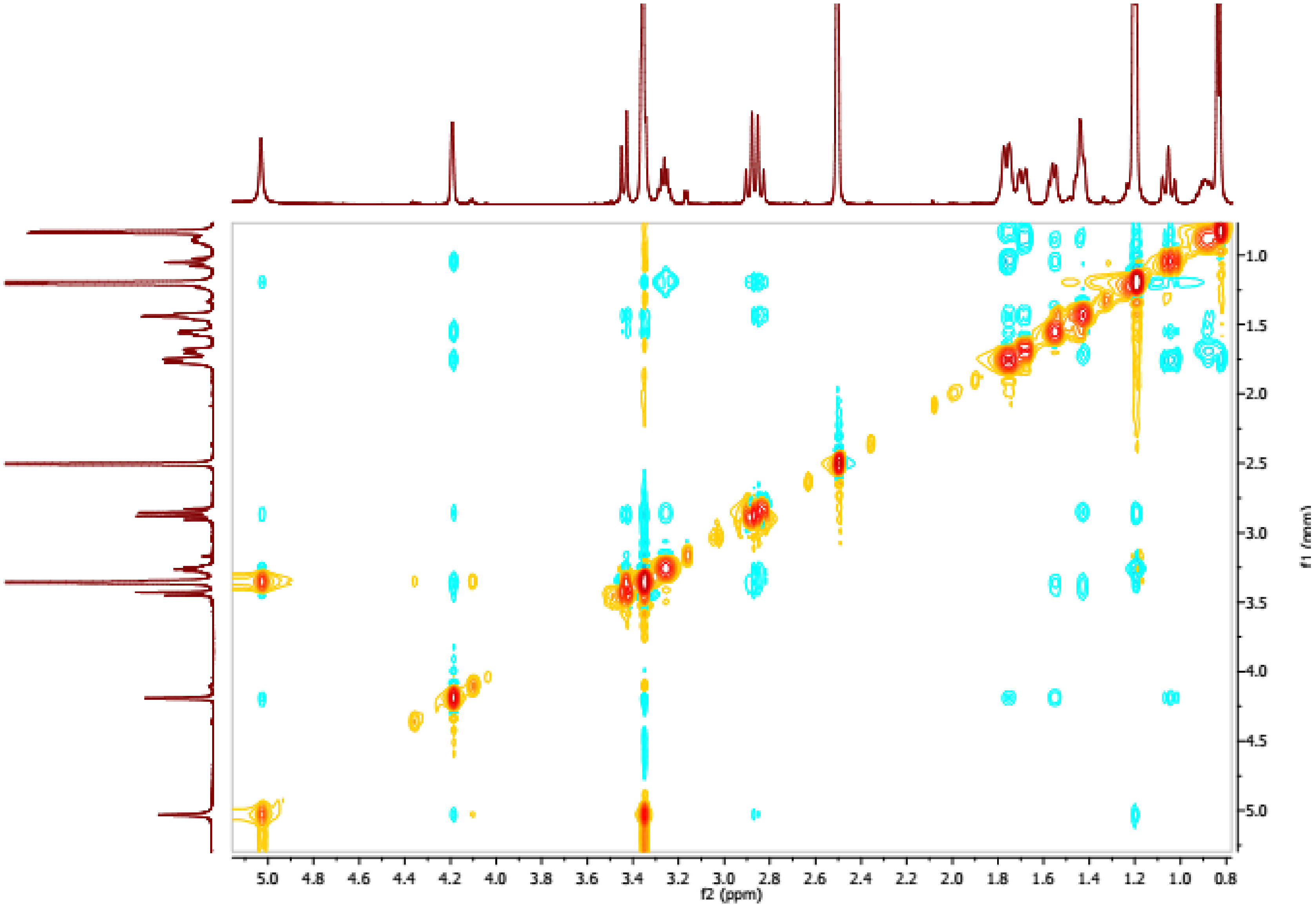

HSQC of compound **35**

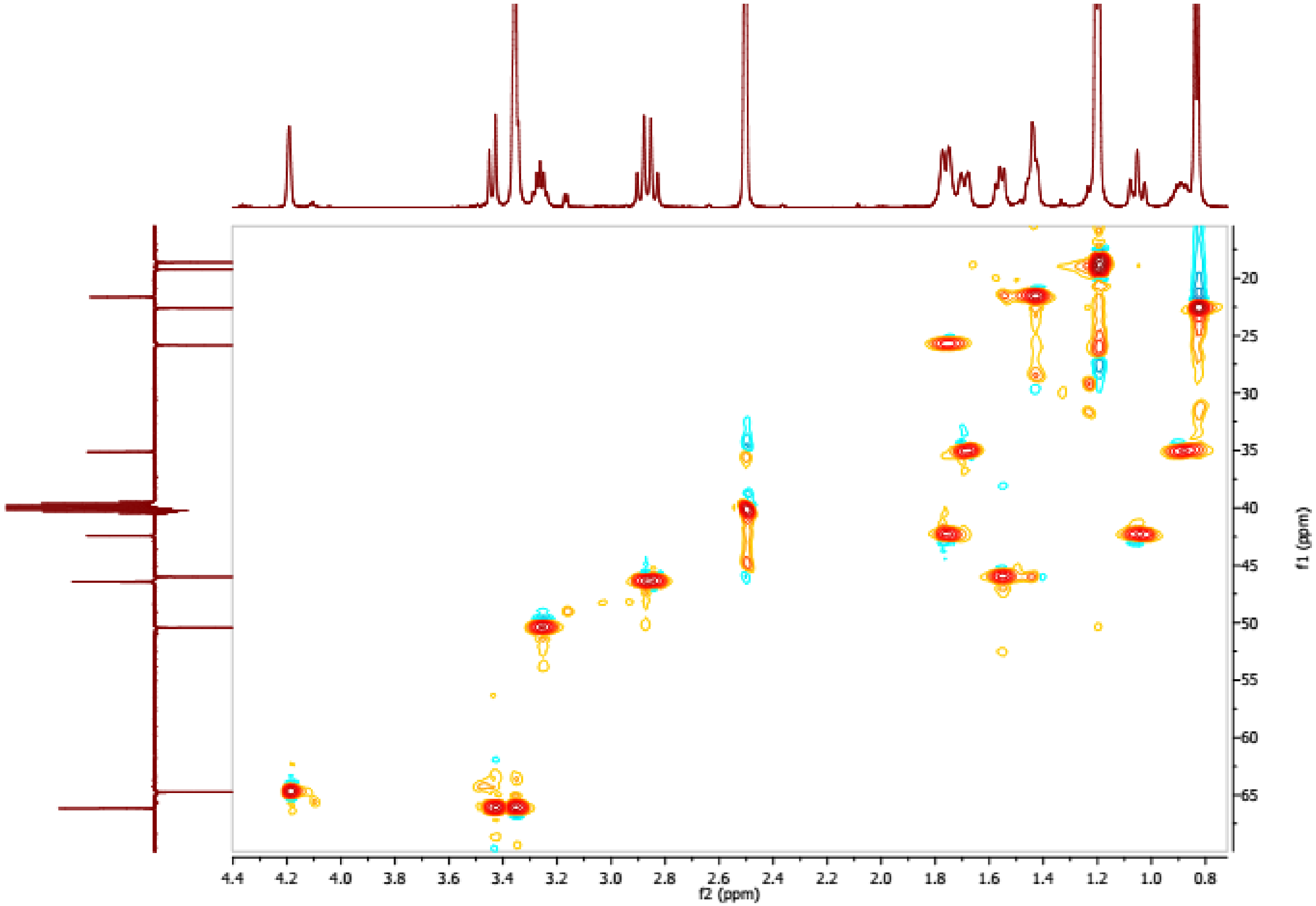

HMBC of compound **35**

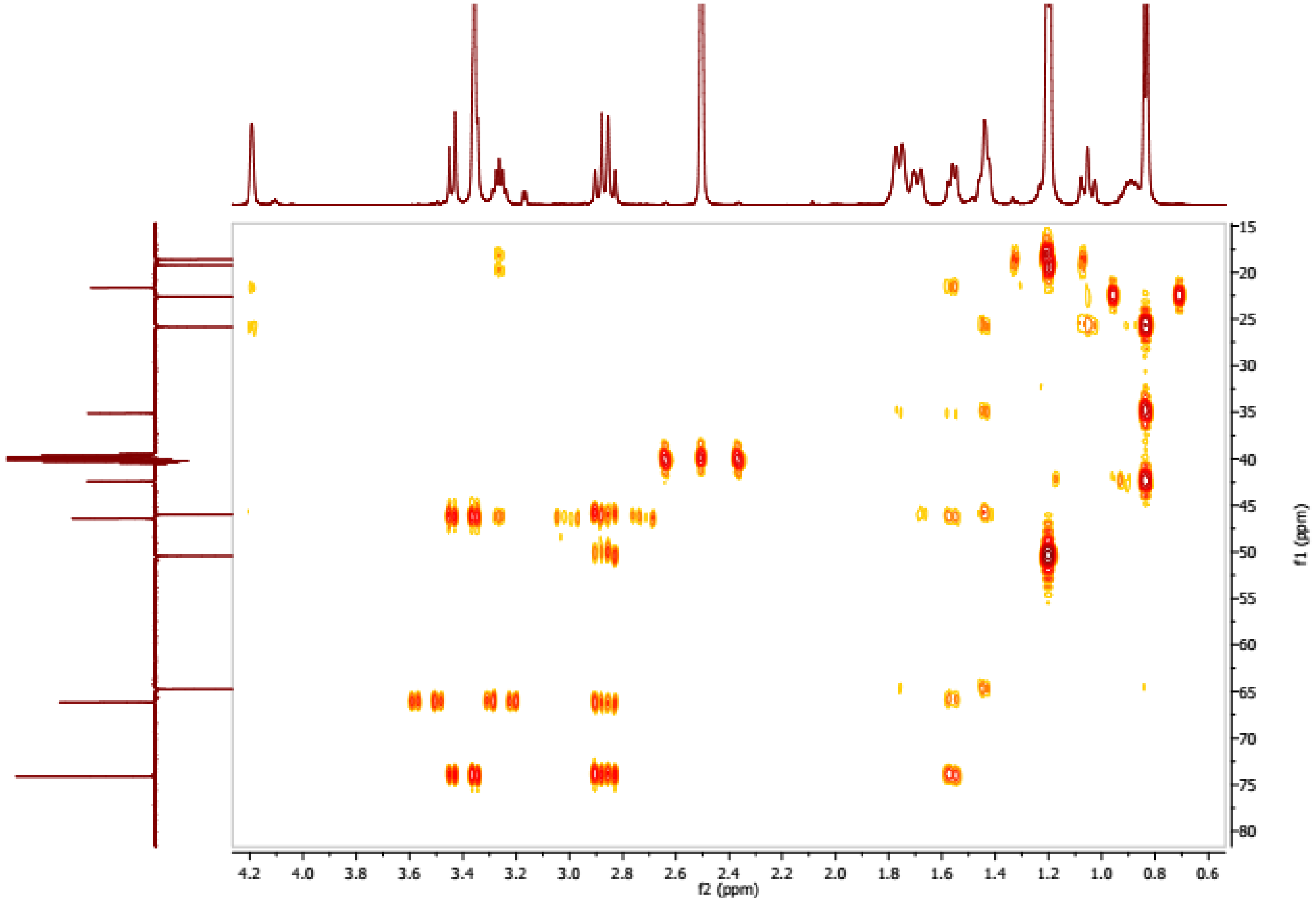

^{1}H-NMR of compound **36**

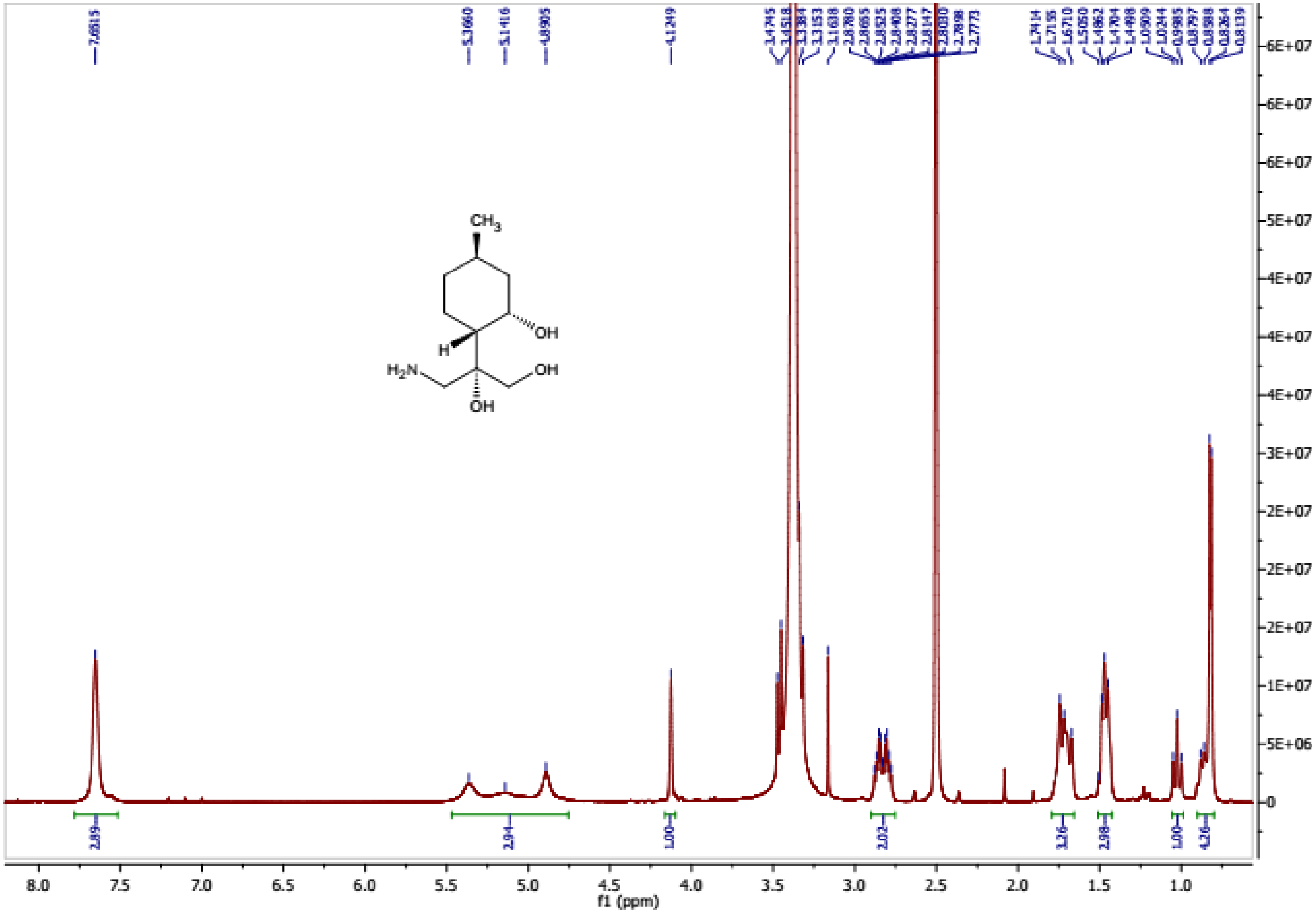

^{13}C-NMR of compound **36**

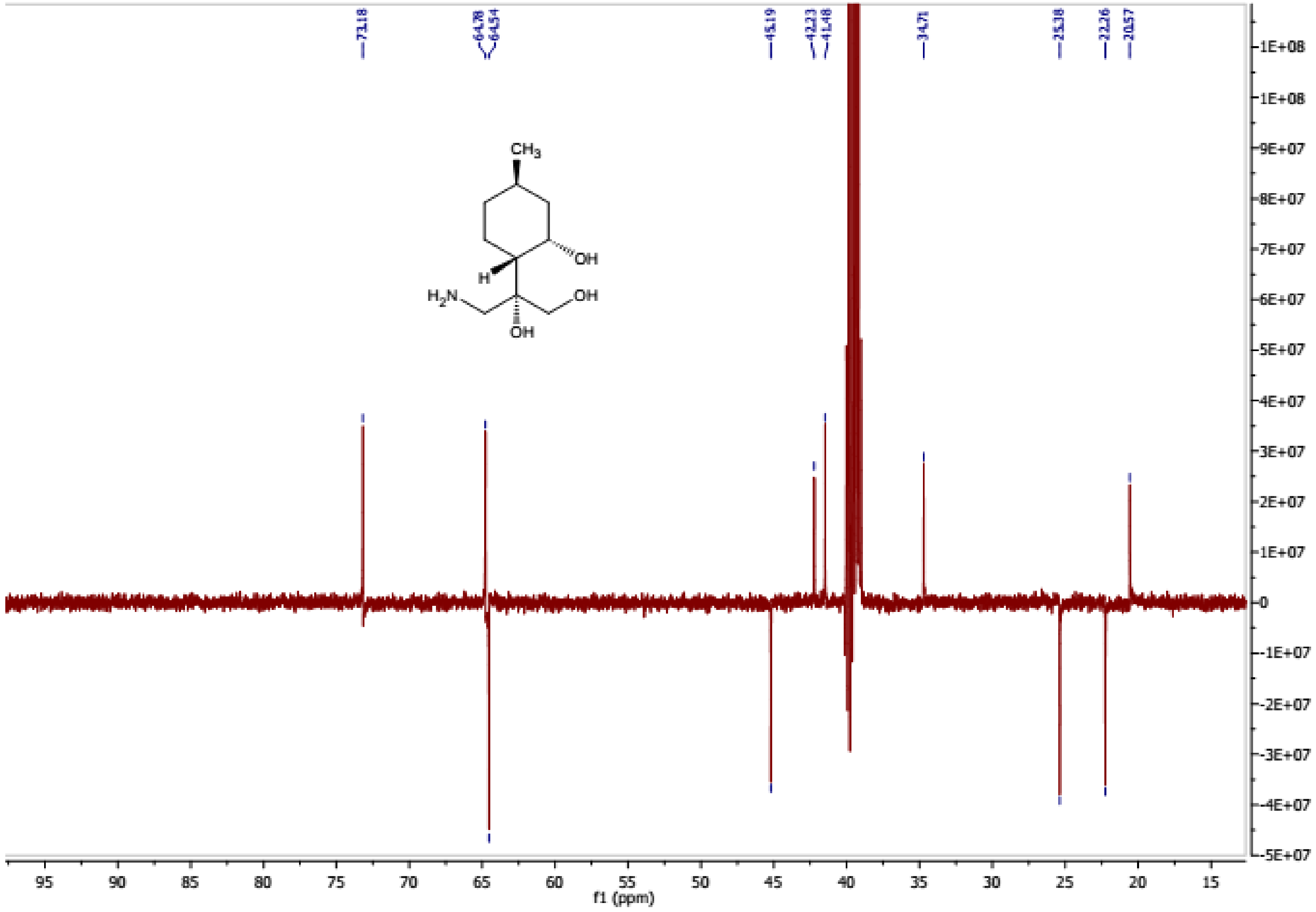

^{1}H-NMR of compound **37**

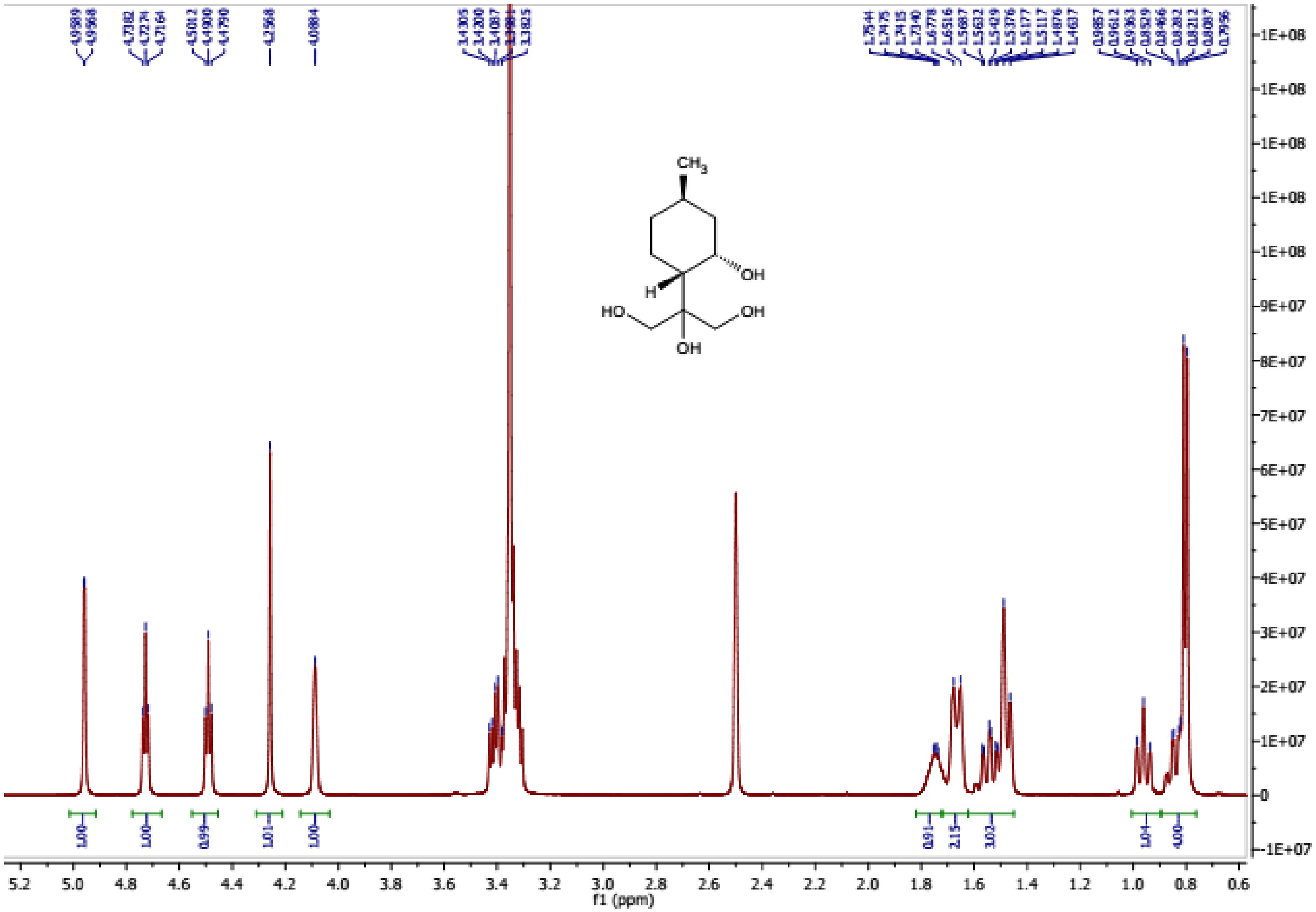

^{13}C-NMR of compound **37**

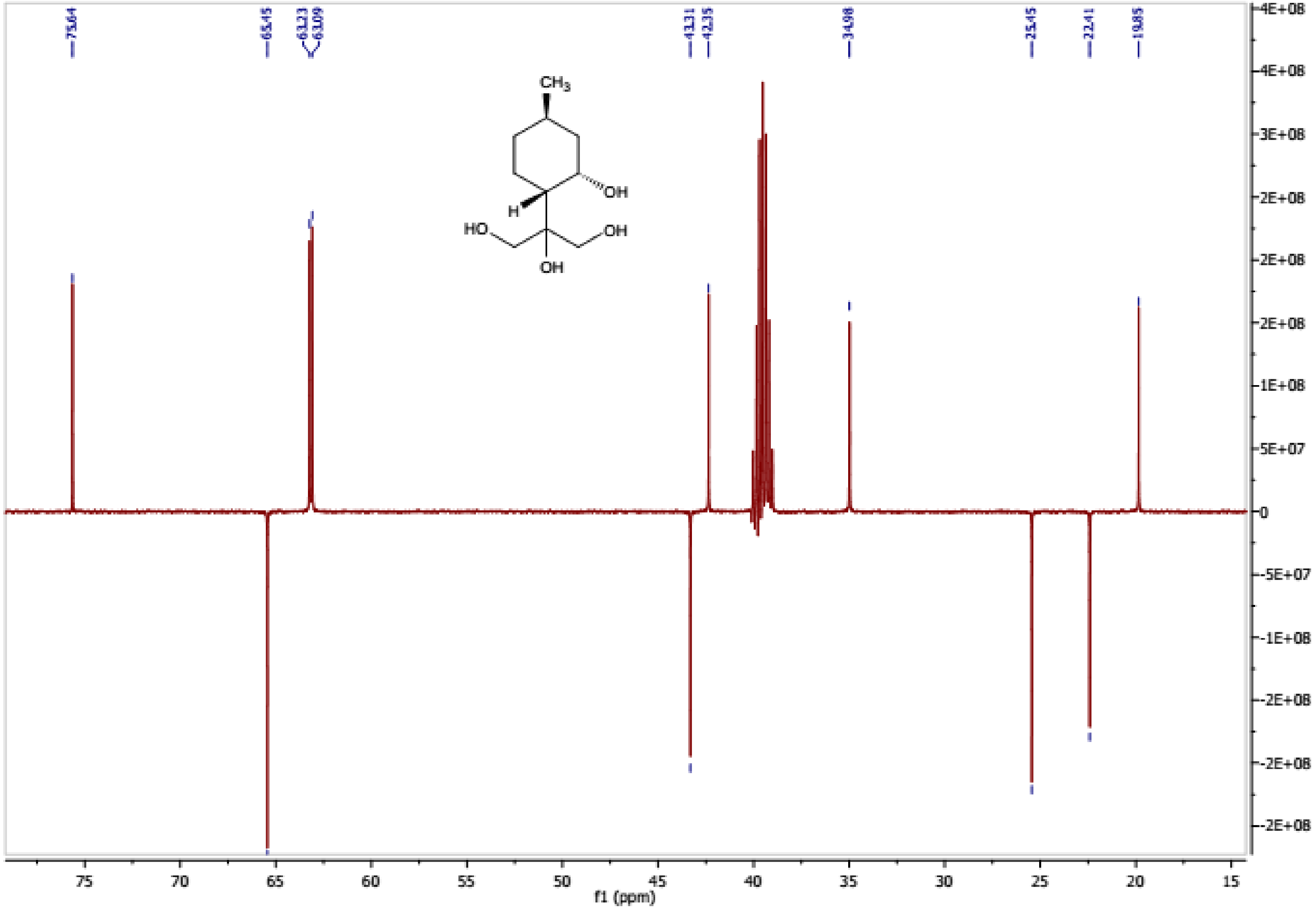

COSY of compound **37**

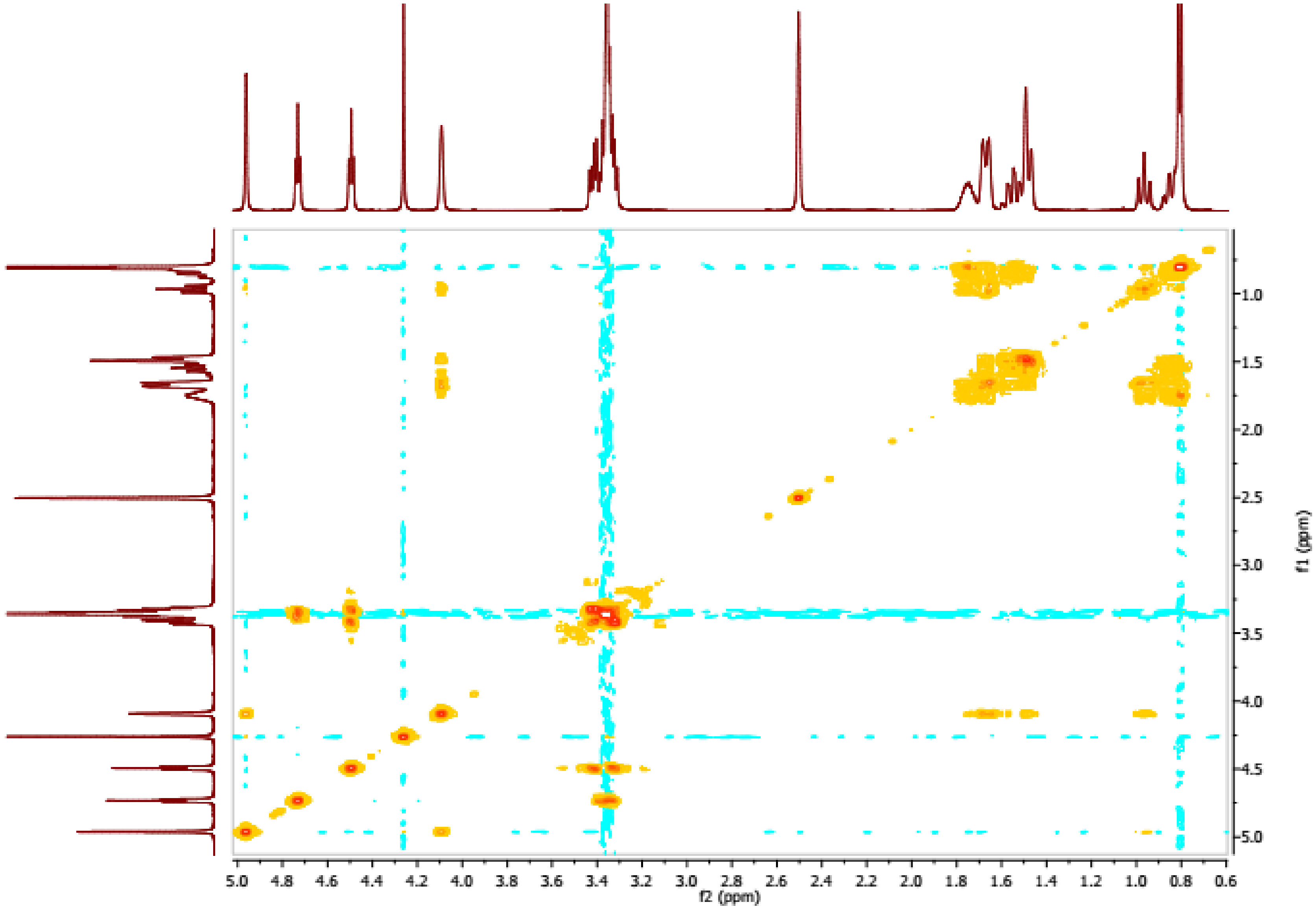

NOESY of compound **37**

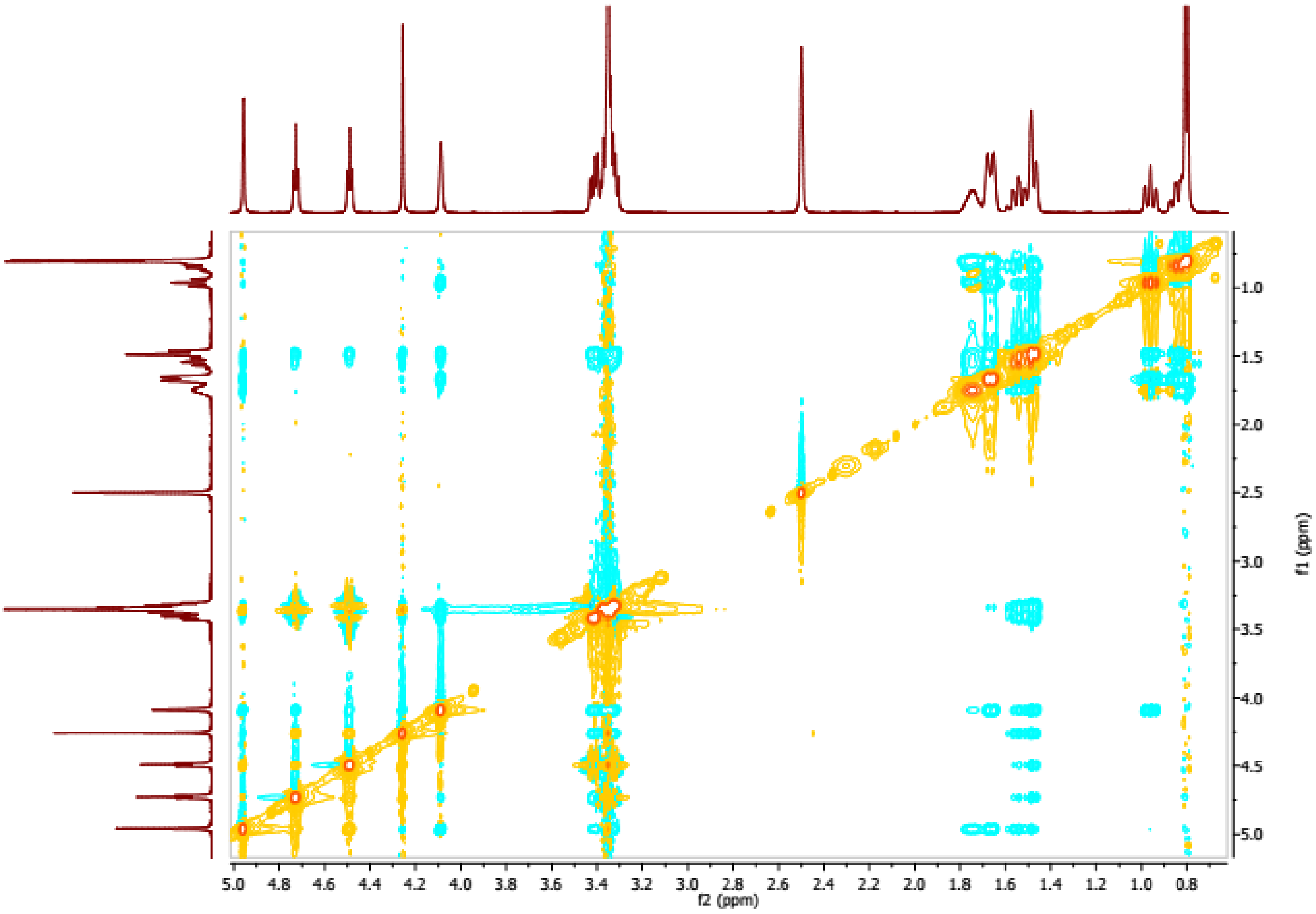

HSQC of compound **37**

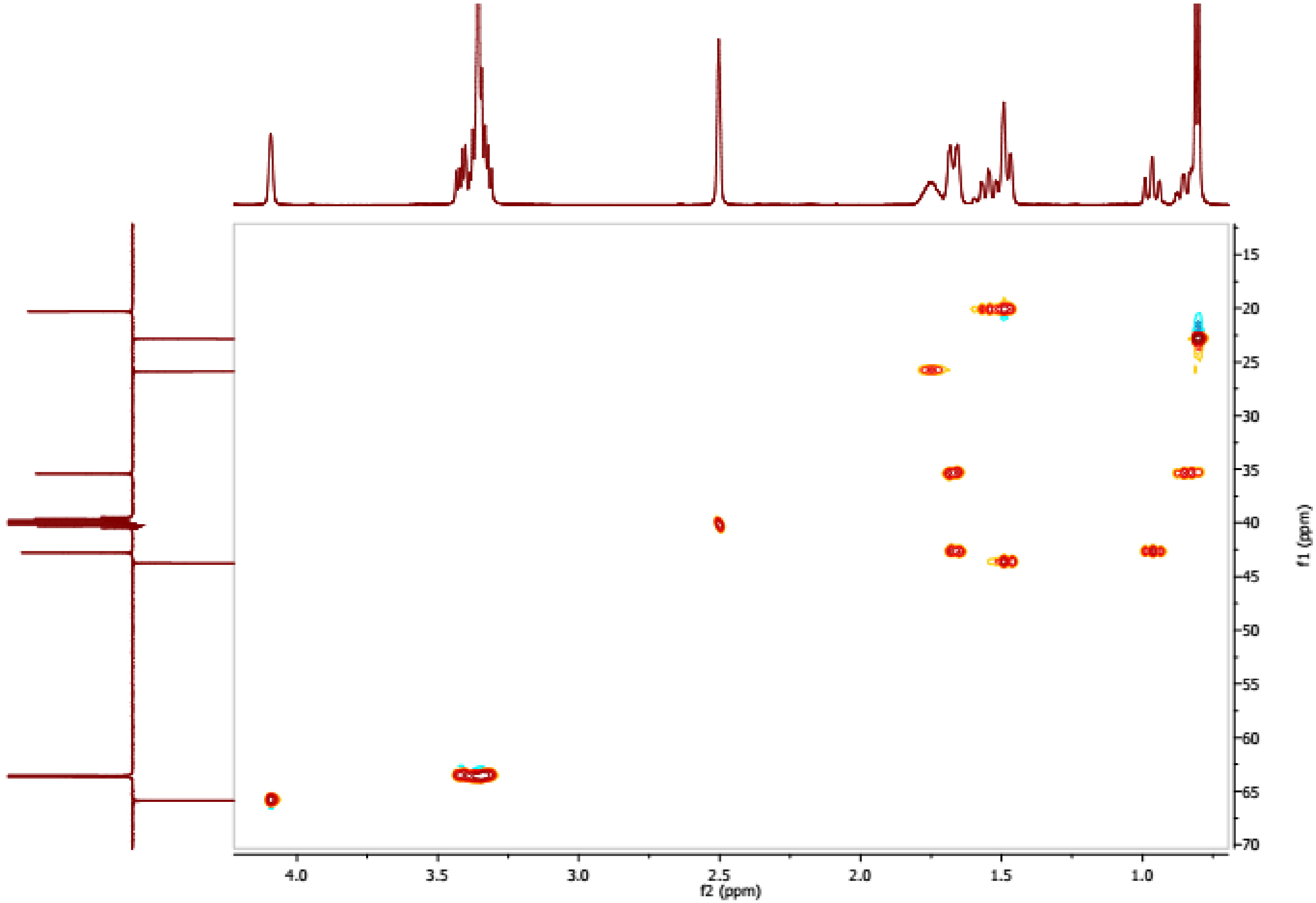

HMBC of compound **37**

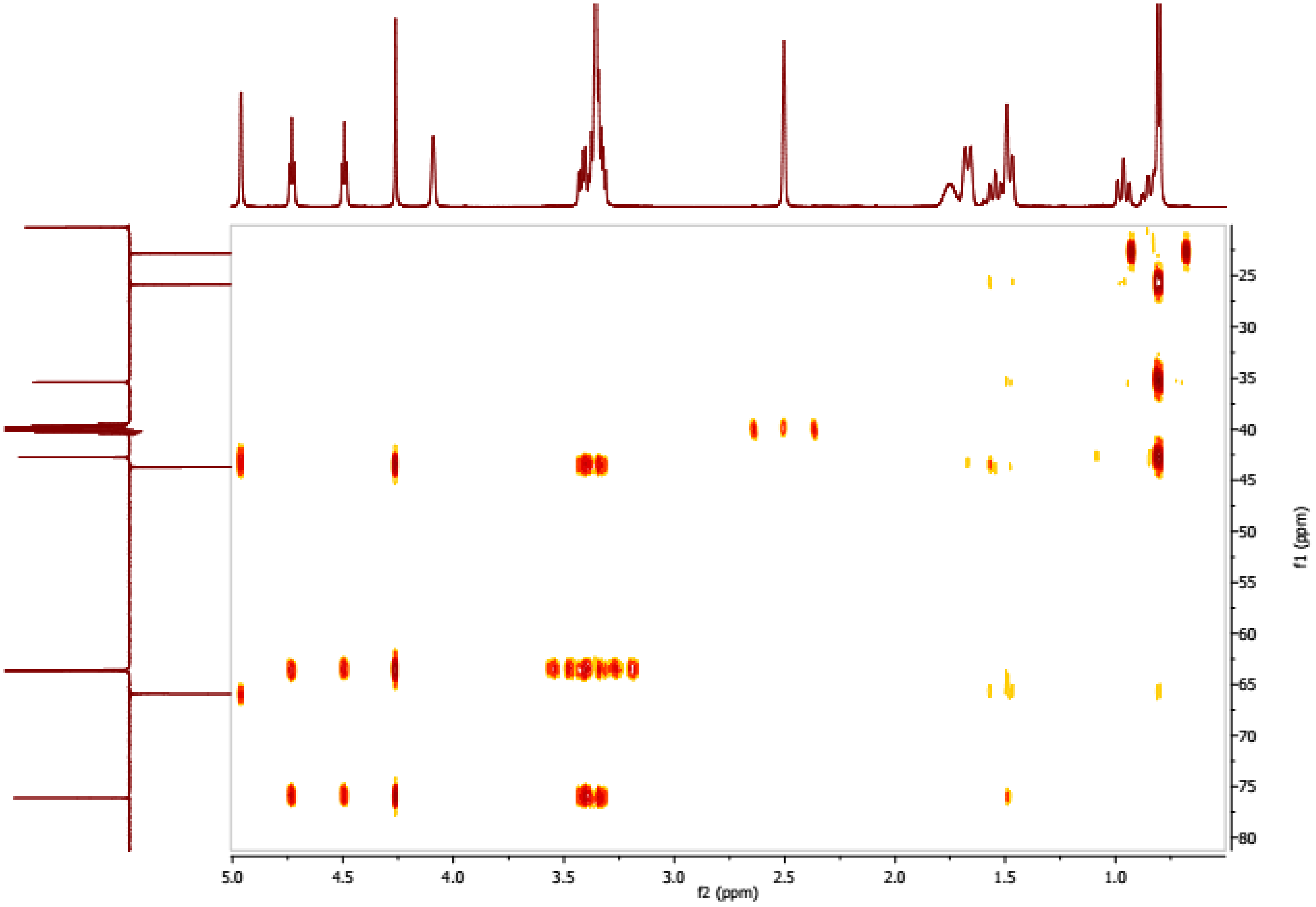

Supplement: Supplementary file 1 [file ijms-20-04050-s001.pdf]
